# Supplementary material for: Synthetic neuromorphic computing in living cells
Source: Nat Commun. 2022 Sep 24;13:5602. doi: 10.1038/s41467-022-33288-8 (PMC9509348; doi:10.1038/s41467-022-33288-8)
Supplement: Supplementary file 1 — Supplementary Information [file 41467_2022_33288_MOESM1_ESM.pdf]

Supplementary Notes for

Synthetic neuromorphic computing in living cells

Luna Rizik†, Loai Danial†, Mouna Habib†, Ron Weiss and Ramez Daniel*

†These authors contributed equally to this work
Correspondence to: ramizda@bm.technion.ac.il

1. Perceptual computing models

A single layer of the artificial neural networks (ANN) receives multiple linear-scale analog inputs (-∞, +∞). This network (Supplementary Fig. 2): (1) multiplies each input x_i by its corresponding analog scalar n_i, which represents the synaptic weight, (2) sums the multiplication products, $y = \sum x_i n_i$, and (3) contains a non-linear activation function, which is commonly described by a sigmoid function $z = \frac{e^{y/K}}{1+e^{y/K}}$, $z \in [0,1]$. This neural model is known as the *perceptron* [1]. Asymptotically, the node or the activation function acts as a decision-making function that determines the digital levels corresponding to the analog inputs.

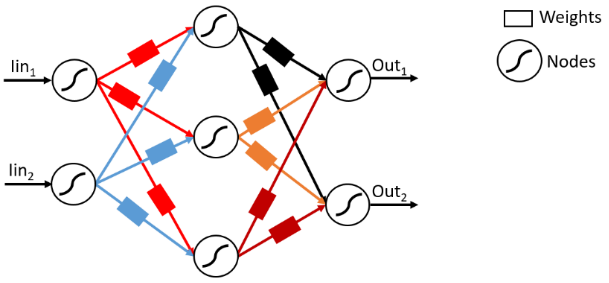

Supplementary Fig. 1. An artificial neural network. The non-linear digital elements in the network are called artificial neurons, and are represented as nodes within the graphical abstraction of the network. The strength of each analog signal is called an artificial synapse (weight) and is represented by an edge. The interactions between nodes through the weights lead to the global behavior of the network.

The simulation results of perceptron including the analog signal and the perceptron output signal are shown in Supplementary Fig 2. We present the results in the 2D contour curve and the 3D surface curve. Every method has its benefits. For example, it is simpler to present the analog pattern in 2D contour compared to the 3D surface curve. By contrast to the perceptron output signal, it is better illustrated in the 3D surface curve.

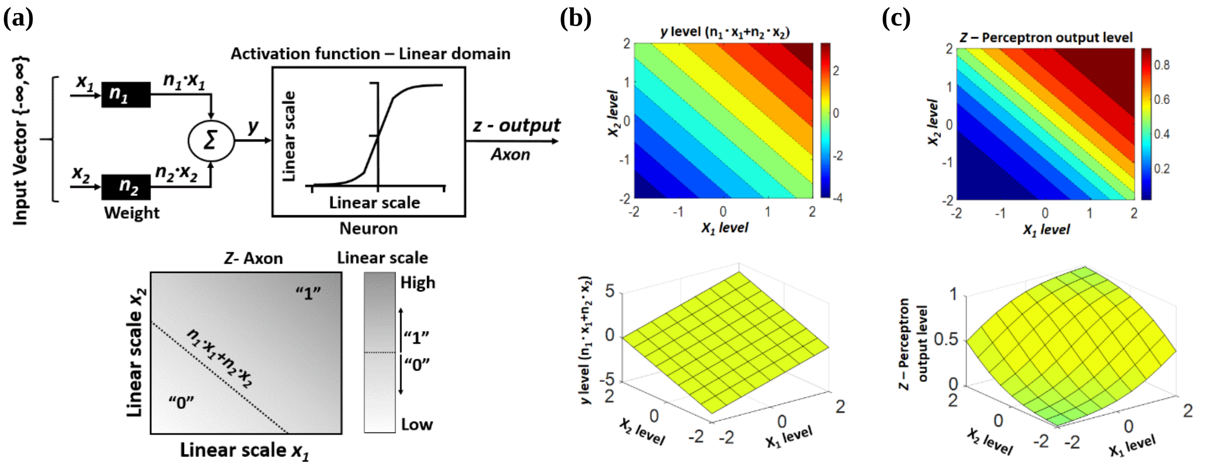

Supplementary Fig. 2. (a) Anatomical structure of abstract perceptron model. The proposed model receives analog inputs processed by analog-weighted elements that collectively interact through non-linear nodes to make an assertive decision. The perceptron model operates in the linear domain, which is widely used as the neuro-processing core in artificial neural networks. **(b)** Simulation results of the analog signal ($n_1x_1 + n_2x_2$) in 2D contour curve and 3D surface curve. **(c)** Simulation results of the perceptron signal in 2D contour curve and 3D surface curve.

Two models of perception can be considered in this work, the perceptgene model (Supplementary Fig. 3a) and Michaelis-Menten (MM)-based perceptron model (Supplementary Fig. 3b). Both models include the bindings between transcription factors (TFs) and DNA, with promoter activities modeled as activation functions. The perceptgene model is a logarithmic transformation of the perceptron and it operates in the logarithmic domain. The MM-based perceptron

model is similar to the perceptron and operates in the linear domain. The MM-based perceptron model is advantageous in its simple design. For example, the summation will be implemented by expressing common proteins by the inputs [2], and the weight n_i is represented as the affinity at ribosome-binding site [2]. Our analysis showed that such a model requires a much higher Hill coefficient (m) to operate than the perceptgene model.

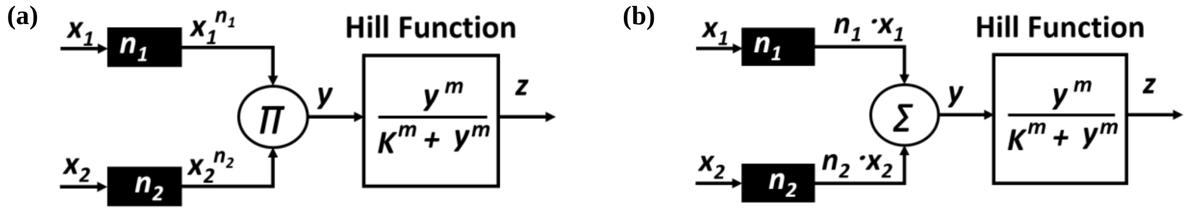

Supplementary Fig. 3. (a) Perceptgene model, **(b)** Michaelis-Menten (MM)-based perceptron model.

The two systems accept two inputs in the range of $x_L < x_1, x_2 < x_H$. For simplicity, we assume that $n_1 = n_2 = n$, and the basal level *(β)* of every promoter is very low. As in Madar et al. [3] and illustrated in Supplementary Fig. 4, for systems that can be described by a Hill function $\frac{y^m}{K^m+y^m}$, we define the output dynamic range (ODR)as the difference between the 90% and 10% of the maximal output z_{max} and the input dynamic range (IDR) as the ratio of the input concentrations required for 90% and 10% of the maximal output. For simplicity, we assume that $z_{max} = 1$.

1.1. Perceptgene model

For low-value inputs $x_1 = x_2 = x_L$, we get:

$y_L = {x_L}^n \cdot {x_L}^n$

$z_L = \frac{{y_L}^m}{K^m+{y_L}^m} = 0.1$

$$\Rightarrow \quad n \cdot m \cdot \log(x_L) = 0.5 \cdot \log\left(\frac{1}{9}\right) + \frac{m}{2} \cdot \log(K) \qquad (1.1)$$

For high value inputs $x_1 = x_2 = x_H$, we get:

$y_H = {x_H}^n \cdot {x_H}^n$

$z_H = \frac{{y_H}^m}{K^m+{y_H}^m} = 0.9$

$$\Rightarrow \quad n \cdot m \cdot \log(x_H) = 0.5 \cdot \log(9) + \frac{m}{2} \cdot \log(K) \qquad (1.2)$$

The $\boldsymbol{IDR}$ can be expressed as (Supplementary Fig. 4):

$$IDR = \log(x_H) - \log(x_L) \qquad (1.3)$$

Substituting Eq. 1.1 and Eq. 1.2 into Eq. 1.3, the IDR of perceptgene is given by:

$$IDR = \frac{1}{n \cdot m} \cdot 0.5 \cdot \log(81) \approx \frac{1}{n \cdot m} \qquad (1.4)$$

1.2. Michaelis-Menten (MM)-based perceptron model

For low-value inputs $x_1 = x_2 = x_L$, we get:

$y_L = n \cdot x_L + n \cdot x_L$

$z_L = \frac{{y_L}^m}{K^m+{y_L}^m} = 0.1$

$$\Rightarrow \quad m \cdot \log(x_L) = \log\left(\frac{1}{9}\right) + m \cdot \log(K) - m \cdot \log(2) - m \cdot \log(n) \qquad (1.5)$$

For high-value inputs $x_1 = x_2 = x_H$, we get:

$y_H = n \cdot x_H + n \cdot x_H$

$z_H = \frac{{y_H}^m}{K^m+{y_H}^m} = 0.9$

$$\Rightarrow \quad m \cdot \log(x_H) = \log(9) + m \cdot \log(K) - m \cdot \log(2) - m \cdot \log(n) \qquad (1.6)$$

Substituting Eq. 1.5 and Eq. 1.6 into Eq. 1.3, the IDR of MM-based perceptron is given by:

$$IDR = \frac{\log(81)}{m} \approx \frac{2}{m} \qquad (1.7)$$

The Hill coefficient values $(\boldsymbol{m}, \boldsymbol{n})$ of synthetic biological parts often are between 1 – 2 [2,4]. Therefore, the $\boldsymbol{IDR}$ of the MM-based perceptron is approximated as 1.333 fold (for $m = 1.5$), and the $\boldsymbol{IDR}$ of perceptgene is approximated as 0.667 fold (for $n = 1, m = 1.5$) and 0.333 fold (for $n = 2, m = 1.5$), respectively. Therefore, Eq. 1.4 and Eq. 1.7 show that the MM-based perceptron model requires a higher value of $\boldsymbol{m}$ than the perceptgene model to operate.

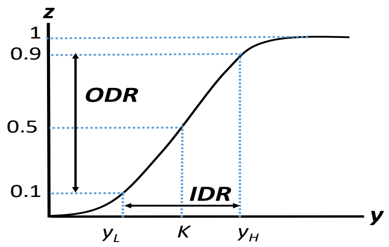

Supplementary Fig. 4. Definition of Input Dynamic Range and Output Dynamic Range.

Supplementary Figs. 5 and 6 show the simulation results of the perceptgene and the MM-based perceptron models for n=1 and 0.5, respectively. The MM-based perceptron model fails to act as a binary classifier (there is no clear separation between "0" and "1" states). By contrast, the perceptgene shows a clear separation between "0" and "1" states.

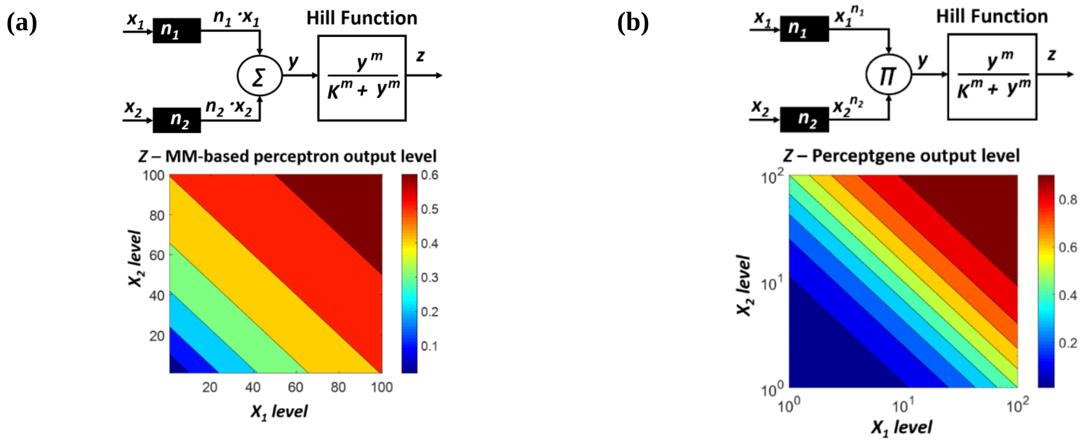

Supplementary Fig. 5. (a) Michaelis-Menten (MM)-based perceptron model combines linear operations and biochemical reactions. The model operates in the linear domain. Simulation results of the MM-based perceptron model, with $n_1 = n_2 = 1, m = 1$. **(b)** Simulation results of the perceptgene model, with $n_1 = n_2 = 1, m = 1$.

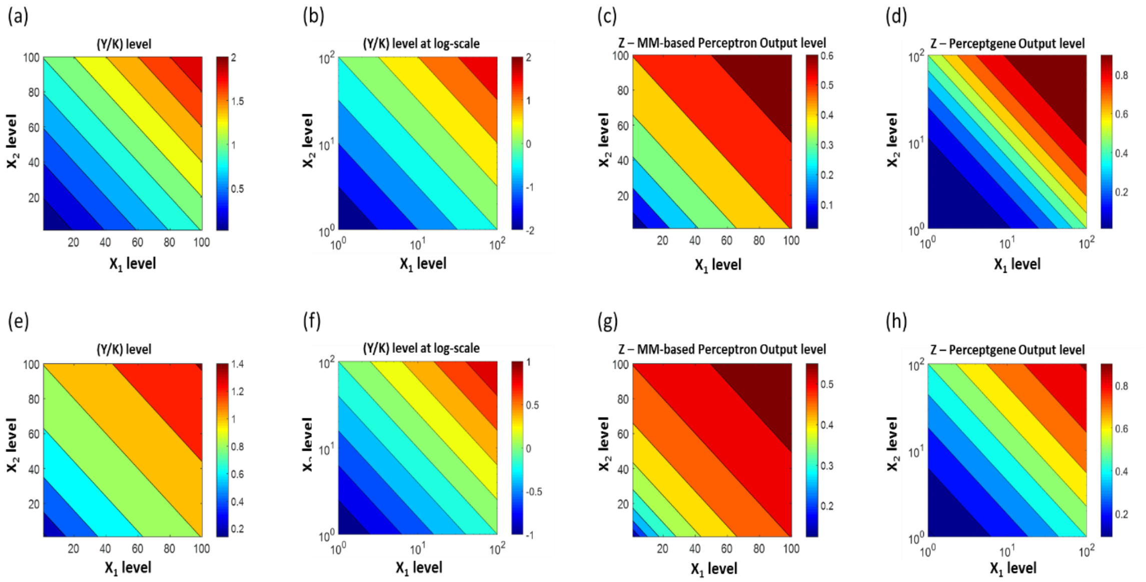

Supplementary Fig. 6. Simulation results of **(a)** Analog signal (Y/K) for MM-based perceptron model, $n = 1$. **(b)** Analog signal (Y/K) for perceptgene model, $n = 1$. **(c)** Output signal for MM-based perceptron model, $n = 1$. **(d)** Output signal for perceptgene model, $n = 1$. **(e)** Analog signal (Y/K) for MM-based perceptron model, $n = 0.5$. **(f)** Analog signal (Y/K) for perceptgene model, $n = 0.5$. **(g)** Output signal for MM-based perceptron model, $n = 0.5$. **(h)** Output signal for perceptgene model, $n = 0.5$. In all the simulations, we assumed that $m = 1$.

1.3. 3D-Plane: Simulation of perceptron and perceptgene models include basal level

The perceptron model includes MM as the activation function failed to classify the analog pattern into two non-linear levels even for a high hill coefficient in the activation function

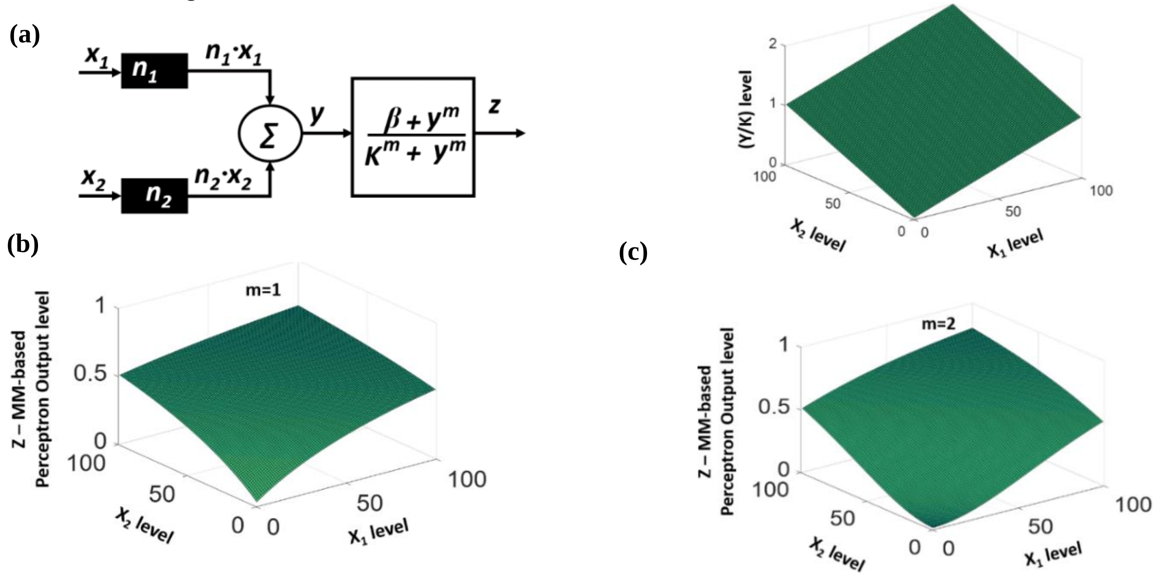

Supplementary Fig. 7. Simulation results of **(a)** Analog signal (Y/K) for MM-based perceptron. **(b)** Output signal for modified perceptron model, $m = 1$. **(c)** Output signal for modified perceptron model, $m = 2$. In all the simulations, we assumed that $n_1 = n_2 = 1$, $\beta = 0.01$.

The perceptgene succeed to classify the analog pattern into two non-linear levels even for low Hill coefficient in the activation function

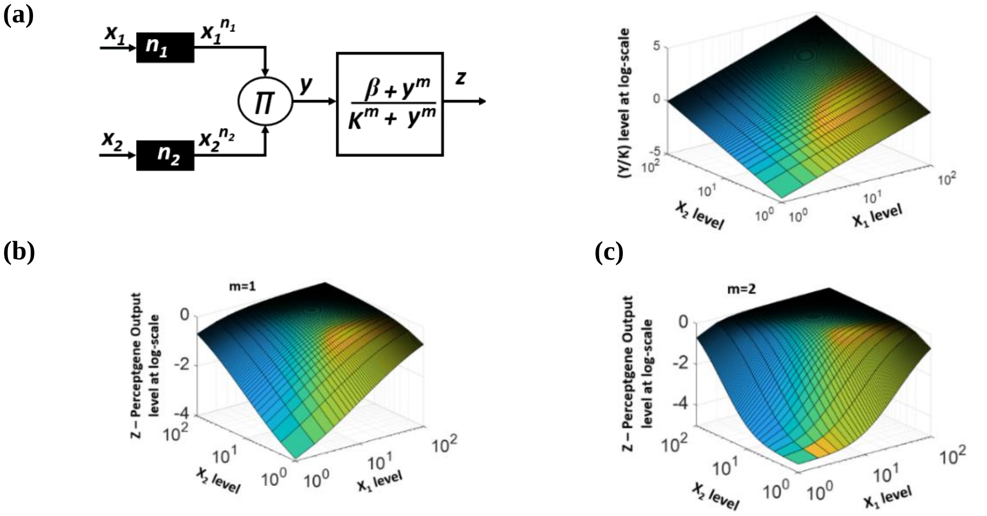

Supplementary Fig. 8. Simulation results of **(a)** Analog signal (Y/K) for perceptgene. **(b)** Output signal for perceptgene model, $m = 1$. **(c)** Output signal for perceptgene model, $m = 2$. In all the simulations, we assumed that $n_1 = n_2 = 1$, $\beta = 0.01$.

1.4. Sensitivity analysis for perceptgene circuit

Sensitivity measures the fold change in the output as a function of the fold change in the input and is given by:

$$S_{Out-In} = \frac{\Delta Out/\langle Out\rangle}{\Delta In/\langle In\rangle} \quad (1.8)$$

Where In is the input and Out is the output (Supplementary Fig. 9a). "< >" denotes the mean of the signal. In this section, we analyze the signals' sensitivity that propagate through the perceptgene (Supplementary Fig. 9b). The signals are given by:

$$y = B \cdot \left(\frac{x}{IDR}\right)^n + y_0 + \sigma_y \quad (1.9.1)$$

$$z = Z_{max} \cdot \frac{y^m}{y^m + K^m} + z_0 + \sigma_z \quad (1.9.2)$$

Where B is the bias with concentration units, $\boldsymbol{IDR}$ is the input dynamic range, x is the perceptgene input (unitless), y_0 is the background signal, z_0 is the promoter basal level, n and m are Hill-coefficients, σ_y and σ_z are random numbers, Z_{max} is the maximum protein expressed in the system.

We calculate the sensitivity of three systems (Supplementary Fig. 9b):

- An analog system maps the analog input signal (x) to collective weighted analog output signal (y) using a power-law and multiplication function:

  $$S_{y-x} = \frac{\Delta y/\langle y\rangle}{\Delta x/\langle x\rangle} \quad (1.10)$$

- A digital system maps analog signal (y) to output levels (z) with a sigmoidal activation function:

  $$S_{z-y} = \frac{\Delta z/\langle z\rangle}{\Delta y/\langle y\rangle} \quad (1.11)$$

- A neuromorphic system combines the analog and digital systems:

  $$S_{z-x} = \frac{\Delta z/\langle z\rangle}{\Delta x/\langle x\rangle} \quad (1.12.1)$$

  $$S_{z-x} = \frac{\Delta z/\langle z\rangle}{\Delta y/\langle y\rangle} \cdot \frac{\Delta y/\langle y\rangle}{\Delta x/\langle x\rangle} \quad (1.12.2)$$

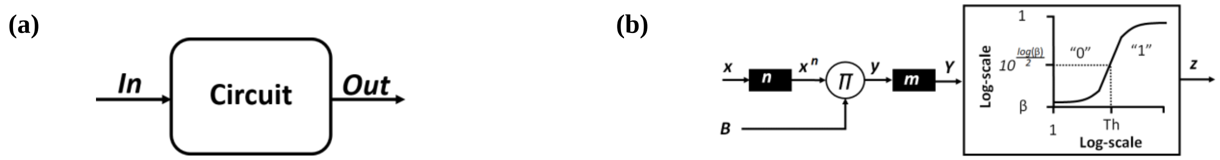

Supplementary Fig. 9. (a) A circuit that has an input In and output Out. **(b)** Perceptgene with one input (x). (y) is the collective analog signal and (z) is the output.

Using Eq. 1.9.1 and 1.9.2, we get:

$$\frac{\Delta y}{<y>} = n \cdot (1 - \frac{y_0}{<y>}) \cdot \frac{\Delta x}{<x>} \tag{1.13.1}$$

$$\frac{\Delta z}{<z>} = m \cdot (1 - \frac{z_0}{<z>}) \cdot (1 - \frac{<z> - z_0}{z_{max}}) \cdot \frac{\Delta y}{<y>} \tag{1.13.2}$$

Therefore, according to the definition of sensitivity, we get:

$$S_{y-x} = n \cdot (1 - \frac{y_0}{<y>}) \tag{1.14.1}$$

$$S_{z-y} = m \cdot (1 - \frac{z_0}{<z>}) \cdot (1 - \frac{<z> - z_0}{z_{max}}) \tag{1.14.2}$$

$$S_{z-x} = n \cdot m \cdot (1 - \frac{y_0}{<y>}) \cdot (1 - \frac{z_0}{<z>}) \cdot (1 - \frac{<z> - z_0}{z_{max}}) \tag{1.14.3}$$

Our simulation results (Supplementary Fig. 10) based on the set of equations 1.14.1-1.14.3 show the influence of the background level (y_0) on the analog, digital and neuromorphic systems and their sensitivity. When y_0 increases, the effective input dynamic range (IDR_{eff}) decreases (Supplementary Fig. 10a). Based on Eq. 1.9.1 when $y_0 = 0$, the $IDR_{eff} = IDR$, meaning that the effective (actual) input dynamic range is equal to the theoretical input dynamic range. While increasing the background level decreases the sensitivity of the analog system (S_{y-x}, Supplementary Fig. 10d), it almost does not affect the sensitivity of the neuromorphic system (S_{z-x}, Supplementary Fig. 10f). More interestingly, the sensitivity of the neuromorphic system (S_{z-x}, Supplementary Fig. 10f) is higher than the sensitivity of the digital system (S_{z-y}, Supplementary Fig. 10e). These results strongly depend on the value of basal promoter level (z_0). In case where $z_0 = 0$, the digital system's sensitivity can be higher than the sensitivity of the neuromorphic system. However, since synthetic biological parts always have basal levels, we expect that the neuromorphic system's sensitivity will not be affected by the background level as compared to the digital system. Therefore, in conclusion, the main contribution of increasing the background level (y_0) on the performance of a neuromorphic (perceptgene) system is decreasing the input dynamic range.

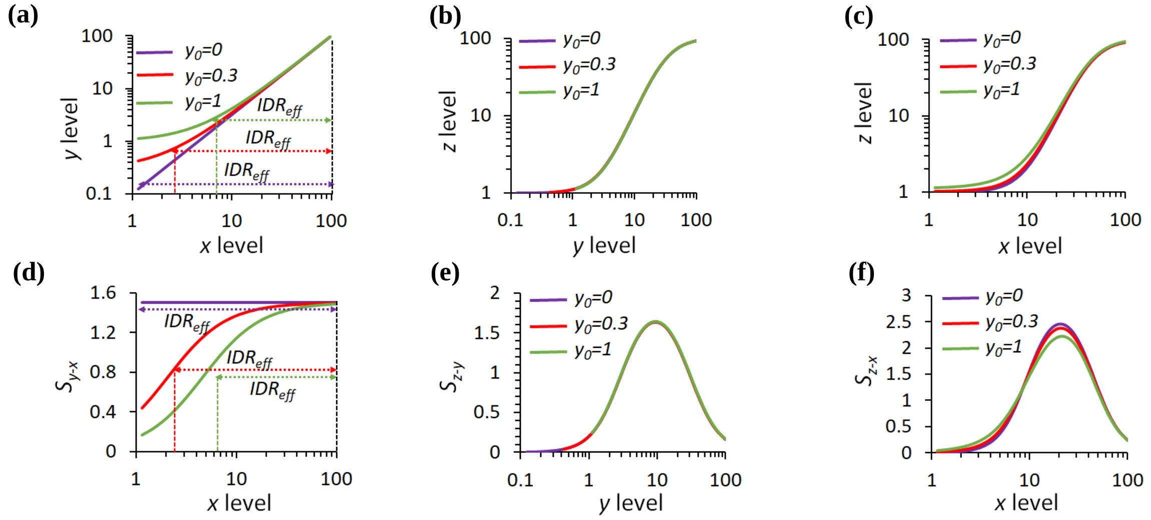

Supplementary Fig. 10. Simulation results show the influence of the background level (y_0) on the signals and sensitivity of perceptgene. **(a)** Simulation results of a weighted and collective analog signal (y) versus input level (x). **(b)** Simulation results of the output signal (z) versus collective signal (y). **(c)** Simulation results of the output signal (z) versus input signal (x). **(d)** Sensitivity of the analog system (S_{y-x}), where x level is the input and y is the output. **(e)** Sensitivity of the digital system (S_{z-y}), where y is the input and z is the output. **(f)** Sensitivity of the neuromorphic system (S_{z-x}), where x is the input and z is the output. IDR_{eff} is the effective input dynamic range when the background signal (y_0) is higher than zero. Simulation parameters: $n = 1.5, IDR = 100, B = 100, Z_{max} = 100, m = 2, z_0 = 1$.

In summary: The analog system maps input levels to output levels using a power-law and multiplication function. The digital system maps input levels to output levels with a sigmoidal activation function in the logarithmic domain. The neuromorphic system combines analog and digital systems to carry out perceptgene computation. Our analysis shows that the analog system's sensitivity is reduced significantly with an increase in basal expression, meaning that analog operation in the low concentration regime is highly prone to errors. The digital system is designed to be insensitive in the low concentration regime, and hence less affected by an increase in basal expression. The neuromorphic system combines the best features of both, namely the ability to perform analog computation while being insensitive to the low concentration regime's noisy aspects, as shown in the sensitivity analysis (Supplementary Fig. 10).

1.5. Noise analysis for analog signals in perceptron

Here we will evaluate the noise that generates during the signal aggregation in genetic perceptron. If we assume that their "l" signals that aggregate and each of them has a noise of ΔX, then the system should satisfy two boundary conditions:

1. $l \cdot \Delta X_L = \overline{X_L}$ (1.15.1)
2. $\Delta X_H = \overline{X_L}$ (1.15.2)

Where X_L is defined as a low signal and X_H is defined as a high signal. In the first condition, we request that the total noise that is generated by the "l" signals is smaller than the low signal X_L. In the second condition, we inquire that the noise that is generated by the high signal (X_H) is smaller than the low signal itself. Signals often originate from the transport of discrete random carriers in systems; in biology, it is the diffusion of biochemical molecules and proteins. Naturally, these signals propagate with random fluctuations inside the networks. These fluctuations follow the Poisson process and generate shot noise that scales as the square root of the molecular count. Typically, there are two orthogonal sources of noise in any biological system [5,6]. The first source is the intrinsic noise, which is inherently generated by the system itself. The second source is the extrinsic noise, which is generated by random fluctuations in the input or another environmental parameter. Here we consider the influence of the intrinsic noise only on the perceptron. A stochastic model for intrinsic cellular noise may be greater than the Poisson process, with the addition of burst size ($\boldsymbol{b_{int}}$) is given by [7]:

$$\Delta X_L = \sqrt{(1 + b_{int}) \cdot \overline{X_L}} \quad (1.16.1)$$

$$\Delta X_H = \sqrt{(1 + b_{int}) \cdot \overline{X_H}} \quad (1.16.2)$$

Substituting Eq. 1.15.1 into Eq.1.16.1 and Eq. 1.15.2 into Eq.1.16.2, we obtain:

$$l \cdot \sqrt{(1 + b_{int}) \cdot \overline{X_L}} = \overline{X_L} \quad \Rightarrow \quad l = \sqrt{\frac{\overline{X_L}}{(1+b_{int})}} \quad (1.17.1)$$

$$\sqrt{(1 + b_{int}) \cdot \overline{X_H}} = \overline{X_L} \quad \Rightarrow \quad \overline{X_H} = \frac{\overline{X_L}^2}{(1+b_{int})} \quad (1.17.2)$$

Therefore, there is a tradeoff between number of inputs and system accuracy.

Supplementary Table 1: The relations between the maximum number of inputs that are allowed and burst size in perceptro

X_L	b_{int}	Maximum l	Maximum X_H
10	0	~3	100
100	0	10	10000
10	3	1	25
100	3	5	2500
100	9	3	1000
1000	9	10	100000

The burst size relies on the translation rate, the number of amino acids (aa) in the synthesized protein, and mRNA half time. Typically, in *Escherichia coli*, the translation rate ranges between 10-20 aa/sec, depending on growth condition [8], and mRNA half time is around 3-5 min [8]. Therefore, the burst size in *Escherichia coli* can be ranged between 3-15. However, the main limitation for scaling the perceptgene beyond 2 inputs, is the construct itself.

1.6. NonLinearity degree

To quantify the nonlinearity degree of gene circuits with the forms of Hill-functions:

$$P_z = \frac{\left(\frac{Y}{K_d}\right)^m + \beta}{1+\left(\frac{Y}{K_d}\right)^m} \qquad (1.18)$$

where β is the basal level of the promoter, K_d is the dissociation constant of binding Y to the promoter, and m is the Hill-coefficient), similar to the circuits that developed in this study, we define a new parameter (NonLinearity) that is given by:

$$\text{NonLinearity} = \frac{\text{m}}{\log(MFC)/\log(IR)} \qquad (1.19)$$

Where, *MFC* is the maximum fold change and it is given by (1/ β), and *IR* is the input range and is given by Y_H/Y_L (Supplementary Fig. 11a). Hill-coefficient is equal to the slope of the log(*Input*) and the log(*Output*) operating around K_d, in the range $\beta \leq \frac{Y}{K_d} \leq 1$. Applying a *log* operation to Eq. 1.18:

$$dlog(P_z) = \log\left(\left(\frac{Y}{K_d}\right)^m + \beta\right) - \log\left(1 + \left(\frac{Y}{K_d}\right)^m\right) \qquad (1.20)$$

In the range $\beta \leq \frac{Y}{K_d} \leq 1$:

$$dlog(P_z) = \log\left(\left(\frac{Y}{K_d}\right)^m\right) - \log(1) \qquad (1.21)$$

Therefore, the Hill-coefficient (m) can be calculated by:

$$m = \frac{dlog(P_z)}{dlog(Y/K_d)} \qquad (1.22)$$

The term is equal to the slope of a linear line at the log scale that spans between the (Y_L, β) and (Y_H, *1*). The Nonlinearity compares slopes of Hill-function (m) and a linear line at a log scale when both functions have the same input range and maximum fold change. Supplementary Fig. 11b demonstrates the NonLinearity of linear function with NonLinearity=1. Supplementary Fig. 11c demonstrates the NonLinearity of step function with NonLinearity>>1.

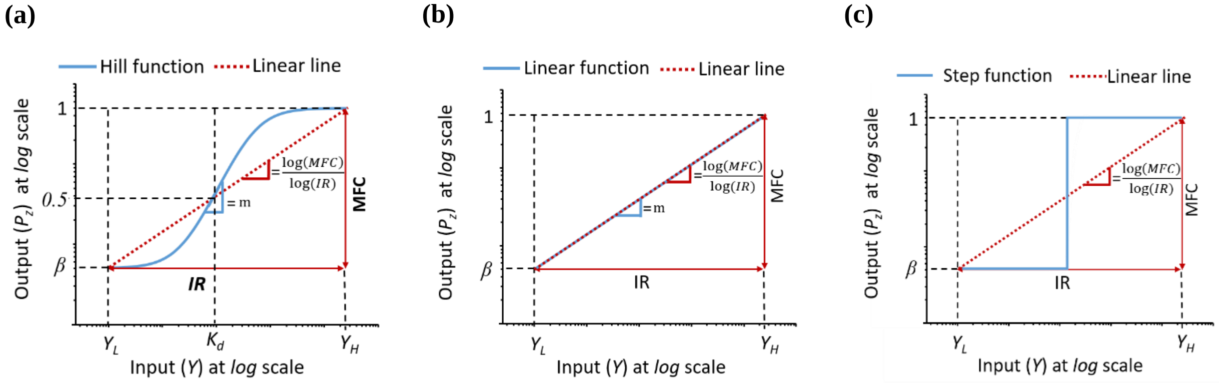

Supplementary Fig. 11. Definition of NonLinearity degree for **(a)** Hill-function, **(b)** linear function, and **(c)** Step function with two-discrete binary states.

We then explored how K_d *and m* parameters can affect the NonLinearity degree of Hill-function (Supplementary Fig. 12)

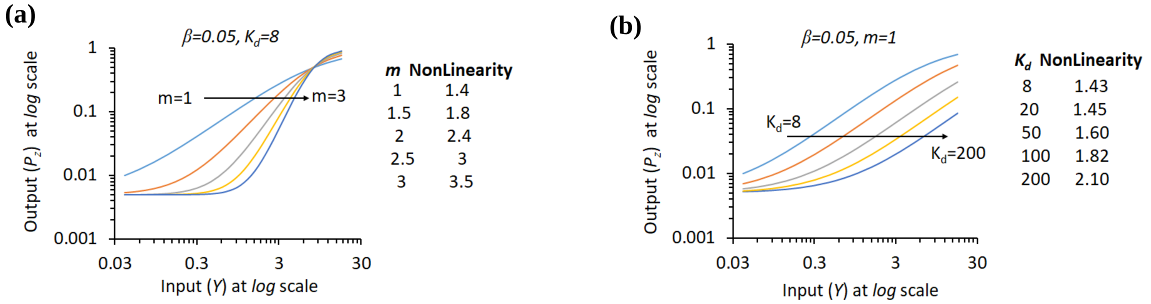

Supplementary Fig. 12. (a) The influence of Hill coefficient (*m*) on the NonLinearity, **(b)** The influence of dissociation constant (K_d) on the NonLinearity.

In electronics, ANNs outperform the conventional computing paradigms (e.g., digital and analog) in a variety of settings (such as classification and signal processing [60–64]) owing to their collective resilient properties. ANNs perform efficient execution of complex functions by utilizing a low number of components similar to analog design and producing reliable results similar to digital circuits (e.g. majority function, Supplementary Notes, Section 5, ADC, Supplementary Notes, Section 7). Implementing ANNs in a biological setting requires an important change to obtain the same performance benefits. Specifically, using logarithmic rather than linear functions is often more appropriate for describing biochemical reactions in gene regulation (e.g., Hill Functions that describe dosage response curves, Weber's law [2,44,65]). The use of logarithmic functions for ANNs places particular requirements on properties of gene regulatory elements in terms of their Hill coefficients, basal and maximal protein expression levels, and transcription factor dissociation constants. These requirements can be met 'easily'. Furthermore, we have shown that common biochemical reactions can be simply converted to perceptgene units (Design principles of neuromorphic circuits, Supplementary Notes, Section 8).

In comparison, a linear genetic implementation would have been based on less reliable parts, for example, we have shown in Section 1 that computation based on the perceptron requires an activation function with a very high Hill-coefficients (>2.5). Such values are very challenging to obtain in synthetic and natural biological systems. Therefore, linear-based ANNs computing would place much more stringent requirements on gene regulatory elements' properties and necessitated a more complex design to achieve the same performance. Furthermore, the logarithmic domain is also more appropriate for attenuating the effects of typical fluctuations in protein expression levels. Subsequently, it provides a more resistant platform for neuromorphic computing

in a gene regulation context. The essence here is the reliance on fold-change regulation, as opposed to absolute-change regulation, with the former being more appropriate for genetic circuits (as previously articulated in the community, e.g., by Uri Alon [66]).

Table 2 List of parameters used in this section

Symbol	Description
m	Hill coefficient of binding transcription factor to promoter
n	Hill coefficient of binding inducer to transcription factor
x_i	Inputs
y	Summation or multiplication of inputs (analog signal)
z_{max}	Maximal output
β	Basal level
S	Sensitivity
K_d	Dissociation constrant
P	Promoter activity

Table 3 List of abbreviations used in this section

Symbol	Description
MM	Michaelis-Menten
TFs	Transcription factors
IDR	Input dynamic range
ODR	Output dynamic range

2. Biophysical models and analysis of single perceptgene networks

In the following sections, we model the genetic network motifs using biophysical models at steady state *d/dt=0.* Our models involve detailed biochemical reactions, such as the bindings between inducers and transcription factors (TFs), as well as between TFs and promoters that consist of multiple binding sites. Our models focus on the effects of negative and positive feedback loops. These detailed biochemical models can accurately capture the behavior of the various proposed circuit topologies by solely changing the parameters that are expected to vary between experiments (e.g., plasmid copy number). In the models, we assume that the concentration of chemical species is uniformly distributed and the behaviors of our genetic circuits can be analyzed at the steady states.

2.1. Model of auto-negative feedback (ANF) loops and combinatorial promoter

In this section, we present a model of ANF loops with a combinatorial promoter (Supplementary Figs. 13 and 14a), which yields the loops' behavior resembling a power-law and multiplication function. But first, we show that the experimental results of this circuit fit the power-law and multiplication function.

Fitting experimental results of ANF loops and $\mathrm{P}_{\mathrm{lacO/tetO}}$ -based combinatorial promoter to power-law and multiplication function: $\left(\frac{IPTG}{1.25}\right)^{0.3375} \cdot \left(\frac{IPTG}{0.7}\right)^{0.4375}$

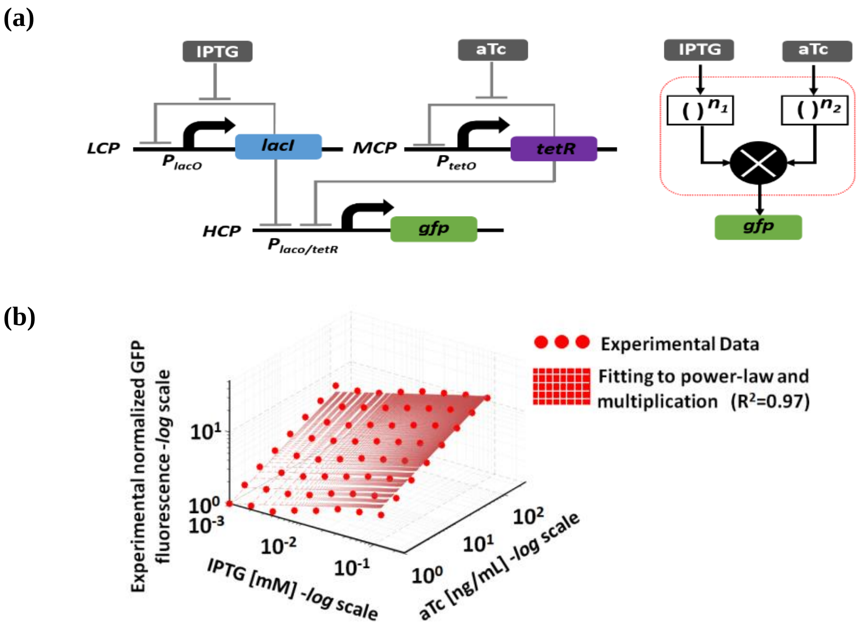

Supplementary Fig. 13. (a) Implementation of power-law and multiplication function based on $P_{lacO/tetO}$ promoter through ANF loops. **(b)** Fitting the experimental results of P_{lacO} and P_{tetO} ANF loops and combinatorial promoter ($P_{lacO/tetO}$- GFP) to power-law and multiplication function $\left(\frac{IPTG}{1.25}\right)^{0.3375} \cdot \left(\frac{aTc}{0.7}\right)^{0.26}$. The data appears in Fig. 1c in the main text and is reproduced here for clarity.

The combinatorial promoter in this section includes two binding sites with different repressors. In this system, we assume that:

- The Hill coefficient of binding R_1 repressor to $P_{\ 1}$(promoter within ANF) is equal to the Hill coefficient of binding R_1 to $P_{\ 1/2}$(combinatorial promoter), n_1
- The Hill coefficient of binding R_2 repressor to P_2 (promoter within ANF) is equal to the Hill coefficient of binding R_2 to $P_{1/2}$ (combinatorial promoter), n_2
- The binding affinity of R_1 repressor to P_1 (promoter within ANF) is equal to binding affinity to $P_{1/2}$ (combinatorial promoter), K_{d1}
- The binding affinity of R_2 repressor to P_2 (promoter within ANF) is equal to binding affinity to $P_{1/2}$ (combinatorial promoter), K_{d2}
- The Basal levels (β_i) of P_1 , P_2 and $P_{\ 1/2}$ are very low.

The binding of TFs to promoters is modeled according to the Shea-Ackers formalism [9,10]. Therefore, the total level of expressed R_{Ti} $(i = 1,2)$ repressors in the case of ANF loops can be expressed as:

$$R_{Ti} = R_{max\,i} \frac{1}{1+\left(\frac{R_i}{K_{di}}\right)^{n_i}} \qquad (2.\ 1)$$

Where R_{maxi} is the maximum protein level achieved by P_i which is proportional to (transcription rate × translation rate) × (mRNA half-life × protein half-life). R_i is the level of repressors that are bound to P_i. The induction of the repressors by x_i inducers is given by:

$$R_i = R_{Ti} \cdot f_i(x_i) \qquad (2.\ 2)$$

$$f_i(x_i) = \frac{1}{1+\left(\frac{x_i}{K_{mi}}\right)^{h_i}} \qquad (2.\ 3)$$

Where $K_{\ mi}$ dissociation constant and $h_{\ i}$Hill coefficients of binding x_i to R_i. The formed new complex (Inducer-repressor) prevents the repressors from binding to P_1 and P_2. By substituting Eq. 2.1 into Eq. 2.2 we get:

$$\frac{R_i}{K_{di}} \cdot \frac{K_{di}}{f_i(x_i)} = R_{max\,i} \frac{1}{1+\left(\frac{R_i}{K_{di}}\right)^{n_i}} \qquad (2.\,4)$$

By developing Eq. 2.4, we get:

$$\left(\frac{R_i}{K_{di}}\right)^{n_i} = R_{max\,i} \cdot \frac{f_i(x_i)}{Y_i} - 1 \qquad (2.\,5)$$

The binding states for $P_{1/2}$ combinatorial promoter is shown in Supplementary Fig. 14b. The probability for $P_{1/2}$ promoter being in open complex P is described by the following equations[9–11]:

$$P_{1/2} = \frac{1}{1+\left(\frac{R_1}{K_{d1}}\right)^{n_1}+\left(\frac{R_2}{K_{d2}}\right)^{n_2}+\theta\cdot\left(\frac{R_1}{K_{d1}}\right)^{n_1}\cdot\left(\frac{R_2}{K_{d2}}\right)^{n_2}} \qquad (2.\,6)$$

Then, the expression level of the output protein at steady is given by:

$$Y = Y_{max} \cdot \frac{1}{1+\left(\frac{R_1}{K_{d1}}\right)^{n_1}+\left(\frac{R_2}{K_{d2}}\right)^{n_2}+\theta\cdot\left(\frac{R_1}{K_{d1}}\right)^{n_1}\cdot\left(\frac{R_2}{K_{d2}}\right)^{n_2}} \qquad (2.\,7)$$

Where Y_{max} is the maximum protein level achieved by $P_{1/2}$ promoter. In case that the two repressors do not interfere with their bindings to $P_{1/2}$ promoter ($\theta = 1$), we substitute Eq. 2.5 into Eq. 2.7 and get:

$$Y = Y_{max} \cdot \left(\frac{R_1}{R_{max1} \cdot f_1(x_1)}\right) \cdot \left(\frac{R_2}{R_{max2} \cdot f_2(x_2)}\right) \qquad (2.\,8)$$

Substituting Eq. 2.3 into Eq. 2.8, we get:

$$Y = Y_{max} \cdot \left(\frac{R_{T1}}{R_{max1}}\right) \cdot \left(\frac{R_{T2}}{R_{max2}}\right) \qquad (2.\,9)$$

Therefore, in an ANF loop motif that regulates a combinatorial promoter, with $\theta = 1$, the expressed signal is effectively the multiplication of the two repressors. Substituting Eq. 2.2 into Eq. 2.1, we get:

$$R_{Ti} = R_{max\,i} \frac{1}{1+\left(\frac{R_{Ti} \cdot f(x_i)}{K_{di}}\right)^{n_i}}$$

$$R_{Ti} + {R_{Ti}}^{n_i+1} \left(\frac{f(x_i)}{K_{di}}\right)^{n_i} = R_{max\,i}$$

$$R_{Ti} \cdot \left(\frac{K_{di}}{f(x_i)}\right)^{n_i} + {R_{Ti}}^{n_i+1} = R_{max\,i} \cdot \left(\frac{K_{di}}{f(x_i)}\right)^{n_i} \qquad (2.\,10)$$

When K_{di} is very small (high binding affinity between repressors and promoters), we get:

$$R_{Ti} = \left(R_{max\,i}\right)^{1/(n_i+1)} \cdot \left(\frac{K_{di}}{f(x_i)}\right)^{n_i/(n_i+1)} \qquad (2.\,11)$$

Substituting Eq. 2.3 into Eq. 2.11:

$$R_{Ti} = \left(R_{max\,i}\right)^{1/(n_i+1)} \cdot (K_{di})^{n_i/(n_i+1)} \cdot \left(1+\left(\frac{x_i}{K_{mi}}\right)^{h_i}\right)^{n_i/(n_i+1)} \qquad (2.\,12)$$

Substituting Eq. 2.12 into Eq. 2.9: we get:

$$Y = Y_{max} \cdot \left(\frac{\left(R_{max_1}\right)^{1/(n_1+1)} \cdot (K_{d1})^{n_1/(n_1+1)} \cdot \left(1+\left(\frac{x_1}{K_{m1}}\right)^{h_1}\right)^{n_1/(n_1+1)}}{R_{max1}}\right) \cdot \left(\frac{\left(R_{max_2}\right)^{1/(n_2+1)} \cdot (K_{d2})^{n_2/(n_2+1)} \cdot \left(1+\left(\frac{x_2}{K_{m2}}\right)^{h_2}\right)^{n_2/(n_2+1)}}{R_{max2}}\right)$$

$$\Rightarrow \quad Y = Y_{max} \cdot \left(\frac{K_{d1}}{R_{max_1}}\right)^{n_1/(n_1+1)} \cdot \left(\frac{K_{d2}}{R_{max_2}}\right)^{n_2/(n_{21}+1)} \cdot \left(1+\left(\frac{x_1}{K_{m1}}\right)^{h_1}\right)^{n_1/(n_1+1)} \cdot \left(1+\left(\frac{x_2}{K_{m2}}\right)^{h_2}\right)^{n_2/(n_2+1)} \qquad (2.\,13)$$

By applying a logarithmic operation to Eq. 2.13, we get:

$$\log(Y) = \log(Y_{max}) + \frac{n_1}{n_1+1}\cdot log\left(\frac{K_{d1}}{R_{max_1}}\right) + \frac{n_2}{n_2+1}\cdot log\left(\frac{K_{d2}}{R_{max_2}}\right) + \frac{n_1}{n_1+1}\cdot log\left(1+\left(\frac{x_1}{K_{m1}}\right)^{h_1}\right) + \frac{n_2}{n_2+1}\cdot log\left(1+\left(\frac{x_2}{K_{m2}}\right)^{h_2}\right) \quad (2.\ 14)$$

We define:

$$C \equiv \log(Y_{max}) + \frac{n_1}{n_1+1}\cdot log\left(\frac{K_{d1}}{R_{max_1}}\right) + \frac{n_2}{n_2+1}\cdot log\left(\frac{K_{d2}}{R_{max_2}}\right) \quad (2.\ 15)$$

Then, we get:

$$\log(Y) = C + \frac{n_1}{n_1+1}\cdot log\left(1+\left(\frac{x_1}{K_{m1}}\right)^{h_1}\right) + \frac{n_2}{n_2+1}\cdot log\left(1+\left(\frac{x_2}{K_{m2}}\right)^{h_2}\right) \quad (2.\ 16)$$

In case that $x_i/K_{mi} > 1$, the ANF loops and combinatorial promoter circuit act as power-law and multiplication function, **and are linearly separable in the *log-log* scale as follows**:

$$\log(Y) = C + \frac{n_1\cdot h_1}{n_1+1}\cdot log\left(\frac{x_1}{K_{m1}}\right) + \frac{n_2\cdot h_2}{n_2+1}\cdot log\left(\frac{x_2}{K_{m2}}\right) \quad (2.\ 17)$$

Supplementary Eq. 2.17 and Supplementary Fig. 15 show that the coefficients of the power-law functions are set by the cooperativity, number of binding sites and Hill coefficients (n_i *and* h_i). Therefore, in our circuit motif, the cooperativity effectively acts as weights in the perceptgene model, equivalent to synaptic weights in the perceptron model.

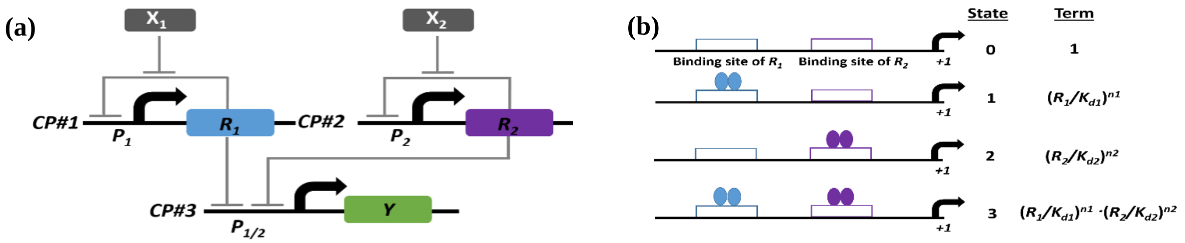

Supplementary Fig. 14. A theoretical model of linearly separable function at the *log-log* scale. **(a)** ANF loops and combinatorial promoter circuit motif, CP#=Copy number of plasmids. **(b)** The binding states of $P_{1/2}$ hybrid promoter.

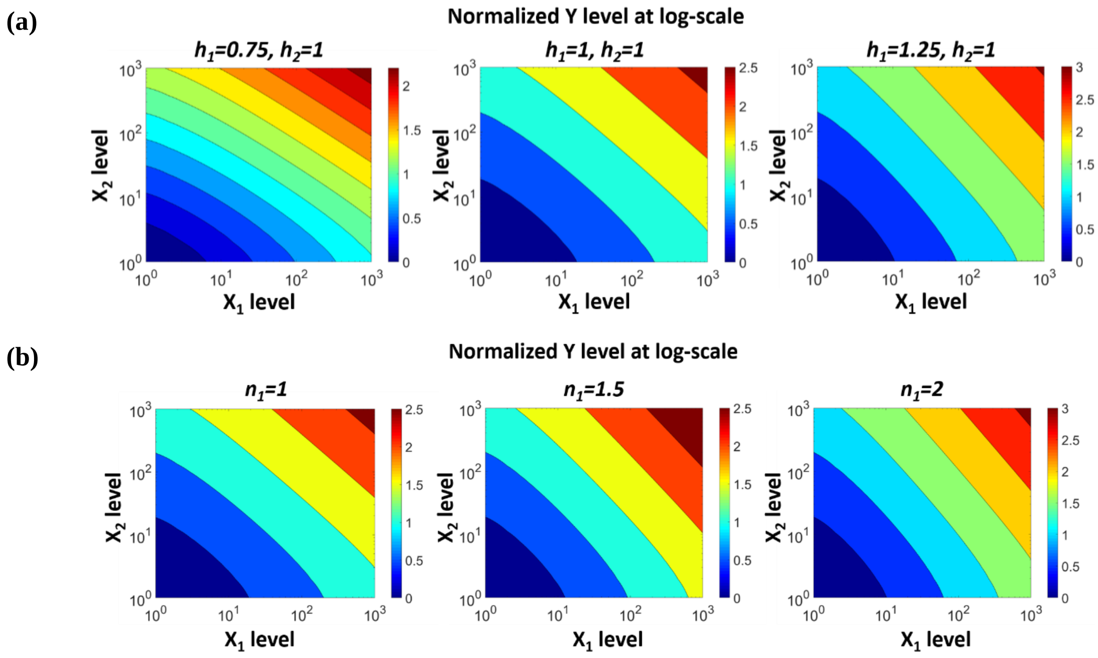

Supplementary Fig. 15. Theoretical results of ANF loops and combinatorial promoter using Eq. 2.3. **(a)** Effect of h: $K_{m1} = 1, K_{m2} = 1, K_{d1} = 1, K_{d2} = 1, R_{max1} = 1000, R_{max2} = 1, n_1 = 1, n_2 = 1$. **(b)** Effect of n: $K_{m1} = 1, K_{m2} = 1, K_{d1} = 1, K_{d2} = 1, R_{max1} = 1000, R_{max2} = 1, n_2 = 1$.

Then we tested the influence of other biophysical and design parameters on the behavior of the system as shown in Supplementary Fig. 16. We expanded our models to include other mechanisms such as the basal levels (β_i) of promoters, as well as the asymmetry between the combinatorial promoter ($P_{1/2}$) and ANF promoters (P_1 & P_2) :

- ANF loops that are based on Eq. 2.1 and include β_i:

$$R_{Ti} = R_{max\,i} \frac{1}{1+\beta_i+\left(\frac{R_i}{K_{di}}\right)^{n_i}} \quad (2.18)$$

- The induction process that is based on Eq. 2.3 can be described as follows:

$$R_i = R_{Ti} \cdot \frac{1}{1+\left(\frac{x_i}{K_{mi}}\right)^{h_i}} \quad (2.19)$$

- For asymmetric combinatorial promoters, we assume that $K_{d1} \neq K_{d1h}$ and $K_{d2} \neq K_{d2h}$

$$Y = Y_{max} \cdot \frac{1}{1+\left(\frac{R_1}{K_{d1h}}\right)^{n_{1h}}+\left(\frac{R_2}{K_{d2h}}\right)^{n_{2h}}+\theta\cdot\left(\frac{R_1}{K_{d1h}}\right)^{n_{1h}}\cdot\left(\frac{R_2}{K_{d2h}}\right)^{n_{2h}}} \quad (2.20)$$

Supplementary Fig. 16a shows the influence of increasing K_m (the dissociation constant of binding inducer X_i and repressor R_i). When K_m decreases, the input dynamic range increases.
Supplementary Fig. 16b shows the influence of increasing K_{di} (the dissociation constant of binding repressors to P_1 & P_2 promoters, respectively). When K_d increases, the input dynamic range decreases.
Supplementary Fig. 16c shows the influence of increasing K_{dh} (the dissociation constant of binding repressors to $P_{1/2}$ combinatorial promoter), without changing the K_{di} of ANF promoters. Our simulations show that there is a tradeoff between the width of theK_{dh} and the input dynamic range. For example, the input dynamic range with $K_{dh} = 10$ is wider than the input dynamic range with $K_{dh} = 100$. To demonstrate, we cloned the combinatorial promoter on HCP and ANF loops on LCP/MCP, which gave a higher K_{dh} value compared to K_{di}.
Supplementary Fig. 16d shows the influence of increasing h (Hill coefficient of binding inducer to repressors). When h increases, the slope of the log-log scale increases. Therefore, the Hill coefficient acts as a weight, resembling the synaptic weight in the perceptron model. These results match our theoretical model (Eq. 2.23).
Supplementary Fig. 16e shows the influence of increasing n (Hill coefficient of binding R repressor to P_1 & P_2 promoters). When n increases (while n_h of binding repressors to $P_{1/2}$ is constant), the slope of the log-log scale increases. Remarkably the maximum protein level which is achieved by the circuit (R_{max}) has an inverse effect than decreasing K_d.
The interference between the binding of different TFs on combinatorial promoter [11] is represented by θ in Eq. 2.6, ($\theta = 1$ when there is no interference). When the binding between one TF and the combinatorial promoter affects the interaction between other TFs, then $\theta < 1$ (Eq. 2.6). Supplementary Fig. 16f shows the influence of increasing θ on the ANF loops and combinatorial promoter circuit. As in Supplementary Fig. 16f, even for a very low θ, which means a high interference between the two repressors, the input-output transfer function of the circuit motif can be fitted to a power-law and multiplication function.

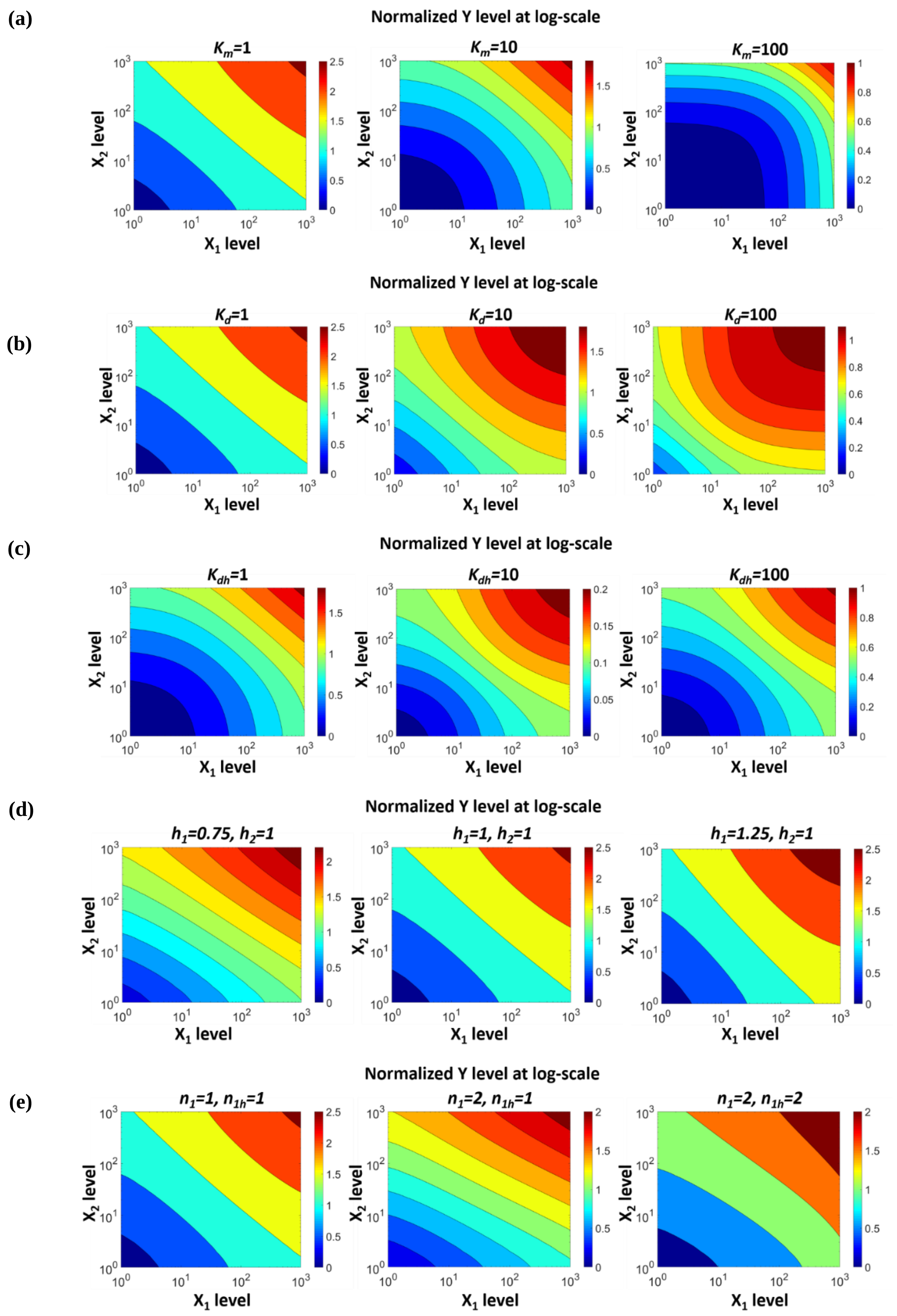
(a)
Normalized Y level at log-scale
Km=1
Km=10
Km=100
(b)
Normalized Y level at log-scale
Kd=1
Kd=10
Kd=100
(c)
Normalized Y level at log-scale
Kdh=1
Kdh=10
Kdh=100
(d)
Normalized Y level at log-scale
h1=0.75, h2=1
h1=1, h2=1
h1=1.25, h2=1
(e)
Normalized Y level at log-scale
n1=1, n1h=1
n1=2, n1h=1
n1=2, n1h=2
X1 level
X2 level

(f)

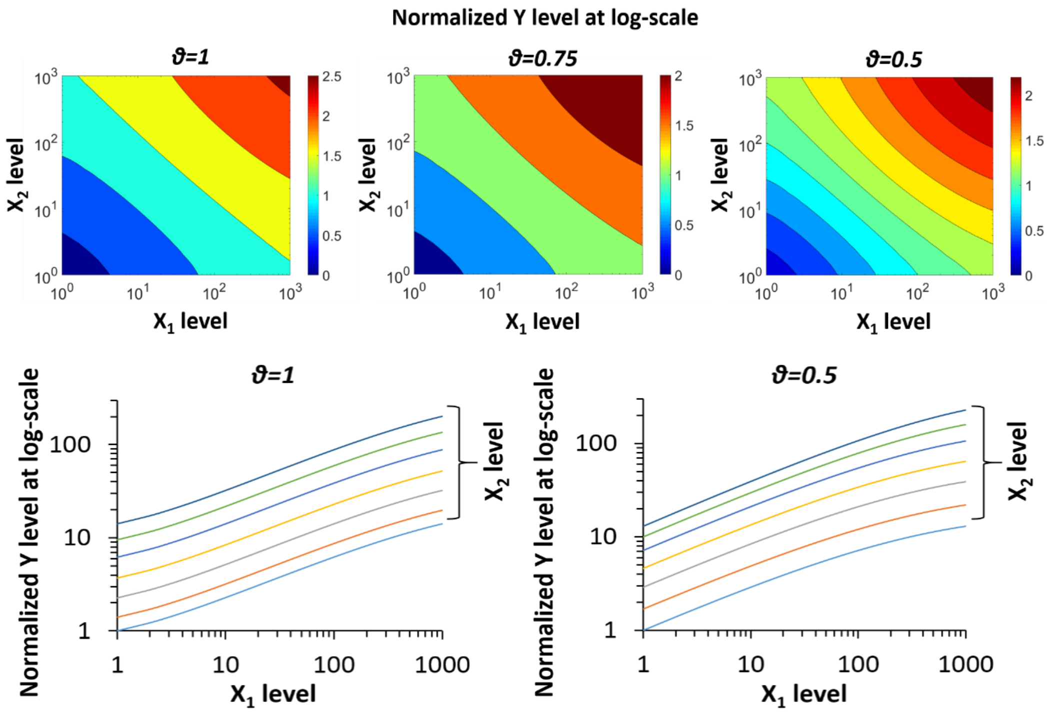

Supplementary Fig. 16. Simulation results of ANF loops and combinatorial promoter circuit motif.

- **a.** Effect of K_m: $K_m = K_{m1} = K_{m2}, h_1 = 1, h_2 = 1, k_{d1} = 1, K_{d2} = 1, K_{d1h} = 1, k_{d2h} = 1, R_{max1} = 1000, R_{max2} = 1, n_1 = 1, n_2 = 1, n_{1h} = 1, n_{2h} = 1, \theta = 1, \beta = 0.001$.
- **b.** Effect of K_d: $K_d = K_{d1} = K_{d2} = K_{d1h} = K_{d2h}, K_{m1} = 1, K_{m2} = 1,\ h_1 = 1, h_2 = 1,\ R_{max1} = 1000, R_{max2} = 1, n_1 = 1, n_2 = 1, n_{1h} = 1, n_{2h} = 1, \theta = 1, \beta = 0.001$.
- **c.** Effect of K_{dh}: $K_{dh} = K_{d1h} = K_{d2h}, k_{d1} = 1, K_{d2} = 1, K_{m1} = 1, K_{m2} = 1,\ h_1 = 1, h_2 = 1,\ R_{max1} = 1000, R_{max2} = 1, n_1 = 1, n_2 = 1, n_{1h} = 1, n_{2h} = 1, \theta = 1, \beta = 0.001$.
- **d.** Effect of h: $K_{m1} = 1, K_{m2} = 1, k_{d1} = 1, K_{d2} = 1, K_{d1h} = 1, k_{d2h} = 1, R_{max1} = 1000, R_{max2} = 1, n_1 = 1, n_2 = 1, n_{1h} = 1, n_{2h} = 1, \theta = 1, \beta = 0.001$.
- **e.** Effect of n: $K_{m1} = 1, K_{m2} = 1, k_{d1} = 1, K_{d2} = 1, K_{d1h} = 1, k_{d2h} = 1, R_{max1} = 1000, R_{max2} = 1, h_1 = 1, h_2 = 1, n_{1h} = 1, n_{2h} = 1, \theta = 1, \beta = 0.001$.
- **f.** Simulation results show the influence of interference between transcription factors (TFs) on the ANF loops and combinatorial promoter circuit motif.
  $K_m = K_{m1} = K_{m2}, h_1 = 1, h_2 = 1, k_{d1} = 1, K_{d2} = 1, K_{d1h} = 1, k_{d2h} = 1, R_{max1} = 1000,\ R_{max2} = 1, n_1 = 1, n_2 = 1, n_{1h} = 1, n_{2h} = 1, \beta = 0.001$.

2.2. Computed transfer function of power-law and multiplication function based on ANF

The simulations are based on Eq. 2.18, Eq. 2.19, and Eq. 2.20. Parameters that were used in simulations:

Based on P_{lacO} within ANF loop – Fig. 1e

$$K_{m1} = 0.8, K_{m2} = 1, K_{d1} = 10, K_{d2} = 5, K_{d1h} = 50, K_{d2h} = 5, h_1 = 1, h_2 = 1.4, R_{max1} = 2000, R_{max2} = 3000, \boldsymbol{n_1} = \mathbf{2}, n_2 = 2, n_{1h} = 1, n_{2h} = 1, \theta = 1, \qquad \beta = 0.001$$

Based on P_{lacO1} within ANF loop – Fig. 1f

$$K_{m1} = 0.8, K_{m2} = 1, K_{d1} = 90, K_{d2} = 9, K_{d1h} = 45, K_{d2h} = 4, h_1 = 1, h_2 = 1.4, R_{max1} = 2000, R_{max2} = 3000, n_1 = 0.95, n_2 = 1.8, n_{1h} = 1, n_{2h} = 1, \theta = 1, \beta = 0.001$$

The parameters that were used in our simulation fit well with the values that were reported in the literature. For example, the binding dissociation constant of LacI is known to be 10 times larger than of TetR. The interference parameter was set to $\theta = 1$. Furthermore, the maximum level of protein achieved by P_{lacO} in our simulation is smaller

than P_{tetO} ($R_{max1} < R_{max2}$), which is consistent with our construction that P_{lacO} is located on LCP and P_{tetO} and is located on MCN. The binding dissociation constant of LacI and TetR to their promoters within the ANF loop (P_{lacO} and P_{tetO}, respectively) is different than their values within the combinatorial promoter, because the promoters were located on different plasmid copy numbers.

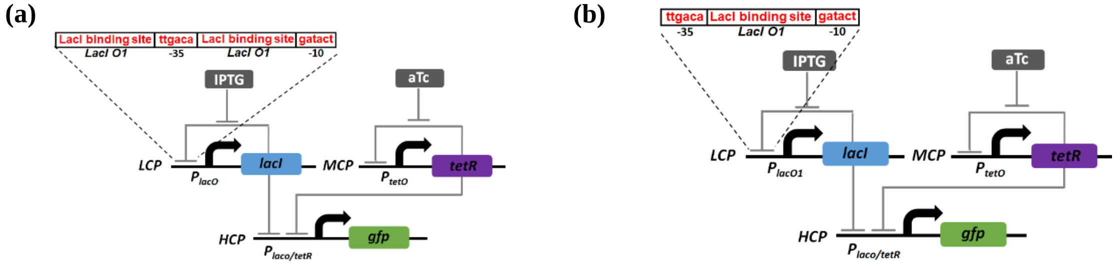

Supplementary Fig. 17. (a) The circuit that was used to produce the computed transfer function from Fig. 1e, power-law and multiplication based on P_{lacO}. **(b)** The circuit that was used to produce the computed transfer function from Fig. 1f, power-law and multiplication based on P_{lacO1}.

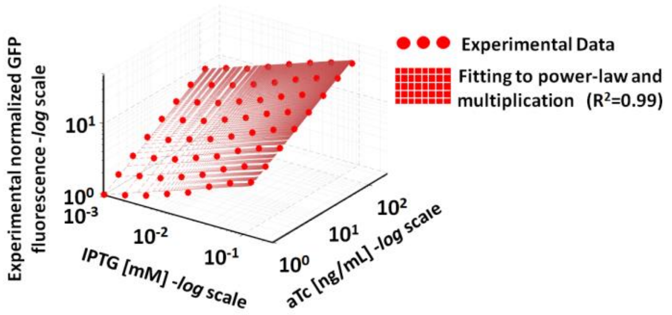

Supplementary Fig. 18. Fitting the experimental results of P_{lacO1} and P_{tetO} ANF loops and combinatorial promoter ($P_{lacO/tetO}$- GFP) to power-law and multiplication function $\left(\frac{IPTG}{1.25}\right)^{0.3375} \cdot \left(\frac{aTc}{0.7}\right)^{0.4375}$.The data appears in Fig. 1d in the main text and is reproduced here for clarity.

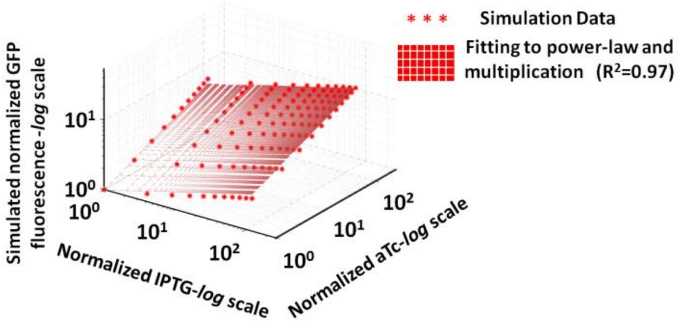

Supplementary Fig. 19. Simulation results of P_{lacO1} and P_{tetO} ANF loops and combinatorial promoter ($P_{\frac{lacO}{tetO}}$- GFP) and fitting to power-law and multiplication function $\left(\frac{IPTG}{1.25}\right)^{0.3375} \cdot \left(\frac{aTc}{0.7}\right)^{0.26}$. The data appears in Fig. 1e in the main text and is reproduced here for clarity.

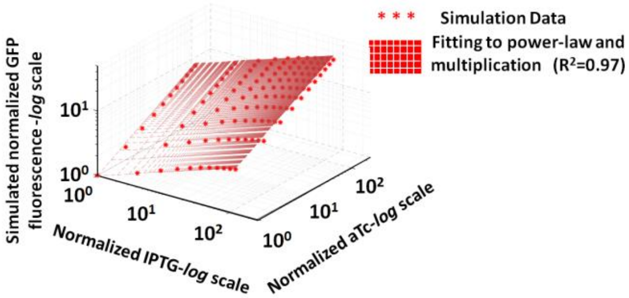

Supplementary Fig. 20. Simulation results of P_{lacO1} and P_{tetO} ANF loops and combinatorial promoter ($P_{lacO/tetO}$- GFP) and fitting to power-law and multiplication function $\left(\frac{IPTG}{1.25}\right)^{0.3375} \cdot \left(\frac{aTc}{0.7}\right)^{0.437}$. The data appears in Fig. 1f in the main text and is reproduced here for clarity.

2.2.1. A simple mode for Auto-negative feedback

$$LacI_T = \frac{\alpha_{11} \cdot \tau_1}{1+\left(\frac{LacI}{K_d}\right)^{n_{11}}} \quad (2.\ 20.1)$$

α_{11} is the production rate of LacI by the P_{lacO_ANF}

n_{11} is the Hill-coefficient of binding LacI to P_{lacO_ANF} promoter

K_d is the dissociation constant of binding LacI to a single copy of P_{lacO_Rep} (promoter within ANF), which equals (our assumption) to the dissociation constant of binding LacI to a single copy of P_{lacO_REP} promoter regulating GFP.

τ_1 is LacI half-life

$$LacI = LacI_T \cdot f(IPTG) \quad (2.\ 20.2)$$

$$f(IPTG) = \frac{1}{K_m{}^{h_1} + IPTG^{h_1}}$$

h_1 is the Hill-coefficient of binding IPTG (x_1) to LacI (R_1)

$$GFP = \frac{\alpha_{21} \cdot \tau_2}{1+\left(\frac{LacI}{K_d}\right)^{n_{21}}} \quad (2.\ 20.3)$$

α_{21} is the production rate of GFP by the P_{lacO_Rep}

n_{21} is the Hill-coefficient of binding LacI to P_{lacO_Rep} promoter

τ_2 is GFP half-life

$$LacI_T = \frac{\alpha_{11} \cdot \tau_1}{1+\left(\frac{LacI_T \cdot f(IPTG)}{K_d}\right)^{n_{11}}}$$

$$LacI_T + LacI_T \cdot \left(\frac{LacI_T \cdot f(IPTG)}{K_d}\right)^{n_{11}} = \alpha_{11} \cdot \tau_1 \quad (2.\ 20.4)$$

Multiplying by K_d ther term

$$K_d \cdot \frac{LacI_T}{K_d} + K_d \cdot \frac{LacI_T}{K_d} \cdot \left(\frac{LacI_T \cdot f(IPTG)}{K_d}\right)^{n_{11}} = K_d \cdot \alpha_{11} \cdot \tau_1 \quad (2.\ 20.5)$$

Multiplying by $f(IPTG)$:

$$K_d \cdot \frac{LacI_T \cdot f(IPTG)}{K_d} + K_d \cdot \frac{LacI_T \cdot f(IPTG)}{K_d} \cdot \left(\frac{LacI_T \cdot f(IPTG)}{K_d}\right)^{n_{11}} = K_d \cdot \alpha_{11} \cdot \tau_1 \cdot f(IPTG) \quad (2.\ 20.6)$$

Substituting $LacI = f(IPTG) \cdot LacI_T$ into Eq. 2.20.6

$$K_d \cdot \frac{LacI}{K_d} + K_d \cdot \frac{LacI}{K_d} \cdot \left(\frac{LacI}{K_d}\right)^{n_{11}} = K_d \cdot \alpha_{11} \cdot \tau_1 \cdot f(IPTG)$$

$$K_d \cdot \frac{LacI}{K_d} + K_d \cdot \left(\frac{LacI}{K_d}\right)^{n_{11}+1} = K_d \cdot \alpha_{11} \cdot \tau_1 \cdot f(IPTG) \qquad (2.\ 20.7)$$

$For\ K_d \ll \alpha_{11} \cdot \tau_{11}:$

$$\frac{LacI}{K_d} = \left(f(IPTG) \cdot \frac{\alpha_{11} \cdot \tau_1}{K_d}\right)^{1/(n_{11}+1)} \qquad (2.\ 20.8)$$

Substituting Eq. 2.20.8 into Eq. 2.20.3:

$$GFP = \frac{\alpha_{21} \cdot \tau_2}{1 + \left(f(IPTG) \cdot \frac{\alpha_{11} \cdot \tau_1}{K_d}\right)^{n_{21}/(n_{11}+1)}} \qquad (2.\ 20.9)$$

Using $\alpha_{21} \cdot \tau_2 = GFP_{max}$, $\alpha_{11} \cdot \tau_1 = LacI_{max}$

$$\Rightarrow GFP = \frac{GFP_{max}}{1 + \left(\frac{LacI_{max} \cdot f(IPTG)}{K_d}\right)^{n_{21}/(n_{11}+1)}} \qquad (2.\ 20.10)$$

f(IPTG) is the induction function, $GFP_{max} = \alpha_{21} \cdot \tau_2$, $LacI_{max} = \alpha_{11} \cdot \tau_1$. In case that $n_{21} = 1$ (e.g., $P_{lacO/tetO}$- GFP), the Hill- coefficient of $IPTG - GFP$ is proportional to $1/(n_{11} + 1)$. Increasing the n_{11} leads to decrease the Hill coefficient of $IPTG - GFP$ transfer function.

Now we will solve equation (2. 20.7) when n_{11}=1:

$$K_d \cdot \frac{LacI}{K_d} + K_d \cdot \left(\frac{LacI}{K_d}\right)^2 = K_d \cdot \alpha_{11} \cdot \tau_1 \cdot f(IPTG) \qquad (2.\ 20.11)$$

$$\frac{LacI}{K_d} = \frac{-1 + \sqrt{1 + 4 \cdot \frac{\alpha_{11} \cdot \tau_1}{K_d} \cdot f(IPTG)}}{2} \qquad (2.\ 20.12)$$

Then, we get that the GFP:

$$GFP = \frac{GFP_{max}}{1 + \left(\frac{-1 + \sqrt{1 + 4 \cdot \frac{LacI_{max}}{K_d}}}{2}\right)^{n_{21}}} \qquad (2.\ 20.13)$$

In case that the copy number of P_{lacO_ANF} and P_{lacO_Rep} is different.

N_1 is the plasmid copy number of P_{lacO_ANF}

N_2 is the plasmid copy number of P_{lacO_Rep}.

Then, the K_d is increased by the total number of P_{lac}o : $K_d \cdot (N_1 + N_2)$

The maximum expressed LacI is also increased by : $N_1 \cdot LacI_{max}$

The maximum expressed GFP is also increased by : $N_2 \cdot GFP_{max}$. Eq. 2.20.10 can be writtein as:

$$GFP = \frac{N_2 \cdot GFP_{max}}{1 + \left(\frac{-1 + \sqrt{1 + 4 \cdot \frac{LacI_{max}}{K_d} \cdot \frac{N_1}{(N_1 + N_2)} \cdot f(IPTG)}}{2} \right)^{n_{21}}} \qquad (2.\ 20.14)$$

Our simulation results demonstrate that we can control the weight by changing the ratio between the plasmid copy number of the auto-negative feedback circuit (P_{lacO_ANF}) and the reporting (P_{lacO_Rep}).

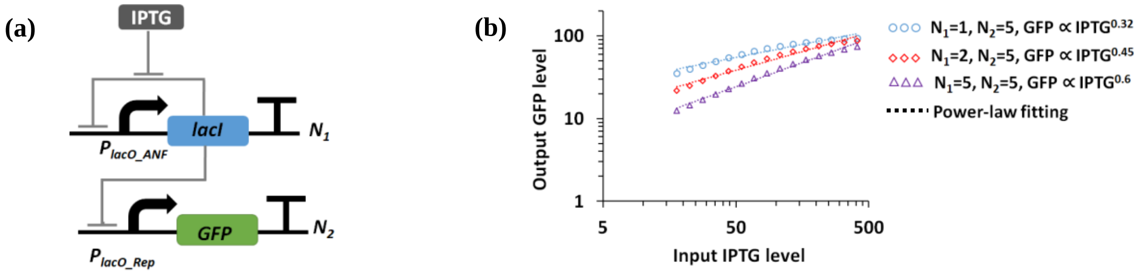

Supplementary Fig. 21. (a) Auto-negative feedback loop circuit. **(b)** Simulation result of Eq. 2. 20.14.

2.3. Characterization of P_{BAD} promoter

It has been reported that the behavior of the Arabinose-inducible promoter P_{BAD} is strongly affected by the arabinose concentration [12]. Using the characteristic of P_{BAD}, we constructed a graded auto-positive feedback (APF) circuit (Supplementary Fig. 22a) to tune the expression level of AraC. The purpose of the graded APF is to increase the dynamic range of P_{lux} which can regulate the AraC level to a very wide range. The analysis for the APF circuit and linearization is provided in the next sections. We added a ssrA degradation tag [13] (LAA) to AraC to ensure low basal in the absence of the input (AHL). The circuit was first induced with different concentrations of Arabinose (0.7mM, 0.2mM, 0.07mM and 0.02mM). Then the experimental results (Supplementary Fig. 22b) were fitted to Hill-function ($a \cdot \frac{AHL^{m_{eff}}}{AHL^{m_{eff}} + K_{eff}^{m_{eff}}} + b$). Our experimental results show that in the presence of a low level of Arabinose (0.02mM), the input dynamic range of AHL to P_{BAD} decreased with $m_{eff} = 3, K_{eff} = 120nM, a = 165\ (a.u.), b = 1\ (a.u.)$. Whereas with a high level of Arabinose (0.7mM), the input dynamic range increased with $m_{eff} = 1.5, K_{eff} = 200nM, a = 170\ (a.u.), b = 1\ (a.u.)$. As a result, the Hill-coefficient of binding Arabinose- AraC complex to P_{BAD} increases when Arabinose is decreased. The relation between the effective dissociation constant K_{eff} and m_{eff} is summarized in Supplementary Fig. 22c and can be well fitted using the power-law function ($K_{eff} = a \times m_{eff}^{-b}$). The transfer function of P_{BAD} promoter with respect to AraC is shown in Supplementary Fig. 22d. The level of AraC is evaluated by the GFP signal from Supplementary Fig. 26c.

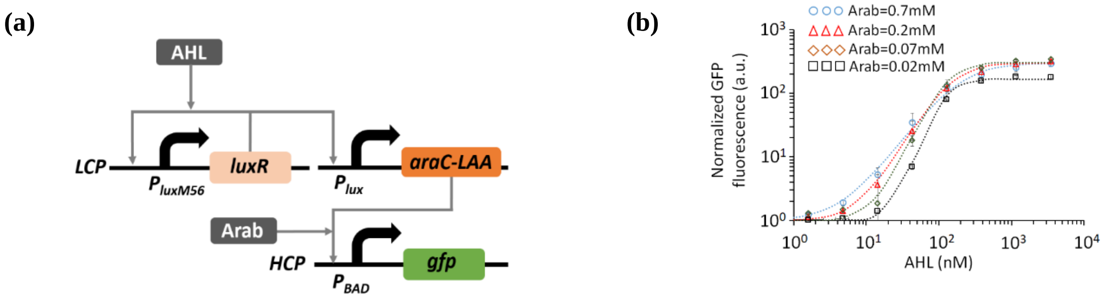

(c)

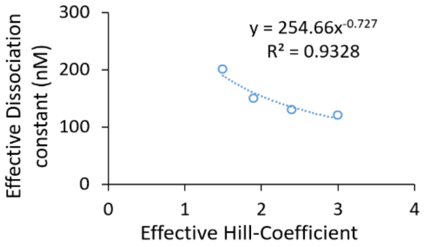

(d)

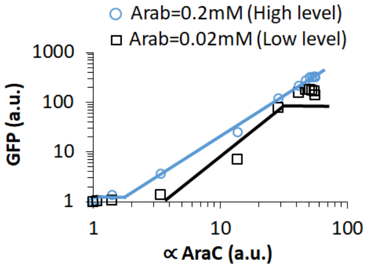

Supplementary Fig. 22. (a) The Characterization of P_{BAD} promoter by tuning the expression level of AarC by mutated APF. **(b)** Experimental results of AHL – GFP transfer function for a low Arabinose (0.02mM) and a high Arabinose (0.2mM). The dotted lines are a Hill-function fitting. Data are presented as average ±standard deviations from independent replicates ($n = 3$). **(c)** The relation between the effective dissociation constant and effective Hill-coefficient is described by a power-law function. **(d)** AraC - P_{BAD} the transfer function for low and high Arabinose levels. The level of AraC is evaluated by the GFP signal from Supplementary Fig. 26c. a.u. represent arbitrary units.

Nonlinear fitting models of AraC -based synthetic perceptgene circuit

We fitted the experimental results of P_{laco} and P_{teto} ANF loops and combinatorial promoter ($P_{lacO/tetO}$- GFP) (data appears in Fig. 1d) to perceptgene model: $\frac{[(\frac{IPTG}{1.25})^{0.3375} \cdot (\frac{aTc}{0.75})^{0.4375}/150]+0.0001}{[(\frac{IPTG}{1.25})^{0.3375} \cdot (\frac{aTc}{0.75})^{0.4375}/150]+1}$ (Supplementary Fig. 23a) and, the experimental results of IPTG, aTc perceptgene circuit (data appears in Fig. 1h) to perceptgene model: $\frac{[(\frac{IPTG}{1.25})^{0.3375} \cdot (\frac{aTc}{0.75})^{0.4375}/19]^{2.2}+0.045}{[(\frac{IPTG}{1.25})^{0.3375} \cdot (\frac{aTc}{0.75})^{0.4375}/19]^{2.2}+1}$ (Supplementary Fig. 23b). A general formula is **:** $\frac{[(\frac{AHL}{K_1})^{n_1} \cdot (\frac{aTc}{K_2})^{n_2}/K_d]^m+\beta}{[(\frac{AHL}{K_1})^{n_1} \cdot (\frac{aTc}{K_2})^{n_2}/K_d]^m+1}$

(a)

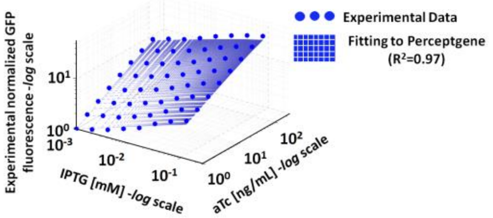

(b)

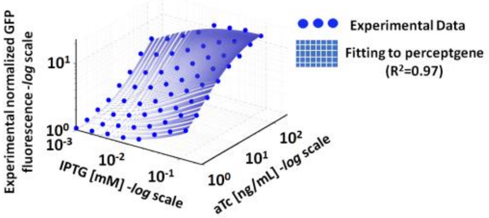

Supplementary Fig. 23. (a) Fitting the experimental results (appear in Fig. 1d) of P_{laco} and P_{teto} ANF loops and combinatorial promoter ($P_{lacO/tetO}$- GFP) to perceptgene model. **(b)** Fitting experimental results of perceptgene based ANF loops and $P_{lacO1/tetO}$ combinatorial promoter (appear in Fig. 1h) to perceptgene model

2.4. Model of AraC -based synthetic perceptgene circuit

The P_{BAD} promoter is activated by the AraC -TF when it is induced by arabinose (Arab). The probability of the P_{BAD} promoter being induced by the arabinose-AraC complex is described by [2]:

$$P = \frac{\frac{AraC_C}{K_{d3}}+\beta_4}{1+\beta_4+\frac{AraC_C}{K_{d3}}+\frac{AraC}{K_{d4}}} \qquad (2.\ 21.1)$$

$$Z = Z_{max} \cdot P \qquad (2.\ 21.2)$$

where $AraC_C$ is the concentration of the Arabinose-AraC complex, AraC is the concentration of free AraC –TF, K_{d3} is the dissociation constant for binding of the Arabinose- AraC complex to P_{BAD} promoter, K_{d4} is the dissociation constant for free AraC binding to P_{BAD}, and β_4 is the basal level of P_{BAD} promoter. Z is the gene expression level, and Z_{max} is the maximum expressed level.

The set of ordinary differential equations which model the process of free inducer (Arabionse) binding to free AraC can be described by:

$$\frac{dAraC_c}{dt} = k_3 \cdot AraC \cdot Arab - k_{-3} \cdot AraC_c \quad (2.22)$$

Where $AraC_c$ is the concentration of the arabinose- AraC complex. k_3 and k_{-3} is the forward and reverse reaction rates. In the case of P_{BAD} promoter, where AraC and $AraC_c$ can bind on the promoter, we can claim:

$$AraC + AraC_c + Z = AraC_T \quad (2.22.1)$$

Where $AraC_T$ is the total concentration of AraC in the steady state, and including Hill coefficient to Arabionse, we get :

$$AraC_c = (AraC_T - z) \cdot \frac{\left(\frac{Arab}{K_{m3}}\right)^{h_3}}{1+\left(\frac{Arab}{K_{m3}}\right)^{h_3}} \quad (2.\ 22.2)$$

$$AraC = (AraC_T - z) \cdot \frac{1}{1+\left(\frac{Arab}{K_{m3}}\right)^{h_3}}$$

With K_{m3} is the dissociation constant of binding arabinose to AraC and h_3 is the Hill coefficient (~2.8 [14]). Substituting, Eq. 22.2 into2.21, we obtain:

$$-Z^2 \cdot \left(f + \frac{K_{d3}}{K_{d4}} \cdot g\right) + Z \cdot \left[K_{d3} + AraC_T \cdot \left(f + \frac{K_{d3}}{K_{d4}} \cdot g\right) + Z_{max} \cdot f\right] = Z_{max} \cdot [\beta_4 \cdot K_{d3} + AraC_T \cdot f]$$

Where: $f \equiv \frac{\left(\frac{Arab}{K_{m3}}\right)^{h_3}}{1+\left(\frac{Arab}{K_{m3}}\right)^{h_3}}$, $g \equiv \frac{1}{1+\left(\frac{Arab}{K_{m3}}\right)^{h_3}}$ (2. 23)

In the perceptgene circuit the $AraC_T$ is equal to the output of the power-law and multiplication function (Y) at steady state:

$$AraC_T = Y \quad (2.\ 24)$$

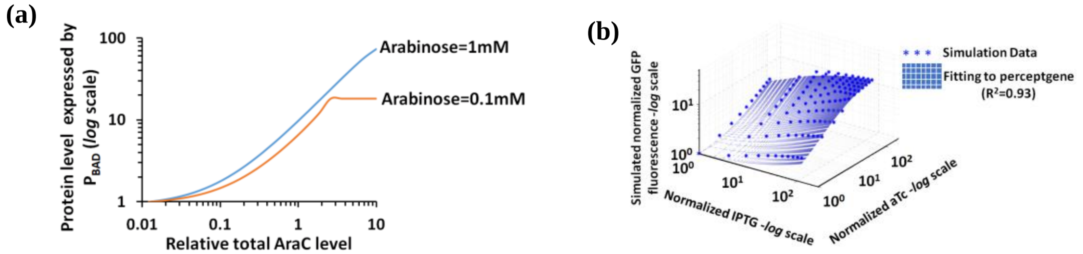

Supplementary Fig. 24. (a) The computed transfer function of P_{BAD} promoter appears in Fig. 1i. Parameters that were used in simulation: Arab=0.085 $AraC_T = 40$, $K_{m3} = 0.09$, $K_{d3} = 5$, $K_{d4} = 50$, $h_3 = 2.8$ [14], $\beta_4 = 0.045$. GFPmax=30. The ratio between K_{d3} and K_{d4} fits well to the values that were reported in the literature [2]. **(b)** perceptgene based on AraC system. The simulation data is fitted to perceptgene model $\frac{[\left(\frac{IPTG}{1.25}\right)^{0.3375} \cdot \left(\frac{aTc}{0.75}\right)^{0.4375}/19]^{2.2}+0.045}{[\left(\frac{IPTG}{1.25}\right)^{0.3375} \cdot \left(\frac{aTc}{0.75}\right)^{0.4375}/150]^{2.2}+1}$.

2.5. Model of APF, ANF loops and combinatorial promoter

In this section, we present a model that describes the behavior of APF, ANF loops with a combinatorial promoter (Supplementary Fig. 25a), which results in power-law and multiplication function:

- The Hill coefficient of binding A activator to P_1 (promoter within APF) is equal to Hill coefficient of binding A to $P_{1/2}$(combinatorial promoter) and are equal to, n_1
- The Hill coefficient of binding R repressor to P_2 (promoter within ANF) is equal to Hill coefficient of binding R to $P_{1/2}$ (combinatorial promoter), n_2
- The binding affinity of A activator to P_1 (within APF) is equal to binding affinity to $P_{1/2}$ (combinatorial promoter), K_{d1}

- The binding affinity of R repressor to P_2 (within ANF) is equal to binding affinity to $P_{1/2}$ (combinatorial promoter), K_{d2}
- The β of $P_{1/2}$ is very low.

The binding states for $P_{1/2}$ combinatorial promoter is shown in Supplementary Fig. 25b. The probability for $P_{1/2}$ promoter being in the open complex is described by the following equations:

$$P_{1/2} = \frac{\left(\frac{A}{K_{d1}}\right)^{n_1}}{1+\left(\frac{A}{K_{d1}}\right)^{n_1}+\left(\frac{R}{K_{d2}}\right)^{n_2}+\theta\cdot\left(\frac{A}{K_{d1}}\right)^{n_1}\cdot\left(\frac{R}{K_{d2}}\right)^{n_2}} \quad (2.\ 25)$$

For simplicity, we assumed that $n_1 = 1$. Then, the expression level of the output protein at a steady state is given by:

$$Z = Z_{max} \cdot \frac{\frac{A}{K_{d1}}}{1+\frac{A}{K_{d1}}+\left(\frac{R}{K_{d2}}\right)^{n_2}+\theta\cdot\frac{A}{K_{d1}}\cdot\left(\frac{R}{K_{d2}}\right)^{n_2}} \quad (2.\ 26)$$

Where Z_{max} is the maximum protein level achieved by $P_{1/2}$ promoter.

A graded Positive feedback model: The first step toward implementation of synthetic power-law and multiplication function in living cells, is to broaden the input dynamic range of genetic synthetic parts. It has shown that a graded PF loop increased the input dynamic range by more than three orders of magnitude [2] (Supplementary Fig. 25c). TFs bindings to promoters are modeled according to the Shea-Ackers formalism [9,10]. Therefore, the total level of expressed A activator (Supplementary Fig. 25c) in the case of APF loops can be expressed as:

$$A_T = A_{max} \cdot \frac{\frac{A}{K_{d1}}+\beta_1}{1+\beta_1+\frac{A}{K_{d1}}} \quad (2.\ 27)$$

$$Y = Y_{max} \cdot \frac{\frac{A}{K_{d2}}+\beta_2}{1+\beta_2+\frac{A}{K_{d2}}} \quad (2.\ 28)$$

Where A_{max} and Y_{max} are the maximum protein levels achieved by P_1 and P_2 respectively, β_1 and β_2 are the Basal levels of P_1 and P_1 respectively, and A is the level of activators that are bound to P_1 and P_2. The induction of the activator by x inducers is given by:

$$A = A_T \cdot g(x) \quad (2.\ 29)$$

$$g(x) = \frac{\left(\frac{x}{K_m}\right)^h}{1+\left(\frac{x}{K_m}\right)^h} \quad (2.\ 30)$$

Where K_m is the dissociation constant and h the Hill coefficient of binding inducer to activator. Substituting Eq. 2.29 into Eq. 2.27 we get:

$$A = A_{max} \cdot \frac{\frac{A_T\cdot g(x)}{K_{d1}}+\beta_1}{1+\beta_1+\frac{A_T\cdot g(x)}{K_{d1}}} \quad (2.\ 31)$$

$$A_T + A_T\cdot\beta_1 + \frac{{A_T}^2\cdot g(x)}{K_{d1}} = A_{max}\cdot\frac{A_T\cdot g(x)}{K_{d1}} + A_{max}\cdot\beta_1$$

$$A_T\cdot\frac{K_{d1}}{g(x)} + A_T\cdot\beta_1\cdot\frac{K_{d1}}{g(x)} + {A_T}^2 = A_{max}\cdot A_T + A_{max}\cdot\beta_1\cdot\frac{K_{d1}}{g(x)}$$

$$A_T\cdot\left(\frac{K_{d1}}{g(x)} + \beta_1\cdot\frac{K_{d1}}{g(x)} - A_{max}\right) + {A_T}^2 = A_{max}\cdot\beta_1\cdot\frac{K_{d1}}{g(x)}$$

$$A_T\cdot\frac{K_{d1}}{g(x)}\cdot\left(1+\beta_1-\frac{A_{max}\cdot g(x)}{K_{d1}}\right) + {A_T}^2 = A_{max}\cdot\beta_1\cdot\frac{K_{d1}}{g(x)} \quad (2.\ 32)$$

In case that $A_{max}/K_{d1}<1$, we can approximate Eq. 2.32 as:

$$A_T \approx \frac{A_{max}\cdot\beta_1}{\left(1+\beta_1-\frac{A_{max}}{K_{d1}}\cdot g(x)\right)} \quad (2.\ 33)$$

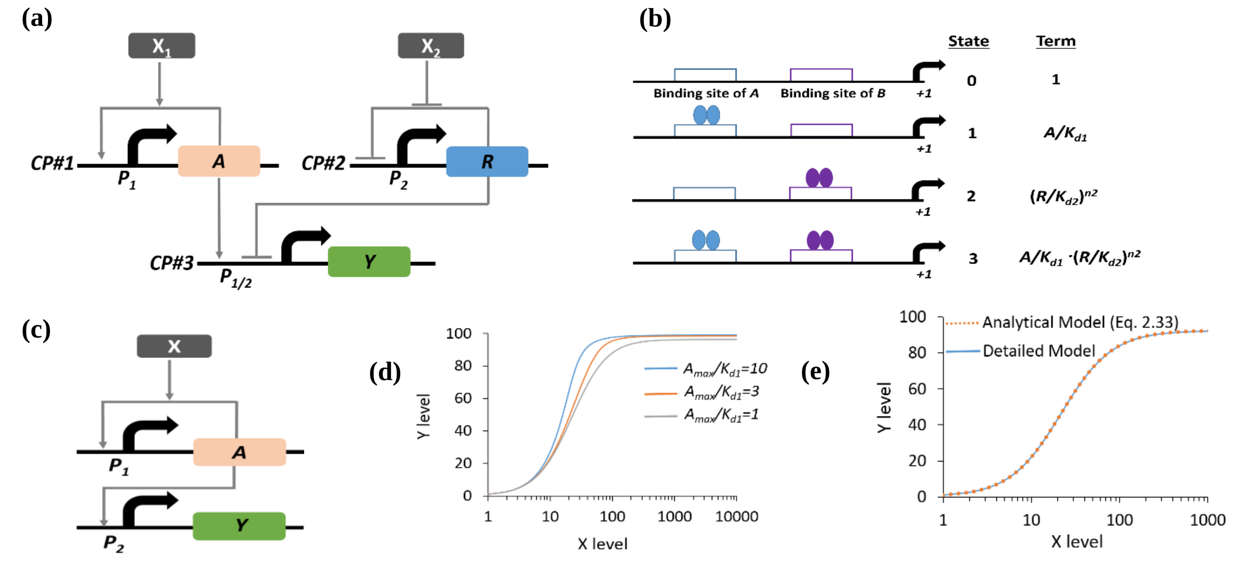

Supplementary Fig. 25. (a) APF and ANF loops combined with a hybrid combinatorial promoter, CP#=Copy number of plasmids. **(b)** The binding states of $P_{1/2}$ promoter. **(c)** APF loop circuit. **(d)** Simulation results of APF loop circuit using a detailed model $K_m = 100$, $m = 1.5, A_{max} = 1000, \beta_1 = 0.1, K_{d2} = 10, Z_{max} = 1, \beta_2 = 0.01$. **(e)** The detailed and analytical models of APF ($K_d = 3000$).

The Simulation results for the exact model of APF (Eq. 2.27-Eq. 2.30) and the approximated model based on Eq. 2.33 are shown in Supplementary Figs. 25d and 25e. When the ratio A_{max}/K_d decreases, the input dynamic range increases.

By applying a logarithmic operation to Eq. 2.33, we get:

$$\log(A_T) \approx log(A_{max} \cdot \beta_1) - log\left(1 + \beta_1 - \frac{A_{max}}{K_{d1}} \cdot g(x)\right) \qquad (2.\ 34)$$

By substituting Eq. 2.30 into Eq. 2.34, and assuming that β_1"1, we get:

$$\log(A_T) \approx log(A_{max} \cdot \beta_1) + log\left(1 + \left(\frac{x}{K_m}\right)^h\right) - log\left(1 + \left(\frac{x}{K_m}\right)^h \cdot \left(1 - \frac{A_{max}}{K_{d1}}\right)\right) \qquad (2.\ 35)$$

In case that $x/K_m >> 1$ and $\left(\frac{x}{K_m}\right)^h \cdot \left(1 - \frac{A_{max}}{K_{d1}}\right) < 1$, we can approximate Eq. 2.35 as:

$$\log(A_T) \approx log(A_{max} \cdot \beta_1) + h \cdot log\left(\frac{x}{K_m}\right) - \left(1 - \frac{A_{max}}{K_{d1}}\right) \cdot \left(\frac{x}{K_m}\right)^h \qquad (2.\ 36)$$

$$\log(A_T) \approx log(A_{max} \cdot \beta_1) + h \cdot log\left(\frac{x}{K_m}\right) - \left(1 - \frac{A_{max}}{K_{d1}}\right) \cdot e^{h \cdot ln\left(\frac{x}{K_m}\right)}$$

$$\log(A_T) \approx log(A_{max} \cdot \beta_1) + h \cdot log\left(\frac{x}{K_m}\right) - \left(1 - \frac{A_{max}}{K_{d1}}\right) \cdot \left(1 + h \cdot ln\left(\frac{x}{K_m}\right)\right)$$

$$\log(A_T) \approx log(A_{max} \cdot \beta_1) + h \cdot log\left(\frac{x}{K_m}\right) - 2.3 \cdot \left(1 - \frac{A_{max}}{K_{d1}}\right) \cdot \left(\frac{1}{2.3} + h \cdot log\left(\frac{x}{K_m}\right)\right)$$

$$\log(A_T) \approx log(A_{max} \cdot \beta_1) - \left(1 - \frac{A_{max}}{K_{d1}}\right) + \left[1 - 2.3 \cdot \left(1 - \frac{A_{max}}{K_{d1}}\right)\right] \cdot h \cdot log\left(\frac{x}{K_m}\right) \qquad (2.\ 37)$$

Eq. 2.37 shows that a graded APF loop, when $A_{max}/K_d << 1$, can be approximated as a power-law function. The power-law coefficient is mainly set by Hill coefficient h and the ratio of A_{max}/K_d. The simulation results of Supplementary Fig. 25d show that the power-law coefficient in the case of strong APF ($A_{max}/K_d = 10$) is 1.5, and for a graded APF ($A_{max}/K_d = 1$) is 1.35.

Experimental Results of APF: To test our approach, we first created various synthetic libraries that permute the sequence features affecting DNA binding site affinity. This was achieved by creating random mutations in the TF-DNA binding site sequence within the promoter. The synthetic P_{lux} promoter was selected due to its simple structure [14]. The promoter consists of a single LuxR binding site upstream to the -35 location. First, we constructed an open-loop gene circuit consisting of two components: a constitutive promoter regulating the expression of the LuxR gene, and a P_{lux} promoter regulating the expression of GFP (Supplementary Fig. 26a). Then we ran a random mutation on the first 7 nucleotides of the LuxR binding site sequence [15], resulting in a new promoter called (P_{luxM56}). To test the new promoter, we reconstructed an open-loop and APF circuits with the P_{luxM56} promoter (Supplementary Fig. 26b). The positive feedback circuit consisting of a positive feedback loop based on the a mutated P_{lux} promoter regulates the expression of the LuxR gene and a wild type P_{lux} promoter, which regulates the expression of GFP. As shown in Supplementary Fig. 26c, the mutated promoters (P_{luxM56} in open circuit) exhibited weaker TF-DNA binding than the wild type promoter (P_{lux} in open circuit), with a lower GFP signal and a wider input dynamic range. In particular, the mutated promoter (P_{luxM56}) gives rise a graded APF transfer function with a broad region of linearity for more than four orders of magnitude without losing its magnitude. The measured transfer functions of multiple circuits were fitted using Hill function $a \cdot \frac{\left(\frac{AHL}{k}\right)^{meff}}{\left(1+\left(\frac{AHL}{k}\right)^{meff}\right)} + b$ (Supplementary Fig. 26c). The strength of a PF loop, which is set by the dissociation constant of binding LuxR to P_{lux} or P_{luxM56}, affects the input dynamic range and the effective Hill coefficient meff, as well as the power-law coefficient. Our experimental results showed that the influence of dissociation constant on m_{eff} is much larger than our theoretical analysis. This is because our theoretical analysis is based on Michaelis-Menten model, which assumes that the TF concentration is much larger than promoter concentration. It has been shown that, when these assumptions are violated, detailed biochemical reaction models can capture the behavior of graded APF accurately [2]. Supplementary Fig. 27 shows the experimental results of AHL – GFP transfer function for other mutated P_{lux} promoter.

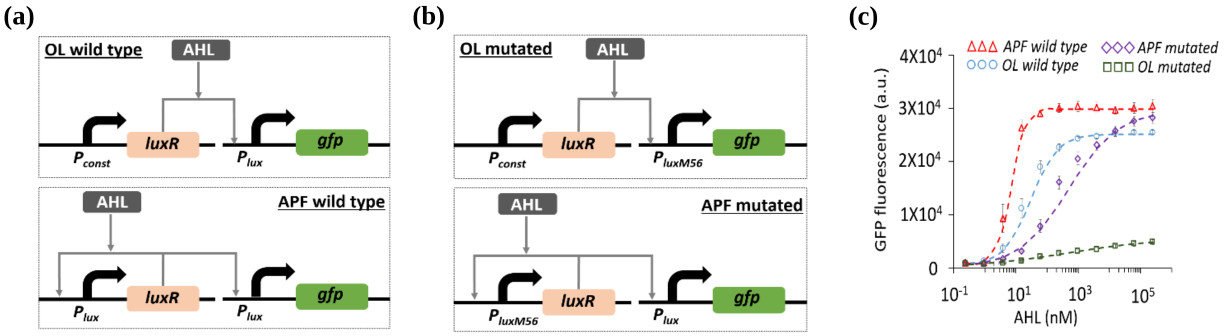

Supplementary Fig. 26. (a) The construction of open-loop (OL) and APF circuits based on P_{lux} promoter. **(b)** The construction of OL and APF circuits based on mutated P_{lux} promoter (P_{luxM56}). **(c)** Measured transfer functions of multiple circuits. Data are presented as average ±standard deviations from independent replicates ($n = 3$). dots are experimental data, and dashed-line is a Hill function fitting with the below parameters:

OL circuit – Wild type P_{lux}: $K = 30, m_{eff} = 1, a = 25 \times 10^3, b = 600$

APF circuit – Wild type P_{lux}: $K = 7, m_{eff} = 2, a = 30 \times 10^3, b = 800$

OL circuit – Mutated P_{luxM56}: $K = 500, m_{eff} = 0.3, a = 5 \times 10^3, b = 100$

APF circuit – Mutated P_{luxM56}: $K = 500, m_{eff} = 0.5, a = 30 \times 10^3, b = 100$

Supplementary Fig. 27 shows the experimental results of AHL – GFP transfer function for other mutated P_{lux} promoter.

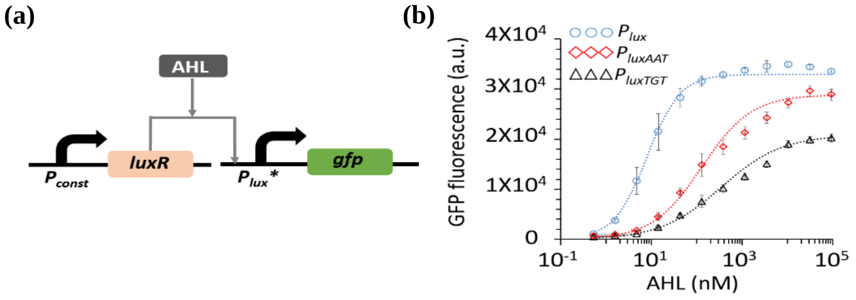

Supplementary Fig. 27. (a) Open Loop circuit using P_{lux}/LuxR mutation. Constitutive promoter controls LuxR expression encoded on LCP, Plux mutated promoters control GFP expression encoded on LCP. **(b)** AHL − GFP transfer function of mutated P_{lux} promoters (P_{luxAAT}, P_{luxTGT}). Data are presented as average ±standard deviations from independent replicates ($n = 3$). Dots are experimental data, and the dashed line is a Hill function fitting.

Power-law and multiplication function based on ANF and graded APF loops: The combination of graded APF and ANF loops with a combinatorial promoter is shown in Supplementary Fig. 25a set of equations that describes the behavior of this system at a steady state is given by:

- APF loop equation:

$$A_{T1} = A_{max1} \cdot \frac{\frac{A}{K_{d1}}+\beta_1}{1+\beta_1+\frac{A}{K_{d1}}} \qquad (2.38)$$

- Induction of X_1 (AHL) and Y_1 (LuxR) equation [2]:

$$A \approx A_T \frac{{X_1}^2}{{X_1}^2+2\cdot X_1\cdot A_T+{K_{m1}}^2} \qquad (2.39)$$

- ANF loops equation:

$$R_T = R_{max} \frac{1}{1+\beta_2+\left(\frac{R}{K_{d2}}\right)^{n_2}} \qquad (2.40)$$

- Induction of X_2 and R:

$$R = R_T \cdot \frac{1}{1+\left(\frac{x_2}{K_{m2}}\right)^{h_2}} \qquad (2.41)$$

- The a ctivity of combinatorial promoter:

$$P_{12} = \frac{\frac{A}{K_{dh1}}+\beta_{12}}{1+\beta_{12}+\frac{A}{K_{dh1}}+\left(\frac{R}{K_{dh2}}\right)^{n_2}+\theta\cdot\frac{A}{K_{dh1}}\cdot\left(\frac{R}{K_{dh2}}\right)^{n_2}} \qquad (2.42)$$

Where β_{12} is the basal level of combinatorial promoter.

- The mass balance between the complex and the promoter; the sum of free TF concentration and complex concentration is equal to the total concentration of TF ($A + Complex = A_T$):

$$A + P_{T1}\cdot\frac{\frac{A}{K_{d1}}+\beta_1}{1+\beta_1+\frac{A}{K_{d1}}} + P_{T12}\cdot\frac{\frac{A}{K_{dh1}}+\theta\cdot\frac{A}{K_{dh1}}\cdot\left(\frac{R}{K_{dh2}}\right)^{n_2}}{1+\frac{A}{K_{dh1}}+\left(\frac{R}{K_{dh2}}\right)^{n_2}+\theta\cdot\frac{A}{K_{dh1}}\cdot\left(\frac{R}{K_{dh2}}\right)^{n_2}} = A_T \qquad (2.43)$$

P_{T1} and P_{T12} are the total concentration of P_1 and P_{12} promoters. For simplicity, we assumed that $\theta \approx 1$, and $P_{T12} >> P_{T1}$. Therefore, we can write:

$$A + P_{T12}\cdot\frac{\frac{A}{K_{d1h}}}{1+\frac{A}{K_{d1h}}} = A_T \qquad (2.44)$$

$$A^2 + A\cdot(K_{d1h} + P_{T12} - A_T) = Y_A \cdot K_{d1h} \qquad (2.45)$$

A general formula for Eq. 2.45 is given by: $A^2 + A\cdot(K_{d1h} + P_{T12} - \xi\cdot A_T) = Y_A \cdot K_{d1h}$

Simulation results of $P_{lux/tetO}$-based power-law and multiplication function (Fig. 2c): Parameters were used: $K_{m1} = 1, K_{m2} = 1, k_{d1} = 200, K_{d2} = 5, K_{d1h} = 8, k_{d2h} = 4, h_2 = 1.4, A_{max} = 200, R_{max} = 500,\ n_2 = 2, n_{1h} = 1, n_{2h} = 1.25, \theta = 1, P_{T12} = 10,\ \beta_{12} = 0.001,\ \beta_1 = 0.002,\ \beta_2 = 0.001$. **Next, we fitted the experimental results of** APF, ANF loops and $P_{lux/tetO}$-based combinatorial promoter to a power-law and multiplication function Fitting to power-law and multiplication function: $\left(\frac{AHL}{1.25}\right)^{0.45} \cdot \left(\frac{aTc}{1.25}\right)^{0.55}$

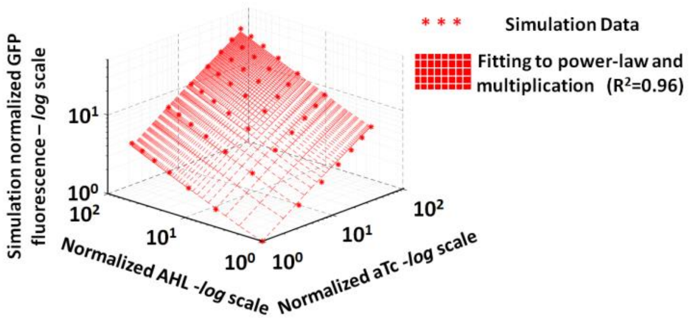

Supplementary Fig. 28. Fitting the simulation results of APF (P_{luxTGT}) and ANF (P_{tetO}) loops and combinatorial promoter ($P_{lux/tetO}$- mCherry) to power-law and multiplication function. The data appears in Fig. 2c in the main text and is reproduced here for clarity.

Now we will show that the experimental results of this circuit fit power law and multiplication function:
Fitting experimental results of APF, ANF loops and $P_{lux/tetO}$-based combinatorial promoter to a power-law and multiplication function $\left(\frac{AHL}{1.25}\right)^{0.45} \cdot \left(\frac{aTc}{1.25}\right)^{0.55}$. Then, we fitted the same experimental results to perceptgene model: $\frac{[\left(\frac{AHL}{1.25}\right)^{0.45} \cdot \left(\frac{aTc}{1.25}\right)^{0.55}/250]+0.0001}{[\left(\frac{AHL}{1.25}\right)^{0.45} \cdot \left(\frac{aTc}{1.25}\right)^{0.55}/250]+1}$

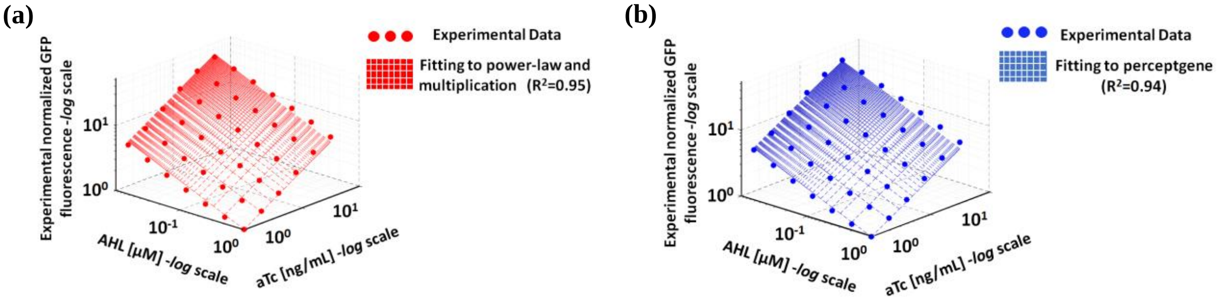

Supplementary Fig. 29. Fitting the experimental results of APF (P_{luxTGT}) and ANF (P_{tetO}) loops and combinatorial promoter ($P_{lux/tetO}$- GFP) to **(a)** power-law and multiplication function. The data appears in Fig. 2b in the main text and is reproduced here for clarity, **(b)** Perceptgene model: $\frac{[\left(\frac{AHL}{K_1}\right)^{n_1} \cdot \left(\frac{aTc}{K_2}\right)^{n_2}/K_d]^m+\beta}{[\left(\frac{AHL}{K_1}\right)^{n_1} \cdot \left(\frac{aTc}{K_2}\right)^{n_2}/K_d]^m+1}$.

Simulation results of $P_{lux/tetO}$-based perceptgene circuit (Fig. 2d) are shown in Supplementary Fig. 30. We used the same equations and parameters that describe the P_{BAD} system (Eq. 2.21-2.24), except the basal level, and assumed that $AraC_T$ is proportional to the output of $P_{lux/tetO}$-based power-law and multiplication function.

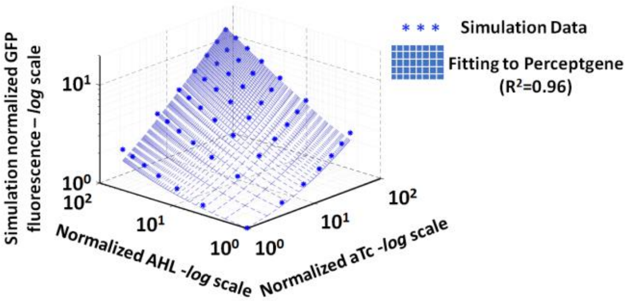

Supplementary Fig. 30. The computed transfer function of synthetic perceptgene based on AraC system. Parameters that were used in simulation: $Arab = 1, AraC_{max} = 0.5, K_{d3} = 5, K_{d4} = 50, m_3 = 2.8$ [2,14], $\beta_4 = 0.0006$. The ratio between K_{d3} and K_{d4} fits well to the values that were reported on literatures [2,14]. The fitting is according to perceptgene model :

$$\frac{[\left(\frac{AHL}{1.25}\right)^{0.45}\cdot\left(\frac{aTc}{1.25}\right)^{0.55}/1400]^{1.27}+0.00045}{[\left(\frac{AHL}{1.25}\right)^{0.45}\cdot\left(\frac{aTc}{1.25}\right)^{0.55}/1400]^{1.27}+1}.$$

Then we fitted the experimental results of AHL, aTc synthetic perceptgene based on AraC system to perceptgene model: $\frac{[\left(\frac{AHL}{1.25}\right)^{0.45}\cdot\left(\frac{aTc}{1.25}\right)^{0.55}/1400]^{1.27}+0.00045}{[\left(\frac{AHL}{1.25}\right)^{0.45}\cdot\left(\frac{aTc}{1.25}\right)^{0.55}/1400]^{1.27}+1}$.

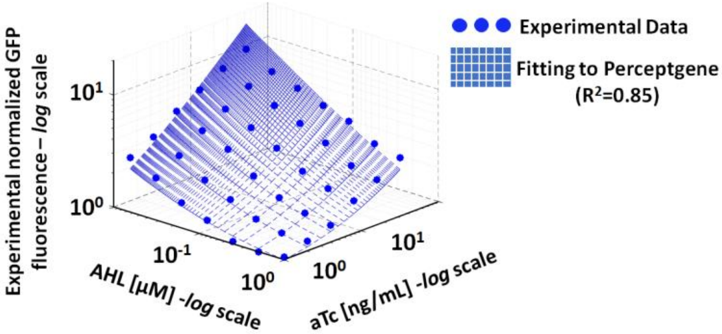

Supplementary Fig. 31. Fitting experimental results of perceptgene based APF, ANF loops and $P_{lux/tetO}$-based combinatorial promoter to perceptgene model (Circuit from Fig. 2d, Data based on Fig. 2e).

Supplementary Table 4 List of parameters used in this section

Symbol	Description
R_i	The level of repressors which are bound to P_i
A	The level of activator which are bound to P_1
X_i	The level of repressors/activator which are bound to P_i
$P_{\ i}$	Promoter within ANF
$P_{\ 1/2}$	Vombinatorial promoter
β_i	Basal level of promoter
n_i	Hill coefficient of binding of repressor/activator to $P_{\ 1}$ & $P_{\ 2}$ promoter
K_{di}	Dissociation constant of binding repressor/activator to $P_{\ 1}$ & $P_{\ 2}$ promoter
R_{Ti}	Total level of expressed R_i
R_{max_i}	The maximum protein level achieved by P_i
A_T	Total level of expressed A
A_{max}	The maximum protein level achieved by P_1
x_i	Inducer
$K_{\ mi}$	Dissociation constant of binding x_i to Y_i
$h_{\ i}$	Hill coefficients of binding x_i to Y_i
Y	The expression level of the output protein
Y_{max}	The maximum protein level achieved by $P_{1/2}$ promoter
θ	Combinatorial promoter binding interfere
K_{dh}	Dissociation constant of binding repressors to $P_{\ 1/2}$combinatorial promoter
n_h	Hill coefficient of binding repressors to $P_{\ 1/2}$ combinatorial promoter
$AraC_C$	Concentration of the Arabinose-$AraC$ complex
$AraC_T$	The total concentration of $AraC$
m_{eff}	Effective Hill coefficient
β_{12}	Basal level of $P_{\ 1/2}$ combinatorial promoter
P_{Ti}	Total concentration of P_1
P_{T12}	Total concentration of P_{12}
K_{eff}	Effective dissociation constant

Supplementary Table 5 List of abbreviations used in this section

Symbol	Description
TFs	transcription factors
ANF	auto-negative feedback
P_{BAD}	AraC promoter is activated by the $AraC$ when it is induced by arabinose (Arab)
OL	open-loop
APF	auto-positive feedback
AHL	Free N-(β-Ketocaproyl)-L-homoserine Lactone $3OC_6HSL$ concentration
Arab	Free arabinose concentration
IPTG	Free Isopropyl 1-β-D-1-thio galactopyranoside concentration
aTc	Free anhydrotetracycline
P_{lux}	LuxR promoter is activated by the $LuxR$ when it is induced by AHL
P_{lacO}	LacI promoter is activated by the $LacI - IPTG$
P_{TetO}	TetR promoter is activated by the $TetR - aTc$

3. <u>Smooth logical functions</u>

Minimum and maximum functions perform logical operations ("if" loop) to a set of analog/digital numbers. While smooth minimum and maximum functions perform the analog operation to a set of analog numbers (x_i). To implement smooth logical functions between two analog numbers, we used rectifier activation functions (Supplementary Fig. 32), which is widely used in artificial neural networks, and is given by [16,17]:

$$\min(u_{01},u)+f_{max}=\begin{cases}u+f_{max} & u<u_{01}\\ f_{max} & u>u_{01}\end{cases} \tag{3.1.1}$$

$$\max(u_{02},u)+f_{min}=\begin{cases}u+f_{min} & u>u_{02}\\ f_{min} & u<u_{02}\end{cases} \tag{3.1.2}$$

We first will prove two mathematical identities:

(1) $min(u_{01},x+y)=\min(u_{01}-y,x)+y$

Computing the left side:

1. For $u_{01}-(x+y)<0$, we obtain $min(u_{01},x+y)=u_{01}$
2. For $u_{01}-(x+y)>0$, we obtain $min(u_{01},x+y)=x+y$

Computing the right side:

1. For $u_{01}-(x+y)<0$, we obtain $\min(u_{01}-y,x)=u_{01}-y$, and, thus $\min(u_{01}-y,x)+y=u_{01}$
2. For $u_{01}-(x+y)>0$, we obtain $\min(u_{01}-y,x)=x$, and, thus $\min(u_{01}-y,x)+y=x+y$

Therefore, we obtain that the left side and the right side are equal in all the conditions.

(2) $max(u_{02},x+y)=\max(u_{02}-y,x)+y$

Computing the left side:

3. For $u_{02}-(x+y)<0$, we obtain $max(u_{02},x+y)=x+y$
4. For $u_{02}-(x+y)>0$, we obtain $min(u_{02},x+y)=u_{02}$

Computing the right side:

3. For $u_{02}-(x+y)<0$, we obtain $\min(u_{02}-y,x)=x$, and, thus $\min(u_{02}-y,x)+y=x+y$
4. For $u_{02}-(x+y)>0$, we obtain $\min(u_{02}-y,x)=u_{02}-y$, and, thus $\min(u_{02}-y,x)+y=u_{02}$

Therefore, we obtain that the left side and the right side are equal in all the conditions.

An analytical expression that approximately describes Eq. 3.1 can be given by [18]:

$$S_\alpha(u_{0i},u)=(u-u_0)\cdot\frac{e^{\alpha\cdot(u-u_0)}}{1+e^{\alpha\cdot(u-u_0)}}+f \tag{3.2.1}$$

When $\alpha<0$, Supplementary Eq. 3.3.1 finds the smooth minimum between u_0 *and* u

$$S_{-|\alpha|}(u_{01},u)=\frac{(u-u_{01})}{1+e^{+|\alpha|\cdot(u-u_{01})}}+f_{max} \quad , \quad \text{when} \quad \alpha\to-\infty \quad ➔ \quad S_{-|\alpha|}(u_{01},u)=\min(u_{01},u) \tag{3.2.2}$$

When $\alpha>0$, Eq. 3.3 finds the smooth maximum between u_0 *and* u

$$S_{|\alpha|}(u_{02},u)=\frac{(u-u_{02})}{1+e^{-|\alpha|\cdot(u-u_{02})}}+f_{min},\ \text{when}\ \alpha\to\infty\ ➔\ S_{|\alpha|}(u_{02},u)=\max(u_{02},u) \tag{3.2.3}$$

The P_{BAD} promoter can exhibit $S_{-10} = min\{u_{01}, u\}$ for high Arabinose level, and $S_4 = max\{u_{02}, u\}$ for low Arabinose level (Supplementary Fig. 32c). This data is based on Supplementary Fig. 22.

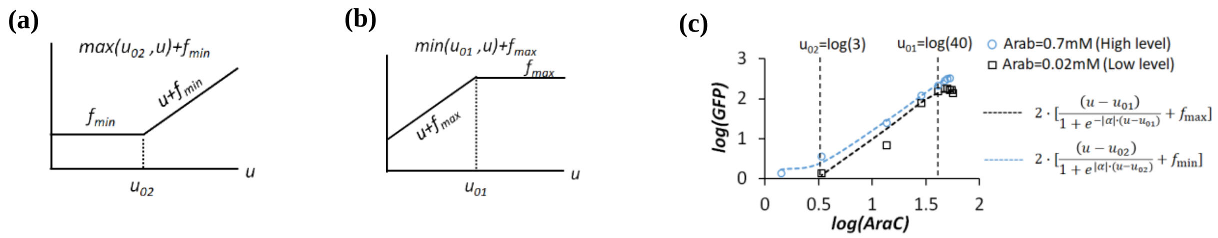

Supplementary Fig. 32. (a) negative-rectifier activation function. **(b)** positive-rectifier activation function. **(c)** Fitting the induced P_{BAD} with low Arabinose (0.02mM) to negative rectifier Eq. 3.2.2 ($\alpha = -10, u_{01} = \log(40)$, f_{max}=0.56, and with high Arabinose (0.2mM) to positive rectifier Eq. 3.2.3 ($\alpha = 4, u_{02} = \log(3)$, f_{min}=0.1. This data is based on Supplementary Fig. 22.

Analyzing log-transformed negative rectifier:
For simplicity, we assumed that $u_0 = 0, x = r, and\ y = -v$. First, we plotted the function $\min(0, u = r - v) + cont$ (Supplementary Fig. 33a). Then, we subtracted the function output by the input *v* which brings all the plots to the same initial point: $\min(0, r - v) + v + const$ (Supplementary Fig. 33b). In the next step, we graphed the function $\min(r, v)$ (Supplementary Fig. 33c), and by comparing Supplementary Fig. 33b to Fig. 33c, we concluded that these two graphs are equivalent. Therefore, eventually, the function $\min(0, r - v) + cont$, which can be implemented by a negative rectifier with a threshold that is controlled by the second analog input can be used to compute the minimum between two analog numbers. For further study of the relation between perceptron and minim function, we plotted a 2-input perceptron using a sigmoid function $\frac{e^{w\cdot(r+v-c)}}{1+e^{w\cdot(r+v-c)}}$ (Supplementary Fig. 33d), and a 2-input perceptron that is normalized by second input ($\frac{e^{w\cdot(r+v-c)}}{1+e^{w\cdot(r+v-c)}} - v$) that brings all the curves to the same initial point. In our simulation, we designed the sigmoid to act as a negative rectifier in the operating dynamic range $w = 7$ and large $c = 7$. We obtained that $\min(0, r - v) + cont$ and perceptron have similar behaviors, however, in an opposite dependency concerning the second input.
Then, we plotted the perceptgene of two inputs $\frac{bais\cdot r^{n_3}\cdot v^{n_4}+\beta}{1+bais\cdot r^{n_1}\cdot v^{n_2}}$ (Supplementary Fig. 33d, only in this case we assumed that $n_3 = n_4 = 1.5, bais = 0.001, \beta = 0.01$).

For operating in partial swing, the perceptgene can be approximated as shifted and biased log-transformed negative rectifier (NR). The NF receives the collective analog signal $k_1 \cdot \log(r) + k_2 \cdot \log(v) + \log(AraC_{max})$, where $AraC_{max}$ is the maximum AraC level. Using Eq. 3.2.2, when the bias depends linearly on the *v* level, the output of the NR:

$$f_{NR} = \frac{k_1\cdot\log(r)+k_2\cdot\log(v)+\log(AraC_{max})-\log(U_{o1})}{1+\left(\frac{b}{U_{o1}}\cdot r^{k_1}\cdot v^{k_2}\right)^{|\alpha|}} + w_1 \cdot \log(v) + const \qquad (3.3.3)$$

Where $u_{01} = \log(U_{o1})$,

(1) when: $\alpha \ll -1$, and $\frac{AraC_{max}}{U_{o1}} \cdot r^{k_1} \cdot v^{k_2} \ll 1$ ➔ $f_{NR} = k_1 \cdot \log(r) + k_2 \cdot \log(v) + \log(AraC_{max}) - \log(U_{o1}) + w_1 \cdot \log(v) + const$. In this case, we can claim that the f_{NR} is equal to the analog argument $bias \cdot r^{n_3} \cdot v^{n_4}$ at the log-scale, where n_3 and n_4 are the weights of the two inputs as calculated by the power-law and multiplication function.
(2) when: $\alpha \ll -1$, and $\frac{AraC_{max}}{U_{o1}} \cdot r^{k_1} \cdot v^{k_2} \gg 1$ ➔ $f_{NR} = w_1 \cdot \log(v) + const$

In the case of the P_{BAD} as shown in Supplementary Fig. 32c, since the promoter activity is proportional to the activity of negative rectifier, we expect that

- ⇨ $k_1 = n_3$,
- ⇨ $(k_2 + w_1) = n_4$
- ⇨ $\log(bias) = \log(AraC_{max}) - \log(U_{o1})$

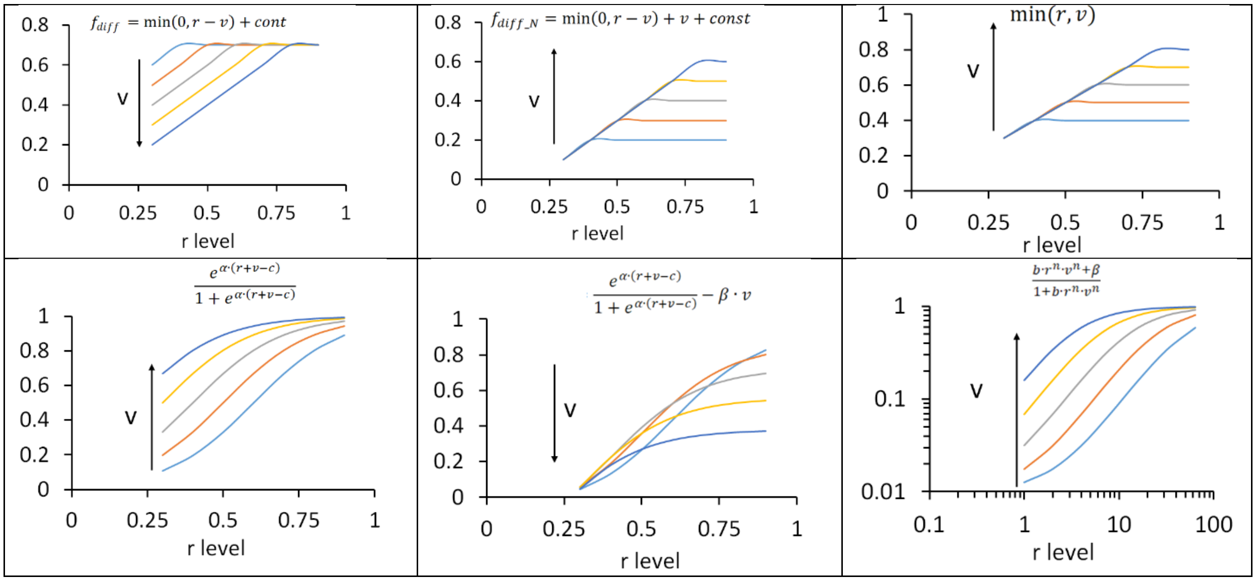

Supplementary Fig. 33. Calculations of several functions related to smooth minimum calculations

First, we normalized the measured data of three circuits from Fig. 1h and Fig. 2e, and Fig. 2h, by the minimum level achieved for each circuit. Second, we transformed Eq. 3.3 operates in the linear-scale, to the logarithmic-scale, by a logarithmic operation to the normalized data. Then, we normalized the inputs for every circuit by its input dynamic range value (x_i/IDR_i).

1. Smooth minimum function – $P_{lacO/tetO}$-based Perceptgene circuit. Supplementary Fig. 34a shows the experimental data of Fig. 1h in the linear-scale, which is well matched to Eq. 3.3:
   Where:
   $u = 0.4 \cdot x_1 + 0.2 \cdot x_2 - 1$
   $b = 0.3 \cdot x_2 + 0.6$
   $\alpha = -10 < 0$ ➔ Soft minimum
   $x_1 = \log(IPTG/IDR_3),\ x_2 = \log(aTc/IDR_4)$

   We can also build the model that that b depends on x_1 $(IPTG)$

$$f_1 = S_{-10}(0, 0.4 \cdot x_1 + 0.2 \cdot x_2 - 1) + 0.3 \cdot x_2 + 0.6 \quad (3.4.1)$$

Where f_1 is the normalized data of Fig. 1h. We can write:

$$f_1 = min(0, 0.4 \cdot x_1 + 0.2 \cdot x_2 - 1) + 0.3 \cdot x_2 + 0.6 \quad (3.4.2)$$

$$= \begin{cases} 0.4 \cdot x_1 + 0.5 \cdot x_2 - 0.4 & 0.4 \cdot x_1 + 0.2 \cdot x_2 - 1 < 0 \\ 0.3 \cdot x_2 + 0.6 & 0.4 \cdot x_1 + 0.2 \cdot x_2 - 1 > 0 \end{cases}$$

A general formula to Eq. 3.4 is:

$$f_1 = min(0, k_1 \cdot x_1 + k_2 \cdot x_2 - \gamma) + 2 \cdot w_1 \cdot x_2 + a_2 \quad (3.5)$$

$$= \begin{cases} k_1 \cdot x_1 + k_2 \cdot x_2 - \gamma + 2 \cdot w_1 \cdot x_2 + a_2 & k_1 \cdot x_1 + k_2 \cdot x_2 - \gamma < 0 \\ 2 \cdot w_1 \cdot x_2 + a_2 & k_1 \cdot x_1 + k_2 \cdot x_2 - \gamma > 0 \end{cases}$$

We can write Eq. 3.5 as:

$$f_2 = f_1 - 2 \cdot w_1 \cdot x_2 + a_2) = \begin{cases} k_1 \cdot x_1 + k_2 \cdot x_2 - \gamma & k_1 \cdot x_1 + k_2 \cdot x_2 - \gamma < 0 \\ 0 & k_1 \cdot x_1 + k_2 \cdot x_2 - \gamma > 0 \end{cases}$$

$$f_3 = f_1 - (k_2 \cdot x_2 - \gamma) - (2 \cdot w_1 \cdot x_2 + a_2) = \begin{cases} k_1 \cdot x_1 & k_1 \cdot x_1 < \gamma - k_2 \cdot x_2 \\ \gamma - k_2 \cdot x_2 & k_1 \cdot x_1 > \gamma - k_2 \cdot x_2 \end{cases} \quad (3.6)$$

Eq. 3.6 can be viewed as a smooth minimum logical function between two analog numbers that are proportional to system inputs x_1 and x_2 (Supplementary Fig. 34b):

$$f_3 = \min(\gamma - k_2 \cdot x_2, k_1 \cdot x_1) = \begin{cases} k_1 \cdot x_1 & k_1 \cdot x_1 < \gamma - k_2 \cdot x_2 \\ \gamma - k_2 \cdot x_2 & k_1 \cdot x_1 > \gamma - k_2 \cdot x_2 \end{cases} \quad (3.7)$$

Supplementary Fig. 34b and Table in Supplementary Fig, 34c give similar results with a little bit of difference, and this is because that each one is calculated in a different way.

The IPTG weight $n_3 = 0.3375$ (Supplementary Table 19), and $k_1 = 0.4$ ➔ $n_3 \approx k_1$ as we expected
The aTc weight $n_4 = 0.4375$ (Supplementary Table 19), and $k_2 = 0.2,\ w_1 = 0.3$ ➔ $n_4 \approx (k_2 + w_1)$ as we expected.

For a more general case: an ideal minimum function is observed when $min(r, v) = min(0, r - v) + v$. Thus, if we assume that $r = k_1 \cdot x_1$ and $v = \gamma - k_2 \cdot x_2$, we can find that the perceptgene computes:

$$min(0, r - v) + v + 0.5 \cdot x_2 - 0.4 ==> min(r, v) + 0.5 \cdot x_2 - 0.4 \quad (3.7.1)$$

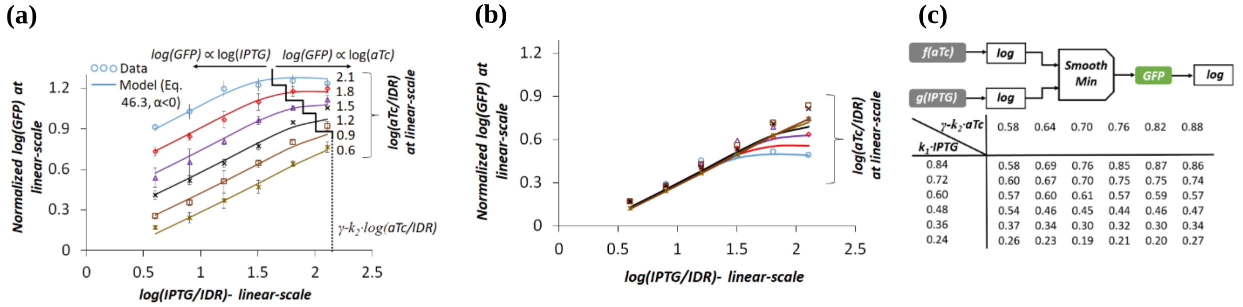

Supplementary Fig. 34. The perceptgene circuit calculates the smooth minimum between the analog inputs ($\log(IPTG)$ *and* $\log(aTc)$). **(a)** the raw data, Where $\gamma = 0\log(B_2)$, $k_2 = (n_4 - w_1)$. Data are presented as average ±standard deviations from independent replicates ($n = 3$). **(b)** the raw data after bringing all the curves to the same initial minimum point, Similar to Supplementary Fig. 33. **(c)** Raw data using Supplementary Eq. 3.7.

We then calculated the errors between the transformed data of the perceptgene circuit and the ideal data of smooth functions.

Supplementary Table 6. Transformed experimental data (D_{exp}) of Fig. 1h (Supplementary Fig. 34)

Experimental Results	**0.58**	**0.64**	**0.70**	**0.76**	**0.82**	**0.88**
**0.84**	0.58	0.69	0.76	0.85	0.87	0.86
**0.72**	0.60	0.67	0.70	0.75	0.75	0.74
**0.60**	0.57	0.60	0.61	0.57	0.59	0.57
**0.48**	0.54	0.46	0.45	0.44	0.46	0.47
**0.36**	0.37	0.34	0.30	0.32	0.30	0.34
**0.24**	0.26	0.23	0.19	0.21	0.20	0.27

Supplementary Table 7. Ideal (Expected) data for minimum function (D_{min-1}) based on Supplementary Table 6

**Expected** MIN	**0.58**	**0.64**	**0.70**	**0.76**	**0.82**	**0.88**
**0.84**	0.58	0.64	0.70	0.76	0.82	0.84
**0.72**	0.58	0.64	0.70	0.72	0.72	0.72
**0.60**	0.58	0.60	0.60	0.60	0.60	0.60
**0.48**	0.48	0.48	0.48	0.48	0.48	0.48
**0.36**	0.36	0.36	0.36	0.36	0.36	0.36
**0.24**	0.24	0.24	0.24	0.24	0.24	0.24

Supplementary Table 8. Calculation of the error between measurement data (Supplementary Table 6) and Ideal data for the minimum function (Supplementary Table 7) using:

$$Error_i = abs\left(\frac{(D_{exp} - D_{min-1})}{D_{min-1}}\right)$$

Error(i)	**0.58**	**0.64**	**0.70**	**0.76**	**0.82**	**0.88**
**0.84**	0.001622052	0.086931	0.089926	0.122127	0.06111	0
**0.72**	0.040504146	0.054275	0.007044	0.036687	0.037627	0.029814
**0.60**	0.017410123	0.007701	0.015816	0.050489	0.011948	0.049949
**0.48**	0.127994603	0.031549	0.059475	0.077629	0.042186	0.025321
**0.36**	0.441393616	0.312972	0.158067	0.219982	0.163661	0.317077
**0.24**	0.013971729	0.124459	0.282187	0.205529	0.217792	0.038107

The error for the experiment is calculated as the:

$$Error = 100 \times \frac{1}{N} \sum_{i=1}^{N} Error_i$$

➔ 10%

Supplementary Table 9. Ideal data for maximum function (D_{max-1}) based on Supplementary Table 6

**Expected MAX**	**0.58**	**0.64**	**0.70**	**0.76**	**0.82**	**0.88**
**0.84**	0.84	0.84	0.84	0.84	0.84	0.88
**0.72**	0.72	0.72	0.72	0.76	0.82	0.88
**0.60**	0.60	0.64	0.70	0.76	0.82	0.88
**0.48**	0.58	0.64	0.70	0.76	0.82	0.88
**0.36**	0.58	0.64	0.70	0.76	0.82	0.88
**0.24**	0.58	0.64	0.70	0.76	0.82	0.88

Supplementary Table 10. Calculation of the error between measurement data (Supplementary Table 6) and Ideal data for the maximum function (Supplementary Table 9) using:

$$Error_i = abs\left(\frac{(D_{exp} - D_{max-1})}{D_{min-1}}\right)$$

Error(i)	**0.58**	**0.64**	**0.70**	**0.76**	**0.82**	**0.88**
**0.84**	0.310123265	0.173461	0.093065	0.014157	0.035065	0.019391
**0.72**	0.163899995	0.064676	0.02237	0.017875	0.088913	0.157425
**0.60**	0.050163119	0.067919	0.128017	0.250386	0.277035	0.352238
**0.48**	0.066487225	0.272256	0.354118	0.41745	0.439328	0.468357
**0.36**	0.353858034	0.465573	0.569227	0.582638	0.631034	0.610863
**0.24**	0.557987327	0.643623	0.732991	0.728207	0.751983	0.693287

The error for the experiment is calculated using Eq. 3.25 ➔ 32%

Supplementary Table 11. Ideal data for the average function (D_{ave-1}) based on Supplementary Table 6

**Expected Average**	**0.58**	**0.64**	**0.70**	**0.76**	**0.82**	**0.88**
**0.84**	0.71	0.74	0.77	0.80	0.83	0.86
**0.72**	0.65	0.68	0.71	0.74	0.77	0.80
**0.60**	0.59	0.62	0.65	0.68	0.71	0.74
**0.48**	0.53	0.56	0.59	0.62	0.65	0.68
**0.36**	0.47	0.50	0.53	0.56	0.59	0.62
**0.24**	0.41	0.44	0.47	0.50	0.53	0.56

Supplementary Table 12. Calculation the error between measurement data (Supplementary Table 6) and Ideal data for average function (Supplementary Table 11) using:

$$Error_i = abs\left(\frac{(D_{exp}-D_{ave-1})}{D_{min-1}}\right)$$

**Error(i)**	**0.58**	**0.64**	**0.70**	**0.76**	**0.82**	**0.88**
**0.84**	0.1846358	0.06281	0.011806	0.063495	0.046108	0.001974
**0.72**	0.074591661	0.010556	0.009607	0.007547	0.030918	0.074359
**0.60**	0.034569811	0.040348	0.063066	0.162957	0.165873	0.230545
**0.48**	0.021381523	0.170205	0.235228	0.28638	0.293251	0.312616
**0.36**	0.202458539	0.317255	0.432002	0.433788	0.487469	0.448044
**0.24**	0.374242173	0.482392	0.602825	0.586875	0.61635	0.518199

The error for the experiment is calculated using Supplementary Eq. 3.25 ➔ 22%

Supplementary Table 13. summarized the data

Std err (experimental vs. expected)	Smooth Min (expected)	Smooth Avg (expected)	Smooth Max (expected)
Smooth Min (experimental)	10%	22%	32%

Analyzing log-transformed positive rectifier:

For simplicity, we assumed that $u_0 = 0, x = r, and\ y = -v$. First, we plotted the function $\max(0, u = r - v) + const$ (Supplementary Fig. 35a). Then, we subtracted the function output by the input v which brings all the plots to the same initial point: $\max(0, r - v) + v + const$ (Supplementary Fig. 35b). In the next step, we graphed the function $\max(r, v)$ (Supplementary Fig. 35c), and by comparing Supplementary Fig. 35b, c, we concluded that these two graphs are equivalent. Therefore, eventually, the function $\max(0, r - v) + const$, which can be implemented by a positive rectifier with a threshold that is controlled by the second analog input, can be used to compute the maximum between two analog numbers. For further study of the relation between perceptron and maximum function, we plotted a 2-input perceptron using a sigmoid function $\frac{e^{\alpha\cdot(r+v-c)}}{1+e^{\alpha\cdot(r+v-c)}}$ (Supplementary Fig. 35d), and a 2-input perceptron that is normalized by second input ($\frac{e^{\alpha\cdot(r+v-c)}}{1+e^{\alpha\cdot(r+v-c)}} - v$) that brings all the curves to the same initial point. In our simulation, we designed the sigmoid to act as a positive rectifier in the operating dynamic range $\alpha = 7$ and large $c = 1.5$. We obtained that $\max(0, r - v) + const$ and perceptron have similar behavior, however, in an opposite relation with the second input.

Then, we plotted the perceptgene of two inputs $\frac{bias\cdot r^{n_1}\cdot v^{n_2}+\beta}{1+bias\cdot r^{n_1}\cdot v^{n_2}}$ (Supplementary Fig. 33d, only in this case we assumed that $n_1 = n_2 = 1.5, bias = 0.00005, \beta = 0.01$).

For operating in partial swing, the perceptgene can be approximated as shifted and biased log-transformed positive rectifier (PR). The PF receives the collective analog signal $k_1 \cdot \log(r) + k_2 \cdot \log(v) + \log(AraC_{max})$, where $AraC_{max}$ is the maximum AraC level. Using Eq. 3.2.3, when the bias depends linearly on the v level, the output of the PR:

$$f_{NR} = \frac{k_1\cdot\log(r)+k_2\cdot\log(v)+\log(AraC_{max})-\log(U_{o2})}{1+\left(\frac{AraC_{max}}{U_{o2}}\cdot r^{k_1}\cdot v^{k_2}\right)^{-|\alpha|}} + w_2 \cdot \log(r) + const \qquad (3.8)$$

Where $u_{02} = \log(U_{o2})$,

(3) when: $\alpha \gg 1$, and $\frac{AraC_{max}}{U_{o2}} \cdot r^{k_1} \cdot v^{k_2} \gg 1$ ➔ $f_{PR} = k_1 \cdot \log(r) + k_2 \cdot \log(v) + \log(AraC_{max}) - \log(U_{o2}) + 2 \cdot w_2 \cdot \log(r) + const$. In this case, we can claim that the f_{PR} is equal to the analog argument $bias \cdot r^{n_5} \cdot v^{n_6}$ at the log-scale., where n_5 and n_6 are the are the weights of the two inputs as calculated by the power-law and multiplication function.

⇨ $k_1 = n_1$,

⇨ $k_2 + k_3 = n_2$

(4) $B = b$when: $\alpha \gg 1$, and $\frac{AraC_{max}}{U_{o2}} \cdot r^{k_1} \cdot v^{k_2} \ll 1$ ➔ $f_{PR} = w_2 \cdot \log(r) + const$

In the case of the P_{BAD} as shown in Supplementary Fig. 32c, since the promoter activity is proportional to the activity of positive rectifier, we expect that

⇨ $k_2 = n_6$,

⇨ $k_1 + w_2 = n_5$

⇨ $\log(bias) = \log(AraC_{max}) - \log(U_{o2})$

Smooth maximum function – $P_{lux/tetO}$-based Perceptgene circuit. Supplementary Fig. 36a. shows the experimental data of Fig. 2e in the linear-scale, is well matched to Eq. 3.3, where

$u_1 = 0.25 \cdot x_1 + 0.38 \cdot x_2 - 0.2$

$b = 0.25 \cdot x_1 + 0.1$

$\alpha = 4 > 0$ ➔ soft maximum

$x_1 = \log(AHL/IDR_5), x_2 = \log(aTc/IDR_6)$

We can also build the model that b depends on x_2 (aTc)

$$f_1 = S_4(0.25 \cdot x_1 + 0.38 \cdot x_2 - 0.2) + 0.22 \cdot x_1 + 0.1 \qquad (3.8.1)$$

Where f_1 is the normalized data of Fig. 2e. We can write:

$$f_1 = max(0{,}0.25 \cdot x_1 + 0.38 \cdot x_2 - 0.2) + 0.22 \cdot x_1 + 0.1 \qquad (3.8.2)$$

$$= \begin{cases} 0.25 \cdot x_1 + 0.38 \cdot x_2 - 0.2 & 0.25 \cdot x_1 + 0.38 \cdot x_2 - 0.2 > 0 \\ 0.22 \cdot x_1 + 0.1 & 0.25 \cdot x_1 + 0.38 \cdot x_2 - 0.2 < 0 \end{cases}$$

Similar to Eq. 3.7 we can write Eq. 3.8 as:

$$f_1 = \max(k_2 \cdot x_2, \gamma - k_1 \cdot x_1) = f_1 - (k_1 \cdot x_1 - \gamma) - (2 \cdot w_2 \cdot x_1 + a_2)$$

$$= \begin{cases} k_1 \cdot x_1 & k_1 \cdot x_1 > \gamma - k_2 \cdot x_2 \\ \gamma - k_2 \cdot x_2 & k_1 \cdot x_1 < \gamma - k_2 \cdot x_2 \end{cases} \qquad (3.9)$$

Eq. 3.9 can be viewed as a smooth maximum logical function between two analog numbers that are proportional to system inputs x_1 and x_2 (Supplementary Fig. 36b).

The aTc weight $n_6 = 0.45$, and $k_2 = 0.38 \rightarrow n_6 \approx k_2$

The AHL weigh $n_5 = 0.55$, and $k_1 = 0.25,\ w_2 = 0.22 \rightarrow n_5 \approx k_1 + w_2$

For a more general case: an ideal maximum function is observed when $max(r, v) = max(0, r - v) + v$. Thus, if we assume that $r = k_2 \cdot x_2$ and $v = \gamma - k_1 \cdot x_1$, we can find that the perceptgene computes:

$$max(0, r - v) + v + 0.47 \cdot x_1 - 0.1 \quad \rightarrow max(r, v) + 0.47 \cdot x_1 - 0.1 \qquad (3.9.1)$$

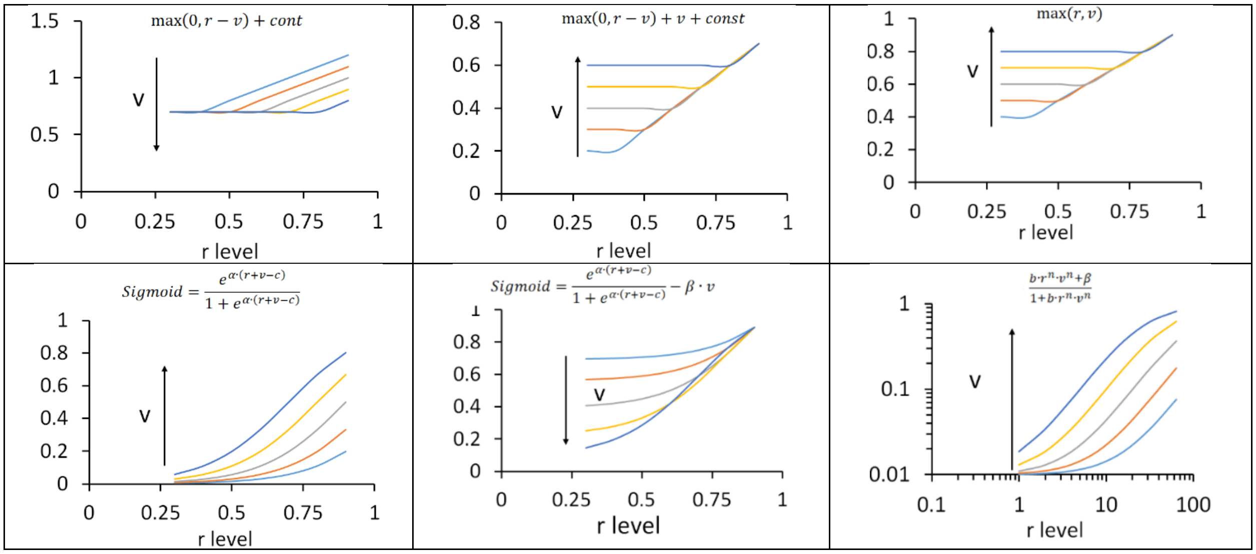

Supplementary Fig. 35. Calculations of several functions related to smooth maximum calculations.

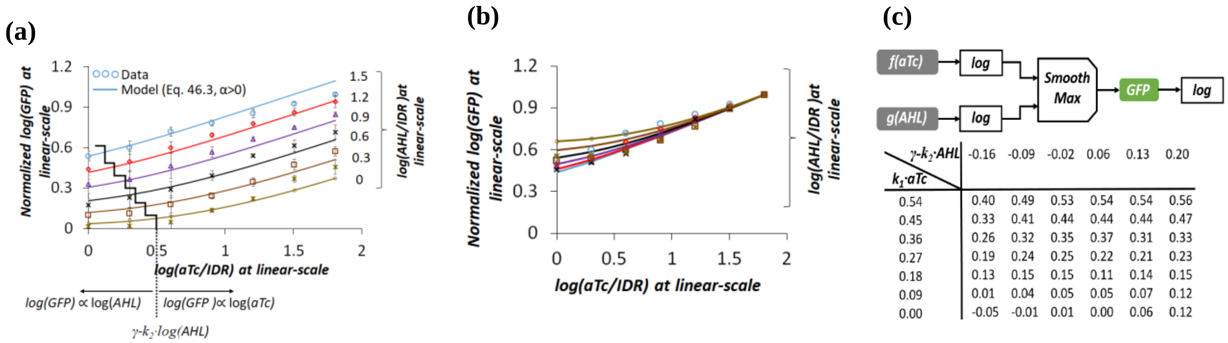

Supplementary Fig. 36. The perceptgene circuit calculates the smooth maximum between the analog inputs $(\log(AHL), \log(aTc))$. **(a)** the raw data, where $\gamma = \log(B_3)$, $k_2 = n_5 - w_2$. Data are presented as average ±standard deviations from independent replicates ($n = 3$). **(b)** the raw data after bringing all the curves to same maximum point, Similar to Supplementary Fig. 35. **(c)** Raw data using Supplementary Eq. 3.9.

Similar calculations of error were performed to the maximum circuit.

Supplementary Table 14. summarized our results

Std err (experimental vs. expected)	Smooth Min (expected)	Smooth Avg (expected)	Smooth Max (expected)
Smooth Max (experimental)	470%	256%	23%

A simple model for the average function:

The output of the power-law and multiplication circuit (Supplementary Fig. 37a) is given by:

$$Y = Y_N \left(\frac{AHL}{IDR_1}\right)^{n_7} \cdot \left(\frac{IPTG}{IDR_2}\right)^{n_8} \quad (3.10)$$

Where Y_N is a normalized parameter with a unit of concentration, IDR_i are the input dynamic ranges. Applying log operation, we get:

$$\log(Y) = \log(Y_N) + n_7 \cdot log\left(\frac{AHL}{IDR_1}\right) + n_8 \cdot log\left(\frac{IPTG}{IDR_2}\right) \quad (3.11)$$

In case that $n_7 = n_8 = 0.5$, we get:

$$Out = \log(Y) - \log(Y_N) = \frac{log\left(\frac{AHL}{IDR_1}\right) + log\left(\frac{IPTG}{IDR_2}\right)}{2} \quad (3.12)$$

Therefore, conceptually, we can implement average only with the power-law and multiplication, without the need for activation function. However, it is challenging to obtain IPTG weight around 0.5. We can solve this challenge by applying a linear activation function using AraC, P_{BAD} and Arabinose (Supplementary Fig. 37b). Equations that describe circuit from Supplementary Fig. 37b are:

$$AraC_T = AraC_N \left(\frac{AHL}{IDR_1}\right)^{n_7} \cdot \left(\frac{IPTG}{IDR_2}\right)^{n_8} \quad (3.13.1)$$

$$GFP = GFP_{max} \frac{\frac{AraC_T}{K_{d3}} + \beta_4}{1 + \frac{AraC_T}{K_{d3}}} \quad (3.13.2)$$

Eq. 3.13.2 is driven from Eq. 2.21, and Eq. 2.22, where the arabinose concentration is very high (Arab>>K_{m3}), β_4<<1, is the basal level of P_{BAD} promoter, GFP_{max} is the maximum GFP achieved by P_{BAD} promoter, $AraC_N$ is corresponding to Y_N. The Eq. 3.13.2, or the P_{BAD} promoter can operate in two linear ranges:

1. $\frac{AraC_T}{K_{d3}} \ll 1$, in this range, we can approximate Eq. 3.13.2 as:

$$GFP = GFP_{max} \left(\frac{AraC_T}{K_{d3}} + \beta_4\right) \quad (3.13.3)$$

Substituting Eq. 3.13.1 into Eq. 3.13.3, we get:

$$\frac{GFP}{GFP_{max}} = \left(\frac{AraC_N}{K_{d3}}\right) \cdot \left(\frac{AHL}{IDR_1}\right)^{n_7} \cdot \left(\frac{IPTG}{IDR_2}\right)^{n_8} + \beta_4 \quad (3.14)$$

Applying a log-operation into 3.14 and in case that $n = n_7 = n_8$, we get:

$$log\left(\frac{GFP}{GFP_{max}} - \beta_4\right) - log\left(\frac{AraC_N}{K_{d3}}\right) = n\left(log\left(\frac{AHL}{IDR_1}\right) + log\left(\frac{IPTG}{IDR_2}\right)\right) \quad (3.15)$$

In this working range, the linear activation function based on P_{BAD} could not solve the challenge of achieving low weights. These are two solutions, (1) working with an activation function with very low hill coefficient, which is not simple to create. (2) working with another working range:

2. Second analog working range: $\beta_4 < \frac{AraC_T}{K_{d3}} < 1$. For simplicity, in this analysis, we neglect the basal level, and applying a log operation to Eq. 3.13.2, we get:

$$\log\left(\frac{GFP}{GFP_{max}}\right) = \log\left(\frac{AraC_T}{K_{d3}}\right) - \log\left(1 + \frac{AraC_T}{K_{d3}}\right) \quad (3.16)$$

The slope in the log-log is equal:

$$m_{eff} \equiv \frac{d\left[\log\left(\frac{GFP}{GFP_{max}}\right)\right]}{d\left[log\left(\frac{AraC_T}{K_{d3}}\right)\right]} = 1 - \frac{\frac{AraC_T}{K_{d3}}}{1 + \frac{AraC_T}{K_{d3}}} \quad (3.17)$$

Our goal now is to approximate Eq. 3.17 when $\beta_4 < \frac{AraC_T}{K_{d3}} < 1$.

By applying Taylor series around $\frac{AraC_T}{K_{d3}} \approx 1$, we get (Supplementary Fig. 38):

$$\frac{\frac{AraC_T}{K_{d3}}}{1 + \frac{AraC_T}{K_{d3}}} \approx \frac{1}{2} + \frac{1}{4} \cdot log\left(\frac{AraC_T}{K_{d3}}\right) \quad (3.18)$$

Substituting Eq. 3.18 into Eq. 3.17, we get

$$m_{eff} = \frac{1}{2} - \frac{1}{4} \cdot log(\frac{AraC_T}{K_{d3}}) \quad \text{(S3.19.1)}$$

$$GFP = GFP_{max} \cdot \left(\frac{AraC_T}{K_{d3}}\right)^{m_{eff}} \quad \text{(3.19.2)}$$

Substituting Eq. 3.13.1 into Eq. 3.19.2, we get:

$$\frac{GFP}{GFP_{max}} = GFP_{max} \cdot \left(\frac{AraC_N}{K_{d3}}\right)^{m_{eff}} \cdot \left(\frac{AHL}{IDR_1}\right)^{n_7 \cdot m_{eff}} \cdot \left(\frac{IPTG}{IDR_2}\right)^{n_8 \cdot m_{eff}} \quad \text{(3.20)}$$

Applying log operation, we get:

$$\log\left(\frac{GFP}{GFP_{max}}\right) - m_{eff} \cdot log\left(\frac{AraC_N}{K_{d3}}\right) = n_7 \cdot m_{eff} \cdot log\left(\frac{AHL}{IDR_1}\right) + n_8 \cdot m_{eff} \cdot log\left(\frac{IPTG}{IDR_2}\right) \quad \text{(3.21)}$$

So that Eq. 3.21 computes the average, we require:

1. $n_7 = n_8 \equiv n$
2. $n \cdot m_{eff} = 0.5$ (Substituting from Eq. 3.19.1)

⇨ $n \cdot \left(\frac{1}{2} - \frac{1}{4} \cdot log(\frac{AraC_T}{K_{d3}})\right) = 0.5$

⇨ $\log\left(\frac{AraC_T}{K_{d3}}\right) = 2 \cdot \left(1 - \frac{1}{n}\right)$

⇨ $$\frac{AraC_T}{K_{d3}} = 10^{2 \cdot \left(1 - \frac{1}{n}\right)} \quad \text{(3.22)}$$

Numbers based on Supplementary Fig. 39: $n = 0.9, m = 0.95$ ➔ $AraC_T \approx 0.6K_{d3}$, $m_{eff} \approx 0.5$. Under these conditions:

$$\log\left(\frac{GFP}{GFP_{max}}\right) - Const = 0.5 \cdot log\left(\frac{AHL}{IDR_1}\right) + 0.5 \cdot log\left(\frac{IPTG}{IDR_2}\right) \quad \text{(3.23)}$$

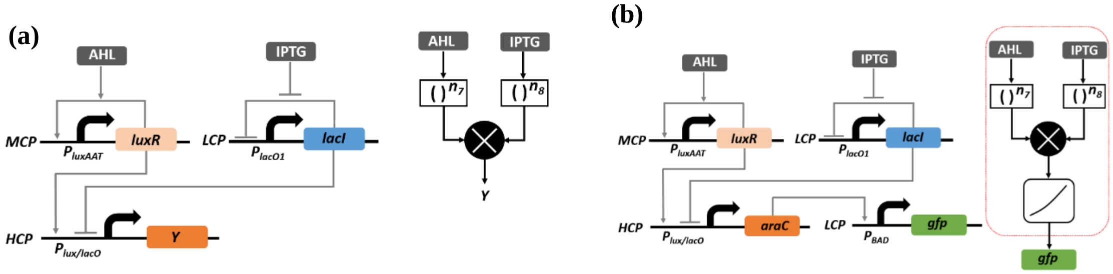

Supplementary Fig. 37. (a) APF (P_{luxAAT}), ANF (P_{lacO1}), and $P_{lux/lacO}$-based Combinatorial promoter. **(b)** Preceptgene based on linear activation function to implement average.

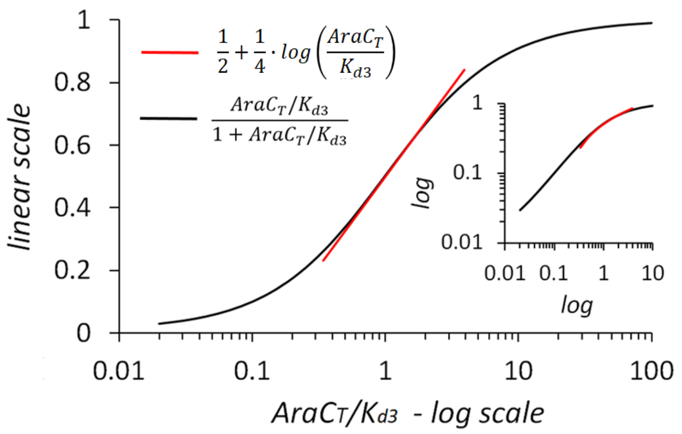

Supplementary Fig. 38. The approximation of Supplementary Eq. 3.18, the inset Supplementary Fig 38. is a representation in the log-log scale.

Experimental results of APF (P_{luxAAT}), ANF (P_{lacO1}) and $P_{lux/lacO}$-based Combinatorial promoter:

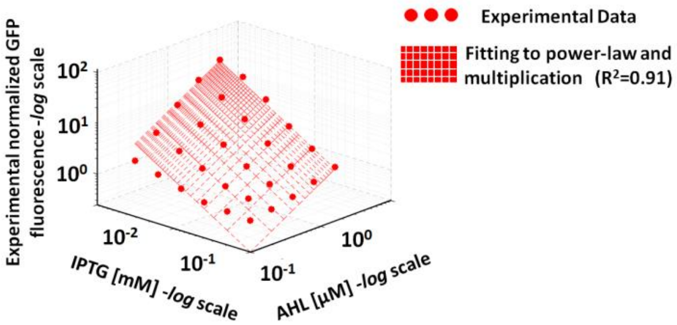

Supplementary Fig. 39. Experimental results of circuit Supplementary Fig. 37. The data represent means calculated from three experiments. Fitting experimental results of APF, ANF loops and $P_{lux/lacO}$-based combinatorial promoter to a power-law and multiplication function $\left(\frac{AHL}{6}\right)^{0.85} \cdot \left(\frac{IPTG}{7.2}\right)^{0.95}$.

Fitting experimental results of P_{luxAAT}-based APF, P_{lacO1}-based ANF loops and $P_{lux/lacO}$-based combinatorial promoter to power-law and multiplication function $\left(\frac{AHL}{6}\right)^{0.45} \cdot \left(\frac{IPTG}{7.2}\right)^{0.42}$ (Supplementary Fig. 40a). Then we fitted the experimental results of system to perceptgene model: $\frac{[\left(\frac{AHL}{6}\right)^{0.45} \cdot \left(\frac{IPTG}{7.2}\right)^{0.42}/400]^{0.95}+0.0001}{[\left(\frac{AHL}{6}\right)^{0.45} \cdot \left(\frac{IPTG}{7.2}\right)^{0.42}/400]^{0.95}+1}$ (Supplementary Fig. 40b)

(a)

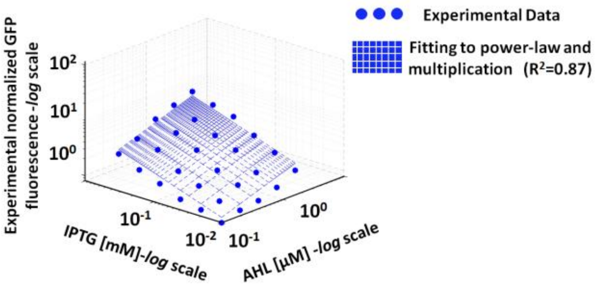

(b)

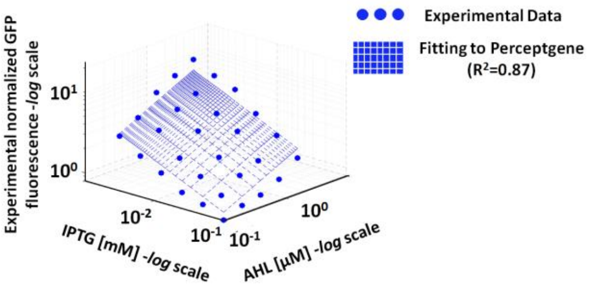

Supplementary Fig. 40. Fitting the experimental results of APF (P_{luxAAT}) and ANF (P_{lacO1}) loops and combinatorial promoter ($P_{lux/lacO}$-GFP) to **(a)** power-law and multiplication function, **(b)** perceptgene model.

2. Average function – $P_{lux/lacO}$-based Perceptgene circuit. To implement the average function between two analog numbers, we used a linear activation function (Supplementary Fig. 41a). A linear activation function is a special case of Eq. 3.3 with $\alpha \approx 0$. Supplementary Fig. 41b shows the experimental data of Fig. 2h in the linear-scale, which is well matched to Eq. 3.3 with $\alpha \approx 0$ (Supplementary Fig. 39b):
   $f_1 = 0.55 \cdot x_1 + 0.51 \cdot x_2 - 0.25$
   ⇨ $f_1 + 0.25 \cong \frac{x_1+x_2}{2}$

The power-law and multiplication functions set the input dynamic range of the smooth logic functions. For example, the average function has an IDR of $\log(32)$ order of magnitude for IPTG and the multiplication function has a IDR of $\log(16)$ order of magnitude for AHL.

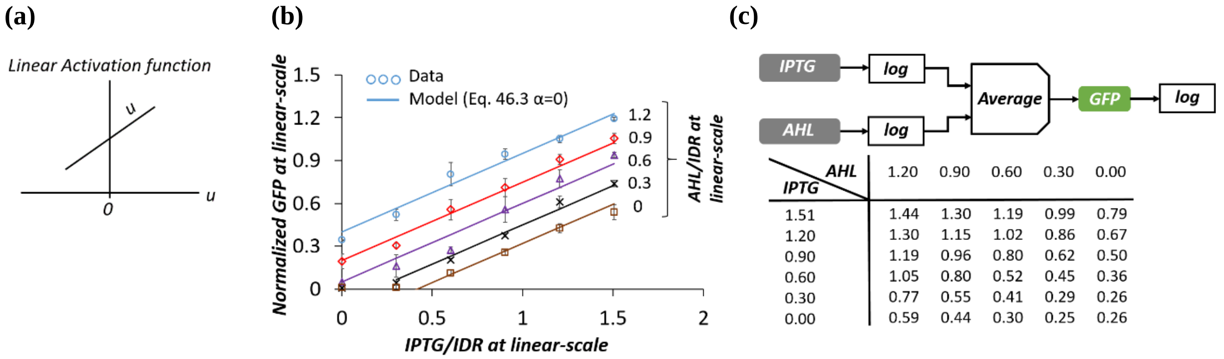

Supplementary Fig. 41. (a) Linear activation function. **(b)** The perceptgene circuit calculates the average between the analog inputs ($\log(AHL), \log(IPTG)$). Data are presented as average ±standard deviations from independent replicates ($n = 3$). **(c)** Raw data.

Similar calculations of error were performed on the average circuits.

Supplementary Table 15 summarized our results

Std err (experimental vs. expected)	Smooth Min (expected)	Smooth Avg (expected)	Smooth Max (expected)
Smooth Avg (experimental)	66%	8.5%	24%

Supplementary Table 16 summarized all the results

Std err (experimental vs. expected)	Smooth Min (expected)	Smooth Avg (expected)	Smooth Max (expected)	Circuits
Smooth Min (experimental)	10%	22%	32%	Inputs: IPTG, aTc<br>ANF (P_{lacO1}), ANF (P_{tetO}), combinatorial promoter ($P_{lacO/tetO}$), AraC /P_{BAD}-GFP<br>Arabinose-low<br>Circuit – Fig. 1g<br>Results: Fig. 1h<br>Transformed results: Fig. 1j
Smooth Max (experimental)	470%	256%	23%	Inputs: AHL, aTc<br>APF (P_{luxTGT}), ANF (P_{tetO}), combinatorial promoter ($P_{lux/tetO}$), AraC /P_{BAD}-GFP<br>Arabinose-high<br>Circuit – Fig. 2d<br>Results: Fig. 2e<br>Transformed results: Fig. 2f
Smooth Avg (experimental)	66%	8.5%	24%	Inputs: AHL, IPTG

				APF (P_{luxAAT}), ANF (P_{lacO}), combinatorial promoter ($P_{lux/tetO}$), AraC /P_{BAD}-GFP<br>Arabinose-high<br>Circuit – Fig. 2g<br>Results: Fig. 2h<br>Transformed results: Fig. 2i

Supplementary Table 17 List of abbreviations used in this section

Symbol	Description
x_i	Analog numbers
$P_{lacO/tetO}$	combinatorial promoter
$IPTG$	Free Isopropyl 1-β-D-1-thio galactopyranoside concentration
aTc	Free anhydrotetracycline
IDR	Input dynamic range
$P_{lux/tetO}$	combinatorial promoter
$P_{lux/lacO}$	combinatorial promoter

4. Calculations of parameters for a single perceptgene

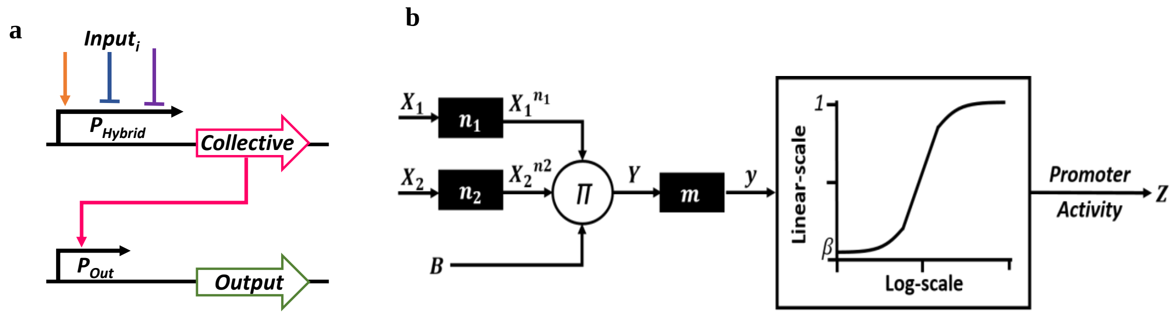

Supplementary Fig. 42. (a) Structure of a multi-input perceptgene, **(b)** Mathematical model.

The output of power-law and multiplication circuit can be approximated as:

$$Y = \prod_{i=1}^{N} Y_m \cdot \left(\frac{X_i}{K_{mi}}\right)^{n_i} \tag{4.1}$$

Where X_i is the input concentration, K_{mi} is the dissociation constant. Y_m has units of concentration, and it equals the maximum level of produced transcription factors. n_i is Hill-coefficient of the input X_i. The operation range of the circuit is defined $X_{Li} < X_i < X_{Hi}$, where $IDR = \log(\frac{X_{Hi}}{X_{Li}})$. The relevant parameters used in models are listed in Supplementary Table 18.

Supplementary Table 18. The Lowest input values were used in our circuits, and the dissociation constants for binding IPTG -LacI, AHL -LuxR, and aTc –TetR

System [Inpu1, Input2]	[IPTG, aTc]	[AHL, aTc]	[AHL, IPTG]
X_{L1}	$1\ \mu M$	$90\ nM$	$90\ nM$
K_{m1}	$1\ \mu M$ [19]	$125\ nM$ [14]	$125\ nM$
X_{L2}	$0.4\ ng/mL$	$0.4\ ng/mL$	$1\ \mu M$
K_{m2}	$1.7\ ng/mL$ [14]	$1.7\ ng/mL$	$1\ \mu M$

The measured signal of the power-law and multiplication circuit is given:

$$GFP \approx \xi \cdot \prod_{i=1}^{N} Y_m \cdot \left(\frac{X_i}{X_{Li}}\right)^{n_i} \tag{4.2}$$

Where ξ is the efficiency of converting GFP molecules to optical signals. The minimum measured signal achieved is when $X_i = X_{Li}$:

$$GFP_{min} = \xi \cdot \prod_{i=1}^{N} Y_m \cdot \left(\frac{X_{Li}}{K_{mi}}\right)^{n_i} \tag{4.3}$$

Then, the normalized signal of the power-law and multiplication circuit is given by:

$$GFP_N = \frac{GFP}{GFP_{min}} = \prod_{i=1}^{N} \left(\frac{X_i}{X_{Li}}\right)^{n_i}$$

$$\log(GFP_N) = n_i \cdot \sum_{i=1}^{N} \left[\log\left(\frac{x_i}{x_{Li}}\right)\right] \quad (4.4)$$

The promoter activity is initiated when the transcription factor Y binds and is given by:

$$P_r = \frac{\left(\frac{Y}{K_d}\right)^m + \beta}{1+\beta+\left(\frac{Y}{K_d}\right)^m} \quad (4.5)$$

β is the basal level of the promoter, K_d is the dissociation constant of binding Y to promoter, and $\boldsymbol{m}$ is the Hill-coefficient (number of binding sites within the promoter). Substituting Eq. 4.2 into Eq. 4.6 gives:

$$P_r = \frac{\left(\frac{\prod_{i=1}^{N} Y_m \cdot \left(\frac{X_i}{K_{mi}}\right)^{n_i}}{K_d}\right)^m + \beta}{1+\beta+\left(\frac{\prod_{i=1}^{N} Y_m \cdot \left(\frac{X_i}{K_{mi}}\right)^{n_i}}{K_d}\right)^m}$$

$$P_r = \frac{\left(\prod_{i=1}^{N} \frac{Y_m}{K_d} \cdot \left(\frac{X_i}{K_{mi}}\right)^{n_i}\right)^m + \beta}{1+\beta+\left(\prod_{i=1}^{N} \frac{Y_m}{K_d} \cdot \left(\frac{X_i}{K_{mi}}\right)^{n_i}\right)^m} \quad (4.6)$$

$$y = \left(\prod_{i=1}^{N} B \cdot x_i^{n_i}\right)^m \quad (4.7)$$

$$P_r = \frac{y+\beta}{1+\beta+y} \quad (4.8)$$

where $x_i = X_i/K_{mi}$, and $B = Y_m/K_d$ .Based on the Supplementary Table 18.1, we can assume that $X_{Li} \approx K_{mi}$, then we can normalize the input by X_{Li}.

The minimum promoter activity is $P_{min} \approx \beta$, and the maximum promoter activity is $P_{max} \approx 1$. The perceptgene is designed as a modular, meaning that the output of the first layer acts as the input of the second layer. Also, the decision at the perceptgene output should be made at the logarithmic-scale. Therefore, we normalized the promoter activity by the basal level:

$1 \le P_r \le 1/\beta$ ➔ linear-scale/log transform: $0 \le \log(P_r) \le -\log(\beta)$ (4.9)

In analogy to perceptron, we approximated the activation function as a step function:

$$\begin{cases} m \cdot n_i \cdot \sum_{i=1}^{N} x_i - m \cdot B \ge \log(Th) & \log(P_r) = -\log(\beta) \\ \text{Otherwise} & \log(P_r) = 0 \end{cases} \quad (4.10)$$

We define the Th is the effective threshold of the activation function and is set by the Basel level (Supplementary Fig. 43):

$$10^{log(\beta)/2} = \frac{Th+\beta}{1+Th+\beta} \quad (4.11)$$

$$Th = \frac{10^{log(\beta)/2} - \beta}{1 - 10^{log(\beta)/2}}$$

The measured signal of the perceptgene circuit in steady state is given:

$$GFP = \xi \cdot GFP_{max} \cdot P_r \quad (4.12)$$

where GFP_{max} is the maximum GFP achieved by the promoter. Then the normalized signal of the preceptgene circuit is given by:

$$GFP_N = \frac{GFP}{GFP_{min}} = 1 + \frac{\xi \cdot GFP_{max} \cdot P_r}{\xi \cdot GFP_{max} \cdot (B^{N+m} + \beta)} \quad (4.13)$$

Here GFP_{min} is the minimum GFP achieved by the perceptgene ($x_i = 1$). The maximum fold change of the perceptgene (GFP_{N_max}) is achieved when the normalized inputs equal to IDR (input dynamic range), then we can assume that the promoter activity is approximately 1. Therefore, the maximum fold change is given by:

$$GFP_{N\_max} = \frac{1}{B^m + \beta}$$

$$B^m = \frac{1-\beta}{GFP_{N\_max}} \approx \frac{1}{GFP_{N\_max}} \quad (4.14)$$

(a)

(b)

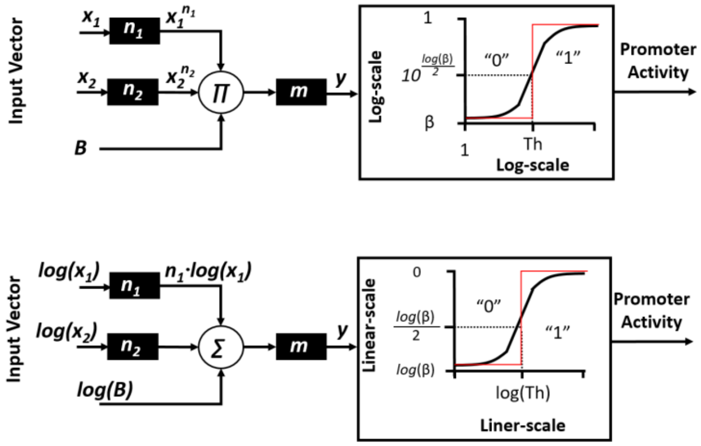

Supplementary Fig. 43. (a) An Abstract genetic model based on Eq. 6 in the main text. **(b)** After applying a logarithmic transform, we obtain a perceptron model including weights, bias, and activation function.

In this work, we used the fundamental properties of ANNs to create genetic circuits that encoded the calculations of smooth maximum, the smooth minimum, and the average of two analog inputs (Supplementary Notes, Section 3). In our implementation the cooperativity, represented by the Hill coefficient, acts as a weight ($\boldsymbol{m, n_i}$), and the node threshold is set by the basal level. Additionally, the bias is set by a linear function of the translation/transcription rates, the mRNA/protein half-life and cell growth rate divided by the binding affinities of protein-protein/protein-DNA reactions. Correspondingly, the logarithmic equivalent product of $\boldsymbol{m \cdot \sum_i n_i \cdot (X_i/IDR_i) + Bias > Threshold}$ sets the operation type shown in Supplementary Table 7 (*e.g.*, the maximum function uses a positive rectifier activation, the minimum function uses a negative rectifier function, and the average function uses a linear activation function; Supplementary Figs. 32 and 37). <u>To enable different operation types, we controlled the Hill coefficient of the P_{BAD} promoter (*m*) by adjusting the arabinose concentration (Supplementary Fig. 24).</u>

Supplementary Table 19. Parameters used in minimum, maximum, and average operations

System	[IPTG, aTc]	[aTc, AHL]	[AHL, IPTG]
n_1	0.3375	0.45	0.95
n_2	0.4375	0.55	0.85
IDR_1 at log scale	$\log(128) = 2.1$	$\log(64) = 1.8$	$\log(16) = 1.2$
IDR_2 at log scale	$\log(128) = 2.1$	$\log(32) = 1.5$	$\log(32) = 1.5$
m	2	1	1
Maximum Fold Change (MFC)	16	14	14
Bias at log scale (b)	$\log(1/16^{1/2})$ $= -0.6$	$\log(1/10)$ $= -1.1$	$\log(1/14)$ $= -1.1$
β (Basal level)	0.045	0.0001	0.0001
log(Th)	0.67	2	2
$\boldsymbol{B \cdot m - Th}$	-1.8	-3.15	-3.15
$n_1 \cdot m \cdot IDR_1$	1.42	0.81	1.14
$n_2 \cdot m_2 \cdot IDR_2$	1.84	0.82	1.27

$n_1 \cdot m \cdot IDR_1 + n_2 \cdot m_2 \cdot IDR_2 + B \cdot m - Th$	**1.38**	**-1.5**	**-0.7**
Operation type	Minimum	Maximum	Average

5. Design and model of multilayer perceptgene networks

In this section we present a biophysical model that describes the behavior of multilayer perceptgene network at steady state. We show that biophysical models can be described in a similar fashion to neural networks with three components: (1) weights that are represented by Hill-coefficients and cooperativity, (2) bias constants that are proportional to the ratio of the total synthesized proteins and promoter binding affinities and (3) activation functions that are represented by a promoter activity and are described by Michaelis-Menten kinetics.

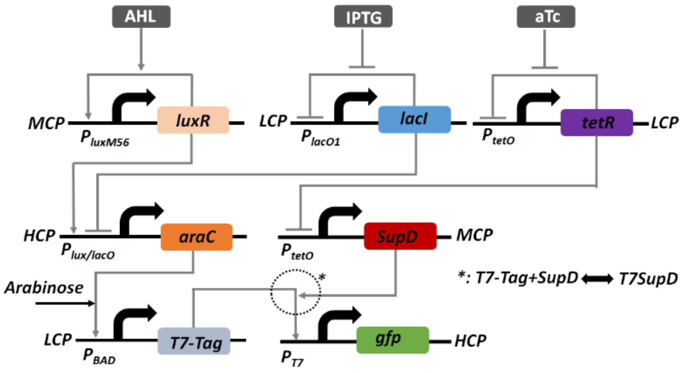

Supplementary Fig. 44. Multilayer perceptgene network accepts three analog inputs (AHL, IPTG and aTc).

In the first layer, the AraC protein is regulated by a circuit consists of a graded APF, an ANF loop and a combinatorial promoter ($P_{lux/lacO}$). The first layer circuit displays a power-law and multiplication function (Supplementary Figs. 48 and 49). The APF loop is induced by AHL and the ANF loop is induced by IPTG. A weak mutated P_{luxM56} promoter was used in the APF part to broaden the IDR of AHL (Supplementary Fig. 26). The activity of AraC protein upon inducers AHL and IPTG is described by:

$$y_1 = y_{m1} \cdot \left(\frac{AHL}{K_{m1}}\right)^{n_1} \cdot \left(\frac{IPTG}{K_{m2}}\right)^{n_2} \quad (5.1)$$

Where y_{m1} has units of concentration, and it depends on the binding affinity between transcription factors and the corresponding promoter, as well as the maximum level of transcription factor (Eq. 2.13). The experimental results of $P_{lux/lacO}$-based power-law and multiplication circuit are shown in Supplementary Figs. 48 and 49, which are well fitted using Eq. 5.1.

The AraC proteins are expressed as the output of the first layer. Subsequently, they interact with the P_{BAD} promoter, which further regulates the T7-RNA polymerase. The activity of the T7-RNA can be modeled in the following way

$$z_1 = z_{m1} \frac{(y_1/K_1)^{m_1} + \beta_1}{1 + \beta_1 + (y_1/K_1)^{m_1}} \quad (5.2)$$

$$K_1 = a \cdot {m_1}^{-b} \quad (5.3)$$

Where K_1 is the dissociation constant of Arabinose- AraC complex binding to P_{BAD} promoter, β_1 is the basal level of P_{BAD}, and m_1 is the effective Hill-coefficient. We have shown that K_1 and m_1 can be tunable by the Arabinose concentration level (Supplementary Fig. 22) with a power-law relation.

The amber suppressor tRNA *supD* is regulated by P_{tetO}-based ANF, and is given by:

$$y_2 = y_{m2} \cdot \left(\frac{aTc}{K_{m3}}\right)^{n_3} \quad (5.4)$$

where y_{m2} has units of concentration. The experimental results of P_{tetO}-based ANF are shown in Supplementary Fig. 50, which are fitted well to a power-law function using Eq. 5.1. The biochemical binding reaction between T7 RNA polymerase and $supD$ is given by[20]:

$$T7_{RNA} + supD \leftrightarrow T7_{RNA}SupD \quad (5.5.1)$$

$$T7_{RNA}SupD + supD \leftrightarrow T7_{RNA}SupD_2 \quad (5.5.2)$$

A simple solution to the set of Eq. 5.5.1 and Eq. 5.5.2 at the steady-state gives:

$$y_3 = y_{m3} \cdot \frac{(y_2/K_2)^{m_2} \cdot K_3 \cdot (z_1/K_3)^{m_3}}{K_4} \quad (5.5.3)$$

Here we assumed that there are two amber stop codons in the open reading frame, which should lead to $m_2 = 2$ [20]. However, as the first biochemical reaction in Eq. 5.5 ($T7_{RNA} + supD \leftrightarrow T7_{RNA}SupD$) can also bind to T7 promoter with a probability larger than zero, the effective value of m_2 can be reduced to less than 2. m_3 depends on the protein quaternary structure (the number of subunits that interact with each other and arrange themselves to form a final structure of the protein). Since the T7 RNA polymerase is a single subunit [20,21], $m_3 = 1$. K_2, K_3 and K_4 are the dissociation constants of biochemical reactions in Eq. 5.5. The T7 RNA polymerase was regulated by a ribosome binding sequence with a very low binding affinity (BBa_B0031) [2,22]. The binding of $T7_{RNA}SupD_2$ complex to T7 promoter, actives it and expresses GFP. This process demonstrates the output of the second perceptgene layer, and is given by:

$$z_2 = z_{m2} \frac{(y_3/K_5)^{m_4} + \beta_2}{1 + \beta_2 + (y_3/K_5)^{m_4}} \quad (5.6)$$

Where K_5 is the dissociation constant $T7_{RNA}SupD_2$ complex to P_{T7}, β_2 is the basal level of P_{T7}, and m_4 is the effective Hill-coefficient. Rewriting the set of Eq. 5.1-Eq. 5.6, gives:

$$y_1 = \left(B_1 \cdot \left(\frac{AHL}{K_{m1}}\right)^{n_1} \cdot \left(\frac{IPTG}{K_{m2}}\right)^{n_2}\right)^{m_1} \quad (5.7)$$

$$z_1 = \frac{y_1 + \beta_1}{1 + \beta_1 + y_1} \quad (5.8)$$

$$y_2 = \left(B_2 \cdot \left(\frac{x_3}{K_{m3}}\right)^{n_3}\right)^{m_2} \quad (5.9)$$

$$y_3 = (B_4 \cdot y_2 \cdot (B_3 \cdot z_1)^{m_3})^{m_4} \quad (5.10)$$

$$z_2 = \frac{y_3 + \beta_2}{1 + \beta_2 + y_3} \quad (5.11)$$

Where: $B_4 \equiv \frac{y_{m3}}{K_5} \cdot \frac{K_3}{K_4}$, $B_3 \equiv \frac{z_{m1}}{K_3}$, $B_2 \equiv \frac{y_{m2}}{K_2}$, $B_1 \equiv \frac{y_{m1}}{a} \cdot {m_1}^b$

An abstract model of the set of Eq. 5.7-Eq. 5.11 is shown in Supplementary Fig. 45, and is built from three computational components:

1. (1) Network Weights (n_i and m_i): are represented by effective Hill-coefficients, which depend on biological cooperativities of protein interactions and protein quaternary structure (the number of subunits that interact with each other and arrange themselves to form a final protein).
2. (2) Bias constants (B_i): are represented by translation/transcription rates, mRNA/protein half-lives, rates of cell growth, binding affinities in protein-protein or protein-DNA interactions. Bias constant is unit-less.
3. (3) Activation functions or network nodes (z_i- the output of each perceptgene) which are represented by a promoter activity and is given by a normalized Michaelis-Menten model $y_i/(1 + y_i)$.

As in Supplementary Fig. 45, each input is normalized and fed into a power-law function with an exponent n_i. Then the product is multiplied with a bias constant, giving rise to an analog signal (y_i). Applying a logarithmic operation to the signal, we get: $log(y_i) = m_l \cdot log(B_i) + m_l \cdot \sum_j n_j \cdot log(I_{ni})$ (I_{ni} is the normalized input). The bias acts as a reference level.

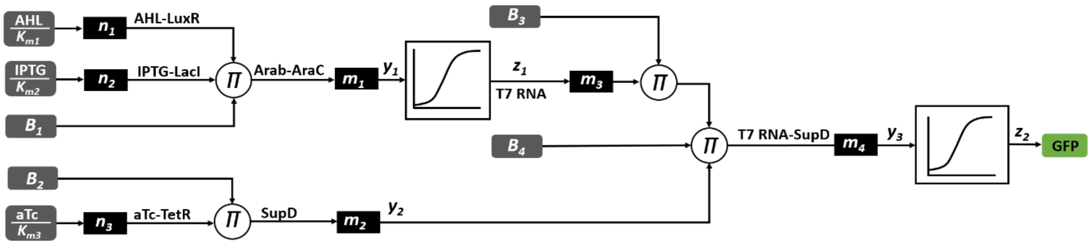

Supplementary Fig. 45. Abstract model of Multilayer perceptgene in analogy to abstract models of artificial neural networks. Comparing to Fig. 3b, the only difference is that B_3 is included in B_4.

5.1. Design of 3-input majority function

The 3-input **majority function** (also called the **median operator**) describes a logic function from three inputs to one output. The output is a high- "1" if and only if the majority of the inputs are high- "1". Otherwise, the output is a low- "0". The majority function can be found in various applications such as adders and subtractors [23]. The truth table and the implementation of the 3-input majority function using a logic gate design are shown in Supplementary Fig. 46a and B. The early works with artificial neural networks were based on a linear threshold unit (LTU) and were targeted to serve as a computational model that can implement any Boolean logic function [24]. The implementation of a 3-input majority function (Supplementary Fig. 44) using a two-layer perceptgene network is based on using principles of artificial neural networks (Supplementary Fig. 45). First, we solved the set of Eq. 5.7-5.11, and then converted them to the linear domain using a logarithmic transformation. Subsequently, an activation function was applied to the transformed output. For simplicity, we approximated the activation function of the last layer as a step function in the log-scale:

$$z_2 = \begin{cases} 10 & y_3 \geq 1 \\ 1 & y_3 < 1 \end{cases} \quad (5.12)$$

And the activation function $[(y_1 + \beta_1)/(1 + y_1 + \beta_1)]$ in the hidden layer was approximated (e.g. Supplementary Fig. 46c) as:

$$z_1 = \begin{cases} 1 & y_1 \geq \alpha \\ \beta_1 < z_1 < 1 & 1/\alpha \leq y_1 < \alpha \\ \beta_1 & y_1 < 1/\alpha \end{cases} \quad (5.13)$$

- We assumed that the normalized input (x_i) is between 1 and 10
- We define:

  $x_1 \equiv \frac{AHL}{K_{m1}}$, $x_2 \equiv \frac{IPTG}{K_{m2}}$, $x_3 \equiv \frac{aTc}{K_{m3}}$

  $A_1 \equiv log\left(\frac{y_{m1}}{a}\right)$, $\gamma \equiv \log(\alpha)$, $b_1 \equiv \log(\beta_1)$

  $A_5 \equiv \log(B_4) + m_2 \cdot \log(B_2) + m_3 \cdot \log(B_3)$

  $B_5 \equiv 10^{A_5} = B_4 \cdot {B_2}^{m_2} \cdot {B_3}^{m_3}$

  $B_1 \equiv 10^{A_1} \cdot {m_1}^{b}$

A simplified model of the set of equations shows that the network consists of two-layers perceptgene, as shown in Supplementary Fig. 46d.

State 0: $x_1 = 1, x_2 = 1, x_3 = 1, z_2 = 1;$

We require that $y_1 = {B_1}^{m_1} < 1/\alpha$

➔ $m_1 \cdot \log(B_1) < -\log(\alpha)$

➔ $m_1 \cdot \left(\log\left(\frac{y_{m1}}{a}\right) + b \cdot \log(m_1)\right) < -\log(\alpha)$

⇨ $m_1 \cdot (A_1 + b \cdot \log(m_1)) < -\gamma$ (5.14.1)

We require that $z_1 = \beta_1$

$y_2 = {B_2}^{m_2}$

$y_3 = (B_4 \cdot {B_2}^{m_2} \cdot (B_3 \cdot \beta_1)^{m_3})^{m_4} < 1$

➔ $m_4 \cdot (\log(B_4) + m_2 \cdot \log(B_2) + m_3 \cdot \log(B_3) + m_3 \log(\beta_1)) < 0$

⇨ $m_4 \cdot (A_5 + m_3 \cdot b_1) < 0$ (5.14.2)

<u>State 1: $x_1 = 1, x_2 = 1, x_3 = 10,\ z_2 = 1;$</u>

We require that $y_1 = {B_1}^{m_1} < 1/\alpha$

⇨ $m_1 \cdot (A_1 + b \cdot \log(m_1)) < -\gamma$

We require that $z_1 = \beta_1$

$y_2 = {B_2}^{m_2} \cdot 10^{n_3 m_2}$

$y_3 = (B_4 \cdot {B_2}^{m_2} \cdot 10^{n_3 m_2} \cdot (B_3 \cdot z_1)^{m_3})^{m_4} < 1$

➔ $m_4 \cdot (\log(B_4) + m_2 \cdot \log(B_2) + m_3 \cdot \log(B_3) + m_2 \cdot n_3 + m_3 \log(\beta_1)) < 0$

⇨ $m_4 \cdot (A_5 + m_2 \cdot n_3 + m_3 \cdot b_1) < 0$ (5.14.3)

<u>State 2: $x_1 = 1, x_2 = 10, x_3 = 1,\ z_2 = 1;$</u>

We require that $1/\alpha<y_1<\alpha$:

$y_1 = (B_1 \cdot (10)^{n_2})^{m_1}$

è $1/\alpha < (B_1 \cdot (10)^{n_2})^{m_1} < \alpha$

➔ $-\log(\alpha) < m_1 \cdot (\log(B_1) + n_2) < \log(\alpha)$

⇨ $-\gamma < m_1 \cdot (A_1 + b \cdot \log(m_1) + n_2) < \gamma$ (5.14.4)

We require that: $\beta_1 < z_1 < 1$

$y_2 = {B_2}^{m_2}$

$y_3 = (B_4 \cdot {B_2}^{m_2} \cdot (B_3 \cdot z_1)^{m_3})^{m_4} < 1$

➔$m_4 \cdot (\log(B_4) + m_2 \cdot \log(B_2) + m_3 \cdot \log(B_3) + m_3 \log(z_1)) < 0$

⇨ $m_4 \cdot (A_5 + m_3 \log(z_1)) < 0$ (5.14.5)

<u>State 3: $x_1 = 1, x_2 = 10, x_3 = 10,\ z_2 = 10;$</u>

We require that $1/\alpha<y_1<\alpha$:

$y_1 = (B_1 \cdot (10)^{n_2})^{m_1}$

$-\gamma < m_1 \cdot (A_1 + b \cdot \log(m_1) + n_2) < \gamma$

We require that: $\beta_1 < z_1 < 1$

$y_2 = {B_2}^{m_2} \cdot 10^{n_3 m_2}$

$y_3 = (B_4 \cdot {B_2}^{m_2} \cdot 10^{n_3 m_2} \cdot (B_3 \cdot z_1)^{m_3})^{m_4} \geq 1$

➔ $m_4 \cdot (\log(B_4) + m_2 \cdot \log(B_2) + m_3 \cdot \log(B_3) + m_2 \cdot n_3 + m_3 \log(z_1)) \geq 0$

⇨ $m_4 \cdot (A_5 + m_2 \cdot n_3 + m_3 \log(z_1)) \geq 0$ (5.14.6)

<u>State 4: $x_1 = 10, x_2 = 1, x_3 = 1,\ z_2 = 1;$</u>

We require that $1/\alpha<y_1<\alpha$:

$y_1 = (B_1 \cdot (10)^{n_1})^{m_1}$

➔ $1/\alpha < (B_1 \cdot (10)^{n_1})^{m_1} < \alpha$

➔ $-\log(\alpha) < m_1 \cdot (\log(B_1) + n_1) < \log(\alpha)$

⇨ $-\gamma < m_1 \cdot (A_1 + b \cdot \log(m_1) + n_1) < \gamma$ (5.14.7)

We require that: $\beta_1 < z_1 < 1$

$y_2 = {B_2}^{m_2}$

$y_3 = (B_4 \cdot {B_2}^{m_2} \cdot (B_3 \cdot z_1)^{m_3})^{m_4} < 1$

⇨ $m_4 \cdot (A_5 + m_3 \log(z_1)) < 0$

<u>State 5: $x_1 = 10, x_2 = 1, x_3 = 10,\ z_2 = 10;$</u>

We require that $1/\alpha<y_1<\alpha$:

$y_1 = (B_1 \cdot (10)^{n_1})^{m_1}$

⇨ $-\gamma < m_1 \cdot (A_1 + b \cdot \log(m_1) + n_1) < \gamma$

We require that: $\beta_1 < z_1 < 1$

$y_2 = {B_2}^{m_2} \cdot 10^{n_3 m_2}$

$y_3 = (B_4 \cdot {B_2}^{m_2} \cdot 10^{n_3 m_2} \cdot (B_3 \cdot z_1)^{m_3})^{m_4} \geq 1$

➔ $m_4 \cdot (\log(B_4) + m_2 \cdot \log(B_2) + m_3 \cdot \log(B_3) + m_2 \cdot n_3 + m_3 \log(z_1)) \geq 0$

⇨ $m_4 \cdot (A_5 + m_2 \cdot n_3 + m_3 \log(z_1)) \geq 0$ (5.14.8)

<u>State 6: $x_1 = 10, x_2 = 10, x_3 = 1,\ z_2 = 10;$</u>

We require that $y_1 > \alpha$

➔ $y_1 = (B_1 \cdot (10)^{n_1} \cdot (10)^{n_2})^{m_1} > \alpha$

➔ $m_1 \cdot (\log(B_1) + n_1 + n_2) > \log(\alpha)$

⇨ $m_1 \cdot (A_1 + b \cdot \log(m_1) + n_1 + n_2) > \gamma$ (5.14.9)

We require that $z_1 = 1$

$y_2 = {B_2}^{m_2}$

$y_3 = (B_4 \cdot {B_2}^{m_2} \cdot (B_3)^{m_3})^{m_4} \geq 1$

➔ $m_4 \cdot (\log(B_4) + m_2 \cdot \log(B_2) + m_3 \cdot \log(B_3)) \geq 0$

⇨ $m_4 \cdot (A_5) \geq 0$ (5.14.10)

<u>State 7: $x_1 = 10, x_2 = 10, x_3 = 10,\ z_2 = 10;$</u>

We require that $y_1 > \alpha$

$y_1 = (B_1 \cdot (10)^{n_1} \cdot (10)^{n_2})^{m_1} \geq \alpha$

⇨ $m_1 \cdot (A_1 + b \cdot \log(m_1) + n_1 + n_2) > \gamma$

We require that $z_1 = 1$

$y_2 = {B_2}^{m_2} \cdot 10^{n_3 m_2}$

$y_3 = (B_4 \cdot {B_2}^{m_2} \cdot 10^{n_3 m_2} \cdot (B_3)^{m_3})^{m_4} \geq 1$

➔ $m_4 \cdot (\log(B_4) + m_2 \cdot \log(B_2) + m_3 \cdot \log(B_3) + m_2 \cdot n_3) \geq 0$

⇨ $m_4 \cdot (A_5 + m_2 \cdot n_3) \geq 0$ (5.14.11)

<u>Summary:</u> The design conditions of A_1 are set by:

I. $A_1 < -\frac{\gamma}{m_1} - b \cdot \log(m_1)$

II. $-\frac{\gamma}{m_1} - b \cdot \log(m_1) - n_2 < A_1 < \frac{\gamma}{m_1} - b \cdot \log(m_1) - n_2$

III. $A_1 > \frac{\gamma}{m_1} - b \cdot \log(m_1) - n_1 - n_2$

Therefore, $A_{1min} < A_1 < A_{1max}$ (5.15.1)

where:

$$A_{1max} = min\left\{-\frac{\gamma}{m_1} - b \cdot \log(m_1), \frac{\gamma}{m_1} - b \cdot \log(m_1) - \max(n_1, n_2)\right\} \quad (5.15.2)$$

$$A_{1min} = max\left\{\frac{\gamma}{m_1} - b \cdot \log(m_1) - n_1 - n_2, -\frac{\gamma}{m_1} - b \cdot \log(m_1) - \min(n_1, n_2)\right\} \quad (5.15.3)$$

We require that $A_{min} < A_{max}$. To gain deeper insights into the effects of A_1 on the behavior of the network, we consider two asymptotic cases:

1. $\gamma \ll 0$; a step function is used as the activation function in the hidden layer

   $A_{1max} = -b \cdot \log(m_1) - \max(n_1, n_2)$

   $A_{1min} = -b \cdot \log(m_1) - \min(n_1, n_2)$ (5.16.1)

   $A_{1max} < A_{1min}$, unachievable condition. Therefore, we cannot implement a majority function using two perceptgene layers with a step function in the hidden layer

2. $\gamma \gg 0$, an analog function is used as the activation function in the hidden layer

   $A_{1max} = -\frac{\gamma}{m_1} - b \cdot \log(m_1)$

   $A_{1min} = \frac{\gamma}{m_1} - b \cdot \log(m_1) - n_1 - n_2$

   $A_{1max} > A_{1min}$, which is achievable when:

   $\frac{\gamma}{m_1} < \frac{n_1 + n_2}{2}$ (5.16.2)

We now continue with the first condition in Eq. 5.15.1, we assume that:

$-\frac{\gamma}{m_1} - b \cdot \log(m_1) < \frac{\gamma}{m_1} - b \cdot \log(m_1) - \max(n_1, n_2)$

⇨ $2 \cdot \gamma > \max(n_1, n_2) \cdot m_1$ (5.17.1)

Under these conditions, we get:

$$\rightarrow \boldsymbol{A_{1max}} = -\frac{\gamma}{m_1} - b \cdot \log(m_1) \quad (5.17.2)$$

According to the second condition in Eq. 5.15.2, we should require:

$$\frac{\gamma}{m_1} - b \cdot \log(m_1) - n_1 - n_2 > -\frac{\gamma}{m_1} - b \cdot \log(m_1) - \min(n_1, n_2)$$

$$\Rightarrow \quad 2 \cdot \gamma > (n_1 + n_2 - \min(n_1, n_2)) \ \cdot m_1 \quad (5.17.3)$$

The conditions in Eq. 5.18.1 and Eq. 5.18.3 are similar

Under these conditions we get:

$$\rightarrow \boldsymbol{A_{1min}} = \frac{\gamma}{m_1} - b \cdot \log(m_1) - n_1 - n_2 \quad (5.17.4)$$

As a summary, we get:

$$\frac{\gamma}{m_1} - b \cdot \log(m_1) - n_1 - n_2 < A_1 < -\frac{\gamma}{m_1} - b \cdot \log(m_1) \quad (5.18)$$

Summary; The design conditions of A_5 are set by:

I. $A_5 < -m_3 \cdot b_1$
II. $A_5 < -m_2 \cdot n_3 - m_3 \cdot b_1$
III. $A_5 < -m_3 \log(z_1)$
IV. $A_5 \geq 0$
V. $A_5 \geq -m_2 \cdot n_3$
VI. $A_5 \geq -m_2 \cdot n_3 - \ m_3 \log(z_1)$

Therefore $A_{5min} < A_5 < A_{5max}$ (5.19.1)

where

$$A_{5max} = min\{-b_1, -m_2 \cdot n_3 - b_1, -m_3 \log(z_1)\ \}$$

$$A_{5min} = max\{0, -m_2 \cdot n_3, -m_2 \cdot n_3 - \ m_3 \log(z_1)\ \}$$

The Basal level of promoter often is $\beta_1 < 1$, and therefore $b_1 < 0$, and $\log(z_1) \leq 0$, therefor:

$$0 < A_5 < -m_2 \cdot n_3 - b_1 \quad (5.19.2)$$

The simulation results as in Supplementary Fig. 47a show that for $m_1 = 2,$ a 3-input majority function can be implemented by a two-layer perceptgene network. Parameters used: $b = 0.73, \alpha = 10, n_1 = 0.5, n_2 = 0.75, n_3 = 0.7, m_4 = 1, m_2 = 3, m_3 = 1$ (T7 RNA polymerase is a single subunit), $B_1 = 0.05(0.006 < B_1 < 0.2$, margin of two orders), $B_5 = 1.25,\ \beta_1 = 0.001, \beta_2 = 0.002$. We used a Michaelis-Menten model to model the activity of z_1. The simulation results also show that for low m_1 (e.g. $m_1 = 1$), it is challenging to implement a 3-input majority function using a two-layer peceptgene network, because the design parameters do not satisfy the conditions in Eq. 5.18-5.19 ($B_1 = 0.05, 0.03 < \ \beta_1 < 0.1$, with a very small margin).

Compared with a one-layer network, a two-layer perceptgene network design has the following advantages:

- **Use less number of parts compared to digital design. In our design we used 7 proteins and 8 promoters, total of 15 parts to implement a 3-input majority function in *E. coli*. In digital design, the same circuit has been implemented in *E. coli* using 10 proteins and 12 promoters (total of 22 parts) [4].**
- **Sigmoid functions can have benefits. For example, the state [1,1,0], which displays a "1" logic state in the output, requires that the hidden layer acts as AND logic gate with a very low value of B_1. Simultaneously, the state [1,0,1], which displays a "1" logic state in the output, requires that the hidden layer acts as OR logic gate with a high value of B_1. By contrast, sigmoid functions can solve such conditions very smoothly.**

(a)

x_1	x_2	x_3	z
0	0	0	0
0	0	1	0
0	1	0	0
0	1	1	1
1	0	0	0
1	0	1	1
1	1	0	1
1	1	1	1

(b)

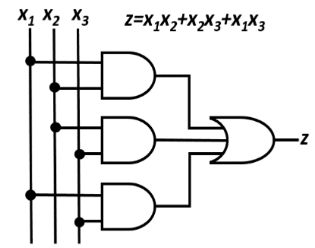

(c)

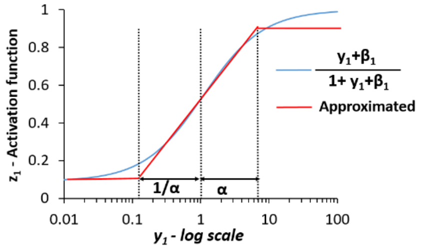

(d)

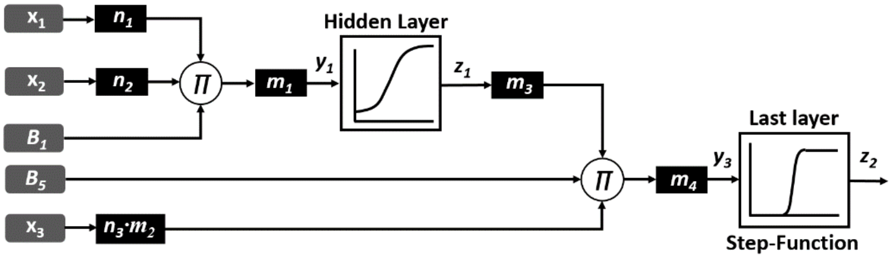

(e)

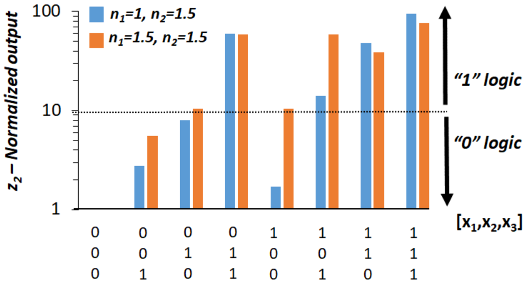

For X_i=[1,16], n_3=0.7, m_1=1.75, m_2=1.5, m_3=1, m_4=1, B_1=4.5×10^{-3}, B_5=1, β_1=0.001

Supplementary Fig. 46. (a) Truth table of 3-input majority function. **(b)** The implementation of 3-input majority function by digital design. **(c)** The approximation of Michaelis-Menten model by a linear function in the log-scale. **(d)** A simplified abstract model based on two-layer perceptgene to implement a 3-input majority function. **(e)** Simulation results for majority function based on Supplementary Fig. 46d. Experimental and simulation results. The horizontal dashed line, determined by the half of the fold change at the logarithmic scale, separates between the "0" and "1". The error function for the asymmetric weights is 12% and for the symmetric weights is 11%. The other parameters are shown at right side. We used a quadric Error function at the log-domain: $E = (\log(Z_2) - \log(Z_D))^2/2$. Z_D is the expected data and equal to $Z_{DL} = 1$, and $Z_{DH} = 100$.

(a)

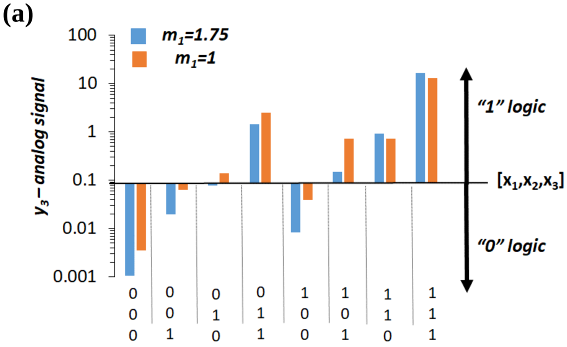

(b)

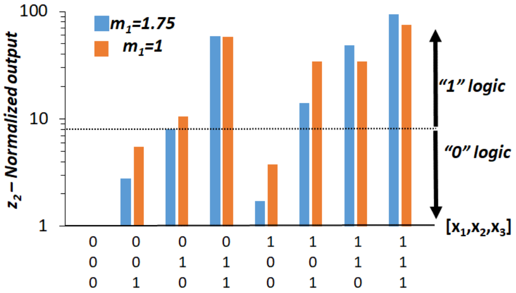

Supplementary Fig. 47. (a) Simulation results for the analog output (y_3) of 3-input majority function based on the two-layer perceptgene network. The threshold was calculated as $10^{-\log(\beta_2)/2}$. **(b)** Simulation results for the output (z_2) of 3-input majority function based on perceptgene network. The simulation were performed with X_i =[1-16]. Experimental and simulation results. The horizontal dashed line, determined by the half of the fold change at the logarithmic scale, separates between the "0" and "1".

5.2. Experimental results of 3-input majority circuit

First, we showed the experimental results of P_{luxM56}-based APF, P_{lacO1}-based ANF loops and $P_{lux/lacO}$-based combinatorial promoter circuit and fitted the data to power-law and multiplication function: by

$(\log(GFP) = c + n_9 \cdot \log(AHL) + n_{10} \cdot \log(IPTG))$. This circuit (Supplementary Fig. 48) is used as the majority function's first layer (Supplementary Fig. 44).

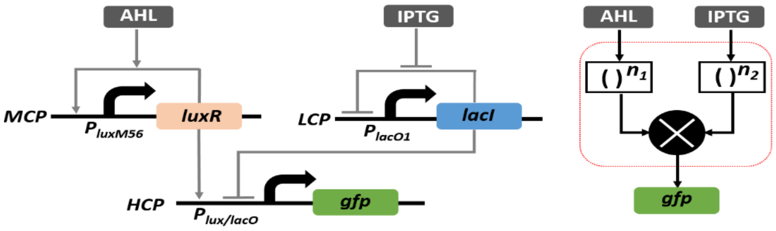

Supplementary Fig. 48. P_{luxM56}-based APF, P_{lacO1}-based ANF loops and $P_{lux/lacO}$-based combinatorial promoter circuit.

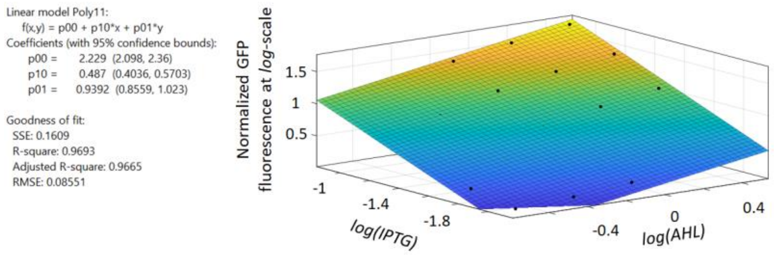

Supplementary Fig. 49. Matlab surface fits the experimental results of APF (P_{luxM56}) and ANF (P_{lacO1}) loops and combinatorial promoter ($P_{lux/lacO}$-GFP) to power-law and multiplication function.

Second, we showed the experimental results of P_{tetO}-based ANF loop and fitted the data to power-law function $(\log(GFP) = c + n_{11} \cdot \log(aTc))$. This circuit (Supplementary Fig. 50) is used to regulate the third inputs aTc of the majority function.

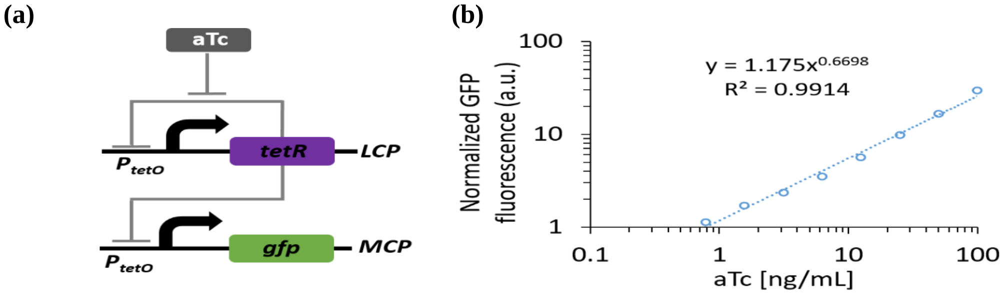

Supplementary Fig. 50. **(a)** Implementation of P_{tetO}-based ANF loop circuit. **(b)** Experimental results of P_{tetO}-based ANF loop circuit that fits of a power-law function.

Each input (e.g., inducer-transcription factor) has its input dynamic range (IDR). However, occasionally when multiple inputs are aggregated at the same computational node, transcription factor binding interference can effectively reduce the dynamic range for neuromorphic computation. Since this disturbance affects the final computation, we characterize every synthetic part separately and combine it with other inputs. For example, we characterized TetR alone and with hybrid promoters TetR/LacI and TetR/LuxR. The IDR for TetR was

observed to be 2.1 orders of magnitude (Supplementary Fig. 50), remained the same for TetR/LacI (Supplementary Figs. 13 and 18), and was reduced to 1.8 orders of magnitude for TetR/LuxR (Supplementary Fig. 28).

The 3-input majority function accepts AHL [0.1875-0.3 μM], IPTG [7.8125-125 μM] and aTc [1.5625-25 ng/mL]. The three inputs have a dynamic input range from 1 to 16. The simulation results of the two-layer perceptgene network are shown in Supplementary Fig. 47b. We used a Michaelis-Menten model as an activation function to calculate the activities of z_1 (the hidden layer) and z_2 (the final layer). We used a consistent set of model parameters as in Supplementary Figs. 47a and 47b except that B_1 is changed to 0.0025 and B_5 is changed to 1. We normalized each measurement by the minimum activity of [0,0,0] state. The experimental results of the 3-input majority circuit (Supplementary Figs. 44 and 51). To keep B_1 very low, we located AraC on a low-copy-number plasmid and added an ssrA degradation tag [13] (LAA) to AraC. To keep B_5 very low, a ribosome binding sequence with a low binding affinity (BBa_B0031[22]) was used to regulate the T7 RNA polymerase. A low Arabinose concentration was set as 0.03125 mM, and a high Arabinose was set as 0.25 mM. Based on biochemical reactions described in Eq. 5.7-Eq. 5.11, our model could capture well the experimental results.

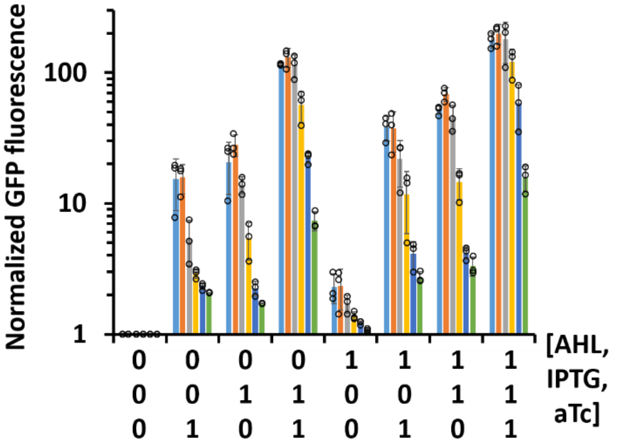

Supplementary Fig. 51. Experimental results of majority circuit for various Arabinose concentrations (0.250, 0.125, 0.062, 0.031, 0.015, 0.007 mM). Colorful bars and their error bars show the average and standard deviation from independent replicates ($n = 3$). Median values of individual replicates are marked in circles.

Hard Majority function

To improve the accuracy of the majority function with a response of two-discrete binary states, we can connect the output of the majority function to a steep input-output transfer function. One possibility is using unidirectional recombinase (e.g., Bxb1 or phiC31) as a final layer (Supplementary Fig. 52). Unidirectional recombinase-based circuits have been used to implement logic functions with distinct low and high digital outputs [25,26]. We can use PhiC31 unidirectional serine recombinase, which targets its own cognate pair of nonidentical recognition sites known as attB and attP. PhiC31 can irreversibly invert or excise DNA based on the orientation of the surrounding pair of recognition sites. The new inverted recognition sites are known as attL and attR. A constitutive promoter proD regulates the expression level of the inverted green fluorescent protein gene (*gfp*), which is located between the two recognition sites attB and attP. In the absence of PhiC31 protein, the proD regulates the inverted *gfp* gene, giving a background GFP signal (very low GFP). Whereas, in the presence of PhiC31 protein inside the cell, the PhiC31-attB and PhiC31-attP complexes flipped the inverted *gfp* gene to be in the same orientation as the proD promoter, resulting in a high GFP signal. Ribozyme-based insulator part (RiboJ) can be added to the 5' UTR of the *gfp* gene to reduce the background signal. Using this construct, we expect that the recombinase level for the input logic states above a certain threshold can yield the same high signal, and the recombinase level for the input logic states below the threshold can yield the same low signal.

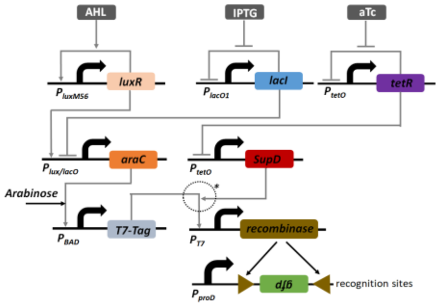

Supplementary Fig. 52: Hard classification: a suggestion to implement a three-input majority function for hard classification based on using the three-input soft majority from Supplementary Fig. 44.

Supplementary Table 20: Truth table of the linear-domain perceptron-based 3-input majority function and evaluation of constraints on the design parameters. B_1 and B_1 are the biases of the first layer and second layer perceptgenes, respectively. The three input weights are n_1, n_2, and n_3, while m is the weight of the first layer perceptegene output (Z_1) that serves as an input to the second layer perceptgene. $\gamma_{L1,}$ γ_{L2} and γ_{H1} and γ_{H2} are the low and high thresholds of the piecewise-linear first and the second activation functions f_{A1} and f_{A2}. The γ_{H2}- γ_{L2}, and γ_{H1}- γ_{L1} are defined as the input dynamic ranges of the activation functions.

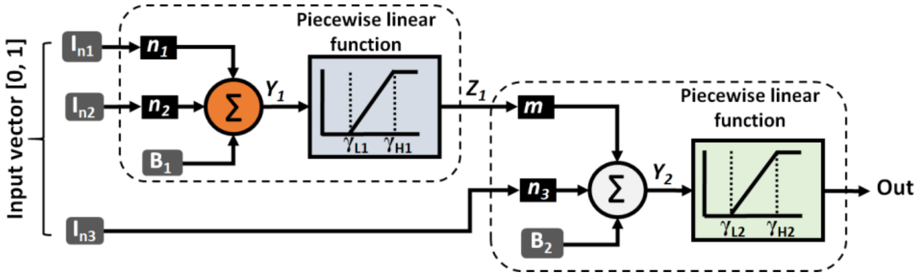

I_{n1} I_{n2} I_{n3}	Out	Y_1	Z_1	Y_2	Constraints on Design parameters
0 0 0	0	Design constraints subsumed by 001 case			
0 0 1	0	B_1	0	B_2+n_3	$B_1 < \gamma_{L1}$<br>$B_2+n_3 < \gamma_{L2}$
0 1 0	0	B_1+n_2	$0 \leq f_{A1}(B_1+n_2)<1$	$B_2+m\times f_{A1}(B_1+n_2)$	$B_2+m\times f_{A1}(B_1+n_2) < \gamma_{L2}$
0 1 1	1	B_1+n_2	$0<f_{A1}(B_1+n_2)\leq 1$	$B_2+n_3+m\times f_{A1}(B_1+n_2)$	$B_1+n_2 > \gamma_{L1}$<br>$B_2+n_3+m\times f_{A1}(B_1+n_2) > \gamma_{H2}$
1 0 0	0	B_1+n_1	$0 \leq f_{A1}(B_1+n_1)<1$	$B_2+m\times f_{A1}(B_1+n_1)$	$B_2+m\times f_{A1}(B_1+n_1)< \gamma_{L2}$
1 0 1	1	B_1+n_1	$0<f_{A1}(B_1+n_1) \leq 1$	$B_2+n_3+m\times f_{A1}(B_1+n_1)$	$B_1+n_1 > \gamma_{L1}$<br>$B_2+n_3+m\times f_{A1}(B_1+n_1) > \gamma_{H2}$
1 1 0	1	$B_1+n_1+n_2$	1	B_2+m	$B_1+n_1+n_2 > \gamma_{H1}$<br>$B_2+m > \gamma_{22}$
1 1 1	1	Design constraints subsumed by 110, 101, 011 cases			

Majority analysis for the first activation function:

To satisfy state [001], we require $B_1 < \gamma_{L1}$

To satisfy state [011], we require $B_1+n_2 > \gamma_{L1} \Rightarrow B_1 > \gamma_{L1}- n_2$

To satisfy state [101], we require $B_1+n_1 > \gamma_{L1} \Rightarrow B_1 > \gamma_{L1}- n_1$

The last three conditions yield to $\gamma_{L1}\text{-min}(n_1,n_2)<B_1 < \gamma_{L1}$

To satisfy state [110], we require $B_1+n_1+n_2 > \gamma_{H1} \Rightarrow B_1 > \gamma_{H1}- n_1\text{-}n_2$

The last and first conditions yield $\gamma_{H1}\text{-}n_1\text{-}n_2<B_1 < \gamma_{L1}$

Thus, the input dynamic range of the first activation function should be $\gamma_{H1}\text{-}\gamma_{L1}<n_1+n_2$

Majority analysis for the second activation function:

To satisfy state [110], we require $B_2+m > \gamma_{H2} \Rightarrow B_2 > \gamma_{H2}\text{-}m$

To satisfy state [011], we require $B_2+n_3+m\times f_{A1}(B_1+n_2) > \gamma_{H2} \Rightarrow B_2 > \gamma_{H2}\text{-}n_3\text{-}m\times f_{A1}(B_1+n_2)$

To satisfy state [101], we require $B_2+n_3+m\times f_{A1}(B_1+n_1) > \gamma_{H2} \Rightarrow B_2 > \gamma_{H2}\text{-}n_3\text{-}m\times f_{A1}(B_1+n_1)$

The last three conditions yield to $B_2 > \gamma_{H2}\text{-min}(m,n_3+ m\times f_{A1}(B_1+n_2), n_3+ m\times f_{A1}(B_1+n_1))$

To satisfy state [001], we require $B_2+n_3 < \gamma_{L2} \Rightarrow B_2 < \gamma_{L2}- n_3$

To satisfy state [010], we require $B_2+m\times f_{A1}(B_1+n_2) < \gamma_{12} \Rightarrow B_2< \gamma_{12}\text{-}m\times f_{A1}(B_1+n_2)$

To satisfy state [101], we require $B_2+m\times f_{A1}(B_1+n_1) < \gamma_{12} \Rightarrow B_2< \gamma_{12}\text{-}m\times f_{A1}(B_1+n_1)$

The last three conditions yield to:

$$\gamma_{H2}\text{- min}(m,n_3+ m\times f_{A1}(B_1+n_2), n_3+ m\times f_{A1}(B_1+n_1))<B_2< \gamma_{L2}\text{-max}(n_3, m\times f_{A1}(B_1+n_2), m\times f_{A1}(B_1+n_1))$$

Thus, the input dynamic range of the second activation function should be

$$\gamma_{H2}- \gamma_{L2} < \text{min}(m,n_3+ m\times f_{A1}(B_1+n_2), n_3+ m\times f_{A1}(B_1+n_1))\text{-max}(n_3, m\times f_{A1}(B_1+n_2), m\times f_{A1}(B_1+n_1))$$

The analysis of the last condition yields that: $m>n_3$

There are three cases: (for simplicity, we assumed that $n_1<n_2$):

1. When $m<n_3$, the last equation yields that $\gamma_{H2}- \gamma_{L2} <m\text{-}n_3<0$
2. When $m=n_3$, the last equation yields that $\gamma_{H2}- \gamma_{L2} <0$
3. When $m>n_3$, the last equation yields that $\gamma_{H2}- \gamma_{L2} <m\text{-}n_3$, or $\gamma_{H2}- \gamma_{L2} < m\times(1\text{-}f_{A1}(B_1+n_2))$

The condition of [001] and [010] yields for $n_3>\gamma_{H2}- \gamma_{L2}$; thus we obtain $m>n_3>\gamma_{H2}\text{-}\gamma_{L2}$.

Supplementary Table 21: Truth table of perceptgene-based majority function and evaluation of the design parameters. D.C. = Don't care.

$m = m_3 \times m_4 = 1 \times 1 = 1$

$n'_1 = n_1 \times m_1 = 0.5 \times 2 = 1$

$n'_2 = n_2 \times m_1 = 1 \times 2 = 2$

$n'_3 = n_3 \times m_2 \times m_4 = 0.7 \times 1.75 \times 1 = 1.25$

$B_1 = 4.5 \times 10^{-3}, B_2 = 1, \gamma_1 = -1, \gamma_2 = 1$

I_{n1} I_{n2} I_{n3}	Out	Y_1	Z_1	Y_2	Constraints on Design parameters
log(1)<br>log(1)<br>log(1)	"1"	B_1<br>$\log(4.5\times10^{-3})=-2.3$	D.C.	$B_2+m\times Z_1$<br>$\log(1)+1\times\log(Z_1)$	$B_2+m\times Z_1 < \gamma_1$<br>$\log(1)+1\times\log(Z_1)< -1$<br>$\log(Z_1)<-1$<br>$Z_1<0.79$
log(1)<br>log(1)<br>log(10)	"0"	B_1<br>$\log(4.5\times10^{-3})=-2.3$	0	B_2+n_3'<br>$\log(1)+1.25$	(1) $B_1 < \gamma_1$<br>$\log(4.5\times10^{-3})<1$<br>(2) $B_2+n_3'+m\times\log(Z_1) < \gamma_1$<br>$\log(1)+1.25+1\times\log(Z_1) < -1$<br>$\log(Z_1)<-2.25$<br>$Z_1<0.005$

log(1)<br>log(10)<br>log(1)	"0"	B_1+n_2'<br>log(4.5×10^{-3})+2<br>-2.3+2=-0.3	D.C	B_2+m×Z_1<br>log(1)+1×log(Z_1)	B_2+m×Z_1 < γ_1<br>log(1)+1×log(Z_1)< -1<br>log(Z_1)<-1<br>Z_1<0.79
log(1)<br>log(10)<br>log(10)	"1"	B_1+n_2'<br>log(4.5×10^{-3})+2<br>-2.3+2=-0.3	Intermed	B_2+n_3'+m<br>log(1)+1.25+1	(1) B_1+n_2' > γ_1<br>1og(4.5×10^{-3})+2 > -1<br>(2) B_2+n_3'+m×log(Z_1) > γ_2<br>1og(1)+1.25+ log(Z_1) >1<br>Z_1>0.56
log(10)<br>log(1)<br>log(1)	0	B_1+n_2'<br>log(4.5×10^{-3})+2<br>-2.3+2=-0.3	D.C.	B_2+m×Z_1<br>log(1)+1×log(Z_1)	B_2+m×Z_1< γ_1<br>log(1)+1×log(Z_1)< -1<br>log(Z_1)<-1<br>Z_1<0.79
log(10)<br>log(1)<br>log(10)	"1"	B_1+n_1'<br>log(4.5×10^{-3})+1<br>-2.3+1=-1.3	Intermed	B_2+n_3'+m<br>log(1)+1.25+1	(1) B_1+n_1' > γ_1<br>1og(0.004)+1 >-1<br>(2) B_2+n_3'+m×log(Z_1) > γ_2<br>1og(1)+1.25+log(Z_1)>1<br>Z_1>0.56
log(10)<br>log(10)<br>log(1)	"1"	B_1+n_1'+n_2'<br>log(4.5×10^{-3})+1+2<br>-2.3+3=0.7	1	B_2+m<br>log(1)+1	(1) B_1+n_1'+n_2' > γ_2<br>log(4.5×10^{-3})+1+2>1<br>(2) B_2+m > γ_2<br>log(1)+1>1
log(10)<br>log(10)<br>log(10)	"1"	B_1+n_1'+n_2'<br>log(4.5×10^{-3})+1+2<br>-2.3+3=0.7	1	B_2+n_3'+m<br>log(1)+1.25+1	(1) B_1+n_1'+n_2' > γ_2<br>log(4.5×10^{-3})+1+2>1<br>(2) B_2+n_3'+m > γ_2<br>log(1)+1.25+1>1

Furthermore, in computation and signal processing, there are several advantages to using soft classification rather than hard classification. For example, our theoretical analysis showed that the three-input majority function can be implemented with a single layer of a step activation function (e.g., the three input weights are equal to one, and the threshold equals 1.5). However, biologically implementing an activation function (e.g., a promoter with three different binding sites) with three inputs and obtaining the desired parameters (weights and bias) is often challenging. Alternatively, we initially thought to implement a three-input majority function by cascading two perceptrons, each having two inputs. From our analysis of such a network, only perceptrons with a sigmoid activation function can satisfy the mathematical constraints on the design parameters to implement a three-input majority function. Therefore, the only feasible way to build a genetic circuit in *E.coli* that computes the majority of three inputs is by using two layers of a two-input sigmoid activation function, as was implemented in our study (Fig. 3).

Theoretical analysis of three-input Majority design using single layer of step activation function:

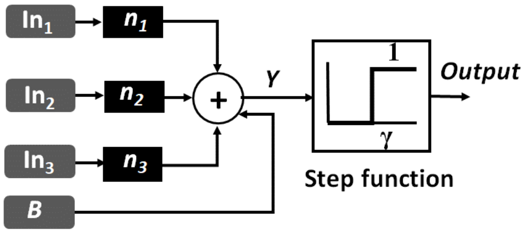

According to the schematic diagram above, we obtain :

$Y = n_1 \cdot I_{n1} + n_2 \cdot I_{n2} + n_3 \cdot I_{n3} + B$

$Output = \begin{cases} 0 & Y < \gamma \\ 1 & Y > \gamma \end{cases}$

For each of the eight input states, we first compute Y and then obtain the constrains on the design parameters to get the required Output as shown in the table below:

Supplementary Table 22: Design constraints to implement a three-input majority function using a single layer with step activation function.

I_{n1} I_{n2} I_{n3}	Out	Y	Constraints on Design parameters
0 0 0	0	Design constraints subsumed by 001, 010,100 case	
0 0 1	0	$B+n_3$	$B+n_3< \gamma$
0 1 0	0	$B+n_2$	$B+n_2< \gamma$
0 1 1	1	$B+n_2+n_3$	$B+n_2+n_3> \gamma$
1 0 0	0	$B+n_1$	$B+n_1< \gamma$
1 0 1	1	$B+n_1+n_3$	$B+n_1+n_3> \gamma$
1 1 0	1	$B+n_1+n_2$	$B+n_1+n_2> \gamma$
1 1 1	1	Design constraints subsumed by 110, 101, 011 cases	

To implement three-input majority function with a single layer of step-activation function based on the Table above Supplementary Table 22, our design parameter should satisfy:
Maximum{$B+n_1< \gamma$, $B+n_2< \gamma$, $B+n_3< \gamma$}, and minimum { $B+n_1+n_2> \gamma$, $B+n_1+n_3> \gamma$, $B+n_2+n_3> \gamma$} which is achievable.

Theoretical analysis of three-input Majority design using two layers of step activation-functions:

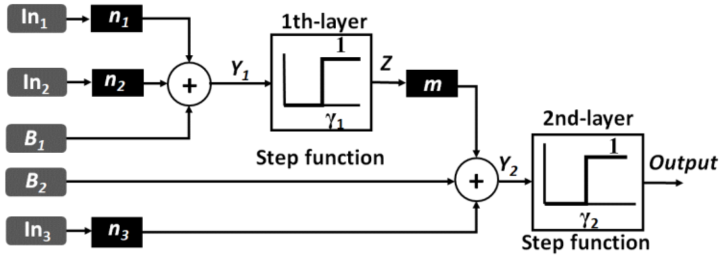

According to the schematic diagram above, we obtain:

$$Y_1 = n_1 \cdot I_{n1} + n_2 \cdot I_{n2} + B_1$$

$$Z = \begin{cases} 0 & Y_1 < \gamma_1 \\ 1 & Y_1 > \gamma_1 \end{cases}$$

$$Y_2 = n_3 \cdot I_{n3} + m \cdot z + B_2$$

$$Output = \begin{cases} 0 & Y_2 < \gamma_2 \\ 1 & Y_2 > \gamma_2 \end{cases}$$

For each of the eight input states, we first compute Y_1, Z, Y_2 and then obtain the constrains on the design parameters to get the required Output as shown in the table below:

Supplementary Table 23: Design constraints to implement a three-input majority function using two layers with step activation functions

I_{n1} I_{n2} I_{n3}	Out	Y_1	Z_1	Y_2	Constraints on Design parameters
0 0 0	0	Design constraints subsumed by 001 case			
0 0 1	0	B_1	*0*	B_2+n_3	$B_1 < \gamma_1$<br>$B_2+n_3 < \gamma_2$
0 1 0	0	B_1+n_2	*1*	B_2+m	$\boldsymbol{B_2+m < \gamma_2}$<br>$B_1+n_2>\gamma_1$
0 1 1	1	B_1+n_2	*1*	B_2+n_3+m	$B_1+n_2 > \gamma_1$<br>$B_2+n_3+m > \gamma_2$
1 0 0	0	B_1+n_1	*0*	B_2	$B_2< \gamma_2$
1 0 1	1	B_1+n_1	*1*	B_2+n_3+m	$B_1+n_1 > \gamma_1$<br>$B_2+n_3+m> \gamma_2$
1 1 0	1	$B_1+n_1+n_2$	*1*	B_2+m	$B_1+n_1+n_2 > \gamma_1$<br>$\boldsymbol{B_2+m > \gamma_2}$
1 1 1	1	Design constraints subsumed by 110, 101, 011 cases			

Based on the Supplementary Table 23, the constrain in state [010], $\boldsymbol{B_2+m < \gamma_2}$**,** is in contradictory with the constrain in state [110], $\boldsymbol{B_2+m < \gamma_2}$**.** *T*herefore, we conclude that we can not implement three majority function with two layers of step function.

Supplementary Table 24 List of parameters used in this section

Symbol	Description
y_i	The power law and multiplication signal (analog signal)
Y_{mi}	Fitting parameter that has concentration units
$K_{\ mi}$	Dissociation constant of binding x_i to Y_i
$n_{i,}m_i$	Hill coefficient
z_i	The expression level of the output protein proportional to the promoter activity
z_{mi}	The maximum expression level of the output protein

β_i	The basal level of the promoter
K_i	Dissociation constant of complex binding to promoter
a	Dissociation constant of binding Arabinose–AraC complex to P_{BAD}
b	Fitting parameter to the effective dissociation constant of binding Arabinose–AraC complex to P_{BAD}
B_i	Bias
I_{ni}	Normalized input

Supplementary Table 25 List of abbreviations used in this section

Symbol	Description
ANF	Auto-negative feedback
APF	Auto-positive feedback
IDR	Input Dynamic range
LTU	linear threshold unit
AHL	Free N-(β-Ketocaproyl)-L-homoserine Lactone $3OC_6HSL$ concentration
$IPTG$	Free Isopropyl1-β-D-1-thiogalactopyranside concentration
aTc	Free anhydrotetracycline
$AraC$	$AraC$ protein
LAA	ssrA degradation tag
$T7_{RNA}$	T7 RNA Polymerase
tRNA $supD$	Amber suppressor tRNA
P_{luxM56}	Mutated LuxR promoter is activated by the $LuxR$ when it is induced by AHL
$P_{lux/lacO}$	Combinatorial promoter
P_{T7}	T7 promoter
P_{TetO}	TetR promoter is activated by the $TetR - aTc$
$P_{luxM56/tetO}$	Combinatorial promoter
$P_{luxM56/lacO1}$	Combinatorial promoter
$P_{lux/tetO}$	Combinatorial promoter

6. Gradient descent and backpropagation algorithms in living cells

The perceptron weights can be adjusted in small steps through iterations, following a first-order optimization algorithm known as a gradient descent [27]. This process converges to the global minimum of the gradient descent of the mean square error metric function or cost function (C). Typically, this ensures that a high resolution of weight adjustments in tradeoff with training time and number of samples [28]. Likewise, we developed a perceptgene-based rule that minimizes the output error, following log-linear domain's gradient descent. We defined a logarithmically quadratic cost function for each state or sample i as:

$$C_i = \frac{1}{2}(\log(Z_{Di}) - \log(Z_i))^2 \quad (6.1)$$

Z_{Di} is the desired output of every state, and Z_i is the actual network output for every state. The cost function of network is the average of cost functions over individual samples:

$$< C >= \frac{1}{N}\sum_{i=1}^{N} C_i \quad (6.2)$$

N is the number of samples and is also called the batch size. Substituting Eq. 6.1 into Eq. 6.2, we get:

$$< C >= \frac{1}{2 \cdot N}\sum_{i}^{N}(\log(Z_{Di}/Z_i))^2 \quad (6.3)$$

We call C the logarithmically (average) *quadratic* cost function of the network, and it is a function of the weights and biases. We can see that $< C >$ function is non-negative, since every term in the sum is non-negative. Following the gradient descent, at every iteration, the network output is adjusted toward the desired value and accordingly, the average-cost function decreases (the cost function becomes small when the output is approximately equal to the desired value for all the samples i ($< C > \approx 0 \; when \; z_i \approx z_{Di}$). Supplementary Fig. 53 shows the average-cost function of a perceptgene network for two inputs and one output. In this case, there are four input states. We attempted to find an algorithm that minimizes the cost function on average with respect to the weights ($\partial < C > / \partial m_i$ or $\partial < C > / \partial n_i$ (m_i and n_i are network weights). In this work, we optimized the m_1 of the majority circuit, which consists of two layers (Supplementary Figs. 44 and 45). Therefore, we used a chain-rule in addition to gradient-descent to update the m_1. In particular at every iteration, we calculated the average error or cost at the output and distributed it back through the network layers which is essentially backpropagation algorithm (*7*):

$$\frac{\partial < C >}{\partial m_1} = \frac{1}{N}\sum_{i=1}^{N}\frac{\partial C_i}{\partial m_1} \quad (6.4)$$

We can write the set of equations that describe the majority circuit as: (we assumed that, the Basal levels are much lower than 1 ($\beta_{1,2} << 1$):

$$y_1 = \left(B_1 \cdot \left(\frac{AHL}{K_{m1}}\right)^{n_1} \cdot \left(\frac{IPTG}{K_{m2}}\right)^{n_2}\right)^{m_1} \quad (6.5)$$

$$z_1 \cong \frac{y_1}{1+y_1} + \beta_1 \quad (6.6)$$

$$y_2 = \left(B_2 \cdot \left(\frac{x_3}{K_{m3}}\right)^{n_3}\right)^{m_2} \quad (6.7)$$

$$y_3 = (B_4 \cdot y_2 \cdot (B_3 \cdot z_1)^{m_3})^{m_4} \quad (6.8)$$

$$z_2 \cong \frac{y_3}{1+y_3} + \beta_2 \quad (6.9)$$

Where $B_4 \equiv \frac{Y_{m3}}{K_5} \cdot \frac{K_3}{K_4}$, $B_3 \equiv \frac{Z_{m1}}{K_3}$, $B_2 \equiv \frac{Y_{m2}}{K_2}$, $B_1 \equiv \frac{Y_{m1}}{a} \cdot {m_1}^b$

Using a chain rule, for every state we can get:

$$\frac{\partial C_i}{\partial m_1} = \frac{\partial C_i}{\partial z_2} \cdot \frac{\partial z_2}{\partial y_3} \cdot \frac{\partial y_3}{\partial z_1} \cdot \frac{\partial z_1}{\partial y_1} \cdot \frac{\partial y_1}{\partial m_1} \quad (6.10)$$

$$\frac{\partial C_i}{\partial z_2} = \log\left(\frac{z_D}{z_2}\right) \cdot \frac{1}{z_2} \quad (6.11)$$

$$\frac{\partial z_2}{\partial y_3} \cong (z_2 - \beta_2) \cdot (1 - z_2) \cdot \frac{1}{y_3} \quad (6.12)$$

$$\frac{\partial y_3}{\partial z_1} = y_3 \cdot \frac{m_3 \cdot m_4}{z_1} \quad (6.13)$$

$$\frac{\partial z_1}{\partial y_1} \cong (z_1 - \beta_1) \cdot (1 - z_1) \cdot \frac{1}{y_1} \quad (6.14)$$

$$\frac{\partial y_1}{\partial m_1} = y_1 \cdot \left(\log\left(\frac{Y_{m1}}{a} \cdot {x_1}^{n_1} \cdot {x_2}^{n_2}\right) + b \cdot (\log(m_1) + 1)\right) \tag{6.15}$$

The partial derivative of the average-cost function of every sample or input state with respect to m_1 is:

$$\frac{\partial C_i}{\partial m_1} = m_3 \cdot m_4 \cdot \log(\frac{z_2}{z_D}) \cdot \left(\frac{z_2-\beta_2}{z_2}\right) \cdot (1-z_2) \cdot \left(\frac{z_1-\beta_1}{z_1}\right) \cdot (1-z_1) \cdot \left(\log\left(\frac{Y_{m1}}{a} \cdot {x_1}^{n_1} \cdot {x_2}^{n_2}\right) + b \cdot (\log(m_1) + 1)\right) \tag{6.16.1}$$

We normalized the partial derivative by $\log(\frac{z_{max2}}{z_{min2}})$ term, because the output dynamic range depends on the Arabinose level:

$$\frac{\partial C_i}{\partial m_1} = m_3 \cdot m_4 \cdot \frac{\log(\frac{z_2}{z_D})}{\log(\frac{z_{max2}}{z_{min2}})} \cdot \left(\frac{z_2-\beta_2}{z_2}\right) \cdot (1-z_2) \cdot \left(\frac{z_1-\beta_1}{z_1}\right) \cdot (1-z_1) \cdot \left(\log\left(\frac{Y_{m1}}{a} \cdot {x_1}^{n_1} \cdot {x_2}^{n_2}\right) + b \cdot (\log(m_1) + 1)\right) \tag{S6.16.2}$$

The weights adjustment in m_1 to minimize the average-cost function is:

$$\Delta m_1 = -\zeta \cdot \frac{1}{N}\sum_{i=1}^{N} \frac{\partial C_i}{\partial m_1} \tag{6.17}$$

Where ζ is a scalar and it determines the rate of m_1 being updated (also known as learning/training rate). The direction of the update is opposite to the partial derivative of a cost function, which guarantees that the weights adjustment is in the direction of a minimum, not a maximum, of the average-cost function. This technique calculates the average error at the output and distributes it back through the network layers. Therefore, it is also called "backward propagation of errors".

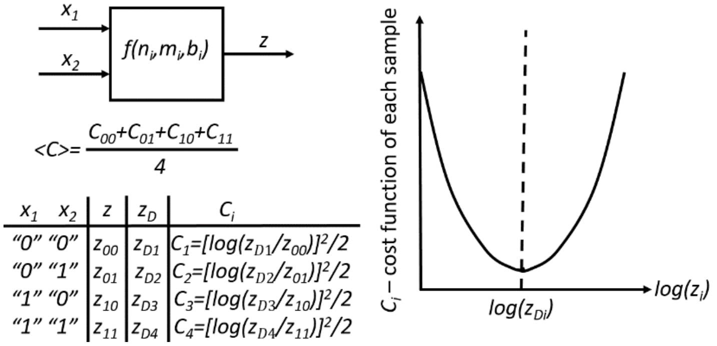

Supplementary Fig. 53. Logarithmically *quadratic* cost function of the perceptgene-based network

Eq. 6.16 and Eq. 6.17 have two important implications:

- The cost function of the network can be written as an average over cost functions for individual samples or input states.
- The cost function can be written as a function of the outputs of each layer independent on the analog signals of the network.

6.1. Calculation the experimental average (normalized) cost function

First, we calculated the average-cost function of the majority circuit based on Eq. 6.1-Eq. 6.3 using experimental results and compared the outcome of Eq. 6.16-Eq. 6.17. Subsequently, we changed m_1 by inducing the circuit with varying Arabinose concentrations (0.250, 0.125, 0.062, 0.031, 0.015, 0.007 mM). The relation between arabinose concentration and the weigh m_1 , was calculated based on the experiment in Supplementary Fig. 22a, and is shown in Supplementary Fig. 54a. Since the maximum level is measured strongly depends on the arabinose concertation (Supplementary Fig. 54b**)**, we defined a normalized average cost function. In this way, we can compare the average cost functions for the different Arabinose concentrations. The process that describing the calculation of average normalized cost function is given by:

1. Given and eights measured outputs: $Z_{xxx} = \{Z_{000}, Z_{001}, Z_{010}, Z_{011}, Z_{100}, Z_{101}, Z_{110}, Z_{111}\}$. Here each element of Z_{xxx} is average of three experiments.
2. Find the minimum between $Z_{min} = minimum\ \{Z_{000}, Z_{001}, Z_{010}, Z_{011}, Z_{100}, Z_{101}, Z_{110}, Z_{111}\}$.

3. Normalized the measured outputs by Z_{min}
   $Z_{nxxx} = \{\frac{Z_{000}}{Z_{min}}, \frac{Z_{001}}{Z_{min}}, \frac{Z_{010}}{Z_{min}}, \frac{Z_{011}}{Z_{min}}, \frac{Z_{100}}{Z_{min}}, \frac{Z_{101}}{Z_{min}}, \frac{Z_{110}}{Z_{min}}, \frac{Z_{111}}{Z_{min}}\}$.
4. The lowest desired value $Z_{nmin} = minimum\{Z_{nxxx}\} = 1$.
5. The highest desired value $Z_{nmax} = \text{maximum}\{Z_{nxxx}\}$.
6. Define normalized signal in the linear domain as $\frac{\log\left(\frac{Z_{xxx}}{Z_{min}}\right)}{Z_{nmax}}$.
7. $$< C_n >= \frac{1}{2 \cdot N}\sum_i^N \left(Z_{Di} - \frac{\log\left(\frac{Z_{xxx}}{Z_{min}}\right)}{Z_{nmax}} \right)^2, \quad Z_{Di} = \{0,1\} . \qquad (6.18)$$

Then, we simulated the process of updating m_1 by applying the backpropagation algorithm (Eq. 6.16-Eq. 6.17), as shown in Supplementary Fig. 55. We used desired values (z_{Di}) that are similar to experimental results: "0" =1 a.u., "1" = maximum normalized GFP (z_{D111}) for each Arabinose concentration. Therefore, we fitted the maximum normalized GFP (z_{D111}) to polynomial function as shown in Supplementary Fig. 54b**.** Hypothetically the relationship between maximum normalized GFP and Arabinose affects the partial derivative of the cost function ($\partial C/\partial m_1$).

The cost function in Fig. 3 in the main text are based on the experimental results in Supplementary Fig. 51 and Supplementary Eq. 6.18.

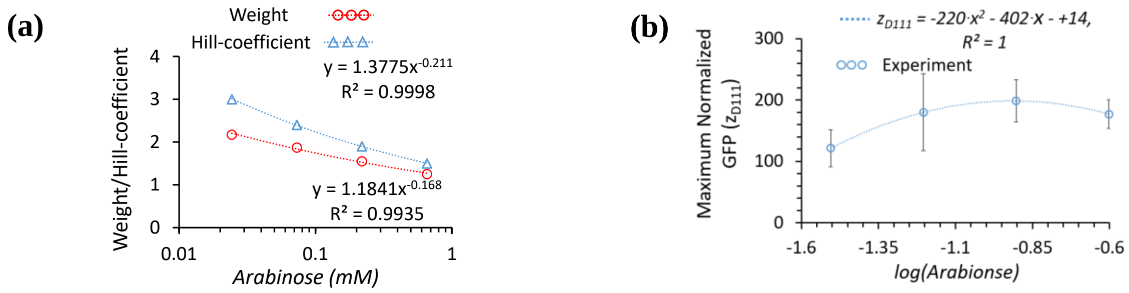

Supplementary Fig. 54. (a) The relation between Arabinose concentration and the weigh m_1, and which was calculated with new Arabinose values based on the experiment Supplementary Fig. 22a**. (b)** The maximum normalized signal achieved for each Arabinose concentration, was used as the "1" logic desired value for calculating the cost function. Data are presented as average ±standard deviations from independent replicates ($n = 3$).

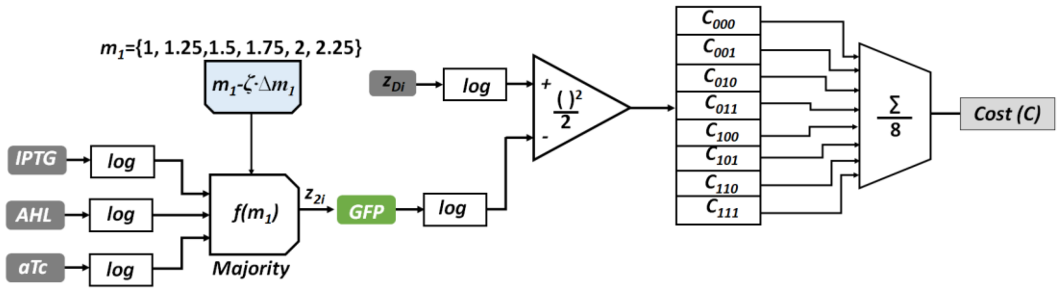

Supplementary Fig. 55. An algorithm for estimating and simulating cost function.

6.2. Backpropagation algorithm for two weights

Our next step is to use the backpropagation algorithm for programming two weights (m_1 for $\mathrm{P_{BAD}}$/AraC and n_1 for $\mathrm{P_{lux}}$/LuxR) within the majority function. To control the $\mathrm{P_{lux}}$/LuxR weight, we introduce random mutations to the operator sequence of a LuxRtranscription factor. Supplementary Fig. 87 describes seven modulations of transcription factor LuxR's DNA binding affinity via Lux operator sequence changes. Here, our backpropagation described in the following equations shows that it was enough to use less than four mutations TCTA, GTTG, GAGC and TGGG (PluxM56) for the APF loop of the first layer (Supplementary Fig. 56) to reach a majority function

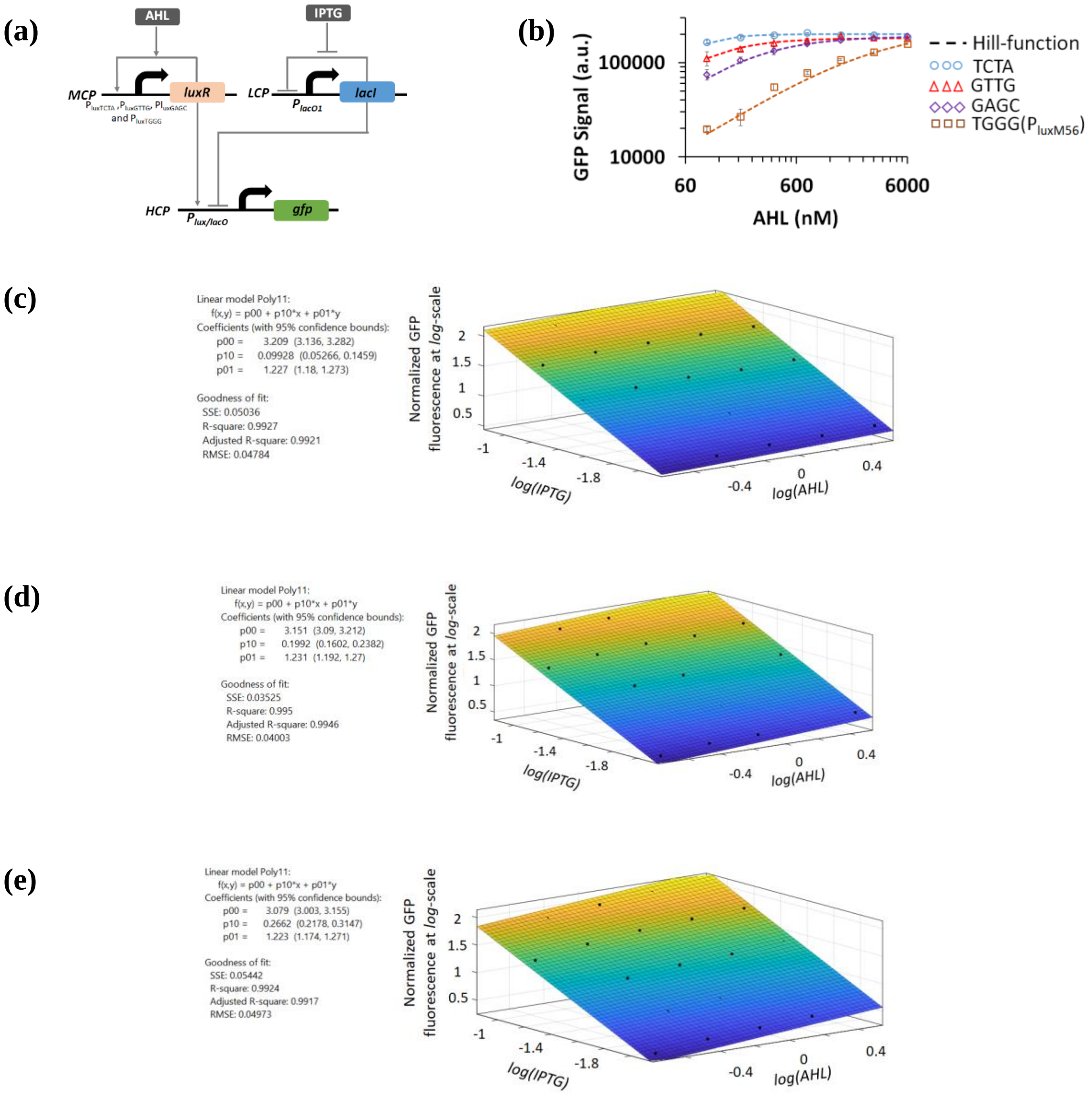

Supplementary Fig. 56. (a) $P_{luxNNNN}$-based APF, $\mathrm{P_{lacO1}}$-based ANF loops, and $P_{lux/lacO}$-based combinatorial promoter circuit, similar to circuit in Supplementary Fig. 48. **(b)** AHL − GFP transfer function for four different mutations (TCTA, GTTG, GAGC, and TGGG ($\mathrm{P_{luxM56}}$)) within $\mathrm{P_{lux}}$ promoter regulating LuxR by APF loop. IPTG = 0.125mM. Data are presented as average ±standard deviations from independent replicates ($n = 3$). **(c)** Matlab surface fits the experimental results of APF ($\mathrm{P_{luxTCTA}}$) and ANF ($\mathrm{P_{lacO1}}$) loops and combinatorial promoter ($\mathrm{P_{lux/lacO}}$-GFP) to power-law and multiplication function, the weight of AHL is 0.1, and the weight of IPTG is 1.23. **(d)** Matlab surface fits the experimental results of APF ($\mathrm{P_{luxGTTG}}$) and ANF ($\mathrm{P_{lacO1}}$) loops and

combinatorial promoter ($P_{lux/lacO}$-GFP) to power-law and multiplication function, the weight of AHL is 0.2, and the weight of IPTG is 1.23. **(e)** Matlab surface fits the experimental results of APF ($P_{luxGAGC}$) and ANF (P_{lacO1}) loops and combinatorial promoter ($P_{lux/lacO}$-GFP) to power-law and multiplication function, the weight of AHL is 0.27, and the weight of IPTG is 1.23. The results of P_{luxM56} and ANF (P_{lacO1}) loops and combinatorial promoter ($P_{lux/lacO}$-GFP) are presented in Supplementary Fig. 49.

Next, we modified the three-input perceptgene network from Supplementary Fig. 44 in the APF loop by changing the first four nucleotides in the P_{lux} promoter sequence as followed; TCTA, GTTG, GAGC (Supplementary Fig. 57). Then, we measured the GFP signal for all the four different circuits including P_{luxM56} across (Supplementary Fig. 44) the eight states of the inputs [AHL, IPTG, aTc]=[0,0,0], [0,0,1], [0,1,0], [0,1,1], [1,0,0], [1,0,1], [1,1,0], [1,1,1].The measured signals are presented in their absolute values (without normalization) and normalized values (Supplementary Fig. 57).

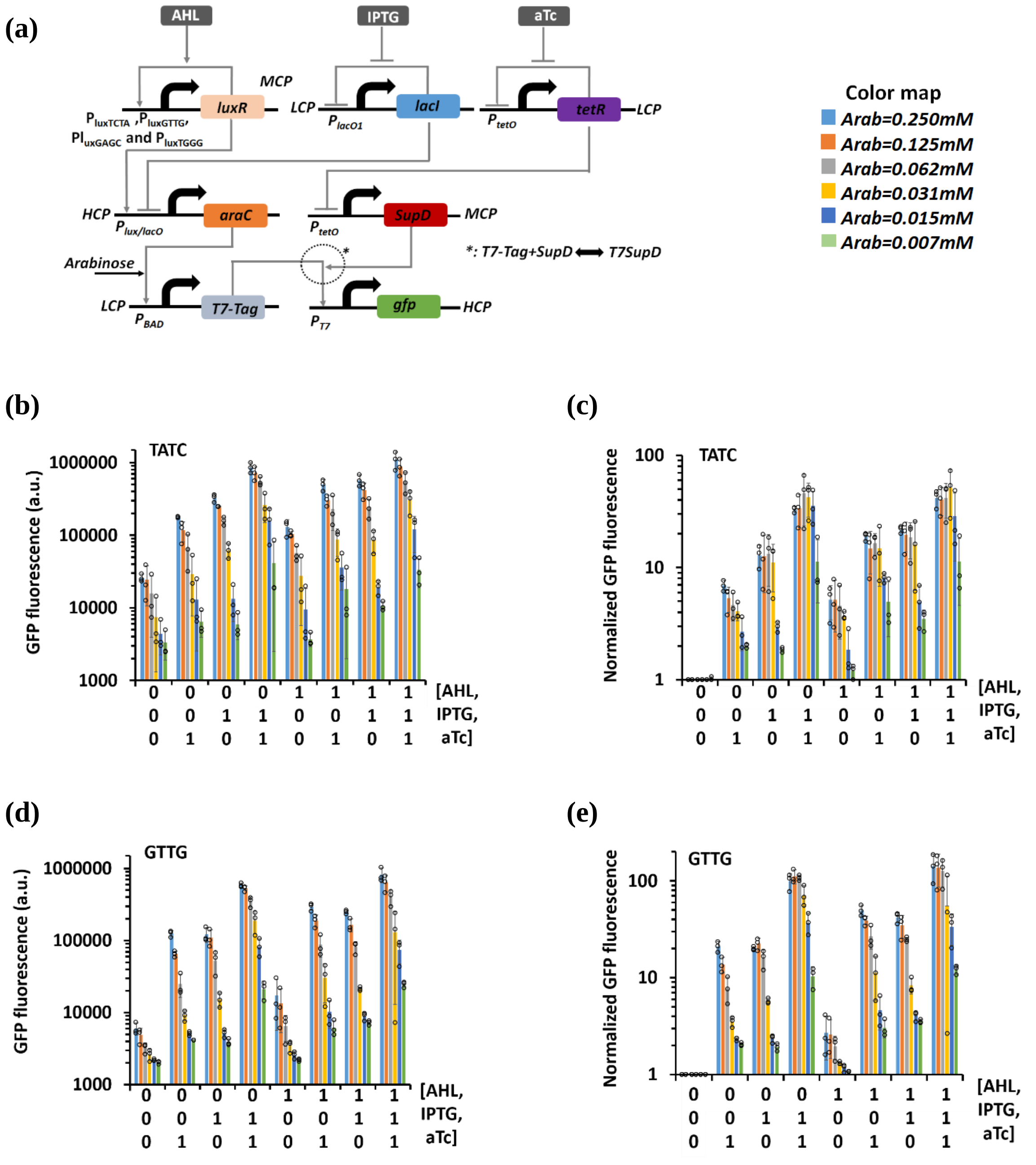

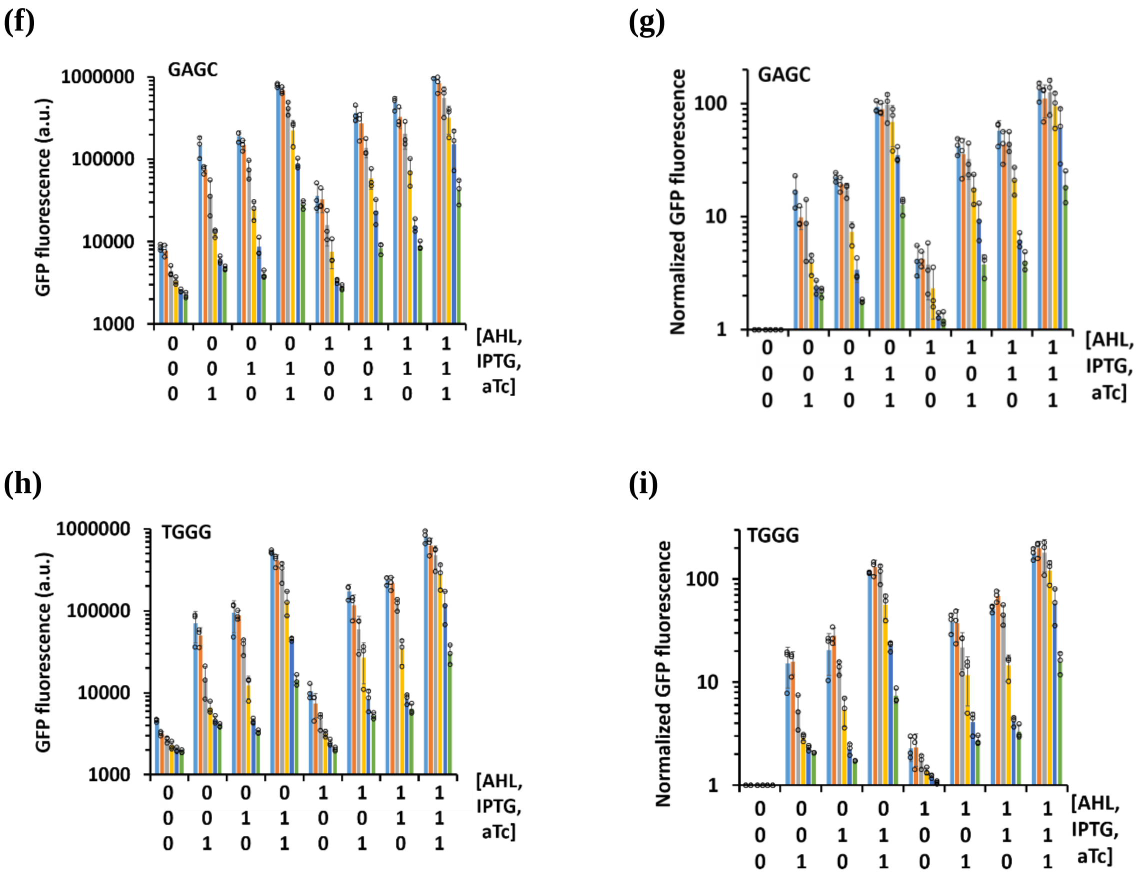

Supplementary Fig. 57. (a) Three-input perceptgene network accepts three analog inputs (AHL, IPTG and aTc) including four P_{lux} mutations (TCTA, GTTG, GAGC, TGGG (P_{luxM56})) within the APF in similar to Supplementary Fig. 44. **(b)** and **(c)** Experimental results of 3-input perceptgene network with TCTA for various Arabinose concentrations (0.250, 0.125, 0.062, 0.031, 0.015, 0.007 mM). The data is presented by absolute signals as measured by the Flow analyzer (b) and normalized signals (c). **(d)** and **(e)** Experimental results of 3-input circuit with GTTG for various Arabinose concentrations (0.250, 0.125, 0.062, 0.031, 0.015, 0.007 mM). The data is presented by absolute signals as measured by the Flow analyzer (d) and normalized signals (e). **(f)** and **(g)** Experimental results of 3-input circuit with GAGC for various Arabinose concentrations (0.250, 0.125, 0.062, 0.031, 0.015, 0.007 mM). The data is presented by absolute signals as measured by the Flow analyzer (f) and normalized signals (g). **(h)** and **(i)** Experimental results of 3-input circuit with TGGG for various Arabinose concentrations (0.250, 0.125, 0.062, 0.031, 0.015, 0.007 mM). The data is presented by absolute signals as measured by the Flow analyzer (h) and normalized signals (i). Colorful bars and their error bars show the average and standard deviation from independent replicates ($n = 3$). Median values of individual replicates are marked in circles.

The AHL − GFP transfer functions for the four P_{lux} mutations show that each modification has its slope (i.e., weight) and bias. Thus, there is a disturb between weight programming and bias levels. The AHL-GFP transfer functions for the four mutations can be written as:

$$GFP \propto B \cdot {n_1}^{-d} \cdot {x_1}^{n_1} \qquad (6.19)$$

Where = 3.7 . Supplementary Fig. 58 shows the dependency of the bias on the weight based on the data from Supplementary Fig. 56. The next step is to calculate the derivative of Eq. 6.19:

$$\frac{dGFP}{dn_1} \propto B \cdot {n_1}^{-d} \cdot {x_1}^{n_1} \cdot \left(log(x_1) - \frac{d}{n_1}\right) \qquad (6.20)$$

Eq. 6.20 shows that update in the weight also changes the bias. Based on that, we modified Eq. 6.5 to $B_1 = \frac{Y_{m1}}{a} \cdot {m_1}^b \cdot {n_1}^{-d}$. The partial derivative of average-cost function of every sample or input state with respect to n_1 is:

$$\frac{\partial <C>}{\partial n_1} = \frac{1}{N}\sum_{i=1}^{N}\frac{\partial C_i}{\partial n_1}$$

$$\Rightarrow$$

$$\frac{\partial C_i}{\partial n_1} = -m_3 \cdot m_4 \cdot \frac{\log(\frac{z_2}{z_D})}{\log(\frac{z_{max2}}{z_{min2}})} \cdot \left(\frac{z_2-\beta_2}{z_2}\right) \cdot (1-z_2) \cdot \left(\frac{z_1-\beta_1}{z_1}\right) \cdot (1-z_1) \cdot m_1 \cdot \left(log(x_1) - \frac{d}{n_1}\right) \qquad (S6.21)$$

In Eq. 6.21, we normalized the error $\log(\frac{z_{max2}}{z_{min2}})$, by output dynamic range $\log(\frac{z_{max2}}{z_{min2}})$, and this is because the output dynamic range depends on the Arabinose level. The partial derivative of average-cost function of every sample or input state with respect to m_1 is similar to Eq. 6.18 with a one modification; we included the $-d \cdot \log(n_1)$:

$$\frac{\partial C_i}{\partial m_1} = -m_3 \cdot m_4 \cdot \frac{\log(\frac{z_2}{z_D})}{\log(\frac{z_{max2}}{z_{min2}})} \cdot \left(\frac{z_2-\beta_2}{z_2}\right) \cdot (1-z_2) \cdot \left(\frac{z_1-\beta_1}{z_1}\right) \cdot (1-z_1) \cdot \left(b \cdot \log(m_1) + n_2 \cdot \log(x_2) + \log\left(\frac{Y_{m1}}{a}\right) + n_1 \cdot (-d \cdot \log(n_1) + \log(x_1)) + b\right) \qquad (6.22)$$

To calculate the update weights, based on Eq. 6.21 ad Eq. 6.22, we measured the output signals of the first layer (Z_1) as shown in Supplementary Fig. 59 and the output signals of the **three-input perceptgene network** (Z_2) as shown in Supplementary Fig. 57. We also measured the basal level of each activation function (β_1 and β_2) by measuring Z_1 and Z_2 when no inducers (AHL, IPTG and aTc) were added to the networks (Supplementary Table 26). The parameters ($b=0.37$, $\frac{Y_{m1}}{a} = 0.0001$) are estimated from the data shown in Supplementary Fig. 22, $d = 3.7$ is estimated from Supplementary Fig. 58, n_1 and n_2 are estimated from Supplementary Fig. 56 and 49 (TCTA: 0.1, 1.23, GTTG: 0.2, 1.23, GAGC: 0.27, 1.23, TGGG: 0.45, 1.23), $m_3 = m_4 = 1$. The first layer is the perceptgene of two inputs (AHL and IPTG) with four P_{lux} mutations of the APF loop; TCTA, GTTG, GAGC and TGGG (P_{luxM56}) (Supplementary Fig. 59a). The experimental results of backpropagation algorithms based on Eq. 6.21 and Eq. 6.22 are presented in Supplementary Table 27. Based on these results, we built an optimized pathway to reach the best majority results. We first update m_1 and then n_1, which reach a minimum cost function. We marked the optimized pathway in Supplementary Table 27.

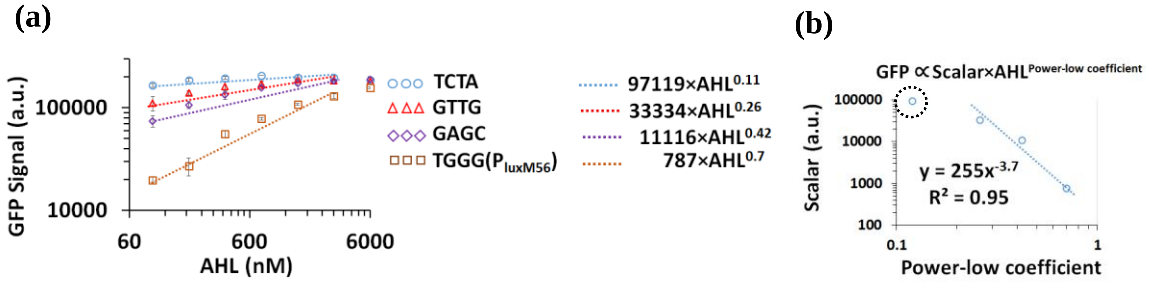

Supplementary Fig. 58. **(a)** Experimental results of $P_{luxNNNN}$-based APF, P_{lacO1}-based ANF loops, and $P_{lux/lacO}$-based combinatorial promoter circuit fits power-law function for $IPTG = 0.125\,mM$. The power-law coefficient is proportional to weight. Data are presented as average ±standard deviations from independent replicates ($n = 3$). **(b)** The scalar number in the power-law fitting from (a), which is proportional to bias, is also a function of the power-law coefficient. We exclusive the data of the TCTA mutation because the slope is low and inaccurate.

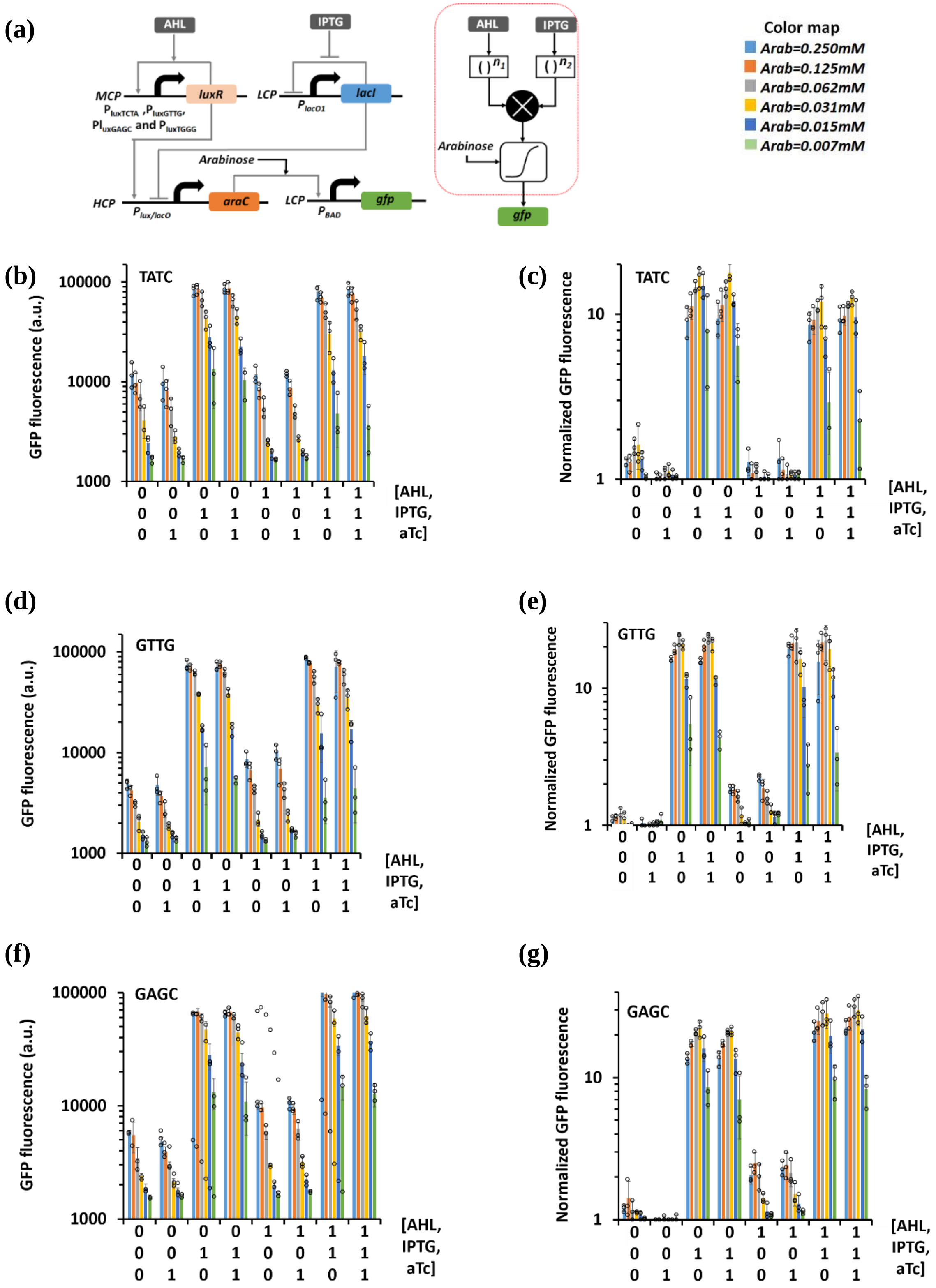
(a)
AHL
IPTG
MCP
luxR
LCP
lacI
$P_{luxTCTA}$, $P_{luxGTTG}$, $P_{luxGAGC}$ and $P_{luxTGGG}$
P_{lacO1}
Arabinose
HCP
$P_{lux/lacO}$
araC
gfp
P_{BAD}
Color map
Arab=0.250mM
Arab=0.125mM
Arab=0.062mM
Arab=0.031mM
Arab=0.015mM
Arab=0.007mM
(b)
TATC
GFP fluorescence (a.u.)
[AHL, IPTG, aTc]
(c)
TATC
Normalized GFP fluorescence
[AHL, IPTG, aTc]
(d)
GTTG
GFP fluorescence (a.u.)
[AHL, IPTG, aTc]
(e)
GTTG
Normalized GFP fluorescence
[AHL, IPTG, aTc]
(f)
GAGC
GFP fluorescence (a.u.)
[AHL, IPTG, aTc]
(g)
GAGC
Normalized GFP fluorescence
[AHL, IPTG, aTc]

(h)

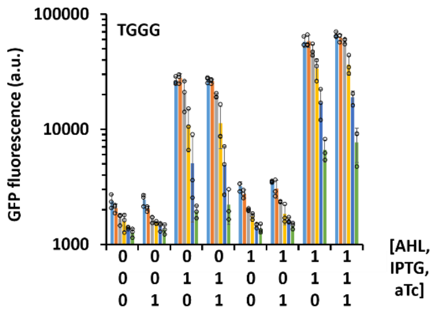

(i)

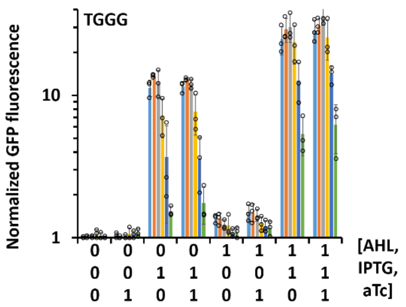

Supplementary Fig. 59. (a) The first perceptgene layer from the three-input perceptgene network (Supplementary Fig. 57). The layer accepts two analog inputs (AHL, IPTG) including four P_{lux} mutations (TCTA, GTTG, GAGC, TGGG (P_{luxM56})) within the APF. **(b)** and **(c)** Experimental results of 2-input circuit with TCTA for various Arabinose concentrations (0.250, 0.125, 0.062, 0.031, 0.015, 0.007 mM). The data is presented by absolute signals as measured by the Flow analyzer (b) and normalized signals (c). **(d)** and **(e)** Experimental results of 2-input circuit with GTTG for various Arabinose concentrations (0.250, 0.125, 0.062, 0.031, 0.015, 0.007 mM). The data is presented by absolute signals as measured by the Flow analyzer (d) and normalized signals (d). **(f)** and **(g)** Experimental results of 2-input circuit with GAGC for various Arabinose concentrations (0.250, 0.125, 0.062, 0.031, 0.015, 0.007 mM). The data is presented by absolute signals as measured by the Flow analyzer (f) and normalized signals (g). **(h)** and **(i)** Experimental results of 2-input circuit with TGGG for various Arabinose concentrations (0.250, 0.125, 0.062, 0.031, 0.015, 0.007 mM). The data is presented by absolute signals as measured by the Flow analyzer (h) and normalized signals (i). Colorful bars and their error bars show the average and standard deviation from independent replicates ($n = 3$). Median values of individual replicates are marked in circles.

Supplementary Table 26. Measured values of β_1 (basal level of the first layer, Supplementary Fig. 59a) and β_2 (basal level of the second layer, Supplementary Fig. 57a) for the four mutations. The measurements were performed when no inducers (AHL, IPTG, aTc) were added.

			Arabinose(mM)					
			0.2500	0.1250	0.0625	0.0313	0.0156	0.0078
TCTA	β_2	Average	1573	1739	1506	1651	1484	1603
		STDEV	97	78	37	49	76	47
	β_1	Average	2403	2003	1722	1453	1629	2512
		STDEV	33	430	97	159	311	1490
GTTG	β_2	Average	1469	1435	1491	1488	1530	1499
		STDEV	1	53	75	17	6	3
	β_1	Average	2185	1886	1643	1645	1378	1314
		STDEV	122	161	12	40	0	131
GAGC	β_2	Average	1443	1453	1448	1501	1504	1581
		STDEV	108	174	95	70	114	134
	β_1	Average	2317	2105	1743	1507	1505	1362
		STDEV	24	300	169	13	66	1
TGGG	β_2	Average	1466	1444	1912	1538	1522	1563
		STDEV	26	68	641	35	129	141
	β_1	Average	1609	1481	1184	1457	1264	1187
		STDEV	26	68	641	35	129	141

Supplementary Table 27. The experimental results of backpropagation algorithms based on Supplementary Eq. 6.21 - Eq. 6.22.

TCTA mutation, n_1 =0.1 weight					
Arabinose (mM)	m_1 weight	Experimental cost function	Digital cost function	$\frac{\partial < C >}{\partial m_1}$	$\frac{\partial < C >}{\partial n_1}$
**(1)** 0.2500	1	0.061	0.1250	0.23	-17.15
**(2)** 0.1250	1.25	0.057	0.0625	0.16	-16.08
0.0625	1.5	0.051	0.0625	0.13	-16.38
0.0312	1.75	0.051	0.0625	-0.01	-7.23
0.0156	2	0.047	0.0625	-0.5	14.88
0.0078	2.25	0.033	0.0625	-0.38	13.31

GTTG mutation, n_1 =0.2 weight					
Arabinose (mM)	m_1 weight	Experimental cost function	Digital cost function	$\frac{\partial < C >}{\partial m_1}$	$\frac{\partial < C >}{\partial n_1}$
0.2500	1	0.056	0.1250	-0.19	-6.59
**(3)** 0.1250	1.25	0.054	0.1250	-0.13	-5.98
**(4)** 0.0625	1.5	0.046	0.0625	-0.1	-1.45
0.0312	1.75	0.044	0.0625	-0.1	4.33
0.0156	2	0.051	0.1250	-0.03	3.21
0.0078	2.25	0.044	0.0625	-0.12	6.51

GAGC mutation, n_1 =0.27 weight					
Arabinose (mM)	m_1 weight	Experimental cost function	Digital cost function	$\frac{\partial < C >}{\partial m_1}$	$\frac{\partial < C >}{\partial n_1}$
0.2500	1	0.058	0.1250	-0.36	-6.63
0.1250	1.25	0.052	0.0625	-0.27	-7.11
**(5)** 0.0625	1.5	0.046	0.0625	-0.15	-6.15
**(6)** 0.0312	1.75	0.035	0	0.01	-1.53
0.0156	2	0.042	0.0625	0.04	1.95
0.0078	2.25	0.045	0.1250	0.03	1.16

TGGG mutation, n_1 =0.45 weight					
Arabinose (mM)	m_1 weight	Experimental cost function	Digital cost function	$\frac{\partial < C >}{\partial m_1}$	$\frac{\partial < C >}{\partial n_1}$
0.2500	1	0.052	0.1250	-0.49	-2.83
0.1250	1.25	0.052	0.1250	-0.37	-2.13
0.0625	1.5	0.038	0.0625	-0.17	-2.30
0.0312	1.75	0.039	0	0	-0.66
0.0156	2	0.061	0.1250	0.12	1.83
0.0078	2.25	0.056	0.1250	0.10	2.13

(a)

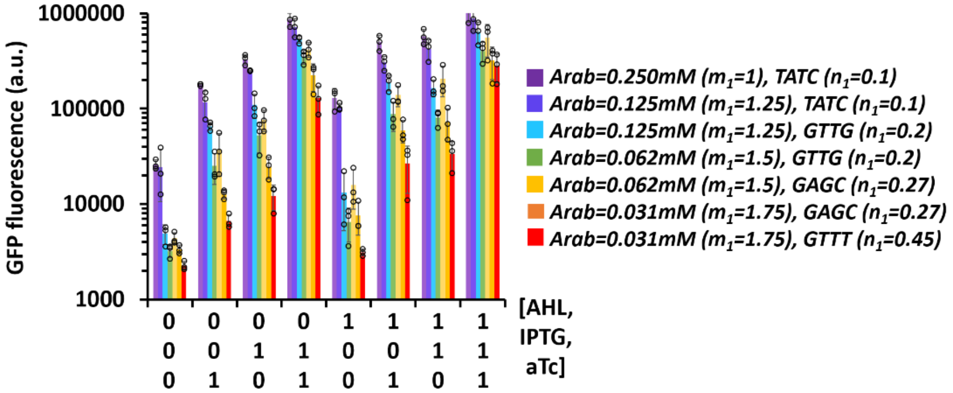

(b)

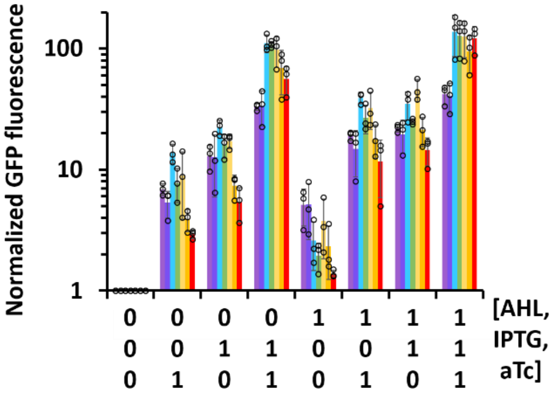

Supplementary Fig. 60. (a) The experimental results of the three-input perceptgene network accept three analog inputs (AHL, IPTG and aTc) to reach a minimum cost function. This data is based on Supplementary Fig. 57. **(b)** Normalized. Colorful bars and their error bars show the average and standard deviation from independent replicates ($n = 3$). Median values of individual replicates are marked in circles.

Supplementary Table 28 List of parameters used in this section

Symbol	Description
C	Cost function of network
C_i	logarithmically quadratic cost function for each state or sample i
m_i	network weights
n_i	network weights
β	Basal level
ζ	Scalar that determines the rate of m_1 being updated (also known as learning/training rate)
Z_i	the network actual output for every state
Z_{Di}	the desired output of every state
K_{mi}	Effective dissociation constant
z_1, z_2	the output of every perceptgene layer in the biophysical model
y_i	the analog signal of every perceptgene layer in the biophysical model
N	the number of samples and is also called the batch size

7. Synthetic Data converters

In this work, we designed and built two types of data converters that operate in the logarithmic domain:

1. Analog-to-Digital converter (ADC) which converts analog signals on a logarithmic scale to digital outputs, as shown in Supplementary Fig. 61a. Namely, each decade is encoded to one discrete level [29].
2. Analog-to-multilevel (Fuzzy) converter which converts analog signals on a logarithmic scale to multi-discrete levels as shown in Supplementary Fig. 61b. Specifically, we built a ternary converter.

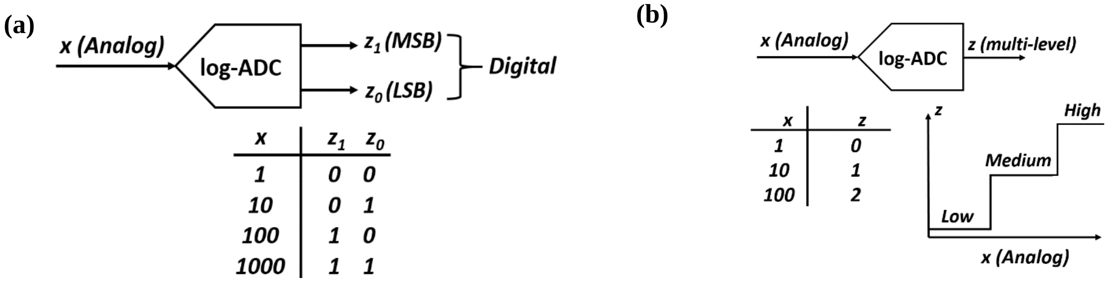

Supplementary Fig. 61. A 2-bit data converter operating on the logarithmic domain: **(a)** ADC-Analog-to-Digital converter. **(b)** Analog-to-multilevel (Fuzzy) converter. LSB: Least Significant Bit, MSB: Most Significant Bit.

7.1. Design I: Design and implementation of 2-bit log-ADC

There are several architectures and concepts to design ADC systems [30]. we used design principles of feed-forward neural networks [31,32] that can provide reliable results with a minimal number of synthetic parts (Supplementary Fig. 62a). First, we designed a 2-bit ADC in the linear domain (equivalent to a perceptron model), and then we transformed it to the logarithmic domain. In the proposed design, a bit comparison is equivalent to neural activation in the proposed design, and each reference scale during the successive binary search algorithm is equivalent to a binary-weighted synapse. For simplicity, we approximated the activation function as a step function:

$$y_1 = n_1 \cdot x + A_1 \quad (7.1)$$

$$Z_1 = \begin{cases} 1 & y_1 \geq 0 \\ 0 & y_1 < 0 \end{cases} \quad (7.2)$$

$$y_0 = n_0 \cdot x + m_0 \cdot z_1 + A_0 \quad (7.3)$$

$$Z_0 = \begin{cases} 1 & y_2 \geq 0 \\ 0 & y_2 < 0 \end{cases} \quad (7.4)$$

Supplementary Fig. 62b shows the output signals of 2-bit ADC in the linear domain. We divided the dynamic input range (IDR) to $2^2 = 4$ intervals. Therefore, the set of equations that describes the design of 2-bit ADC based on perceptron (Supplementary Fig. 62a) is given by:

Interval 1: $0 \leq x < \frac{IDR}{4}$ – $Z_1 = 0, Z_0 = 0$:

$$y_1 = n_1 \cdot \frac{IDR}{4} + A_1 \leq 0 \quad ➔ \quad A_1 \leq -n_1 \cdot \frac{IDR}{4} \quad (7.5)$$

$$y_0 = n_0 \cdot \frac{IDR}{4} + A_0 \leq 0 \quad ➔ \quad A_0 \leq -n_0 \cdot \frac{IDR}{4} \quad (7.6)$$

Interval 2: $\frac{IDR}{4} \leq x < \frac{IDR}{2}$ – $Z_1 = 0, Z_0 = 1$:

$$y_1 = n_1 \cdot \frac{IDR}{2} + A_1 < 0 \quad ➔ \quad A_1 < -n_1 \cdot \frac{IDR}{2} \quad (7.7)$$

$$y_0 = n_0 \cdot \frac{IDR}{2} + A_0 > 0 \quad ➔ \quad A_0 > -n_0 \cdot \frac{IDR}{2} \quad (7.8)$$

Interval 3: $\frac{IDR}{2} \leq x < \frac{3 \cdot IDR}{4}$ – $Z_1 = 1, Z_0 = 0$:

$$y_1 = n_1 \cdot \frac{3 \cdot IDR}{4} + A_1 > 0 \quad ➔ \quad A_1 > -n_1 \cdot \frac{3 \cdot IDR}{4} \quad (7.9)$$

$y_0 = n_0 \cdot \frac{3 \cdot IDR}{4} + m_0 + A_0 < 0 \qquad \rightarrow A_0 < -n_0 \cdot \frac{3 \cdot IDR}{4} - m_0 \qquad (7.10)$

Interval 4: $\frac{3 \cdot IDR}{4} \leq x \leq IDR \; - \; Z_1 = 1, Z_0 = 1$:

$y_1 = n_1 \cdot IDR + A_1 \geq 0 \qquad \rightarrow A_1 \geq -n_1 \cdot IDR \qquad (7.11)$

$y_0 = n_0 \cdot IDR + m_0 + A_0 \geq 0 \qquad \rightarrow A_0 \geq -n_0 \cdot IDR - m_0 \qquad (7.12)$

In summary, the conditions on the weights and biases to implement a 2-bit ADC in the linear domain:

$$-n_1 \cdot \frac{3 \cdot IDR}{4} < A_1 < -n_1 \cdot \frac{IDR}{2} \qquad (7.13)$$

$$-n_0 \cdot \frac{IDR}{2} < A_0 \leq -n_0 \cdot \frac{IDR}{4} \qquad (7.14)$$

$$-n_0 \cdot IDR - A_0 \leq m_0 < -n_0 \cdot \frac{3 \cdot IDR}{4} - A_0 \;\rightarrow m_0 = -n_0 \cdot \frac{IDR}{2} \qquad (7.15)$$

The simulation results of Supplementary Fig. 62c suggest that 2-bit ADC can be implemented by a feedforward neural network when the design parameters (weights and biases) satisfy Eq. 7.13-Eq. 7.15. The simulation parameters are $IDR = 5$, $n_0 = n_1 = 4, m_0 = -10, A_1 = -12.5, A_0 = -7.5$. In our simulation, we used a sigmoid function to calculate z_i ($z_i = \frac{1}{1+e^{-y_i}}$, the output of each perceptron), instead of step function as was done in the analysis, where all the outputs above 0.5 as a "1" logic and those below 0.5 as "0" logic. To implement such a design in living cells, we must convert the design parameters to the logarithmic domain. However, these parameters cannot be achieved in living cells directly, and thus the ADC design needs to be modified. In the proposed design, the MSB dynamically controls the LSB threshold through a negative regulation (inhibitory weight) (Supplementary Fig. 62d). The molecular 2-Bit ADC consists of two reactions (Z_0 and Z_1) which are regulated by the same input (x) (Supplementary Fig. 63a). Each reaction represents a digital bit, and Z_1 enhances the reverse reaction of Z_0. So that the amount of Z_1* changes and accordingly the amount of Z_0 is affected. The set of biochemical reactions that describe the reaction network is given by:

$$\frac{dZ_1^*}{dt} = k_{f1} \cdot x^{n_1} \cdot Z_1 - k_{r1} \cdot Z_1^* \qquad (7.16)$$

$$\frac{dZ_0^*}{dt} = k_{f0} \cdot x^{n_0} \cdot Z_0 - k_{10} \cdot {Z_1^*}^{m_0} \cdot Z_0^* - k_{r0} \cdot Z_0^* \qquad (7.17)$$

$$Z_{T1} = Z_1^* + Z_1 \qquad (7.18)$$

$$Z_{T0} = Z_0^* + Z_0 \qquad (7.19)$$

Where Z_0, and $Z_1$are the product concentrations of biochemical reactions. k_{f0} and k_{f1} are the rates for the forward reactions from Z_0 to Z_0 and Z_1 to Z_1, respectively. Likewise, k_{r0} and k_{r1} are the rates for the corresponding backward reactions. The rate k_{10} describes the regulation of Z_1 on the activation of Z_0. Z_{T0} and Z_{T1} are the total concentration of molecules Z_0 and Z_1. At the steady-state:

$$Z_1^* = Z_{T1} \cdot \frac{\left(\frac{x}{K_{n1}}\right)^{n_1}}{1+\left(\frac{x}{K_{n1}}\right)^{n_1}} \qquad (7.20)$$

$$Z_0^* = Z_{T0} \cdot \frac{\left(\frac{x}{K_{n0}}\right)^{n_0}}{1+\left(\frac{x}{K_{n0}}\right)^{n_0}+\left(\frac{Z_1^*}{K_{m0}}\right)^{m_0}} \qquad (7.21)$$

Where n_0, n_1 and m_0 are Hill Coefficients. K_{n0}, K_{n1} *and* K_{m0} are the dissociation constants ($K_{n0} = \left(k_{r0}/k_{f0}\right)^{1/n_0}, K_{n1} = \left(k_{r1}/k_{f1}\right)^{1/n1}, K_{m0} = (k_{r0}/k_{10})^{1/m_0}$). The reaction activity is defined as ratio of the product and the total concentration of molecules. Thus, we can rewrite Eq. 7.20 and Eq. 7.21 as:

$$P_1 = \frac{Z_1^*}{Z_{T1}} = \frac{\left(\frac{x}{K_{n1}}\right)^{n_1}}{1+\left(\frac{x}{K_{n1}}\right)^{n_1}} \qquad (7.22)$$

$$P_0 = \frac{Z_0^*}{Z_{T0}} = \frac{\left(\frac{x}{K_{n0}}\right)^{n_0}}{1+\left(\frac{x}{K_{n0}}\right)^{n_0}+\left(\frac{P_1}{K_{m0}/Z_{T1}}\right)^{m_0}} \qquad (7.23)$$

$$P_0 = \frac{Z_0^*}{Z_{T0}} = \frac{\left(\frac{x}{K_{n0}}\right)^{n_0} \cdot \left(\frac{P_1}{K_{m0}/Z_{T1}}\right)^{-m_0}}{\left(\frac{P_1}{K_{m0}/Z_{T1}}\right)^{-m_0}+\left(\frac{x}{K_{n0}}\right)^{n_0} \cdot \left(\frac{P_1}{K_{m0}/Z_{T1}}\right)^{-m_0}+1} \qquad (7.24)$$

In case that $Z_{T1} >> K_{m0}$, we can approximate Eq. 7.23:

$$P_0 \approx \frac{\left(\frac{x}{K_{n0}}\right)^{n_0} \cdot \left(\frac{P_1}{K_{m0}/Z_{T1}}\right)^{-m_0}}{\left(\frac{x}{K_{n0}}\right)^{n_0} \cdot \left(\frac{P_1}{K_{m0}/Z_{T1}}\right)^{-m_0} + 1} \quad (7.25)$$

Supplementary Fig. 63b shows a schematic model of the set of reactions based on Eq. 7.24 and Eq. 7.25. The schematic model consists of two perceptgenes that are connected in feedforward neural networks through a negative weight, similar to the ADC design (Supplementary Fig. 62a), where:

$$B_1 = K_{n1}^{-n_1} \quad (7.26)$$

$$B_0 = K_{n0}^{-n_0} \cdot \left(\frac{K_{m0}}{Z_{T1}}\right)^{m_0} \quad (7.27)$$

$$B_1 = 10^{A_1} \quad (7.28)$$

$$B_0 = 10^{A_2} \quad (7.29)$$

To estimate the weights and biases parameters, which operates in the log-domain, we transformed Eq. 7.13-Eq. 7.15 from the linear scale to the logarithmic scale using Eq. 7.26-Eq. 7.29:

(1) $-n_1 \cdot \frac{3 \cdot IDR}{4} < \log(B_1) < -n_1 \cdot \frac{IDR}{2}$

$$-\frac{3 \cdot IDR}{4} < -\log(K_{n1}) < -\frac{IDR}{2} \quad (7.30)$$

(2) $-n_0 \cdot \frac{IDR}{2} < \log(B_0) \leq -n_0 \cdot \frac{IDR}{4}$

$$-\frac{IDR}{2} < -\log(K_{n0}) + \frac{m_0}{n_0} \cdot \log\left(\frac{K_{m0}}{Z_{T1}}\right) \leq -\frac{IDR}{4} \quad (7.31)$$

(m_0>0)

(3) $-n_0 \cdot IDR - \log(B_0) \leq -m_0 < -n_0 \cdot \frac{3 \cdot IDR}{4} - \log(B_0)$

$$-IDR \leq -\log(K_{n0}) + \frac{m_0}{n_0} \cdot \log\left(\frac{K_{m0}}{Z_{T1}}\right) - \frac{m_0}{n_0} < -\frac{3 \cdot IDR}{4} \quad (7.32)$$

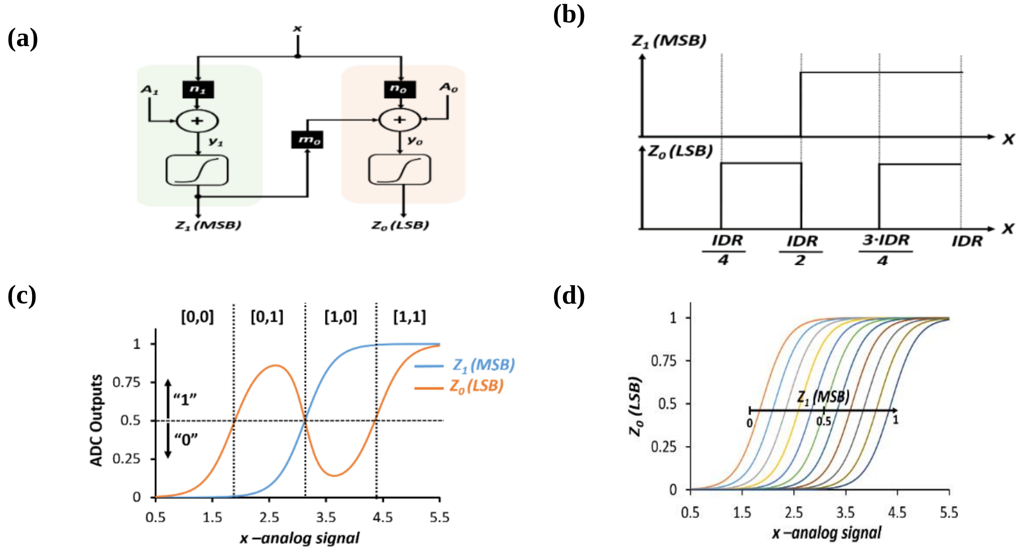

Supplementary Fig. 62. (a) Design of 2-bit ADC based on feedforward neural networks. **(b)** Digital outputs of 2-bit ADC operate in the linear domain. **(c)** Simulation results of 2-bit neural-network ADC design. **(d)** Most Significant Bit (MSB) dynamically controls the Least Significant Bit (LSB) of 2-bit ADC via varying the value of m_0. Experimental and simulation results. The horizontal dashed line, determined by the half of the fold change at the linear scale, separates between the "0" and "1".

The simulation results as shown in Supplementary Fig. 63c suggest that a 2-bit molecular ADC that operates in the log domain can be implemented using a feedforward neural network when the parameters (weights and biases) satisfy Eq. 7.30-Eq. 7.32. In the particular simulation, $IDR = 5, n_0 = n_1 = 2, m_0 = 4, K_{n0} = 25, K_{n1} = 800, K_{m0} =$

$40, Z_{T1} = 800, B_0 = 2.4 \times 10^{-11}, B_1 = 1.56 \times 10^{-6}, A_0 = -10.5, A_1 = -5.8$.Notably, operating in the log domain allows parameter values to be compressed, comparing with the ADCs in the linear domain with similar features $(IDR = 5, 2\ bits)$. That means the design parameters required to implement molecular ADC in living cells are achievable by contrast to linear ADC. Furthermore, varying m_0 (Hill coefficient) affects the LSB (Supplementary Fig. 63d), in particular, the behavior of LSB is qualitatively changed. As in Supplementary Fig. 63e when $n_0 \approx m_0$, LSB acts as ternary logic **(**$IDR = 5, n_0 = n_1 = 1.5,\ K_{n0} = 25, K_{n1} = 500,\ K_{m0} = 40,\ Z_{T1} = 700, m_0 = 1.5$ (for ternary logic, the blue curve**)** and $m_0 = 2.5$ (for quaternary logic, the red curve).

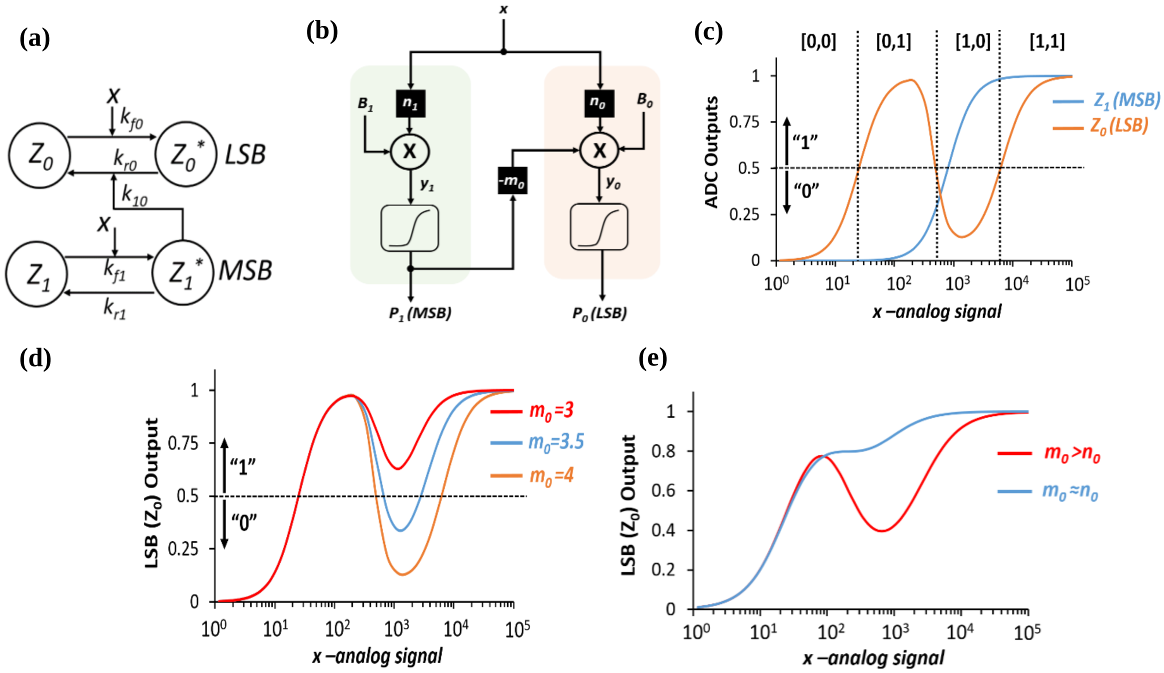

Supplementary Fig. 63. (a) Design of 2-bit molecular ADC by controlling LSB via MSB. **(b)** An abstract model of 2-bit molecular ADC based on the perceptgene feedforward network. **(c)** Simulation results of 2-bit molecular ADC. **(d)** The influence of m_0 on the LSB behavior. **(e)** 2-bit Molecular ADC displays ternary logic by programming the m_0 weight. Experimental and simulation results. The horizontal dashed line, determined by the half of the fold change at the linear scale, separates between the "0" and "1"

7.1.1. Optimization process of 2-bit ADC

In this process, we changed the original mathematical model (Supplementary Fig. 63) to be suitable for genetic networks. Supplementary Fig. 64a is built based on Supplementary Fig. 63b, including:

1. Positive feedback loop for linearization.
2. AraC as a new wire to implement the LSB.
3. Transcriptional interference promoter to implement the subtraction.

Supplementary Fig. 64b is built based on Supplementary Fig. 64a, including:

1. $m_0 = -1$, since there is only one binding site of LuxR in the transcriptional interference promoter.
2. Inhibition to P_{BAD}, which is achieved by the transcriptional interference of P_{lux} promoter.

Supplementary Fig. 64c is the final construct which is built based on Supplementary Fig. 64b, including the TetR repressor to reduce the disturb of the inhibition.

(a)

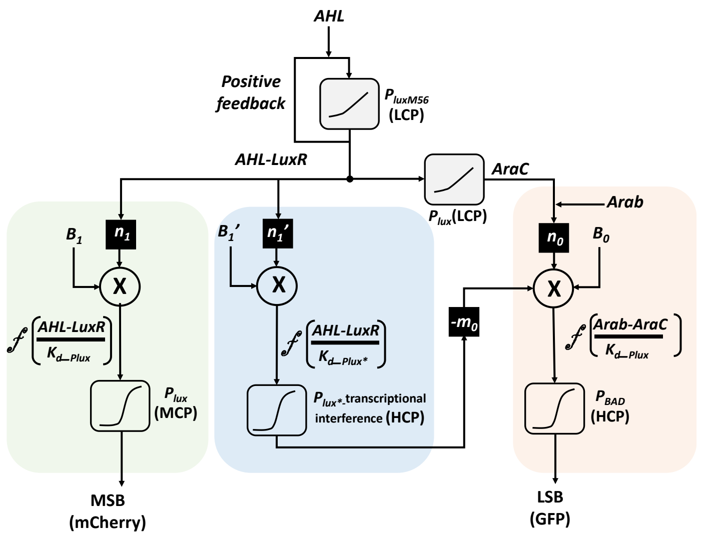

(b)

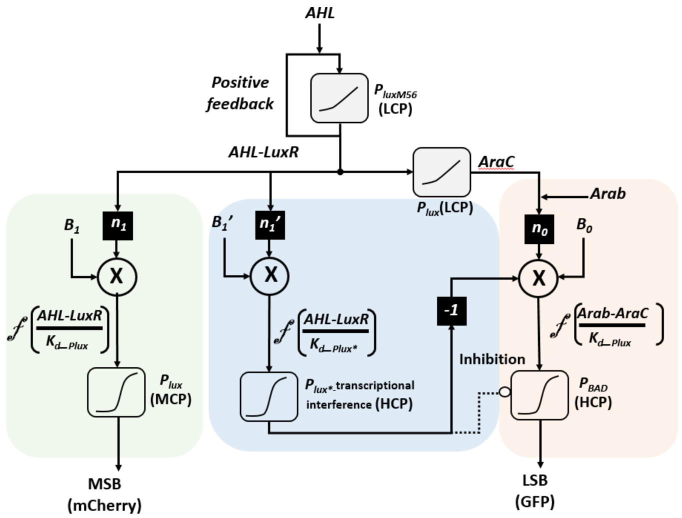

(c)

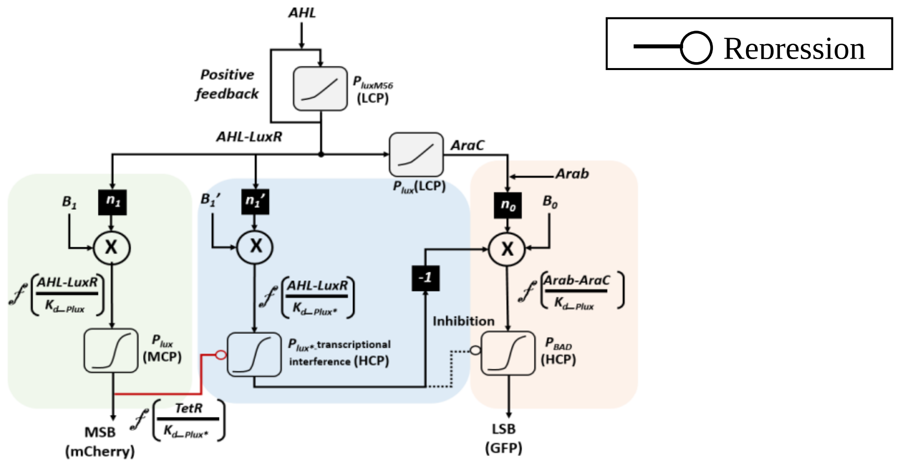

Supplementary Fig. 64. Process optimization of 2-bit ADC.

To implement the 2-bit Molecular ADC in living cells, we first constructed a genetic circuit with an effective tunable threshold. To that end, we utilized two competitive promoters (P_{BAD} vs P_{lux}) that are located in opposite orientation to each other, which P_{lux} produces transcriptional interference with P_{BAD} (Supplementary Fig. 65a). For P_{BAD} we call forward promoter and for P_{lux} we call reverse promoters. Oppositely oriented promoters relative to a gene have been reported in previous studies [33–35] to tune gene expression [36], control the input threshold of genetic switches [37,38], and reduce the leaky expression of toxic proteins [39]. A RiboJ [40] was used to cleave the 5′-UTR of GFP mRNA, a computationally designed RBS, the GFP-coding sequence and a transcriptional terminator. A reverse complementary terminator was cloned upstream to P_{BAD} to disturb the activity of RNA polymerase for P_{lux}. Adetailed biophysical model was developed [36] to describe such systems, in our work, for simplicity. Because the two opposite promoters are located close to each other, we treat the system as one statistical thermodynamic model (Supplementary Fig. 65b). The model describes 5 different statistical states, (1) promoters are empty, (2) RNA polymerases (RNAp) with transcription factors are bound on both promoters, leading to a basal level, (3) the complex activator (Y_1)-RNAp is bound to the forward promoter, leading to active the output signal, (4) the complex activator (Y_2)-RNAp is bound to the reverse promoter, leading to inhibit the output signal, and (5) the complex activator (Y_1)-RNAp is bound to the forward promoter, and the complex activator (Y_2)-RNAp is bound to the reverse promoter. In our model, we also assume that the collision interference is large and thus the probability that the forward and the reverse RNA polymerases can simultaneously bind to the DNA is very low ($\theta << 1$). Therefore, the level of gene expression is proportional to the probability (P) that RNA polymerase is bound to the forward promoter at the equilibrium:

$$P = \frac{\left(\frac{Y_1}{K_{d1}}\right)^{n_1}+\beta}{1+\left(\frac{Y_1}{K_{d1}}\right)^{n_1}+\left(\frac{Y_2}{K_{d2}}\right)^{n_2}} \quad (7.33)$$

Y_1 is the concentration of Arab-$AraC$ complex. Y_2 is the concentration of AHL -LuxR complex. These variables are given by the following set of equations:

$$g(x_i) = \frac{\left(\frac{x_i}{K_{mi}}\right)^{m_i}}{1+\left(\frac{x_i}{K_{mi}}\right)^{m_i}} \quad (7.34)$$

$$Y_1 = AraC_T \cdot g(Arab) \quad (7.35)$$

$$Y_2 = LuxR_T \cdot g(AHL) \quad 7.36)$$

We can rewrite the promoter activity (Eq. 7.33) as follows:

$$P = \frac{\frac{AraC_T \cdot g(Arab)}{K_{deff}}+\beta_{eff}}{1+\frac{AraC_T \cdot g(Arab)}{K_{deff}}} \quad (S7.37)$$

$$\beta_{eff} = \frac{\beta}{1+\frac{LuxR_T \cdot g(AHL)}{K_{d2}}} \quad (7.38)$$

$$K_{deff} = K_{d1} \cdot \left(1+\frac{LuxR_T \cdot g(AHL)}{K_{d2}}\right) \quad (7.39)$$

Where we assume that $n_1 = 1, n_2 = 1$. Supplementary Fig. 65c shows the experimental and simulation results of the normalized signals for the forward (P_{BAD}) promoter and reverse promoter (P_{lux}) using the set of Eq. 7.37- Eq. 7.39. For each AHL concentration, we normalized the measured GFP by the maximum achieved GFP level. Assuming that Arabinose>>K_{m1}, the fold-change of GFP can be given by:

$$\frac{P_{max}}{P_{min}} = \frac{AraC_T}{K_{deff}+AraC_T} \cdot \frac{1}{\beta_{eff}} \quad (7.40)$$

The experimental results show that varying the concentration of AHL affects the Arabinose-to-GFP transfer function. When AHL concentration increases, the fold-change of GFP decreases, which is consistent with Eq. 7.40. The experimental results can be well captured by the simulation results. Also, our experimental and simulation results imply that there is interference between the P_{BAD} promoter and P_{lux}, leading to a shift in the threshold or effective dissociation constant by 1.5 magnitudes of orders. Parameters that were used in the simulation are $K_{m1} = 1000, m_1 = 1.5, K_{m2} = 0.3, m_2 = 1, AraC_T/K_{d1} = 11, LuxR_T/K_{d2} = 10, \beta = 0.0055$. To fit the absolute GFP signals to our simulation results, we modified Eq. 7.37 by including a repression term:

$$GFP = GFP_{max} \cdot \frac{\frac{AraC_T \cdot g(Arab)}{K_{deff}} + \beta_{eff}}{1+\frac{AraC_T \cdot g(Arab)}{K_{deff}}} \cdot \left(\frac{1}{1+\rho \cdot \frac{LuxR_T \cdot g(AHL)}{K_{d2}}} + \beta_2\right) \tag{7.41}$$

Supplementary Fig. 65d shows that Eq. 7.41 captures well the behavior of the absolute GFP signals ($GFP_{max} = 5.5 \times 10^5, \beta_2 = 0.04, \rho = 0.8$). The repression term takes effect when $\theta > 0$ in Eq. 7.33. In conclusion, the competition between the two promoters increases the threshold, and decreases the promoter activity (signal).

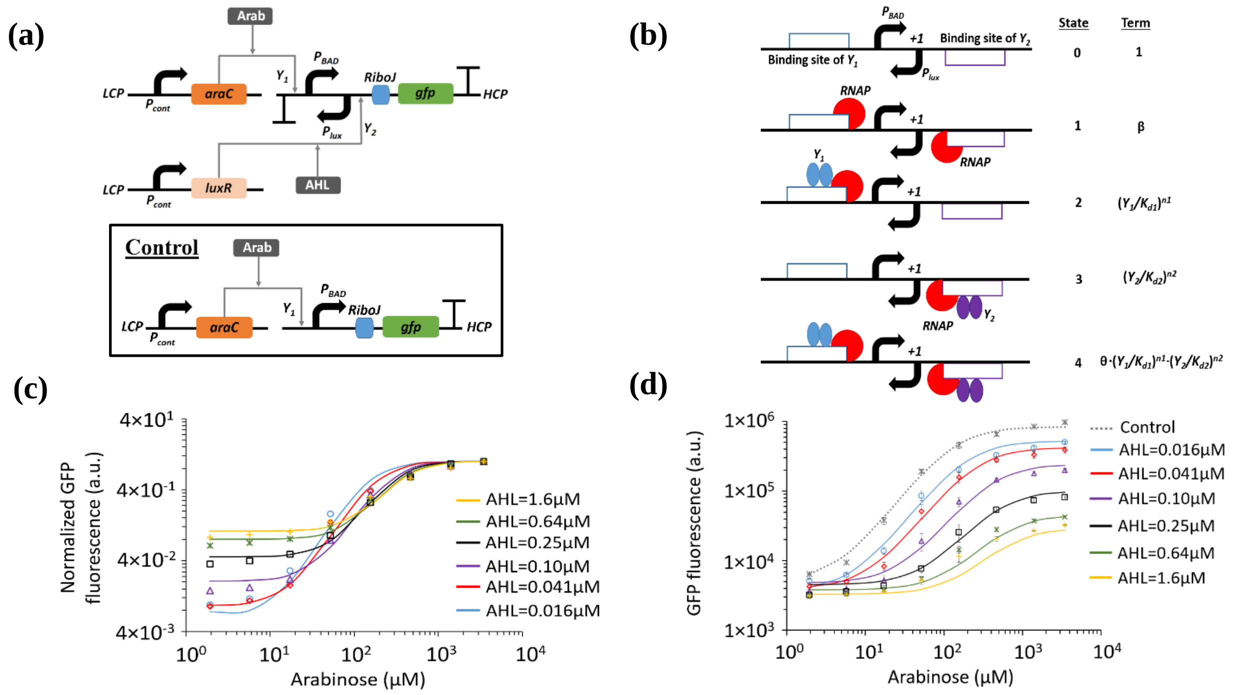

Supplementary Fig. 65. (a) Genetic circuit with a tunable effective threshold based on a forward (P_{BAD}) promoter and a reverse (P_{lux}) promoter that is oriented in opposite direction to P_{BAD}. The $P_{lux}$is having a transcriptional interference with P_{BAD}. **(b)** The binding states of the forward promoter and the reverse P_{lux}. **(c)** Experimental and simulation (based on Eq. 7.37-Eq. 7.39) results of normalized measured GFP signal. **(d)** Experimental and simulation (Eq. 7.38-Eq. 7.39 and Eq. 7.41) results of absolute measured GFP signal. The control experiment is shown in Supplementary Fig. 65a inset and the experimental results are shown in Supplementary Fig. 65d. Data are presented as average ±standard deviations from independent replicates ($n = 3$).

Second approach: The optimization process of implementing a 2-bit Molecular ADC in living cells (Supplementary Fig. 66) consists of three steps:

1. Open loop circuit: AHL is the input signal. The AHL -LuxR complex binds to the mutated promoter P_{luxM56}. The latter promoter regulates AraC and A (another activator) in analog fashion. AraC affects the LSB by binding to P_{BAD}, and A affects the MSB by binding to P_1. The transcription factor B, which is regulated by P_1, regulates the P_{BAD} promoter.
2. PF circuit: we replaced the open loop with a positive feedback circuit.
3. We replaced the transcription factor B with LuxR. We used mutations of P_{lux} that has a different strength.

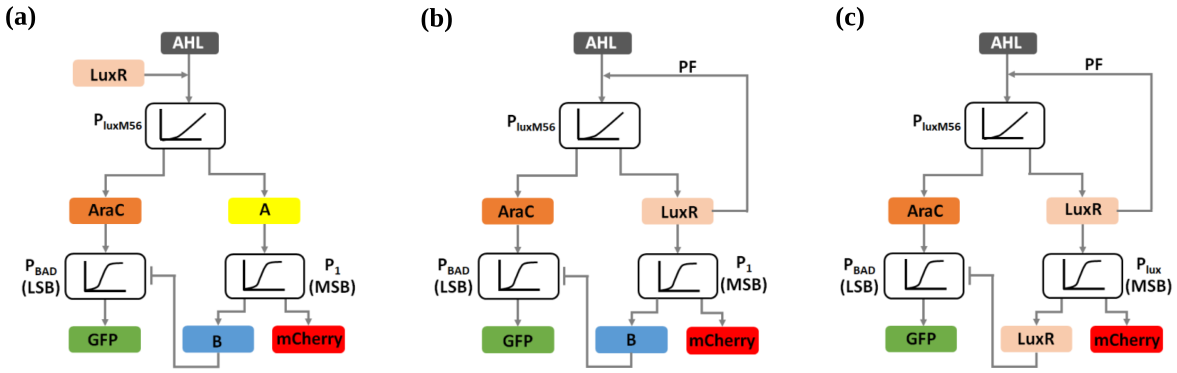

Supplementary Fig. 66. Optimization process of implementation 2-bit molecular ADC in living cells.

Based on our optimization, we created two circuits that implement LSB and MSB (Supplementary Figs. 67a and b), and another two control circuits (Supplementary Figs. 67c and d). The LSB and MSB circuits accept AHL as an analog input and include a graded PF (P_{luxM56}), which regulates LuxR in an analog fashion. The P_{lux} of LSB circuit is located on LCP and regulates AraC in an analog manner (Supplementary Fig. 26). In particular, we kept a low expression level of AraC by altering the binding between RNA polymerase and the promoter P_{lux} [41]. Subsequently, the Arabinose-AraC complex binds to the forward P_{BAD} the promoter, while the LuxR − AHL complex binds to the reverse P_{lux} promoter. The binding reaction of LuxR − AHL complex, causes the RNA polymerase to reverse P_{lux} dynamically increases the P_{BAD} threshold and decreases the GFP expression (Supplementary Fig. 68a). The MSB circuit that locates on MCP (Supplementary Fig. 67b), regulates the output signal in a digital fashion. Following the previous Eq. 7.34 to Eq. 7.41, a set of empirical models are used to describe the LSB and MSB signals:

$$MSB \propto \frac{\left(\frac{AHL}{K_1}\right)^{r_1}+\beta_1}{1+\left(\frac{AHL}{K_1}\right)^{r_1}} \quad (7.42)$$

$$LSB \propto \frac{\left(\frac{AHL}{K_0}\right)^{r_0}+\beta_0}{1+\left(\frac{AHL}{K_0}\right)^{r_0}+\alpha\cdot\left(\frac{MSB}{K_2}\right)^{r_2}}\cdot\left(\frac{1}{1+\left(\frac{AHL}{K_3}\right)^{r_3}}\right)+\beta_2 \quad (7.43)$$

To find the model parameters, we first fitted the control experimental results (Supplementary Fig. 68a) to a Hill-function (Eq. 7.42). Both control circuits contain a graded PF (P_{luxM56}) and P_{BAD} promoter. As a side note, control 1 appears similar to the circuit in Supplementary Fig. 22, except that a degradation tag was added to AraC. In control 2 circuit, the transcriptional interference is controlled by a constitutive promoter P_{const} to P_{BAD}, which reduced the basal level and increased the threshold of P_{BAD} activation (Supplementary Fig. 68a). The data was normalized by the maximum achieved level. The circuits were modeled as follows:

Control 1: $r_1 = 0.5, K_1 = 20$

Control 2: $r_1 = 0.7, K_1 = 50$

MSB: $r_1 = 1.5, K_1 = 500, \beta_1 = 0.1$

LSB: $r_0 = 0.8, K_0 = 40, \beta_0 = 0.001, \alpha = 200, r_2 = 1.5, K_2 = 10, r_3 = 1, K_3 = 500, \beta_2 = 0.04$

As in Supplementary Fig. 68b, the proposed LSB and MSB circuits successfully converted the dynamic range of AHL concentration to [0,0], [0,1] and [1,0] logic states, while failed to achieve the [1,1] state. This circuit acts a 1.5-bit ADC.

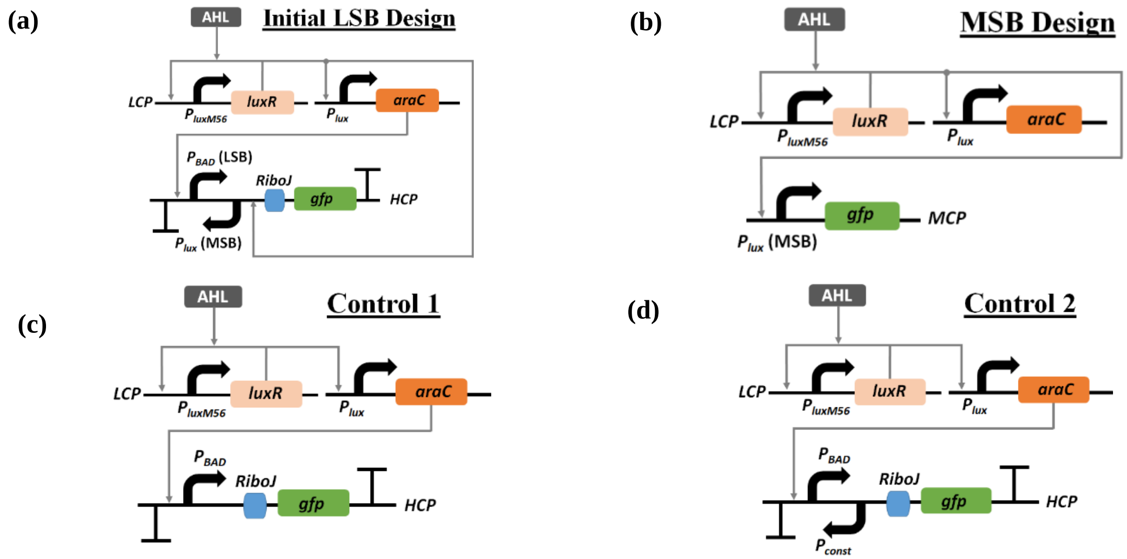

Supplementary Fig. 67. (a) The implementation of LSB genetic circuit using a forward P_{BAD} promoter and reverse P_{lux} promoter which produces a transcriptional interference. **(b)** The implementation of MSB. **(c)** Control 1 circuit. **(d)** Control 2 circuit using a constitutive promoter to produce the transcriptional interference.

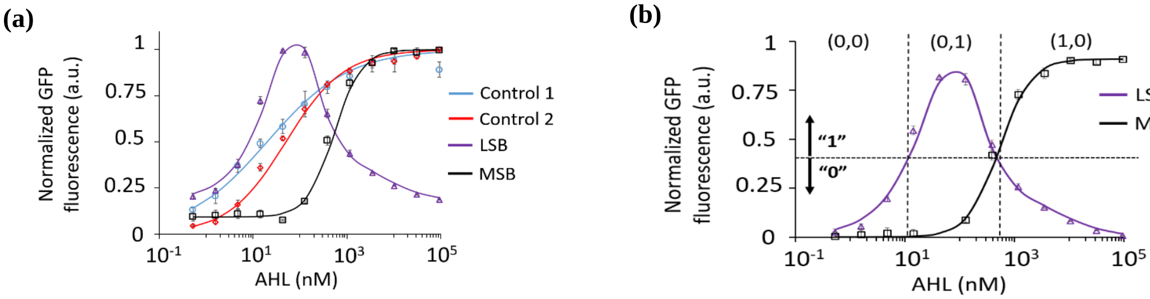

Supplementary Fig. 68. (a) Experimental results of LSB, MSB and control circuits. Solid lines indicate modeling results based on the empirical model (Eq. 7.42, Eq. 7.43). We used Arabinose of 0.05 M for all the circuits. Data are presented as average ±standard deviations from independent replicates ($n = 3$). **(b)** The normalized GFP signals of LSB and MSB referred to each basal level (the data was subtracted by the basal levels of LSB and MSB). Experimental and simulation results. The horizontal dashed line, determined by the half of the fold change at the linear scale, separates between the "0" and "1".

The failure of the proposed LSB and MSB circuits to achieve the [1,1] logic state is possibly due to the repression of the P_{lux}transcriptional interference with P_{BAD} promoter in the presence of high levels of AHL. To solve this issue, a repressor (TetR) was added to the MSB (Supplementary Fig. 69a). The TetR indirectly inhibits the activity of the P_{lux} transcriptional interference in the presence of high levels of AHL, through binding to the combinatorial $P_{lux/tetO}$ promoter (Supplementary Fig. 69b). To implement, we constructed a hybrid promoter that consists of a forward P_{BAD} promoter and a combinatorial $P_{lux/tetO}$ promoter which is oriented in opposite direction to P_{BAD} (Supplementary Fig. 69b). To understand the mechanism of $P_{lux/tetO}$, we created two control circuits (control 3 and 4) that are regulated by AHL and a graded PF and includes a forward P_{BAD} and a reverse combinatorial $P_{lux/tetO}$ as shown in Supplementary Fig. 69c. In control 3, TetR is regulated by a constitutive promoter, and in control 4 there is no expression of TetR. The experimental results of control circuits 3 and 4 (Supplementary Fig. 70) indicate that when TetR binds to the $P_{lux/tetO}$ promoter, there is no transcriptional interference with P_{BAD} promoter. Therefore, the control 3 circuit achieves a high GFP signal for high AHL levels. The results of control 3 are similar to those of control 1. The results of control 4 are similar to those of LSB circuit (Supplementary Fig. 68a). The data of control 3 and 4 are well fitted by our empirical model (Eqs. 7.42 and 7.43), with the parameters as follows:

Control 3: $r_1 = 0.7, K_1 = 200, \beta_1 = 0.07$

Control 4: $r_0 = 0.8, K_0 = 40, \beta_0 = 0.001, \alpha = 200, r_2 = 1.5, K_2 = 100, r_3 = 1, K_3 = 500, \beta_2 = 0.005.$

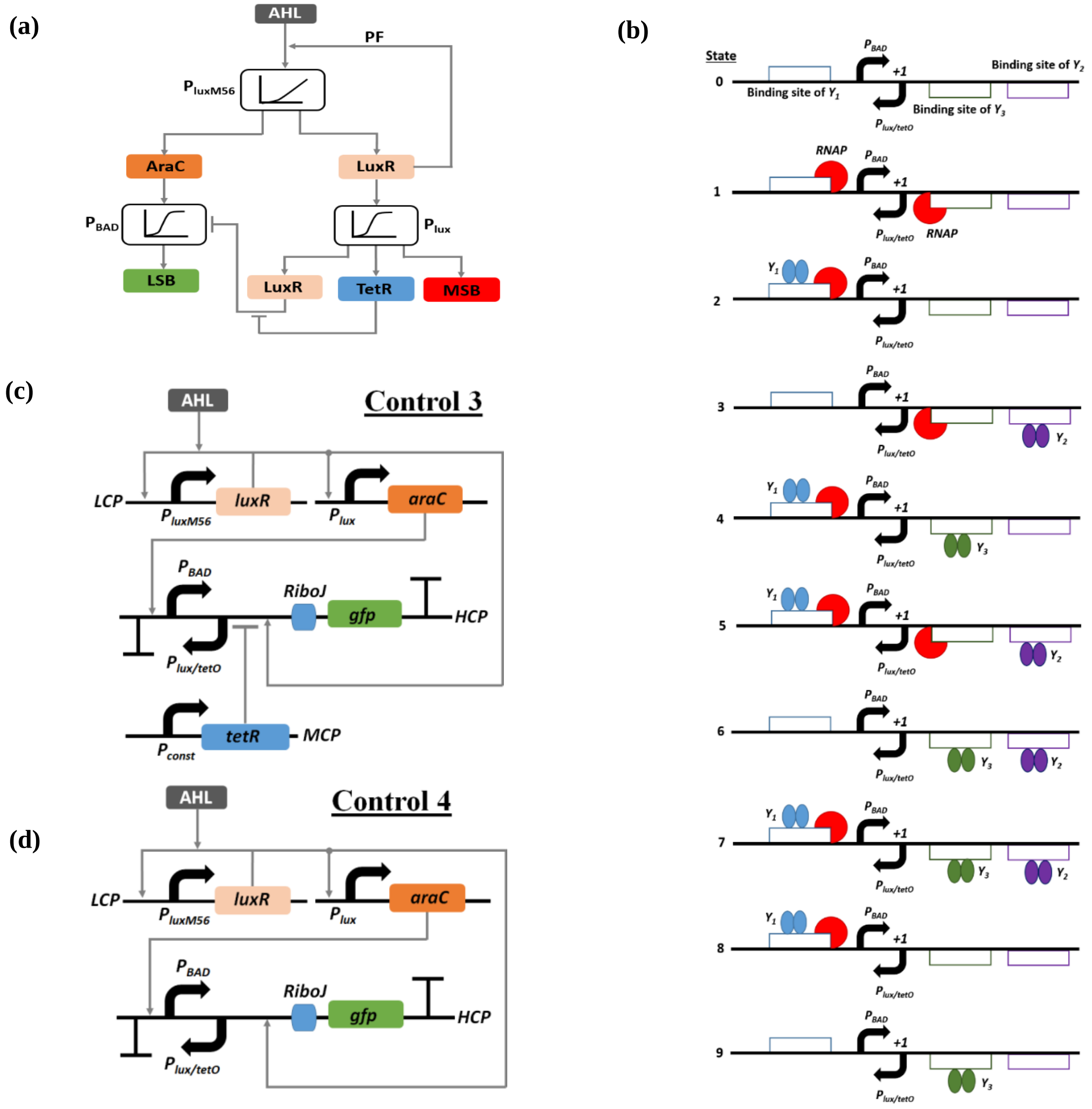

Supplementary Fig. 69. (a) A design of 2-bit ADC includes the TetR to inhibit the regulation on LSB by MSB for high AHL. **(b)** The binding states of forward P_{BAD} promoter and a combinatorial $P_{lux/tetO}$ promoter which is oriented in opposite direction and produces a transcriptional interference with P_{BAD}. **(c)** A control circuit consists of a graded PF that regulates P_{BAD} promoter and a combinatorial $P_{lux/tetO}$, while $TetR$ repressor is constitutively expressed. **(d)** A control circuit consists of a graded PF that regulates P_{BAD} promoter and a combinatorial $P_{lux/tetO}$, when there is no expression of TetR repressor.

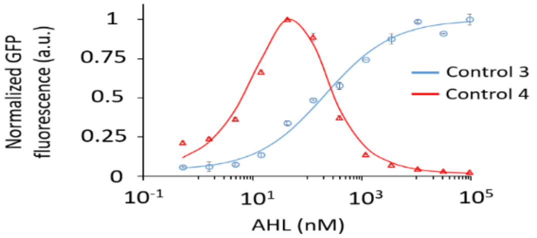

Supplementary Fig. 70. Experimental results of control circuits 3 and 4. Solid lines represent modelling results from the empirical models (Supplementary Eq. 7.42 and Eq. 7.43). We induced the circuits with Arabinose of 0.6 mM.

Based on our experimental control results, we modified the LSB circuit to allow TetR being dynamically regulated by P_{lux} promoter **(Supplementary Fig. 71a shows the construction of 2-bit ADC)**. Empirical models for the new circuit are given by:

$$MSB \propto \frac{\left(\frac{AHL}{K_1}\right)^{r_1}+\beta_1}{1+\left(\frac{AHL}{K_1}\right)^{r_1}} \tag{7.44}$$

$$LSB \propto \frac{\left(\frac{AHL}{K_0}\right)^{r_0}+\beta_0}{1+\left(\frac{AHL}{K_0}\right)^{r_0}+\alpha\cdot\left(\frac{MSB}{K_2}\right)^{r_2}}\cdot\left(\frac{1}{1+f(AHL)}\right)+\beta_2 \tag{7.45}$$

Where $f(AHL)$ represents the amount of GFP as a function as AHL. For a high concentration of TetR $f(AHL) \to 0$, and for a low concentration of TetR, Supplementary Eq. 7.45 tends to converge to Supplementary Eq. 7.43 therefore, an empirical model of $f(AHL)$ can be given by:

$$f(AHL) = \frac{LuxR-AHL}{1+TetR} \tag{7.46}$$

For simplicity, we assumed that $f(AHL) \propto MSB$, then:

$$LSB \propto \frac{\left(\frac{AHL}{K_0}\right)^{r_0}+\beta_0}{1+\left(\frac{AHL}{K_0}\right)^{r_0}+\alpha\cdot\left(\frac{MSB}{K_2}\right)^{r_2}}\cdot\left(\frac{1}{1+\alpha\cdot\left(\frac{MSB}{K_3}\right)^{r_3}}\right)+\beta_2 \tag{7.47}$$

Supplementary Fig. 71b shows the experimental results of the modified LSB circuit using the new design, when TetR is regulated by MSB. These results demonstrate that 2-bit ADC can be achieved using such a design (Supplementary Fig. 71c). The data of 2-bit ADC is well fitted by our empirical models (Eq. 7.44, Eq. 7.47), with a set of model parameters:

MSB: $r_1 = 1.5, K_1 = 500, \beta_1 = 0.015$

LSB: $r_0 = 1.45, K_0 = 20, \beta_0 = 0.03, \alpha = 300, r_2 = 1.2, K_2 = 1.5, r_3 = 1.3, K_3 = 85, \beta_2 = 0.04$

(a) **(b)** **(c)**

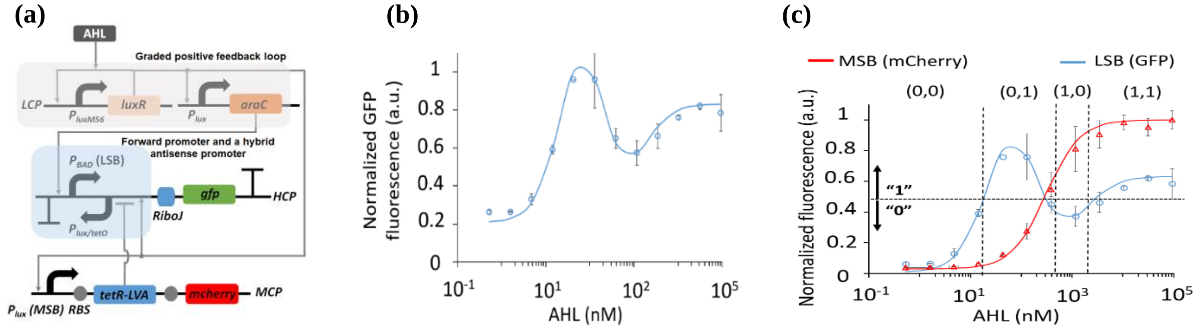

Supplementary Fig. 71. (a) Implementation of a 2-bit ADC, where the LSB circuit is modified to allow TetR to be regulated by MSB. **(b)** Experimental and simulation (Supplementary Eq. 7.47) results of the modified LSB circuit. Data are presented as mean values of median GFP ± SEM from independent replicates ($n = 3$). **(c)** The normalized GFP and mCherry signals of the 2-bit ADC. The signals referred to each basal level (The data was subtracted by the basal levels of GFP and mCherry). Solid lines indicate the modeling results of the empirical models (Supplementary Eq. 7.44 and Eq. 7.47). The horizontal dashed line, determined by the half of the fold change at the linear scale, separates between the "0" and "1". We induced the circuits with Arabinose of 0.4 mM. Data are presented as average ±standard deviations from independent replicates ($n = 3$).

7.2. Design II: Design and implementation of 2-bit log-ADC

ADC systems have significant applications in biotechnology and medicine [42], for example, ADC can be used to regulate several genes with all logic combinations using only a single inducer. To improve the performance of our 2-bit ADC, we combined principles of neural-network and pipelined ADC [43] design (Supplementary Fig. 72a). Pipelined ADC consists of several cascaded stages; every stage is built from comparators with each one has its own linear threshold [43]. In the new design, we added a third perceptgene which receives AHL and acts as comparator for very high levels of AHL concentration. In this case, we require that $B_2 > B_1$, and $n_2 \approx n_1$. The design of hybrid ADC is shown in Supplementary Fig. 72b. The LSB (GFP) signal is regulated by two parts: (1) a forward P_{BAD} promoter and

an reverse P_{lux} promoter, which is oriented in opposite direction to P_{BAD} (Supplementary Fig. 67a); (2) a quorum sensing P_{rhlR} promoter that interacts with AHL inducer (Supplementary Fig. 73) [1]. The MSB is implemented by a P_{lux} promoter and regulates mCherry. By fitting the activity of P_{rhlR} to Eq. 7.44, we obtained that $K_1 = 20 \times 10^3$ nM which is larger than K_1 (500 nM) of P_{lux}, satisfying $B_2 > B_1$.

The 2-bit hybrid ADC was constructed as shown in Supplementary Fig. 74. The circuit receives AHL as an analog input and contains a graded PF (P_{luxM56}) which regulates LuxR. The LSB circuit is built from two GFP signals: (1) Forward P_{BAD} promoter reverse P_{lux} promoter and (2) the P_{rhlR} promoter. The AraC regulated by P_{lux} is located on LCP (Supplementary Fig. 74a). We altered the binding efficiency of RNA polymerase [41] to ensure a low expression level of AraC. Meanwhile, the AraC and LuxR – AHL complex binds to the forward P_{BAD} and reverse P_{lux} promoter, respectively, which increases the threshold of P_{BAD} and decreases the expression level of GFP. The P_{rhlR} promoter located on HCP is activated by LuxR – AHLcomplex and regulates the GFP signal for high AHL concentrations. To achieve similar GFP levels in the two parts, an ssrA degradation tag [13] (LVA) was added on HCP. The P_{lux} of MSB circuit which is located on MCP, regulates the output mCherry signal. Supplementary Fig. 74b shows the experimental results of 2-bit hybrid ADC with distinct four logic states. The results show that there is a narrow region (marked in gray color) that the ADC is irregular behavior.

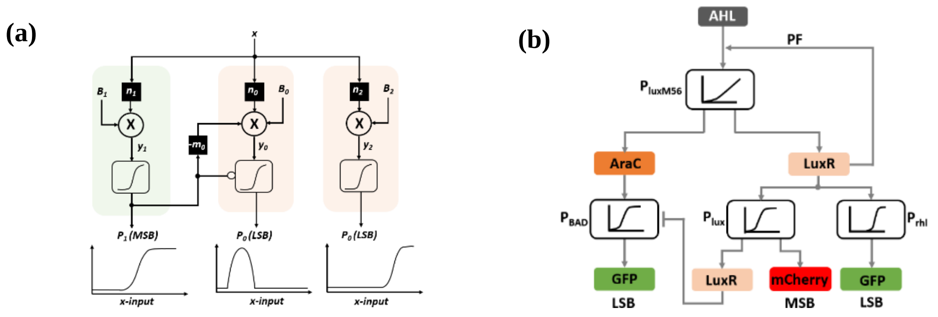

Supplementary Fig. 72. **(a)** A 2-bit hybrid ADC combined with neural networks and Pipelined ADC. **(b)** A 2-bit hybrid ADC using a third comparator, which activates P_{rhlR} promoter only for a high AHL concentration.

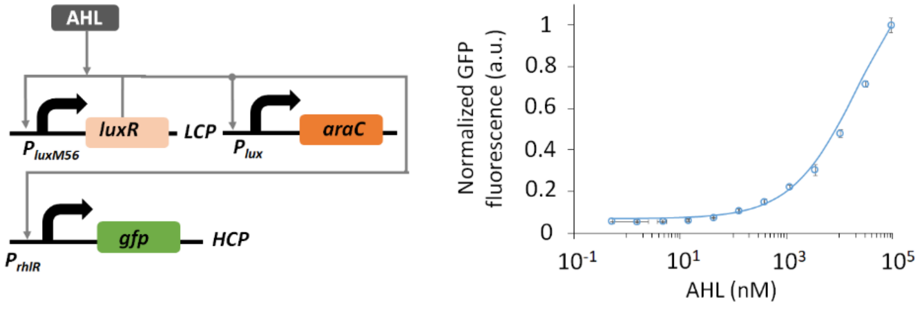

Supplementary Fig. 73. AHL -GFP transfer function of P_{rhlR} promoter. Solid line indicates the fitting results of the empirical models (Supplementary Eq. 7.44).

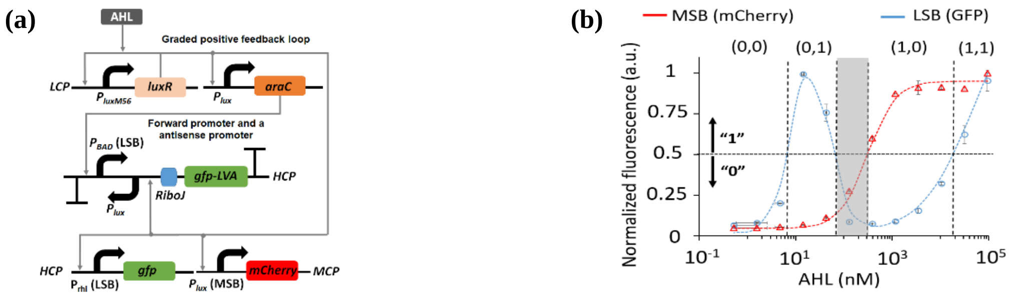

Supplementary Fig. 74. (a) Implementation of 2-bit hybrid ADC. **(b)** The normalized GFP and mCherry signals of 2-bit ADC. Solid lines indicate fitting to the empirical models (Supplementary Eq. 7.44, and Eq. 7.47). The horizontal dashed line, determined by the half of the fold change at the linear scale, separates between the "0" and "1". We induced the circuits with Arabinose of 0.06 mM. Data are presented as average ±standard deviations from independent replicates ($n = 3$).

7.3.Programmable a simple logic gates based on perceptgene

Our simulation models show that by changing the bias of a single perceptgene, we can achieve different logic gates. As was shown in Section 2, the perceptgene simulations consists of two parts:

1. The power-law and multiplication function: The simulations are based on Eq. 2.18, Eq. 2.19, and Eq. 2.20. Parameters that were used in simulations:

   Based on P_{lacO1} and P_{tetO} within ANF loop, and combinatorial promoter ($P_{lacO/tetO}$) – Fig. 1f

$$K_{m1} = 0.8, K_{m2} = 1, K_{d1} = 90, K_{d2} = 6, K_{d1h} = 45, K_{d2h} = 4, h_1 = 1, h_2 = 1.4, R_{max1} = 2000, R_{max2} = 3000, \boldsymbol{n_1} = \mathbf{1}, n_2 = 2, n_{1h} = 1, n_{2h} = 1, \theta = 1, \beta = 0.001$$

2. Activation function: here we used a similar activation function to P_{BAD}/ AraC (Eq. 2.21, Section 2.3), but without a repression element. We also assumed that there are several binding sites of AraC in the promoter P_{BAD}. Therefore, the P_{BAD} activation function can be described as:

$$P = \frac{\left(\frac{AraC_C}{K_{d3}}\right)^{n_3} + \beta_4}{1 + \beta_4 + \left(\frac{AraC_C}{K_{d3}}\right)^{n_3}} \qquad (7.48)$$

Where: $AraC_T = 30$, $K_{m3} = 0.09, \beta_4 = 0.002, n_3 = 1.5$,
For the OR gate, we used: $K_{d3} = 2$,
For the AND gate, we used: $K_{d3} = 30$,

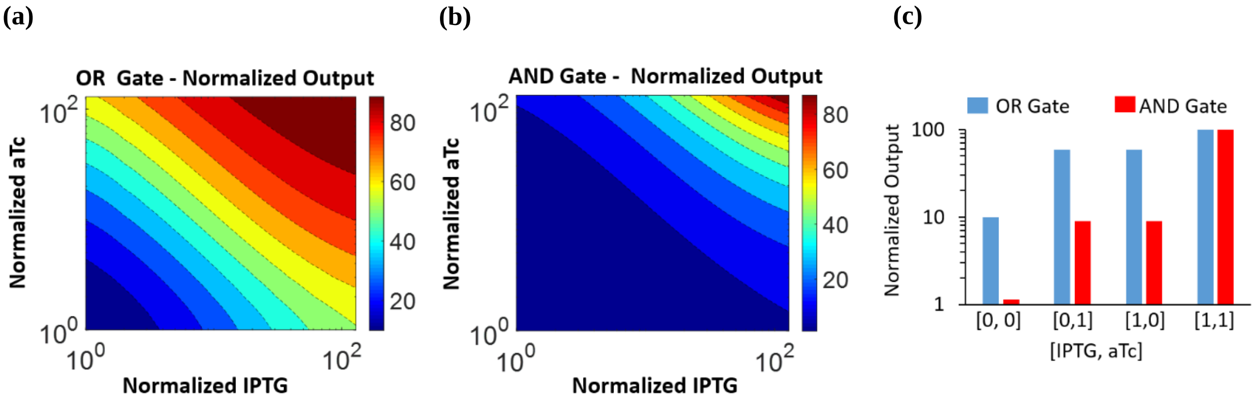

Supplementary Fig. 75. Simulation results of a single perceptgene for simple logic gates **(a)** OR logic gate: $K_{d3} = 2$, **(b)** AND logic gate $K_{d3} = 30$. **(c)** shows the data as logic states, "0" ➔ Normalized inducer level=1, "1" ➔ Normalized inducer level=128.

7.4 Design and implementation of ternary switch

From a dynamic system point of view, changing model parameters can lead to qualitatively different patterns of steady states. We therefore explored the model parameters (Eq. 7.47), by varying the ratio between repression and thresholding terms in Eq. 7.47 (K_2 Vs K_3), or by controlling the weights (Supplementary Fig. 63e). Interestingly we obtained a new behavior of the LSB circuit (Supplementary Fig. 76). We demonstrated this behavior by controlling different levels of Arabinose. The Arabinose concentration controls the repression as shown in Eq. 2.21, meaningly, In high Arabinose concentrations, the AraC acts only as an activator without repression. Because AraC is regulated by P_{lux}, we can assume that AraC ∝ MSB. The experimental resulted AHL -GFP transfer function using the new design is a three-valued logic (ternary logic) as shown in Supplementary Fig. 77. These results demonstrate that ternary was achieved using neural networks (Supplementary Fig. 63e). The data of ternary data converter is well fitted by our empirical models (Eq. 7.44 and Eq. 7.47), with a set of model parameters:

MSB: $r_1 = 1.5, K_1 = 450, \beta_1 = 0.005$

LSB: $r_0 = 1.6, K_0 = 28,\ \beta_0 = 0.02,\ \alpha = 200, r_2 = 1.2, K_2 = 1, r_3 = 1.4, K_3 = 300, \beta_2 = 0.02$

Ternary genetic circuits, converts analog signals to fuzzy levels, may find new applications in biotechnology, such as allowing engineers to tune the expression level of toxic proteins, enzymes in metabolic pathways in a reliable way. Furthermore, such systems can use it in building biosensors, which able to report in three states: low, medium and high. By contrast, the digital circuits can report only in two states.

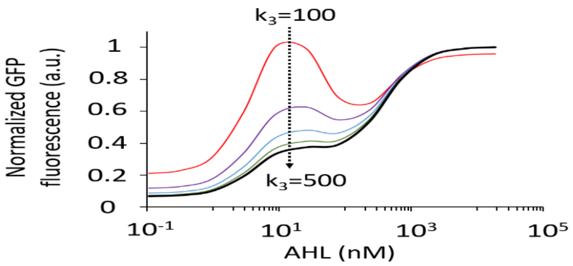

Supplementary Fig. 76. Simulation results of the influence of repression K_3 versus thresholding k_2 on LSB circuit. Modeling parameters: MSB: $r_1 = 1.5, K_1 = 450, \beta_1 = 0.005$, LSB: $r_0 = 1.6, K_0 = 28, \beta_0 = 0.03, \alpha = 160, r_2 = 1.2, K_2 = 1, r_3 = 1, \beta_2 = 0.04$.

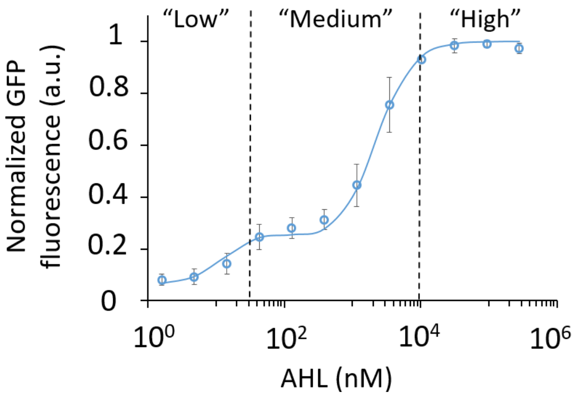

Supplementary Fig. 77. Implementation of ternary data converter, based on the regulation of repression versus thresholding. Experimental and modeling (Eq. 7.47) results of ternary circuit. Solid lines indicate modelling results of the empirical models (Eq. 7.44 and Eq. 7.47). We induced the circuits with high Arabinose of 50 mM compared to Supplementary Fig. 73 with 0.4 mM. Data are presented as average ±standard deviations from independent replicates ($n = 3$).

7.5. Reconfigurable perceptgene-based logic networks

To demonstrate the computational efficiency of perceptgene design, we modified the 3-input majority circuit by replacing the P_{tetO} promoter with $P_{luxM56/tetO}$ combinatorial promoter as shown in Supplementary Fig. 78a. The effect of AHL input on the GFP signal is collectively integrated by the $P_{luxM56/tetO}$ and $P_{luxM56/lacO1}$ prompters. Otherwise, the $P_{luxM56/tetO}$ combinatorial promoter acts as a logical conjunction operation rather than an integrative operation, which means it is active only if the AHL and IPTG are "1". The new network architecture allows AHL to exert more reliable effect on GFP by affecting both the AraC branch and the SupD branch of the network. The biophysical model that describes the new genetic circuit is based on the equation set Eq. 5.7-Eq. 5.11, with a modification of y_2:

$$y_1 = \left(B_1 \cdot \left(\frac{AHL}{K_{m1}}\right)^{n_1} \cdot \left(\frac{IPTG}{K_{m2}}\right)^{n_2}\right)^{m_1} \quad (7.49.1)$$

$$z_1 = \frac{y_1+\beta_1}{1+\beta_1+y_1} \quad (7.49.2)$$

$$y_2 = \left(B_2 \cdot \left(\frac{aTc}{K_{m3}}\right)^{n_3} \left(\frac{AHL}{K_{m1}}\right)^{n_1'}\right)^{m_2} \quad (7.49.3)$$

$$y_3 = (B_4 \cdot y_2 \cdot (B_3 \cdot z_1)^{m_3})^{m_4} \quad (7.49.4)$$

$$z_2 = \frac{y_3+\beta_2}{1+\beta_2+y_3} \quad (7.49.5)$$

Where $B_4 \equiv \frac{y_{m3}}{K_5} \cdot \frac{K_3}{K_4}$, $B_3 \equiv \frac{z_{m1}}{K_3}$, $B_2 \equiv \frac{y_{m2}}{K_2}$, $B_1 \equiv \frac{y}{a} \cdot {m_1}^b$, $B_5 \equiv B_4 \cdot {B_2}^{m_2} \cdot {B_3}^{m_3}$

Eq. 7.49.4 and Eq. 7.49.5 show that the network consists of two layers (Supplementary Fig. 78b). We used parameters consistent with the previous majority function model, except that $n_3 = 0.6$ (compared with $n_3 = 0.7$, because the effective Hill-coefficient of aTc on P_{tetO} is slightly different from $P_{lux/tetO}$ promoter). Our model accurately captures the behavior of our new circuit (Supplementary Fig. 78c). We used different Hill-coefficients of AHL -LuxR for $P_{lux/tetO}$ and $P_{lux/lacO}$; $n_1' = 0.65$, $n_1 = 1$. The results of the new circuit show that the [1,0,0] state gave a "1", by contrast to 3-input majority function that gave "0". Because the AHL was collectively integrated by $P_{luxM56/tetO}$ and $P_{luxM56/lacO1}$ prompters.

(a)

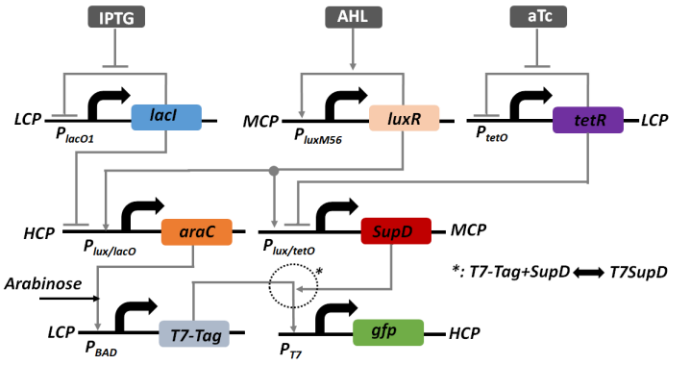

(b)

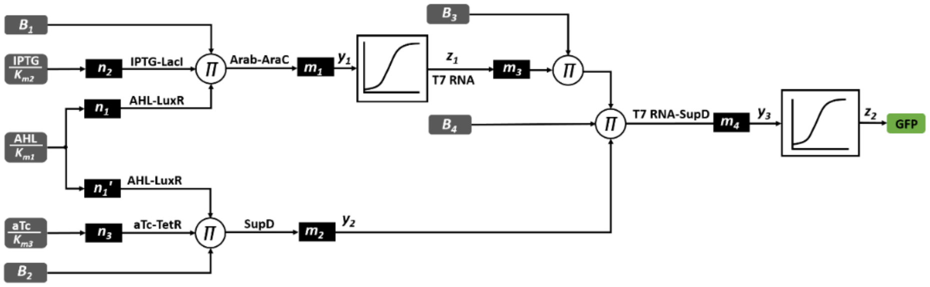

(c)

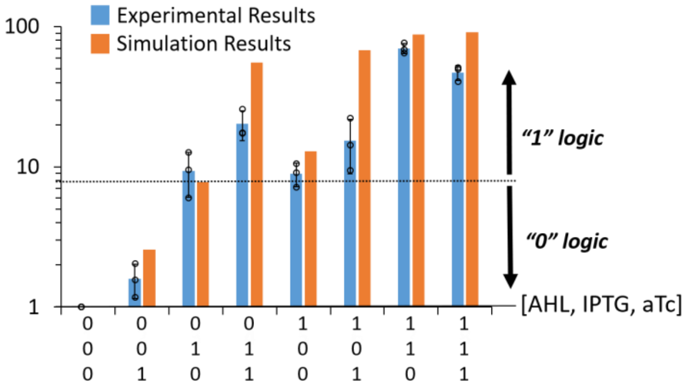

Supplementary Fig. 78. (a) Multilayer perceptgene displays a new logic function for three inputs (AHL, IPTG and aTc). **(b)** Abstract model of the new multilayer perceptgene network. **(c)** Experimental and simulation results. The horizontal dashed line, determined by the half of the fold change at the logarithmic scale, separates between the "0" and "1". Blue bars and their error bars show the average and standard deviation from independent replicates ($n = 3$). Median values of individual replicates are marked in circles.

7.6. Programmable perceptgene based on ExsA/ExsD protein sequestration:

(a)

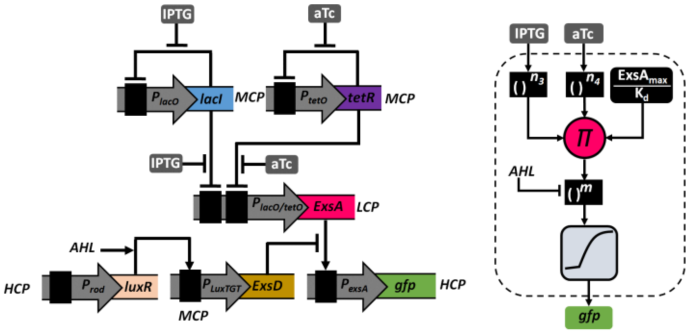

(b)

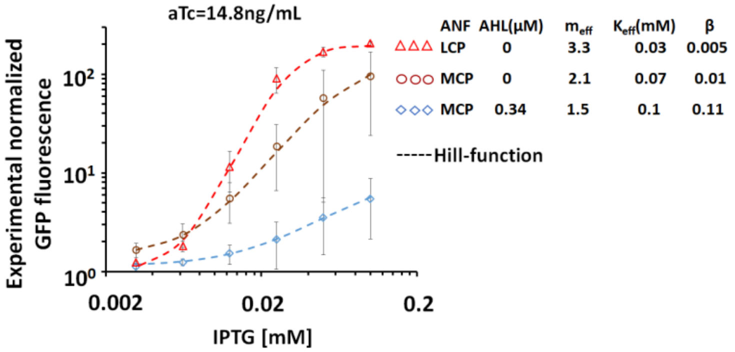

(c)

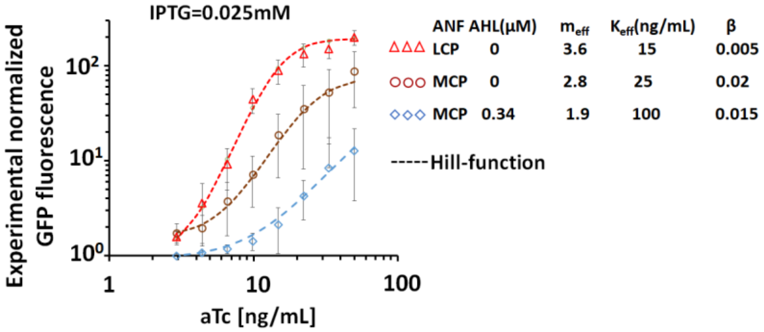

Supplementary Fig. 79. (a) Gene implementation for a programmable two-input perceptgene from Fig. 5d. The power-law and multiplication function circuit for inputs IPTG and aTc from Fig. 1b was connected with ExsA/P_{exs} system. The ExsD is controlled by AHL. In this circuit, the auto-negative feedback (ANF) loops for LacI and TetR are encoded on MCP. **(b)** Measured IPTG transfer function with aTc= 14.8ng/mL for three circuits: (1) ANF loops for LacI and TetR are encoded on a low-copy-number plasmid (LCP) and AHL=0, (2) ANF loops for LacI and TetR are encoded on a medium-copy-number plasmid (MCP and AHL=0, (3) ANF loops for LacI and TetR are encoded on a medium-copy-number plasmid (MCP and AHL=0.34μM. **(c)** Measured aTc transfer function with IPTG= 0.025mM for three circuits: (1) ANF loops for LacI and TetR are encoded on a low-copy-number plasmid (LCP) and AHL=0, (2) ANF loops for LacI and TetR are encoded on a medium-copy-number plasmid (MCP and AHL=0, (3) ANF loops for LacI and TetR are encoded on a medium-copy-number plasmid (MCP and AHL=0.34μM. The dotted lines are Hill-function fittings $\frac{\left(\frac{AHL}{K_{eff}}\right)^{m_{eff}}+\beta}{1+\left(\frac{AHL}{K_{eff}}\right)^{m_{eff}}}$ that are normalized to their minimum levels. Data are presented as average ±standard deviations from independent replicates ($n = 3$).

Supplementary Table 29 List of parameters used in this section

Symbol	Description
X	Input
Z_i	Output
y_i	Analog signals
n_i	Weights or Hill Coefficients
A_i	Biases
k_{f0}	The rates for the forward reactions from Z_0 to Z_0*
k_{f1}	The rates for the forward reactions from Z_1 to Z_1*
k_{r0}	The rates for the corresponding backward reactions Z_0* to Z_0
k_{r1}	The rates for the corresponding backward reactions Z_1* to Z_1

k_{10}	The regulation of Z_1* on the activation of Z_0
Z_{T0}	The total concentration of molecules Z_0
Z_{T1}	The total concentration of molecules Z_1
K_{n0}, K_{n1} *and* K_{m1}	Dissociation constants
P_i	The reaction activity is defined as ratio of the product and the total concentration of molecules
P	Probability that RNA polymerase is bound to the forward promoter at the equilibrium
β	Basal level
Y_i	The concentration of inducer-TF complex
K_{di}	Dissociation constants
θ	Interference
Arab	Free arabinose concentration
$AraC_T$	The total concentration of $AraC$
AHL	Free N-(β-Ketocaproyl)-L-homoserine Lactone $3OC_6HSL$ concentration
$LuxR_T$	The total concentration of $LuxR$
β_{eff}	Effective basal constant
K_{deff}	Effective dissociation constant
ρ	Fitting parameter
A	Activator
B	Transcription factor
r_i	Hill Coefficients
K_i	Effective dissociation constant
β_i	Promoter basal level
α	Fitting parameter

Supplementary Table 30 List of abbreviations used in this section

**Symbol**	**Description**
ADC	Analog to digital convertor
DAC	Digital to analog convertor
LSB	Last Significant Bit
MSB	Most Significant Bit
IDR	Input dynamic range
Arab	Free arabinose concentration
AHL	Free N-(β-Ketocaproyl)-L-homoserine Lactone $3OC_6HSL$ concentration
P_{BAD}	AraC promoter is activated by the $AraC$ when it is induced by arabinose (Arab)
P_{luxM56}	Mutated LuxR promoter is activated by the $LuxR$ when it is induced by AHL
P_{const}	Constitutive promoter
P_{lux}	LuxR promoter is activated by the $LuxR$ when it is induced by AHL
TetR	Concentration of TetR
$P_{lux/tetO}$	Combinatorial promoter
P_{rhlR}	Quorum sensing promoter that interacts with AHL inducer
LVA	ssrA degradation tag

8. Design principles of neuromorphic gene circuits

Weights and biases in neuromorphic circuits are determined by several factors, including Hill coefficients of small molecule inducers that serve as perceptgene inputs, the number and sequence of transcription factor binding sites, regulation of negative feedback strength (Figs.1 and 2 and Supplementary Fig. 82), regulation of incoherent feedforward strength (Supplementary Fig. 84), transcription factor sequestration via protein-protein interactions (Supplementary Fig. 85), transcription factors that competitively inhibit expression via steric hindrance (Supplementary Fig. 86), operator sequence that controls binding affinity of transcription factor in open loop and positive feedback (Supplementary Figs. 87-89), activation via RNA-protein interactions (Fig. 3), and protein structure (*e.g.,* dimerization and cooperativity), and circuit topology. Of particular importance, we demonstrate modulation of activation function weight m_1 for the majority function via administration of various Arabinose levels (Supplementary Fig. 54a, Figs. 3e and f). Specifically, we induce the system with eight different Arabinose concentrations and obtain fine-grain control of AraC-Arabinose weight, allowing continuous control of the system. The process of affecting weights and biases begins with a hypothesis of modulating the dosage response (e.g. transfer function) of a regulatory element. This is inspired by what has already been demonstrated in the literature, by a new approach that builds upon existing knowledge, or with completely new innovative methods. After implementing the circuit modifications, the new transfer functions are evaluated to determine the resultant weight and bias.

The first step toward the design of neuromorphic gene circuits is to understand the nature of the input molecules and determine their computing weights. In neuromorphic gene circuits, the computing weights of small molecules can be controlled by modifying the log domain slope of a regulated promoter's dosage response curve and can be characterized with Hill coefficients (e.g., the number of identical inducers that bind to transcription factors, and cooperativity of transcription factors). Regulatory topologies such as a negative feedback loop and an incoherent feedforward loop provide additional strategies that can be used to program the computing weights of small molecules and proteins. A mathematical model for the open-loop circuit, shown in Supplementary Fig. 80a, describes an input (In) that inhibits the activityof repressor R, which in turn represses the output. The production of R is constitutive and can be expressed as follows

$$Out = \frac{\alpha \cdot \tau}{1+\left(\frac{R}{K_d}\right)^n} \quad (8.1.1)$$

$$R = \frac{R_T}{1+\left(\frac{In}{K_m}\right)^h} \quad (8.1.2)$$

Where α is output production rate, τ is protein half-life, K_d is binding dissociation constant of the repressor (R) to the output, R_T is the total concentration level of R, K_m is binding dissociation constant of input (In) to R, n and m are Hill-coefficients of In and R. While the repressor R level is constant in the open-loop circuit, it is regulated by the output protein in the negative feedback circuit. A mathematical model for the auto-negative feedback loop circuit (Supplementary Fig. 80b) is given by:

$$Out_T = \frac{\alpha \cdot \tau}{1+\left(\frac{Out}{K_d}\right)^n} \quad (8.2.1)$$

$$Out = \frac{Out_T}{1+\left(\frac{In}{K_m}\right)^h} \quad (8.2.2)$$

Simulation results that compare the characteristic and computing weights of the open loop and auto-negative feedback circuits are shown in Supplementary Fig. 80c. By programming the strength of the auto-negative feedback loop, one can obtain fine grain control over the input weights (Supplementary Fig. 80d). In this work, we varied the number of binding sites for transcription factors in the promoter to control the strength of auto-negative feedback (Fig. 1).

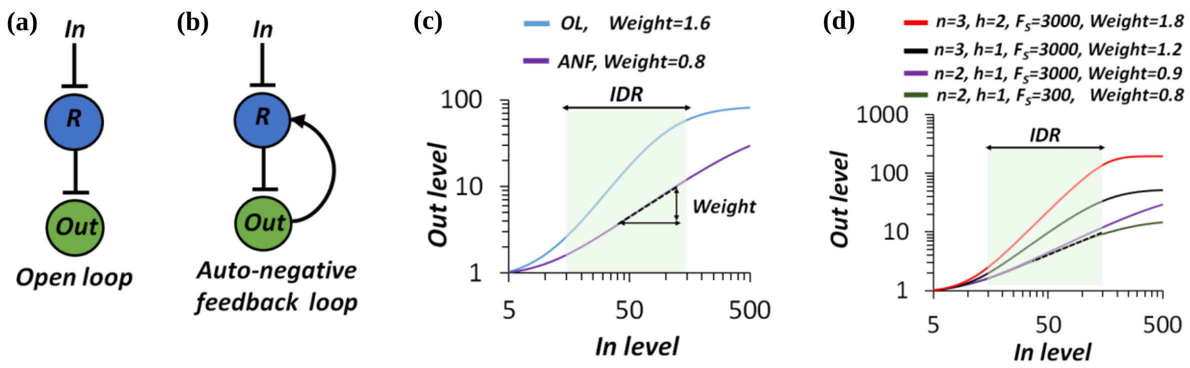

Supplementary Fig. 80. (a) Open loop design. **(b)** Auto-negative feedback design. **(c)** Simulation results for open loop and auto-negative feedback loop circuits. Simulation parameters: $K_m = 10, h = 2, K_d = 1, R_T = 100, \alpha \times \tau = 3000$. **(d)** Simulation for the auto-negative feedback circuit, where the feedback loop strength $F_s = \alpha \times \tau / K_d$.

In order to program the input weights continuously within a range, we split the auto-negative feedback loop into two reactions, one is the feedforward (R ➔ Out) loop and second is the negative feedback loop that is controlled by small molecule inducer x (A➔R). A mathematical model describing such a system is given by:

$$A_T = \frac{\alpha_1 \cdot \tau}{1 + \left(\frac{R}{K_{d1}}\right)^{n_1}} \tag{8.3.1}$$

$$R = \frac{R_T}{1 + \left(\frac{In}{K_{m1}}\right)^{h}} \tag{8.3.2}$$

$$R_T = \alpha_2 \cdot \tau \frac{\left(\frac{AX}{K_{d2}}\right)^{n_2}}{1 + \left(\frac{AX}{K_{d2}}\right)^{n_2}} \tag{8.3.3}$$

$$AX = A_T \frac{\left(\frac{x}{K_{m2}}\right)^{m}}{1 + \left(\frac{x}{K_{m2}}\right)^{m}} \tag{8.3.4}$$

The simulation results show that the level of inducer (x) can control the input weight by regulating the strength of the negative feedback (Supplementary Fig. 81).

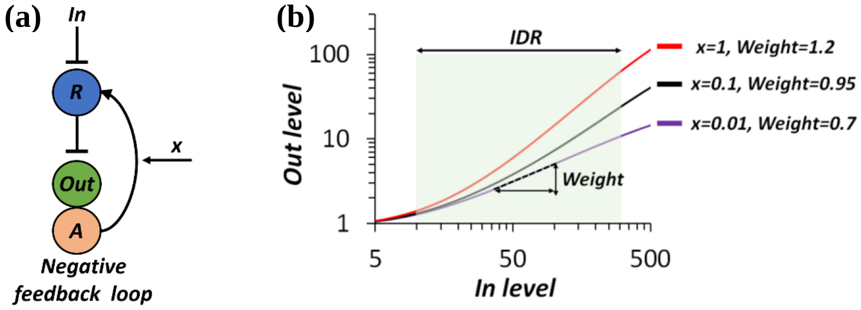

Supplementary Fig. 81. (a) Negative feedback design based on splitting the feedforward and feedback by using different proteins. **(b)** simulation results for the negative feedback loop. Simulation parameters: $K_{m1} = 10, h = 1.5, K_{d1} = 1, \alpha_2 \times \tau = 1000, n_1 = 1, K_{m2} = 1, m = 1.5, K_{d2} = 10, \alpha_1 \times \tau = 1000, n_2 = 1$.

In Supplementary Fig. 82 we describe small molecule control of negative feedback regulation via transcription factor activation of repressor. This design allows us to continuously program the weight of IPTG by changing the level of AHL. First, we compared the negative feedback circuit (Supplementary Fig. 82b) with an open-loop circuit (Supplementary Fig. 82c). The open loop circuit includes regulation of mCherry by P_{lacO} promoter that is induced by IPTG. While LacI is constitutively expressed in the open loop circuit, it is regulated by the P_{lux} promoter in the negative feedback circuit. Input IPTG regulates the activity of promoter P_{lacO} and expression of LuxR. The LuxR/AHL complex regulates LacI levels, which represses promoter P_{lacO}, creating a negative feedback loop. Supplementary Fig.

82d shows experimental results of open loop and negative loop circuits, in agreement with our theoretical results (Supplementary Fig. 80). Furthermore, this negative feedback loop design allows us to continuously program the weight of IPTG by changing the level of AHL (Supplementary Fig. 82e). The negative feedback loop strength, which is controlled by AHL, determines the IPTG input weight (Supplementary Fig. 82f). These experimental results are consistent with our simulation results (Supplementary Fig. 81).

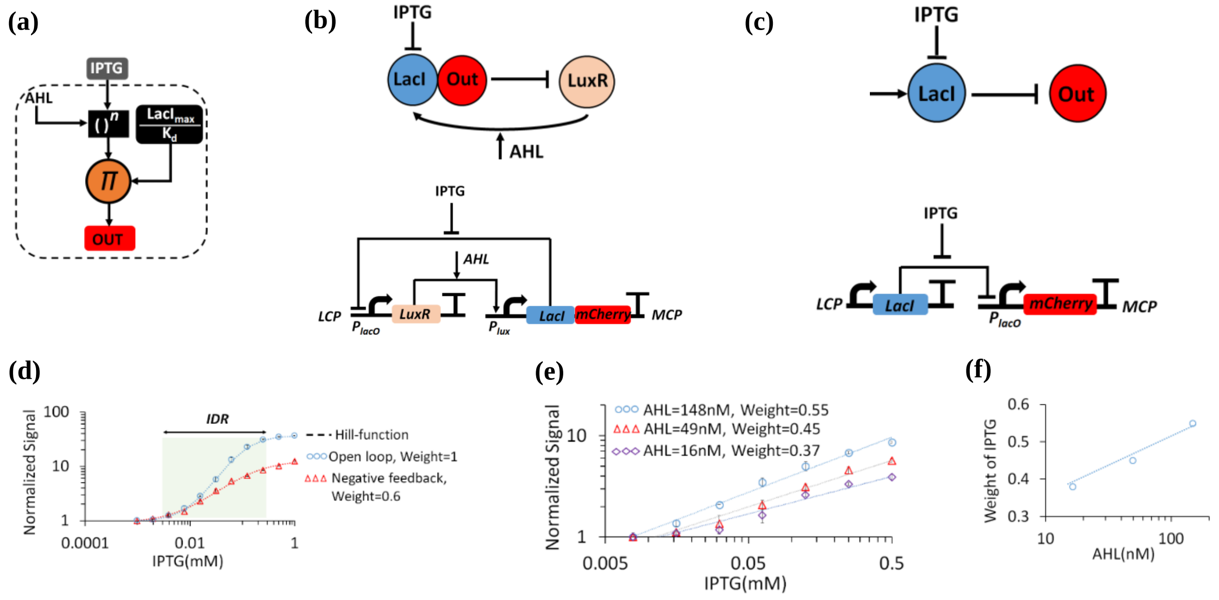

Supplementary Fig. 82. (a) Programmable perceptgene input weight. IPTG is the input and AHL regulates IPTG weight. **(b)** High level circuit diagram and genetic circuit implementation of programmable perceptgene input weight based on a negative feedback. AHL binds LuxR, forming a complex that controls the strength of the negative feedback loop. When IPTG binds LacI, it induces promoter P_{laco} activity, increasing LuxR levels. AHL binds LuxR, forming a complex that regulates expression of LacI. LuxR and GFP are regulated by P_{laco} promoter. P_{lux} is encoded on MCP, while P_{laco} is encoded on LCP. **(c)** High level circuit diagram and genetic circuit implementation of open loop circuit. **(d)** Experimentally measured IPTG/GFP transfer function of open loop and negative feedback circuits (AHL = 0.1 mM). **(e)** Experimentally measured IPTG/GFP transfer function under three different AHL concentrations. Data are presented as average ±standard deviations from independent replicates ($n = 3$). **(f)** IPTG input weight is shown as a function of AHL concentration.

Incoherent feedforward loops can also be used to program the weights of small molecules and proteins. In these networks, the upstream regulator (A) directly activates the target gene (Out) and indirectly represses it by activating repressor (R) of the target gene (Supplementary Fig. 83a). In our design, we assumed that the upstream regulator (A) is induced by the input (In). A mathematical model for such a system is given by:

$$InA = \alpha_1 \cdot \tau \frac{\left(\frac{In}{Km}\right)^h}{1+\left(\frac{In}{Km}\right)^h} \quad (8.4.1)$$

$$R_T = \alpha_2 \cdot \tau \cdot \frac{\left(\frac{InA}{Kar}\right)^n + \beta}{1+\left(\frac{InA}{Kar}\right)^n} \quad (8.4.2)$$

$$Out = \alpha_3 \cdot \tau \cdot \frac{\left(\frac{InA}{Ka}\right)^n + \beta}{1+\left(\frac{AX}{Ka}\right)^n} \cdot \frac{1}{1+\left(\frac{R}{Kr}\right)^m} \quad (8.4.3)$$

Where α_i are protein production rate, τ is protein half-life, K_a, K_{ar} and K_r are binding dissociation constants of the regulator (A) and repressor (R) to the output, K_m is binding dissociation constant of input (In) to A, n and m are Hill-

coefficients and β is the basal level. The simulation results of incoherent feedforward circuit are shown in Supplementary Fig. 83b. These results indicate finely tunable weight with positive and negative values.

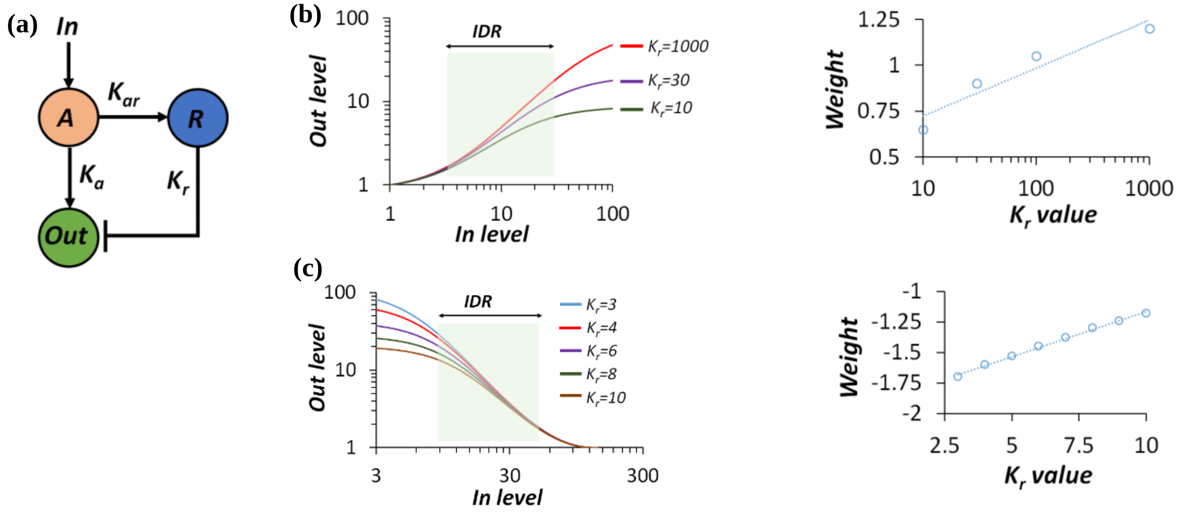

Supplementary Fig. 83. **(a)** Design of incoherent feedforward loop. **(b)** Design of programmable perceptgene with a positive weight, simulation results with parameters: $K_{m1} = 100, h = 1.5, K_a = 10, K_{ar} = 10, \alpha_1 \times \tau = 100, n = 1, \beta = 0.01, m = 1, \alpha_2 \times \tau = 100, K_r = 10, 30, 1000$ **(c)** Design of programmable perceptgene with negative weight, simulation results with parameters: $K_{m1} = 100, h = 1.5, K_a = 100, K_{ar} = 1, \alpha_1 \times \tau = 10, n = 1, \beta = 0.005, m = 1.5, \alpha_2 \times \tau = 150, K_r = 3 - 10$.

In Supplementary Fig. 84 we demonstrate experimentally small molecule control of transcription factor competitive inhibition via binding to an output promoter. This design allows us to modulate the weight of input AHL continuously by changing the aTc level (Supplementary Fig. 84e). The input AHL binds LuxR and forms a complex that induces expression of activator (AraC) and repressor (LacI), which combine to regulate GFP output, resulting in an incoherent feed-forward loop. Small molecule aTc controls LacI expression via de-repression of TetR, which in turn affects the overall AHL-GFP transfer function. We show the resulting input weight as a function of aTc relevant for the input dynamic range. Our incoherent feed-forward circuit provides negative weights.

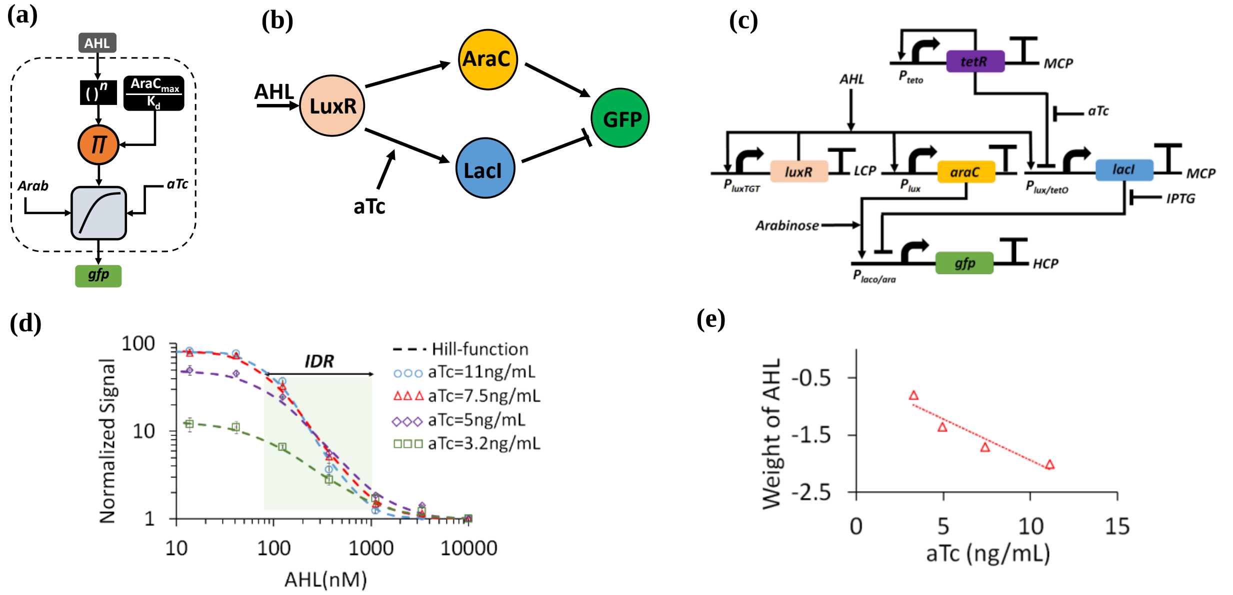

Supplementary Fig. 84. **(a)** A programmable percentgene with single input weight. aTc is used to modulate the weight of AHL continuously. **(b)** High level circuit design. Input AHL binds LuxR and forms a complex that regulates AraC and LacI. The AraC transcription factor activates GFP output expression, while the LacI transcription factor represses GFP expression. The

activation function is determined by the AraC/LacI interaction, where LacI expression is controlled by aTc, and hence impacts input weight. **(c)** Genetic circuit implementation. P_{tetO} promoter is regulated by TetR through an auto-negative feedback loop and induced by aTc. P_{TGT} and P_{lux} promoters are regulated by LuxR through a positive feedback loop and induced by AHL. AraC is regulated by P_{lux} promoter, and LacI is regulated by combinatorial $P_{lux/tetO}$ promoter. A ssrA degradation tag (LVA) was added to LacI to reduce the maximum protein level. GFP is regulated by AraC/LacI through activation/repression of combinatorial $P_{ara/lacO}$ promoter. The feedback loops in this circuit increase the input dynamic ranges of AHL and aTc. The P_{tetO} and combinatorial $P_{lux/tetO}$ promoters are encoded on a medium-copy-number plasmid (MCP). The combinatorial $P_{ara/lacO}$ promoter is encoded on a high-copy-number plasmid (HCP). The P_{lux} and P_{TGT} promoters are encoded on a low-copy-number plasmid (LCP). **(d)** Measured AHL - GFP transfer function where aTc is varied ($\text{aTc} = 11, 7.5, 5, 3.2\ ng/_{ml}$, $\text{Arabinose} = 50\text{ mM}, \text{IPTG} = 1\text{ mM}$). Data are presented as average ±standard deviations from independent replicates ($n = 3$). The dotted lines are Hill-function fitting with $GFP \propto \frac{\left(\frac{AHL}{K_{eff}}\right)^{h_{eff}} + \beta}{1 + \left(\frac{AHL}{K_{eff}}\right)^{h_{eff}}}$:

(1) $\text{aTc} = 11\ ng/_{ml},\ h_{eff} = 2.2,\ \ K_{eff} = 100\ mM,\ \beta = 0.013,$

(2) $\text{aTc} = 7.5\ ng/_{ml},\ h_{eff} = 1.9,\ \ K_{eff} = 85\ mM,\ \beta = 0.012,$

(3) $\text{aTc} = 5\ ng/_{ml},\ h_{eff} = 1.6,\ \ K_{eff} = 100\ mM,\ \beta = 0.02,$

(4) $\text{aTc} = 3.2\ ng/_{ml}, h_{eff} = 1.3,\ \ K_{eff} = 100\ mM,\ \beta = 0.08,$

(e) AHL weight based on the experimental results as a function of aTc .

The second step in the design of neuromorphic gene circuits is to aggregate the multiple inputs to one node in order to implement the multiplication function, which serves as a collective analog node. There are several biological mechanisms that can be used to accomplish such a function. For example, in this work combinatorial promoters ($P_{lacO/tetO}$, $P_{lux/lacO}$, $P_{lux/tetO}$) in Figs. 1 and 2, and mRNA-protein interaction in Fig. 3 was used to aggregate the analog weighted inputs and implement multiplication function. In Fig. 4, we showed that transcriptional interference can also be used to aggregate inputs acting as division with negative weights.

The third step in the design of neuromorphic gene circuits is to add an activation function that converts the analog pattern of the multiple inputs into a non-linear function for performing analog classification. This can be achieved by wiring the output of the multiplication circuit with an activator and promoter to regulate the perceptgene output.

In our neuromorphic genetic circuits, controlling the bias is perhaps easier than controlling the weights. The bias is determined by the ratio between the maximum protein expression level of the power-law/multiplication circuit output and the dissociation constant of transcription factor binding to DNA. The maximum protein expression level is determined by transcription rate, translation rate, mRNA and protein half-lives, and is given by:

$$\text{Bias} = \frac{\text{transcription rate} \times \text{mRNA half life} \times \text{translation rate} \times \text{protein half life}}{\text{dissociation constant of transcription factor binding to DNA}} \quad (8.5)$$

In this study, we use different methods to control the maximum protein level such as promoter strength, ribosome binding site strength, ssrA degradation tag and plasmid copy number. We now show experimentally that it is possible to readily increase the dissociation constant of a transcription factor by controlling expression of a second biological element that competitively inhibits the transcription factor. To this end, we use two biological systems: (1) dCas9 regulation (Supplementary Fig. 85), and (2) protein sequestration (Supplementary Fig. 86).

In Supplementary Fig. 85, we show competitive inhibition of gene activation via steric hindrance binding of DNA that is tuned by the DNA binding location of the dCas9/single guide RNA (sgRNA) complex. This design allows us to program bias continuously by choosing different sgRNA sequences. The dCas9 regulation system is built from two parts; a P_{BAD} promoter that activates the target gene by binding the Arabinose-AraC complex. The second part is the complex sgRNA-dCas9 which binds the AraC operator, and prevents the Arabinose-AraC complex from activating promoter P_{BAD}. The affinity of dCas9-sgRNA complex to its binding site and ability to sterically hinder transcription factor the promoter, control the binding dissociation constant of Arabinose-AraC complex to P_{BAD}. These factors, and hence perceptgene bias, can be readily controlled by building a library of sgRNA sequences (Supplementary Fig. 85d

and e). We used Hill-function (Supplementary Eq. 8.6) to estimate the effective dissociation constant of Arabinose (K_{eff}):

$$GFP \propto \frac{\left(\frac{In}{K_{eff}}\right)^{h_{eff}}}{1+\left(\frac{In}{K_{eff}}\right)^{h_{eff}}} + GFP_0 \qquad (8.6)$$

Where In is the Arabinose concentration. In order to evaluate the bias based on the changes of dissociation constant, we fit a model that includes induction and activation to our experimental results. Such model can be given by:

$$P = \frac{\left(\frac{InA}{K_d}\right)^n + \beta}{1+\left(\frac{InA}{K_d}\right)^n} \qquad (8.7.1)$$

$$InA = A_{max} \cdot \frac{\left(\frac{In}{K_m}\right)^h}{1+\left(\frac{In}{K_m}\right)^h} \qquad (8.7.2)$$

Where K_d is the binding dissociation constant of inducer-activator (Arabinose-AraC) complex (InA) to promoter, β is the promoter basal level, K_m is the binding dissociation constant of inducer (Arabinose) to activator (AraC), A_{max} is the activator maximum level achieved by promoter, and h is Hill-coefficient of inducer. In this simple model, the bias is defined as $B = A_{max}/K_d$.

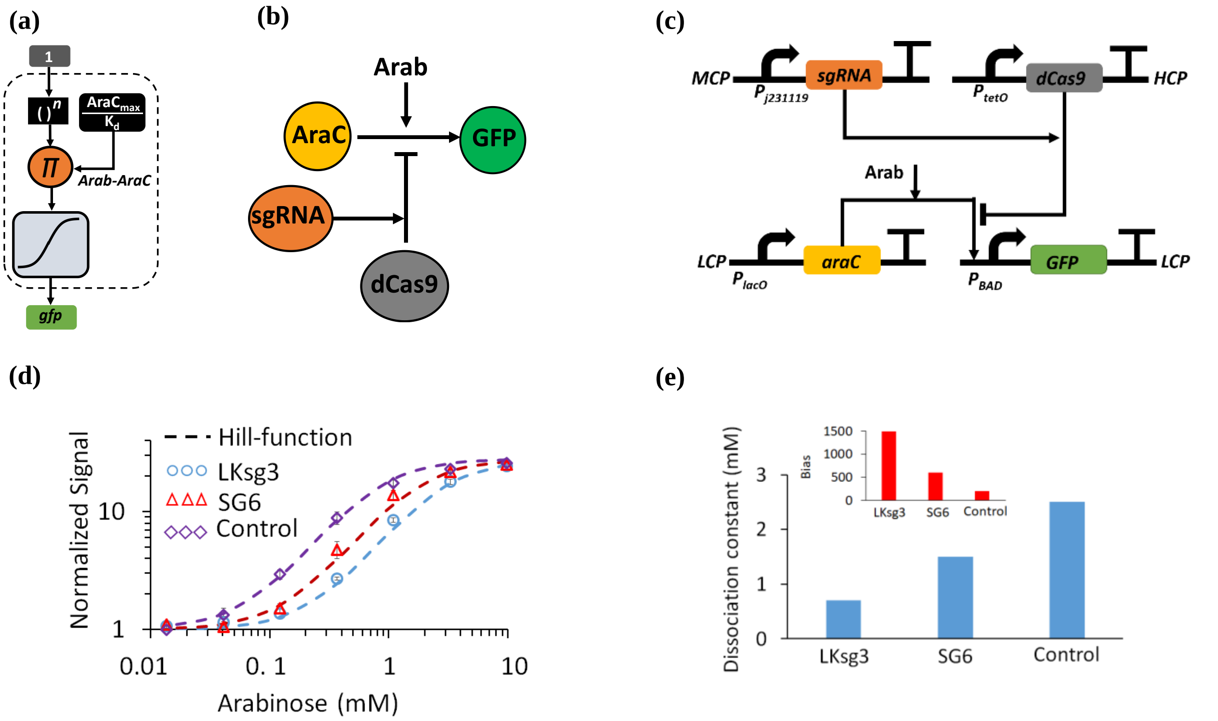

Supplementary Fig. 85. (a) A percentgene with a constant input value of 1, allowing analysis of the activation function's programmable bias. P_{BAD} promoter serves as the activation function, and the AraC/Arabinose complex is an analog signal that modulates bias. **(b)** High level circuit schematics. The design is based on competitive inhibition of gene expression via a tunable dCas9/sgRNA complex. The sgRNA sequence determines the affinity of dCas9/sgRNA binding to AraC operator, and hence can modulate bias by preventing AraC/Arabinose activation of P_{BAD}, which results in an increase of the AraC/Arabinose dissociation constant. **(c)** Genetic circuit implementation. AraC, dCas9 and sgRNA are constitutively expressed by P_{lacO}, P_{tetO} and $P_{J231119}$ promoters. P_{BAD} promoter is encoded on a low-copy-number plasmid (LCP), dCas9 is encoded on a high-copy-number plasmid (HCP), and sgRNA is encoded on a medium-copy-number plasmid (MCP). **(d)** Experimentally measured transfer functions for three circuit variants encoding two different sgRNA sequences and a control (purple; without sgRNA and dCas9). SG6 targets the middle of P_{BAD} promoter, while LKsg3's target is at the end of the promoter. SG6: GACGCTTTTTATCGCAACTC; LKsg3: TTTTTTTGGGCTAGCGAATT. The dotted lines are Hill-function fittings. $K_m = 90\ mM,\ h = 1.5,\ \beta = 0.035,\ n = 1$. **(e)** Arabinose

dissociation constant and bias (inset) for all three circuit variants. $Bias = AraC_{max}/K_d$, where K_d is the dissociation constant of AraC/Arabinose complex, $AraC_{max}$ is the maximum AraC produced.

The system ExsA/ExsD shwon in Fig. 5 can also be used to continuously program perceptgene bias. In the design in Supplementary Fig. 86, the Arabinose-AraC regulates the expression of ExsA that activates GFP output expression. aTc induces expression of anti-activator ExsD, which inhibits ExsA gene activation. Hence, the extent of the ExsA/ExsD protein-protein interaction and resultant perceptgene bias is controlled by aTc. We used Hill-function (Supplementary Eq. 8.6) to estimate the effective dissociation constant of Arabinose and set of equations Eq. 8.7.1-2 to estimate the bias. Comparing the two systems used in Fig. 5 and Supplementary Fig. 86, we can conclude that the ratio between the levels of ExsA and ExsD can determine if the system can be used to program the weight or the bias. When ExsA/ExsD>>1, changing the ExsD level yields significant changes in the bias, and when ExsA~ExsD, changing the ExsD level yields significant changes in the weight.

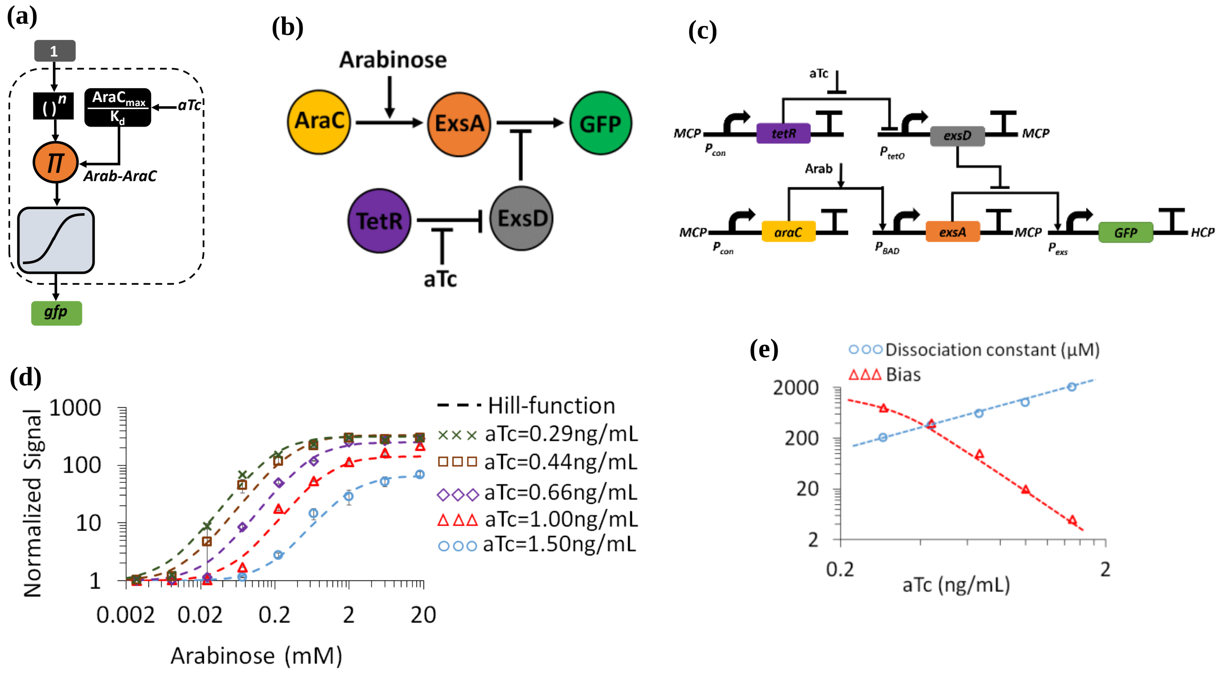

Supplementary Fig. 86. (a) A percentgene with a constant input value of 1, allowing analysis of the activation function's programmable bias. P_{exs} promoter serves as the activation function and the AraC/Arabinose complex is the analog signal. aTc level controls the activation function bias. **(b)** High level circuit diagram. The design is based on protein sequestration where ExsD shunts ExsA from activating GFP expression. This sequestration increases the dissociation constant of ExsA promoter binding and hence modulates bias. **(c)** Genetic circuit implementation. The ExsD- ExsA interaction that is used to regulate the activation function bias and is controlled via aTc. The AraC/Arabinose complex regulates expression of the ExsA activator. The TetR/aTc complex regulates expression level of anti-activator ExsD, which binds ExsA and inhibits its activation of P_{exs} promoter. AraC and TetR are constitutively expressed. P_{exs} promoter is encoded on HCP while the other promoters are encoded on MCP. **(d)** Experimentally measured Arabinose transfer functions under different aTc conditions. The dotted lines are Hill-function fittings, $h = 1.5,\ \beta = 0.035,\ n = 1$. **(e)** The Arabinose dissociation constant and relative bias (inset) as a function of aTc.

Introducing random mutations to operator sequence of transcription factor can also be used to control the weights and bias. Supplementary Fig. 87 describes modulations of transcription factor LuxR's DNA binding affinity via changes in Lux operator sequence. We introduced 7 random mutations into the first four nucleotides of the LuxR binding site. In order to precisely calculate the Hill-coefficient and effective dissociation constant, we simultaneously measured the activity of the P_{lux} promoters using GFP signal and measured the activity of the host cell using constitutive mCherry signal for each AHL level (Supplementary Fig. 87a). With our open loop circuit topology, we experimentally measured Hill-coefficient values ranging essentially continuously between 0.4 and 1 with a step of 0.1 (Supplementary

Figs. 87c and c). The AHL input weight is derived from the Hill-coefficient. Indeed, the input weight is the same as the Hill-coefficient when the basal level is very low, because both are equal to the slope at the log-log scale.

As we showed above, while the negative feedback can reduce the Hill-coefficient, here we show that positive feedback can increase the Hill-coefficient. This result matches other efforts that utilized auto-positive feedback (APF) to produce a sharp threshold in the response of inducer-promoter activity [44]. Supplementary Fig. 88 represent the experimental results of open and auto-positive feedback loops, from Supplementary Fig. 26 at the logarithmic scale. The Hill-coefficient values were doubled when auto-positive feedback was used compared to open loop. We then incorporated other six Lux operator mutants into a Lux response circuit with positive feedback regulation, and obtained Hill-coefficient values ranging between 1.1 and 2 and computed weights between 0.75 and 1.7 (Supplementary Fig. 89).

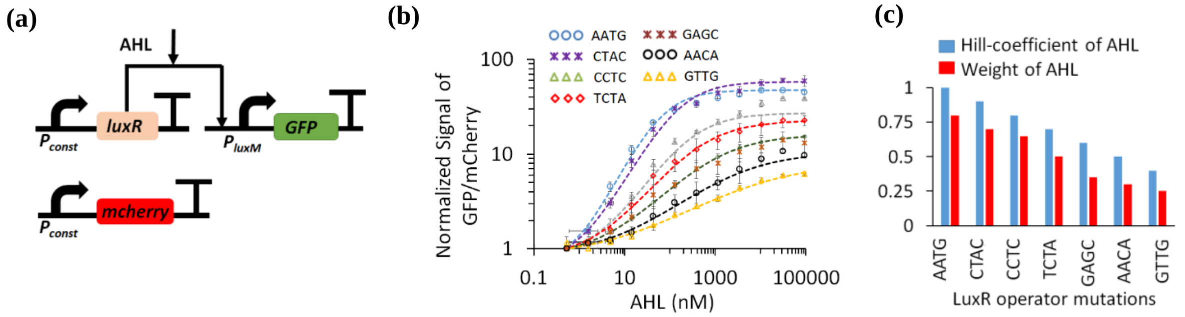

Supplementary Fig. 87. (a) Circuit design for open loop followed AHL induction. LuxR is constitutively produced. LuxR/AHL binds Lux operator mutants within P_{lux} promoter and activates GFP expression. **(b)** Experimentally measured AHL-GFP transfer functions of the Lux operator mutants used to determine Hill coefficients of the AHL input. Data are presented as average ±standard deviations from independent replicates ($n = 3$). **(c)** Experimental data shows that random mutations in the first four bases of the Lux operator result in an essentially continuous range of Hill-coefficients throw AHL input and AHL input weights. The dotted lines are Hill-function fittings.

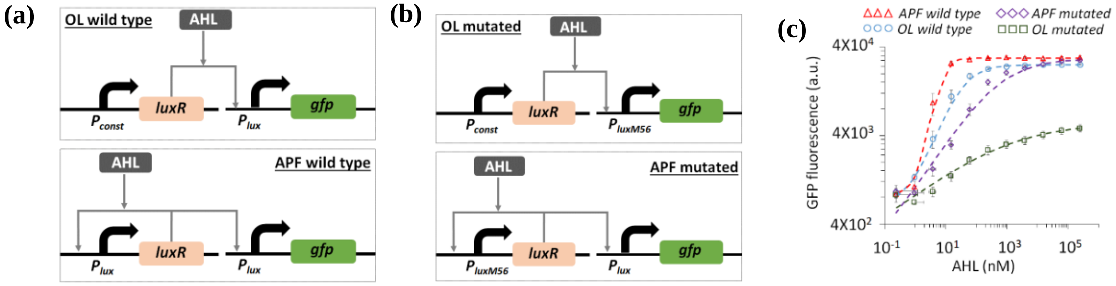

Supplementary Fig. 88. Hill coefficients for a modified circuit that encodes auto-positive feedback regulation (APF), where LuxR is expressed by the same mutated P_{lux} promoters. **(a)** The construction of open loop (OL) and APF circuits based on P_{lux} promoter. **(b)** The construction of OL and APF circuits based on mutated P_{lux} promoter (P_{luxM56}). **(c)** Measured transfer functions of multiple circuits. Data are presented as average ±standard deviations from independent replicates ($n = 3$). Dots are experimental data, and dashed-line is a Hill function fitting with the below parameters (See Supplementary Fig. 26):

OL circuit – Wild type P_{lux}: $K = 30, m_{eff} = 1, a = 25 \times 10^3, b = 600$

APF circuit – Wild type P_{lux}: $K = 7, m_{eff} = 2, a = 30 \times 10^3, b = 800$

OL circuit – Mutated P_{luxM56}: $K = 500, m_{eff} = 0.3, a = 5 \times 10^3, b = 100$

APF circuit – Mutated P_{luxM56}: $K = 500, m_{eff} = 0.5, a = 30 \times 10^3, b = 100$

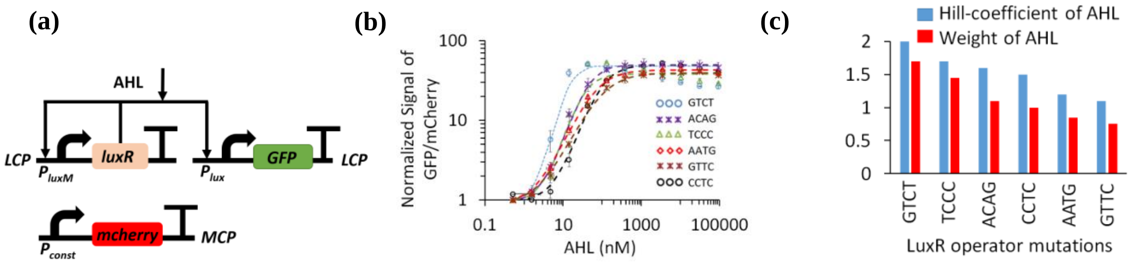

Supplementary Fig. 89. (a) Circuit design for positive feedback AHL. LuxR is regulated by the mutated $\mathrm{P_{luxM}}$ promoter. LuxR/AHL binds mutant Lux operators within promoter $\mathrm{P_{luxM}}$ and activates LuxR expression, and also LuxR/AHL binds wild type Lux operators within promoter $\mathrm{P_{lux}}$ and activates GFP expression. **(b)** Experimentally measured AHL-GFP transfer functions of the Lux operator mutants used to determine the AHL input Hill coefficients. Data are presented as average ±standard deviations from independent replicates ($n = 3$). **(c)** Experimental data shows that random mutations in the first four bases of the Lux operator, resulting in an essentially continuous range of AHL input Hill-coefficients and AHL input weights. The dotted lines are Hill-function fitting.

So far, we showed theoretically and experimentally that biological factors and design topologies determine weights and biases in our neuromorphic circuits. To provide a better view of design principles for neuromorphic gene circuits, we summarize and show below other examples (Supplementary Table 31). Remarkably, we conclude that the same biological mechanism can be used to tune the bias and also to implement power law circuit. The Supplementary Table 31 starts with auto-negative feedback (ANF) loops ($\mathrm{P_1}$, $\mathrm{P_2}$), a dual repression node ($\mathrm{P_{1/2}}$), and feedforward loop (P_z). The analog signal (Y*)* is represented by the activity of the dual repression node. The elements $\mathrm{P_1}$ and $\mathrm{P_2}$ are self-regulated and negatively induced by the inputs $\mathrm{In_1}$ and $\mathrm{In_2}$ respectively. The analog signal (Y) is combined with a nonlinear function to produce the output Z. This design has positive weights and is experimentally implemented using genetic components, as shown in Fig. 1. The design includes ANF loops consist of promoters that are regulated by repressors ($\mathrm{R_1}$, $\mathrm{R_2}$), (2) the inputs $\mathrm{In_1}$ and $\mathrm{In_2}$ are small molecules that inhibit the repressors activity, (3) the dual repression node is implemented by combinatorial promoter, and is also regulated by $\mathrm{R_1}$ and $\mathrm{R_2}$ and (4) the nonlinear activation function is realized by the regulation of the activator Y to P_z promoter. Our simulation results (Supplementary Table 31a) show that the output of the combinatorial promoter can be described by the analog pattern at the logarithmic domain ($log(Y) = n_1 \cdot log(In_1) + n_2 \cdot log(In_2) + Cons$), and the activator Y with P_z promoter convert this analog behavior to non-linear pattern with two states, asymptotically. Essentially, the circuit makes a decision based on collective interaction of transcription factors with analog behavior through their binding to a combinatorial promoter. Supplementary Table 31b shows our design to implement a perceptgene with negative weights. This design is slightly similar to the previous one, where $\mathrm{P_1}$ and $\mathrm{P_2}$are replaced by activation-repression (hybrid) nodes. Such nodes are directly activated by the inputs ($\mathrm{In_1}$ and $\mathrm{In_2}$) and self-repressed. We can implement the activation-repression nodes in living cells using combinatorial promoters that are regulated by activators and repressors. The inputs can be small molecules or transcription factors. Supplementary Table 31c shows our third design and it implements a perceptgene with negative and positive weights. This design is based on the previous design. Specifically, the dual repression node that regulates the collective analog signal (Y)was replaced by an activation-repression (hybrid) node. Perceptgenes can also be implemented by other biological mechanisms. For example, Supplementary Table 31d shows that a perceptgene with negative and positive weights was implemented by protein sequestration, where activator and anti-activator pair is involved. Other examples are shown in Supplementary Table 31e where a binding interaction between two sub-proteins can occur [41], and Supplementary Table 31f where phosphorylation and dephosphorylation reactions in two-component signaling system [45] are involved. Lastly, antisense transcription [36], which occurs counter to gene orientation, can also be applied to implement a power-law function with a positive and negative weights (Supplementary Table 31g).

Supplementary Tables 31. Examples for neuromorphic gene circuits.

**(a) Positive- weight perceptgene based dual repression system**				
**Schematic design**	**Schematic block**	**Genetic design**	**Mathematical model at steady state**	**Simulation results**
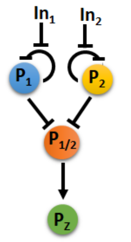	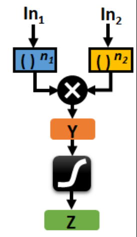	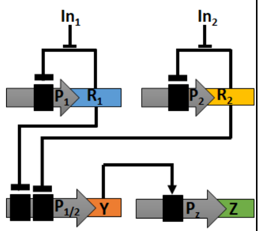	$R_i = \frac{R_{Ti}}{1+\left(\frac{In_i}{K_{mi}}\right)^{h_i}}$ $R_{Ti} = \frac{\alpha_R\cdot\tau}{1+\left(\frac{R_i}{K_{di}}\right)^{n_i}}$<br>$Y = \frac{\alpha_Y\cdot\tau}{1+\left(\frac{R_1}{K_{d1}}\right)^{n_1}+\left(\frac{R_2}{K_{d2}}\right)^{n_2}+\left(\frac{R_1}{K_{d1}}\right)^{n_1}\cdot\left(\frac{R_2}{K_{d2}}\right)^{n_2}}$<br>$Z = \frac{\alpha_Z\cdot\tau\cdot\left(\left(\frac{Y}{K_d}\right)^m+\beta\right)}{1+\left(\frac{Y}{K_d}\right)^m}$<br>$K_{mi}=1,\ h_i=1.5, \alpha_R\cdot\tau$=300, $K_{di}=1$,<br>$n_i=1,\ m=2.5,\ \alpha_Y\cdot\tau$=100, K_d=30, β =0.01	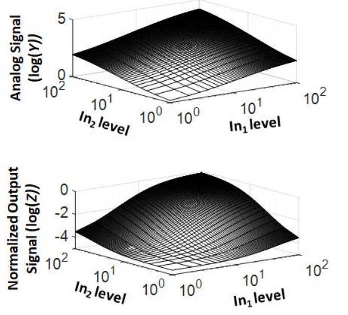

**(b) Negative-weight perceptgene based dual repression**				
**Schematic design**	**Schematic block**	**Genetic design**	**Mathematical model at steady state**	**Simulation results**
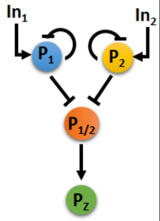	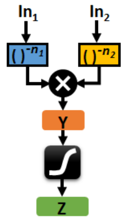	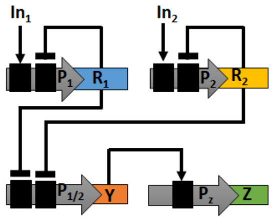	$R_i = \frac{\alpha_R\cdot\tau}{1+\left(\frac{R_i}{K_{di}}\right)^{n_i}}\cdot\frac{\left(\left(\frac{In_i}{K_{mi}}\right)^{h_i}+\beta_i\right)}{1+\left(\frac{In_i}{K_{mi}}\right)^{h_i}}$<br>$Y = \frac{\alpha_Y\cdot\tau}{1+\left(\frac{R_1}{K_{d1}}\right)^{n_1}+\left(\frac{R_2}{K_{d2}}\right)^{n_2}+\left(\frac{R_1}{K_{d1}}\right)^{n_1}\cdot\left(\frac{R_2}{K_{d2}}\right)^{n_2}}$<br>$Z = \frac{\alpha_Z\cdot\tau\cdot\left(\left(\frac{Y}{K_d}\right)^m+\beta\right)}{1+\left(\frac{Y}{K_d}\right)^m}$<br>$K_{mi}=100,\ h_i=1.5, \alpha_R\cdot\tau$=300, $K_{di}=1$,<br>$n_i=1,\ m=2.5,\ \alpha_Y\cdot\tau$=100, K_d=30, β =0.01	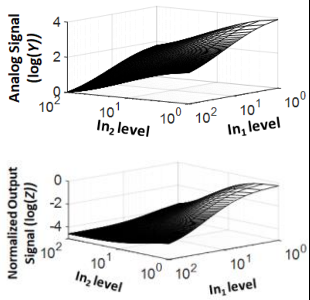

**(c) Perceptgene based hybrid activation-repression system**				
**Schematic design**	**Schematic block**	**Genetic design**	**Mathematical model at steady state**	**Simulation results**
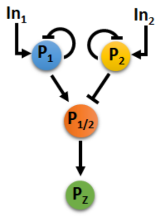	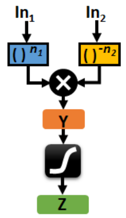	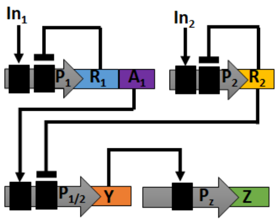	$R_i = \frac{\alpha_R\cdot\tau}{1+\left(\frac{R_i}{K_{di}}\right)^{n_i}}\cdot\frac{\left(\left(\frac{In_i}{K_{mi}}\right)^{h_i}+\beta_i\right)}{1+\left(\frac{In_i}{K_{mi}}\right)^{h_i}}$ $A_1 = g_1\cdot R_1$<br>$Y = \frac{\alpha_Y\cdot\tau\cdot\left(\left(\frac{A_1}{K_{da1}}\right)^{n_1}+\beta\right)}{1+\left(\frac{A_1}{K_{da1}}\right)^{n_1}+\left(\frac{R_2}{K_{d2}}\right)^{n_2}+\left(\frac{A_1}{K_{da1}}\right)^{n_1}\cdot\left(\frac{R_2}{K_{d2}}\right)^{n_2}}$<br>$Z = \frac{\alpha_Z\cdot\tau\cdot\left(\left(\frac{Y}{K_d}\right)^m+\beta\right)}{1+\left(\frac{Y}{K_d}\right)^m}$<br>$K_{mi}=100,\ h_i=1.5, \alpha_R\cdot\tau$=300, g_1=1, $K_{di}=1$<br>$n_i=1,\ m=2.5,\ \alpha_Y\cdot\tau$=200, K_{da1}=5, K_d=30,<br>β =0.01	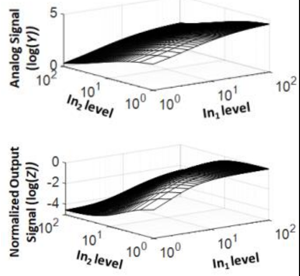

(d) Perceptgene based protein sequestration system

Schematic design	Schematic block	Genetic design	Mathematical model at steady state	Simulation results
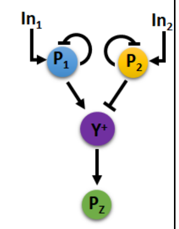	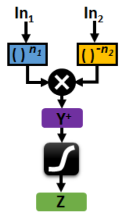	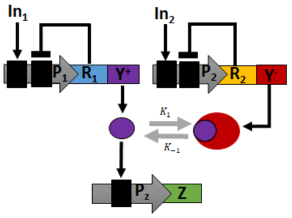	$R_i = \frac{\alpha_R \cdot \tau}{1+\left(\frac{R_i}{K_{di}}\right)^{n_i}} \cdot \frac{\left(\left(\frac{In_i}{K_{mi}}\right)^{h_i}+\beta_i\right)}{1+\left(\frac{In_i}{K_{mi}}\right)^{h_i}}$ $Y_T{}^+ = g_1 \cdot R_1$ $Y_T{}^- = g_2 \cdot R_2$<br>$C = \frac{K_{mc}+Y_T{}^+ + Y_T{}^-}{2} - \frac{\sqrt{(K_{mc}+Y_T{}^+ + Y_T{}^-)^2 - 4 \cdot Y_T{}^+ \cdot Y_T{}^-}}{2}$<br>$Y^+ = Y_T{}^+ - C$ $K_{mc} = K_{-1}/K_1$<br>$Z = \frac{\alpha_Z \cdot \tau \cdot \left(\left(\frac{Y^+}{K_d}\right)^m + \beta\right)}{1+\left(\frac{Y^+}{K_d}\right)^m}$<br>$K_{mi} = 100$, $h_i = 1.5$, $\alpha_R \cdot \tau = 300$, $g_1 = 1$, $g_2 = 15$ $K_{di} = 1$, $n_i = 1$, $K_{mc} = 10$, $m = 2.5$, $K_d = 3$, $\beta = 0.01$	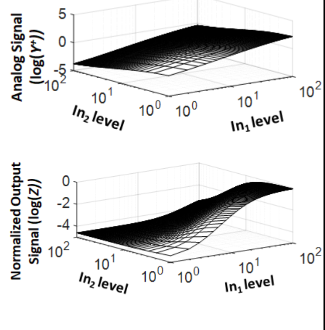

(e) Perceptgene based fusion protein system

Schematic design	Schematic block	Genetic design	Mathematical model at steady state	Simulation results
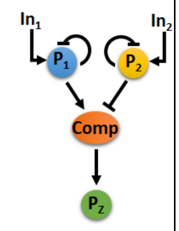	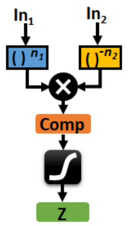	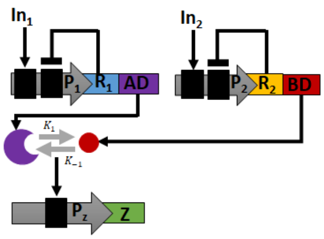	$R_i = \frac{\alpha_R \cdot \tau}{1+\left(\frac{R_i}{K_{di}}\right)^{n_i}} \cdot \frac{\left(\left(\frac{In_i}{K_{mi}}\right)^{h_i}+\beta_i\right)}{1+\left(\frac{In_i}{K_{mi}}\right)^{h_i}}$<br>$AD_T = g_1 \cdot R_1$ $BD_T = g_2 \cdot R_2$<br>$Comp = \frac{K_m + AD_T + BD_T}{2} - \frac{\sqrt{(K_{mc}+AD_T+BD_T)^2 - 4 \cdot AD_T}}{2}$<br>$K_{mc} = K_{-1}/K_1$<br>$Z = \frac{\alpha_Z \cdot \tau \cdot \left(\left(\frac{C}{K_d}\right)^m + \beta\right)}{1+\left(\frac{C}{K_d}\right)^m}$<br>$K_{mi} = 100$, $h_i = 1.5$, $\alpha_R \cdot \tau = 300$, $g_1 = 1$, $g_2 = 1$, $K_{di} = 1$, $n_i = 1$, $K_{mc} = 10$, $m = 2.5$, $K_d = 3$, $\beta = 0.01$	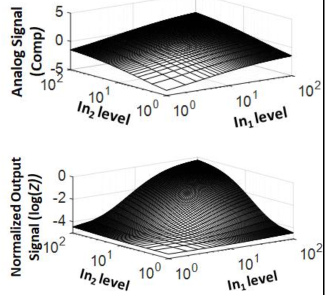

(f) Perceptgene based two-component regulatory system

Schematic design	Schematic block	Genetic design	Mathematical model at steady state	Simulation results
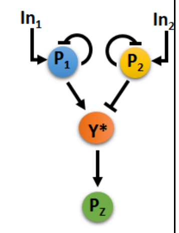	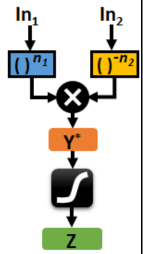	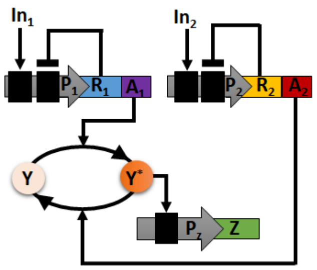	$R_i = \frac{\alpha_R \cdot \tau}{1+\left(\frac{R_i}{K_{di}}\right)^{n_i}} \cdot \frac{\left(\left(\frac{In_i}{K_{mi}}\right)^{h_i}+\beta_i\right)}{1+\left(\frac{In_i}{K_{mi}}\right)^{h_i}}$<br>$A_i = g_i \cdot R_i$<br>$Y^* = Y_T \frac{\frac{A_1}{A_2 \cdot K_m}}{\frac{A_1}{A_2 \cdot K_m}+1}$<br>$K_{mc} = K_{-1}/K_1$<br>$Z = \frac{\alpha_Z \cdot \tau \cdot \left(\left(\frac{Y^*}{K_d}\right)^m + \beta\right)}{1+\left(\frac{Y^*}{K_d}\right)^m}$<br>$K_{mi} = 100$, $h_i = 1.5$, $\alpha_R \cdot \tau = 300$, $g_1 = 1$, $g_2 = 1$, $K_{di} = 1$, $n_i = 1$, $Y_T = 100$, $K_{mc} = 10$, $m = 2.5$, $K_d = 30$, $\beta = 0.01$	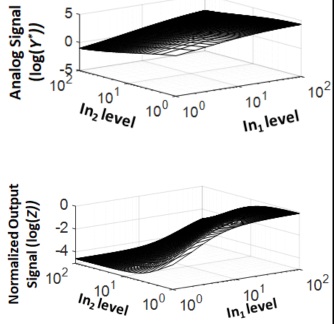

<table>
<tr><th colspan="5">(g) Perceptgene based antisense transcriptional regulatory system</th></tr>
<tr><th>Schematic design</th><th>Schematic block</th><th>Genetic design</th><th>Mathematical model at steady state</th><th>Simulation results</th></tr>
<tr>
<td>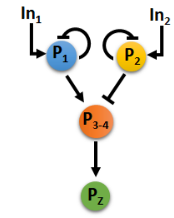</td>
<td>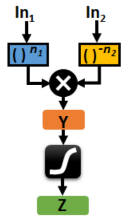</td>
<td>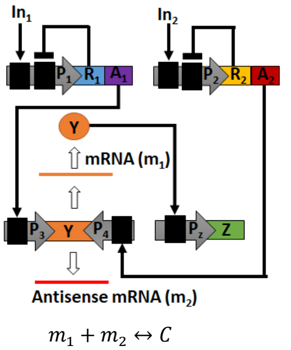</td>
<td>$R_i = \frac{\alpha_R \cdot \tau}{1+\left(\frac{R_i}{K_{di}}\right)^{n_i}} \cdot \frac{\left(\left(\frac{In_i}{K_{mi}}\right)^{h_i}+\beta_i\right)}{1+\left(\frac{In_i}{K_{mi}}\right)^{h_i}}$
$m_{Ti} = g_i \cdot R_i$
$C = \frac{(m_{T1}+m_{T2}+K_{mc})}{2} - \frac{\sqrt{(m_{T1}+m_{T2}+K_{mc})^2 - 4 \cdot m_{T1} \cdot m_{T2}}}{2}$
$Y = m_{T1} - C$
$Z = \frac{\alpha_Z \cdot \tau \cdot \left(\left(\frac{Y}{K_d}\right)^m + \beta\right)}{1+\left(\frac{Y}{K_d}\right)^m}$
$K_{mi} = 100$, $h_i = 1.5$, $\alpha_R \cdot \tau$=300, g_1=1, g_2=1, $K_{di} = 1$, $n_i = 1$, $K_{mc} = 10$, $m = 2.5$, K_d=3, β =0.01</td>
<td>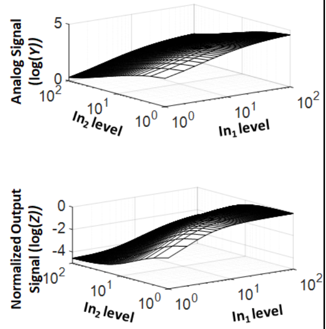</td>
</tr>
</table>

Supplementary Tables 31. Examples for neuromorphic gene circuits. The schematic design describes the regulatory elements, the schematic block shows the mathematical operations, the genetic design shows the genetic implementation and biological regulatory components.

In addition to our own experimental data and simulation results, previous articles have also demonstrated the ability to modulate various properties of engineered gene circuits that are relevant to our neuromorphic circuit engineering efforts. The engineered libraries of genetic device variants described briefly below could be used in our neuromorphic approach to obtain essentially continuous modulation of weights and biases:

- The Ribosome Binding Site Calculator is a tool that predicts the binding affinity of a Ribosome and synthetic binding sites in *Escherichia coli*. It enables rational control over transcription factor expression levels [46]. This tool can be useful for programming perceptgene bias, which is directly affected by the translation rate.

- The RNA polymerase Binding Site Calculator is a tool that predicts the binding affinity of an RNA polymerase and synthetic promoters in *Escherichia coli*. It enables rational control over transcription factor expression levels [47]. This tool can be useful for programming perceptgene bias, which is directly affected by the translation rate.

- The Anderson synthetic promoter library includes more than 30 characterized promoters with variable strength of approximately 100 fold between the weakest and strongest (http://parts.igem.org/Promoters/Catalog/Anderson). This tool can also be useful for programming bias, which is directly affected by the transcription rate. Another synthetic promoter library was also published around the same time [48].
- The Weiss TALER library includes 26 programmed transcriptional repressors that bind synthetic combinatorial promoters in mammalian cells [49]. With TALE modular protein construction, any DNA sequence can be targeted, leading to an essentially limitless design search space (with a usable length of anywhere between 14 and 26 DNA bases for TALER binding). The library elements have an approximately 2 orders of magnitude difference in repression folds from around 20 to greater than 10^3 leading to different Hill coefficients and hence different input weights. Promoter engineering by inclusion of two versus four TALER binding sites increased fold repression by five and ten fold for TALER21 and TALER14 respectively.
- The Voigt repressor library includes 16 orthogonal TetR-family repressors and their cognate promoters. Each repressor/promoter pair's transfer function has been characterized. The measured Hill coefficients range between 1.5 and 6.5, with fold changes between 1-2 orders of magnitudes [50]. This tool can be useful for programming input weights.

- A recent effort in *Escherichia coli* has demonstrated several inducible synthetic promoters with varying ligand-promoter activity transfer functions. The synthetic promoters are regulated by TtgR, PmeR and NalC and are induced by phloretin, Naringenin, and PCP, receptively [51]. This tool can be useful for programming input weights similar to Supplementary Fig. 87.
- The Riboswitch Binding Sequence Calculator predicts ligand induced gene activation of riboswitch sequences using a physics based model. Then, computational design with this tool is used to create a library of 62 different synthetic riboswitches with activation fold of up to 383x [52]. This tool can be useful for programming weight and bias.
- A library of LuxR transcription factors were developed [53]. AHL-dependent transcriptional activation can be selected to meet design specifications. This tool can be useful for programming input weight similar to Supplementary Fig. 87.
- A library with 238 member of tunable control for protein degradation in bacteria were developed [54]. This tool can be useful for programming bias, which is directly affected by the protein half-life.
- A library of antisense constitutive promoters was developed [36] . Every member of the library includes a target gene that is regulated by a repressor and by another promoter that is oriented opposite to the target gene. The library includes 5,668 terminator–promoter combinations that was used to control the expression of three repressors (PhlF, SrpR, and TarA). Such design can be used reliably to tune gene expression level and control small molecules' dissociation constant. This tool can be useful for programming bias.

Other methods to alter the dosage response curves of genetic regulation elements have also been published, and these could also be used to modulate weight and bias in neuromorphic circuits:

- Landry *et al.* 2018 developed a two-component signaling system that can dynamically tune the dissociation constant of small molecules. This system can be used to control bias [55].
- Segall-Shapiro *et al.* 2014 split T7 proteins into several parts and changed cooperativity. This method can be used to control the weights for inputs and activation functions [56].
- Morel *et al.* 2016 introduced extra binding sites into promoters and changed cooperativity. This method can be used to control input weight [57].

We also analyzed the properties of common synthetic biological parts, including weights for some of the parts used in this manuscript (Supplementary Fig. 90a) and Hill coefficients for devices that were previously published (Supplementary Fig. 90b) providing another source of parts with desired weights and Hill-coefficients for small molecules and transcription factors.

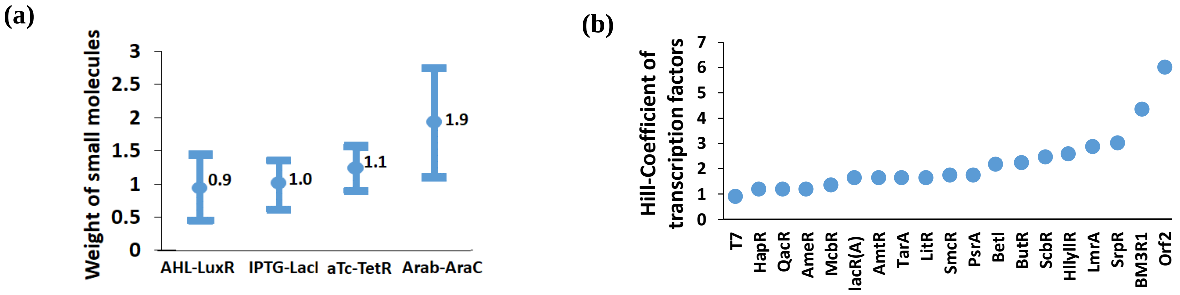

Supplementary Fig. 90. (a) Hill coefficient values of small molecules that are used in this study. **(b)** Hill coefficient values of transcription factors that are used in Stanton et al. 2014 [50].

The process of fine-tuning design parameters of analog/digital/neuromorphic circuits begins with a hypothesis of how to modulate the dosage response (e.g. transfer function) of a regulatory element. This can be inspired by what has already been demonstrated in the literature, by a new approach that builds upon existing knowledge, or with completely new innovative methods. After implementing the circuit modifications, the new transfer functions are evaluated to determine the resultant design parameters.

In summary, there are many methods to control the weights and biases, these include transcription factor binding sites, operator mutations, and T7 RNA polymerase mutations. With respect to LacI regulation of a promoter via the number of binding sites, there is a practical limit on the number of binding sites that can be used in a single promoter. As such, the number of binding sites in a single promoter only represents one coarse grain 'knob' for tweaking weights. The example of T7 RNA polymerase is also coarse-grained. The power and flexibility come from combining such coarse-grain approaches with others that provide more fine-grain tuning (e.g., operator sequence mutations). For the operator mutations, we show experimentally seven different weights with good coverage of the desired range and support the feasibility of obtainingnear-continuous control (Supplementary Fig. 86). Importantly, we show experimentally that replacing the open-loop control with closed loop feedback control shifted the range of weights from 0.25-0.80 to 0.75-1.70 (Supplementary Fig. 89). This is an example of how coarse grain and fine grain control can be used synergistically to obtain desired weights. In terms of additional control, other synergistic approaches mentioned above include Hill coefficients of small molecule inducers that serves as perceptgene inputs, transcription factors that competitively inhibit expression via steric hindrance, regulation of negative feedback strength, transcription factor sequestration via protein-protein interactions, protein structure (*e.g.*, dimerization and cooperativity), and circuit topology. As such, optimization and reconfiguration of neuronal circuit function is not solely dependent on the success or failure of any particular approach. These approaches can be mixed and matched, and the impact on the cost function can be then observed in order to further refine neuronal circuit behavior. Clearly, at the moment, these modulations are not as easy to manipulate as, for example, modifying weights in a computer simulation of neuronal circuits.

Scaling and optimization of neuromorphic circuit based on using design principles from analog and digital computation (Supplementary Notes, Sections 5 and 7), fuzzy computation (Supplementary Notes, Sections 3 and 4), algorithms from ANNs as gradient descent and backpropagation (Supplementary Notes, Section 6), and modules that were developed in the neuromorphic field such as Hopfield networks [58]. Here we provide another example to design 2-Bit Full adder based on neuromorphic design.
We start by presenting the truth table of our circuit (Supplementary Table 32):

Supplementary Table 32. Truth table of 2-bit Full Adder.

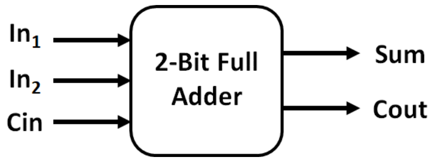

In_1	In_2	C_{in}	In_2+C_{in}	Sum	Cout
**0**	0	0	0	0	0
**0**	0	1	1	1	0
**0**	1	0	1	1	0
**0**	1	1	2	0	1
**1**	0	0	0	1	0
**1**	0	1	1	0	1
**1**	1	0	1	0	1
**1**	1	1	2	1	1

As shown in the Supplementary Table 32, the *Cout* output displays "1" if and only if the majority of the inputs are "1", and displays "0" if and only if the majority of the inputs are "0". Such function is called 3-input majority and is implemented in this study (Fig. 3 and Supplementary Notes, Section 5). The implementation of *Sum* output of 2-bit Full adder is more complex depending on the design roles (e.g., the input numbers of single perceptgene). According to the truth Supplementary Table 32, when the input In_1 is "0", the summation of In_2 and C_{in} inputs can be encoded to band-pass filter circuit (BPF), and when the input In_1 is "1" the summation of In_2 and C_{in} inputs can be encoded to NOT-BPF (NBPF). Our 2-bit Full adder comprises BPF, NBPF and 2-1 multiplexer (Supplementary Fig. 91a). A NBPF circuit is an inverted BPF circuit, which means, a high output results if and only if the input level is very low/high, and low output results for intermediate levels of input. The implementation of BPF is shown in

Supplementary Notes, Section 7, and it includes two cascaded perceptgenes that one inhibits the other (Supplementary Fig. 91). By wiring the output of BPF with an inhibitor, one can simply implement the NBPF. The 2-1 multiplexer selects between the BPF and NBPF output signals and forwards it to Sum output (Supplementary Fig. 91c). In our design, the In_1 acts as a selector and the outputs of BPF and NBPF are the data signals which are forwarded to the Sum output. The operation of Multiplexer can be described as $u\{Z_{BPF} \cdot (1 - In_1)\} + u\{Z_{NBPF} \cdot In_1\}$, where u is the sigmoid function. Supplementary Fig. 91d shows a digital design of 2-bit Full adder which include 6 AND gates, 3 OR gates, and 2 NOT gates. According to the assumption that each 2-input logic gate can be implemented only by 2 transcription factors, we get that neuromorphic design requires 9 transcription factors while the digital design requires 20 transcription factors. Notably, that 2-bit Full adder can be implemented with other biological parts [59] than transcription factors, which might require fewer components.

(a)

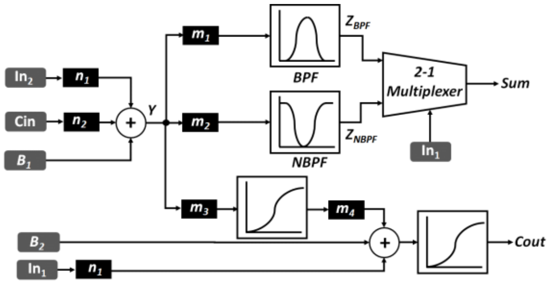

(b)

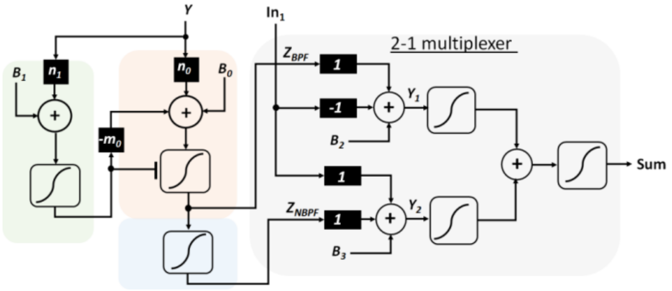

(c)

In_0	Z_{BPF}	Z_{NBPF}	Sum
0	0	0	0
0	0	1	0
0	1	0	1
0	1	1	1
1	0	0	0
1	0	1	1
1	1	0	0
1	1	1	1

(d)

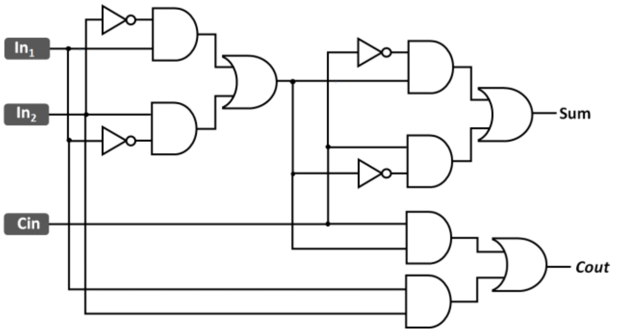

Supplementary Fig. 91. (a) Design of 2-bit Full adder. **(b)** Design of band-pass (BFP) filter circuit BPF, NOT-BPF (NBPF) and 2-bit Multiplexer. **(c)** Truth table of 2-bit Multiplexer. **(d)** Digital design of 2-bit Full adder.

9. Dynamic Measurements of Neuromorphic Genetic Circuits

We characterized the dynamics of perceptgenes implementing the power-law and multiplication function and the average function, as well as the multi-layered 2-bit ADC. These experiments monitor the progress of circuit output at multiple time points (4.5, 7, 8.5 and 10 hours). In general, circuit output is quite stable across all of these time points (Supplementary Figs. 92, 94, and 95). The maximum and minimum levels of the LSB and MSB circuit in the multi-layered 2-bit ADC across all input dosages reach approximately 2/3 of their highest values after 4.5 hours (Supplementary Fig. 96). These levels gradually increase until they reach their peak at 8.5 hours and then decreases back to about 2/3 of the maximum at 10 hours. Importantly, the input levels where LSB and MSB outputs transition between low and high levels are consistent across all time points (Supplementary Fig. 97). Therefore, at all-time points measured the ADC continues to properly convert AHL input concentration levels to the appropriate four output states [0,0], [0,1], [1,0] and [1,1]. Similar dynamic behavior is observed for the average circuit and power-law and multiplication circuit (Supplementary Figs. 92 and 94). The dynamics of our circuits are mainly determined by the characteristics of the synthetic parts and the regulatory topologies. The synthetic parts we use are based on parts that have been extensively characterized in the literature. The regulatory topologies that govern the behavior of our circuits include cascades, feed-forward, and feedback motifs – again, motifs that frequently occur in synthetic biology. by definition, we expect that the dynamics of our neuromorphic circuits are roughly the same as existing digital and analog circuits using similar synthetic parts and motifs [2,4,14], e.g., response times in few hours.

Time-course experiments were performed on perceptgene for computing power-law and multiplication function (Figs. 1b and d), perceptgene for computing an average function (Figs. 2g and i) and on ADC circuits (Figs. 4f and g). *E. coli* strains were picked from LB agar plates and grown overnight at 37°C, 300 r.p.m. in 5 mL of LB medium with appropriate antibiotics and inducers (Carbenicillin (50 µg/ml), Kanamycin (30 µg/ml), Chloramphenicol (34 µg/ml)). Overnight cultures were diluted 1:100 into 5 mL of LB medium with added antibiotics and were then incubated at 37°C, 300 r.p.m. for 30 min. 200 µl of culture was then moved into a 96-well plate, combined with inducers (Arabinose and AHL 3OC6HSL), and incubated in a VWR microplate shaker at 37°C, 500 r.p.m. Once the diluted cultures grew to an OD_{600} of ~0.5 (~4 hours and 30 min), 120 µl of culture was taken to a FACS machine for measurement. Simultaneously, we performed two steps:

1. 40 µl of culture was moved into a new 96-well plate containing 200 µl of media, antibiotics, and inducers and then incubated in a VWR microplate shaker at 37°C, 500 r.p.m. At OD_{600} ~0.5 (after 2.5 hours), 200 µl of culture was taken to a FACS machine for measurement and 40 µl of culture was moved into a new 96-well plate containing 200 µl of media, antibiotics, and inducers and then incubated in a VWR microplate shaker at 37°C, 500 r.p.m. This iterative dilution, growth, and measurement process was repeated and resulted the dynamics after 7 hours and 10 hours.
2. 20 µl of culture was moved into a new 96-well plate containing 200 µl of media, antibiotics and inducers, then incubated in a VWR microplate shaker at 37°C, 500 r.p.m. At OD_{600} ~0.5 (4 hours and 20 min), 200 µl of culture was taken to a FACS machine for measurement and resulted the dynamics after 8.30 hours.

The experimental results of power-law, multiplication function, and average circuits corresponding to different times are shown in Supplementary Fig. 92 and 94 below shown by fitting our experimental results to surface, the weight values are consistent over time (Supplementary Fig. 93). The average circuit continues to operate correctly and compute the average between two analog numbers (Supplementary Fig. 94).

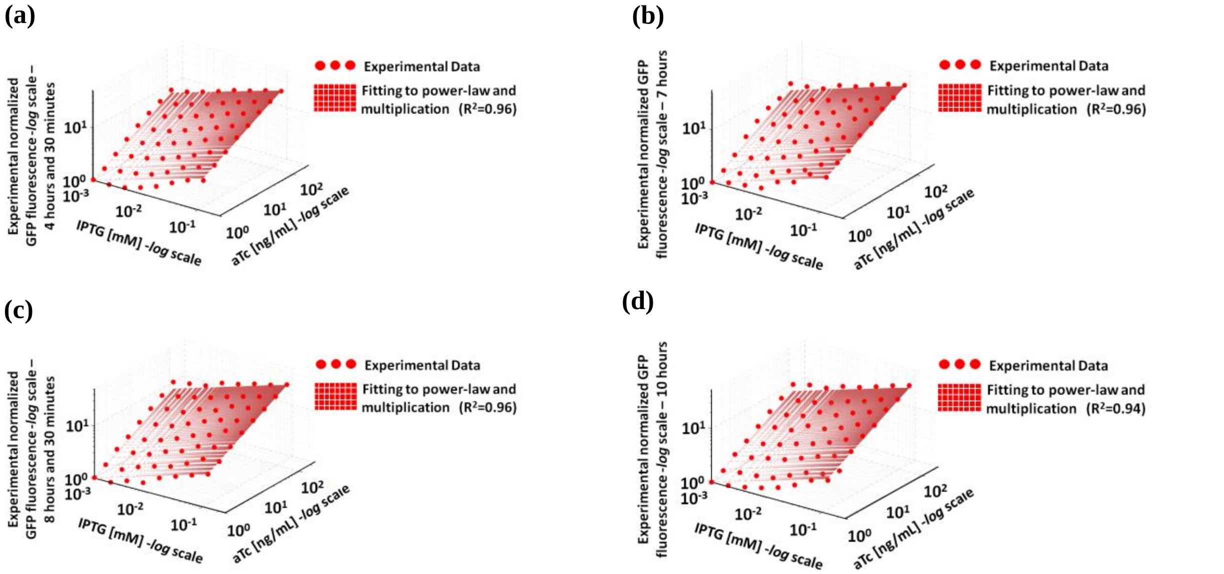

Supplementary Fig. 92. Dynamics of power-law and multiplication computing circuit. Normalized data of Time-course experiments (4.5 hours, 7 hours, 8.5 hours and 10 hours) for perceptgene that computes power-law and multiplication function (Matching Fig. 1d with P_{lacO1}/P_{tetO}). Solid line indicates the fitting of the experimental data to power-law and multiplication function : **(a)** 4.5 hours with $\left(\frac{IPTG}{1.25}\right)^{0.3} \cdot \left(\frac{aTc}{0.7}\right)^{0.4}$ with R^2=0.96, **(b)** 7 hours with $\left(\frac{IPTG}{1.25}\right)^{0.32} \cdot \left(\frac{aTc}{0.7}\right)^{0.42}$ with R^2=0.96, **(c)** 8.5 hours with $\left(\frac{IPTG}{1.25}\right)^{0.31} \cdot \left(\frac{aTc}{0.7}\right)^{0.41}$ with R^2=0.96, **(d)** 10 hours with $\left(\frac{IPTG}{1.25}\right)^{0.31} \cdot \left(\frac{aTc}{0.7}\right)^{0.405}$ with R^2=0.94.

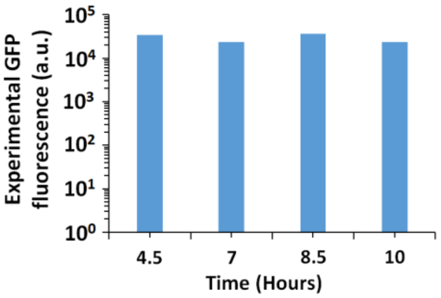

Supplementary Fig. 93. Maximum values of raw data of time-course experiments (4.5 hours, 7 hours, 8.5 hours and 10 hours) for for perceptgene that computes power-law and multiplication function based on Supplementary Fig. 92.

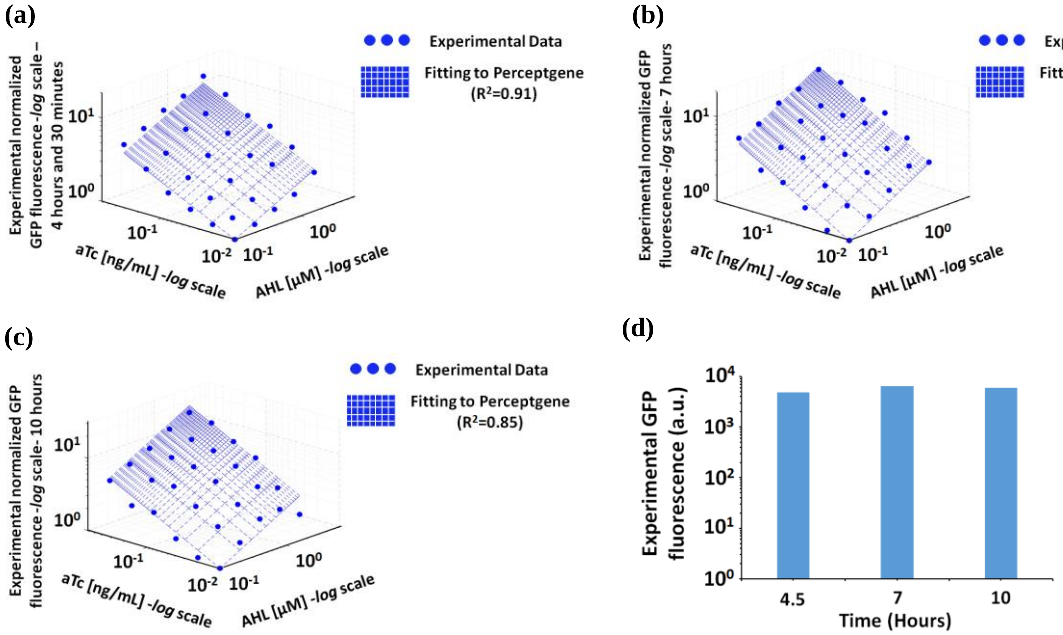

Supplementary Fig. 94. Dynamics of average computing circuit. Normalized data of Time-course experiments (4.5 hours, 7 hours and 10 hours) for perceptgene that computes average function (Matching Figs. 2g and h). Solid line indicates the fitting of the experimental data to perceptgene model: **(a)** 4.5 hours with $\frac{[\left(\frac{AHL}{6}\right)^{0.45}\cdot\left(\frac{IPTG}{7.2}\right)^{0.42}/400]^{0.95}+0.0001}{[\left(\frac{AHL}{6}\right)^{0.45}\cdot\left(\frac{IPTG}{7.2}\right)^{0.42}/400]^{0.95}+1}$ with R^2=0.92, **(b)** 7 hours with $\frac{[\left(\frac{AHL}{6}\right)^{0.45}\cdot\left(\frac{IPTG}{7.2}\right)^{0.42}/400]^{1.05}+0.0001}{[\left(\frac{AHL}{6}\right)^{0.45}\cdot\left(\frac{IPTG}{7.2}\right)^{0.42}/400]^{1.05}+1}$ with R^2=0.88, **(c)** 10 hours with $\frac{[\left(\frac{AHL}{6}\right)^{0.45}\cdot\left(\frac{IPTG}{7.2}\right)^{0.42}/400]^{1.14}+0.0001}{[\left(\frac{AHL}{6}\right)^{0.45}\cdot\left(\frac{IPTG}{7.2}\right)^{0.42}/400]^{1.14}+1}$ with R^2=0.85, **(d)** Maximum values of raw data of time-course experiments (4.5 hours, 7 hours, and 10 hours) for for perceptgene that computes average function.

The experimental results of ADC corresponding to different times are shown in Supplementary Fig. 95 below. The GFP signal of the ADC circuit represents the LSB output, and the mCherry signal of the ADC circuit represents the MSB output. In the fours time points (4.5 hours, 7 hours, 8.5 hours and 10 hours) our ADC continues to operate properly and convert the AHL concentration level to four states [0,0], [0,1], [1,0] and [1,1] (Supplementary Fig. 96) We also compared the minimum and maximum expression levels of GFP and mCherry at different time points (Supplementary Fig. 97). Furthermore, we fitted the data to an empirical model, and we found that the fitting parameters change slightly across time (Supplementary Table 33). The empirical model is based on Section 7 and is given by:

$$LSB_{low} = \frac{\left(\frac{AHL}{K_{m1}}\right)^{h1}}{1+\left(\frac{AHL}{K_{m1}}\right)^{h1}}\cdot\frac{1}{1+\left(\frac{AHL}{K_{m2}}\right)^{h2}} + b_1 \quad (9.1)$$

$$LSB_{high} = \frac{\left(\frac{AHL}{K_{m3}}\right)^{h3}}{1+\left(\frac{AHL}{K_{m3}}\right)^{h3}} + b_2 \quad (9.2)$$

$$LSB = \frac{LSB_{low}}{Max(LSB_{low})} + \frac{LSB_{high}}{Max(LSB_{high})} \quad (9.3)$$

$$MSB = \frac{\left(\frac{AHL}{K_{m4}}\right)^{h4}}{1+\left(\frac{AHL}{K_{m4}}\right)^{h4}} + b_3 \quad (9.4)$$

Where K_{mi} are dissociation constants, h_i are Hill-coefficients and b_i are basal levels.

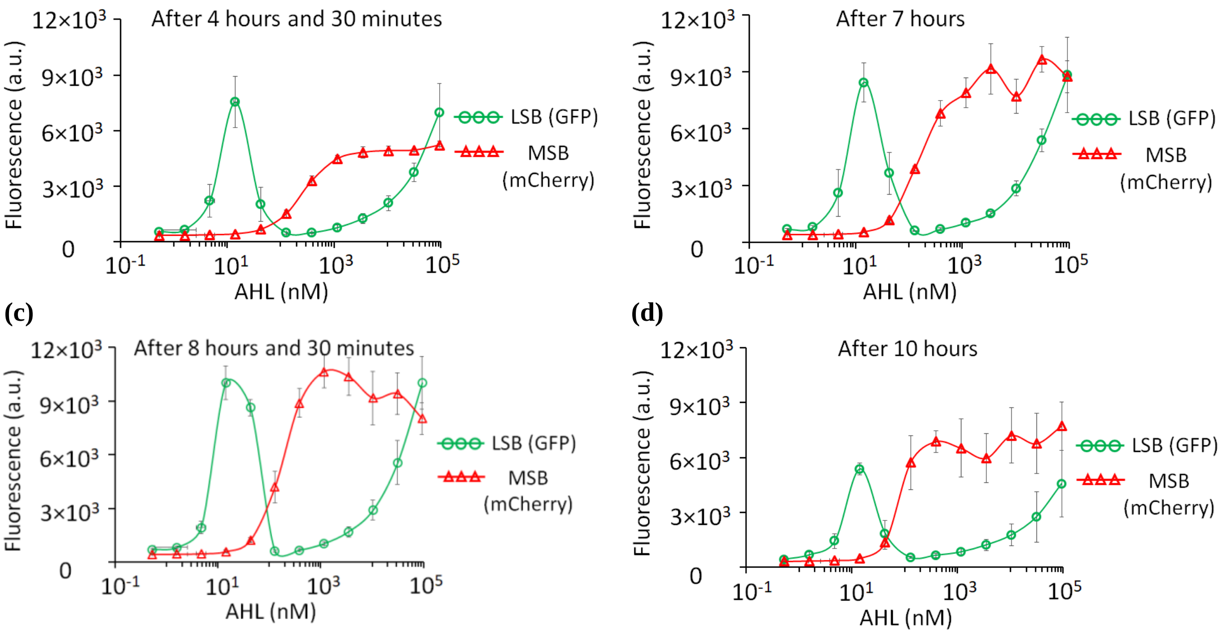

Supplementary Fig. 95. Raw data of time-course experiments (**(a)** 4.5 hours, **(b)** 7 hours, **(c)** 8.5 hours and **(d)**10 hours) for the ADC circuit. To enable a suitable comparison between the GFP and mCherry signals, we scaled the measured mCherry signal by 23. Data are presented as average ±standard deviations from independent replicates ($n = 3$).

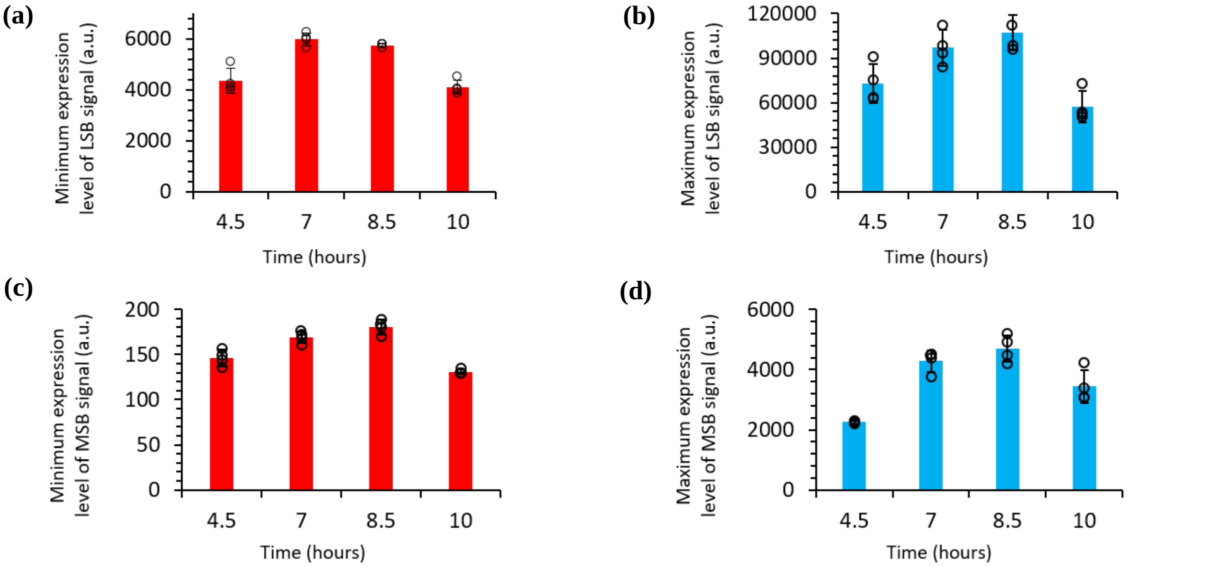

Supplementary Fig. 96. Time course of expression levels for the ADC circuit. **(a)** Minimum level of GFP (LSB). **(b)** Maximum level of GFP (LSB). **(c)** Minimum level of mCherry (MSB). **(d)** Maximum level of mCherry (MSB). Blue and red bars and their error bars show the average and standard deviation from independent replicates ($n = 3$).

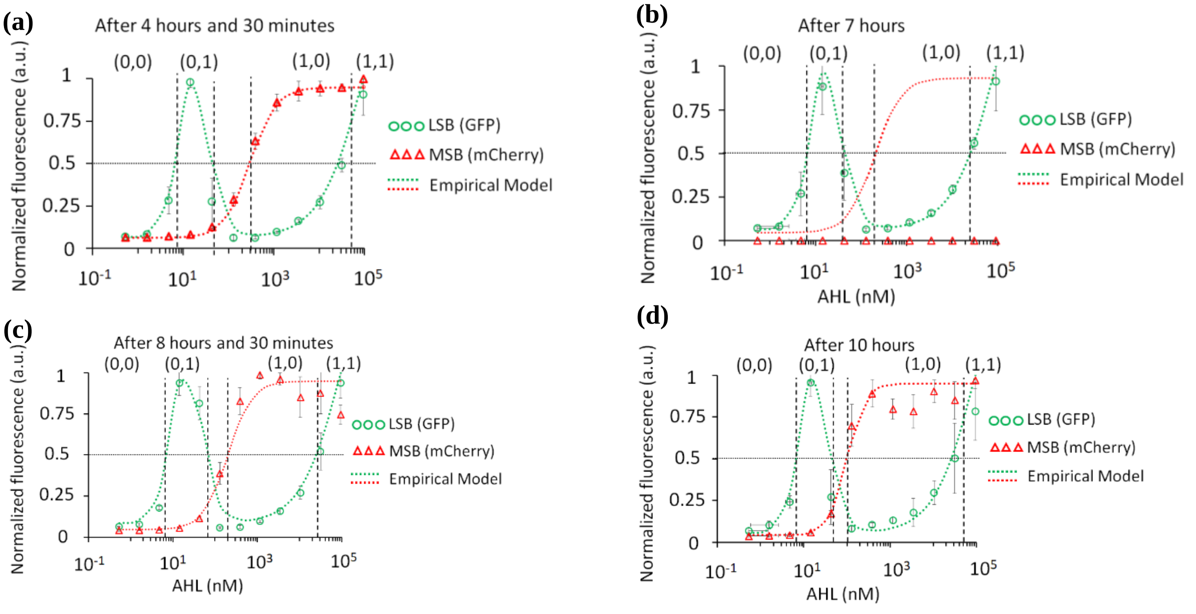

Supplementary Fig. 97. Normalized data for Time-course experiments (**(a)** 4.5 hours, **(b)** 7 hours, **(c)** 8.5 hours and **(d)** 10 hours) of the ADC circuit. The dotted line corresponds to a set of equations; Eq. 9.1-4. The average signals and standard divisions were calculated for the normalized signals. Therefore, there is a difference in the standard deviation values compared to Supplementary Fig. 95. Data are presented as average ±standard deviations from independent replicates ($n = 3$).

Supplementary Table 33. Shows the time course fitting parameters (Eq. 9.1-.4) for the ADC circuit. The empirical model is based on Section 8.

Parameter	Time=4.5 hours	Time=7 hours	Time=8.5 hours	Time=10 hours
K_{m1}	20nM	20nM	**30nM**	**20nM**
K_{m2}	15nM	15nM	15nM	15nM
K_{m3}	10^5nM	10^5nM	10^5nM	10^5nM
K_{m4}	300nM	**200nM**	200nM	**100nM**
h_1	2	2	2	2
h_2	2	2	2	2
h_3	0.9	0.9	0.9	0.9
h_4	1.5	1.5	1.5	1.5
b_1	0.01	0.01	**0.008**	0.008
b_2	0.005	0.005	0.005	0.005
b_3	0.07	**0.05**	0.05	**0.03**

10. Noise Analysis in Neuromorphic Circuits

In this section, we perform the Signal-to-Noise ratio (SNR). The sensitivity analysis from Section 1 and SNR parameters can provide quantitative information for the precision and reliability of circuits. We quantified the noise of neuromorphic circuits by further analysis of single-cell FACS experimental data. Specifically, for each single layer perceptgene circuit (from Figs. 1 and 2) we quantified the signal-to-noise ratio for each input dosage that we tested and graphed a corresponding SNR histogram (Supplementary Fig. 98). These histograms show the distribution of SNR exhibited by each of the circuits. The general observation is that the power law and multiplication circuits that use only auto-negative feedback for the inputs (LacI/IPTG and TetR/aTc) generally tend to have higher SNRs than circuits that include auto-positive feedback (LuxI/AHL with either TetR or LacI). Another observation is that the activation function (AraC) addition tends to coalesce the SNR distributions of all three circuits to roughly the same values. Thus, activation functions utilized in two circuits were able to increase the SNR.

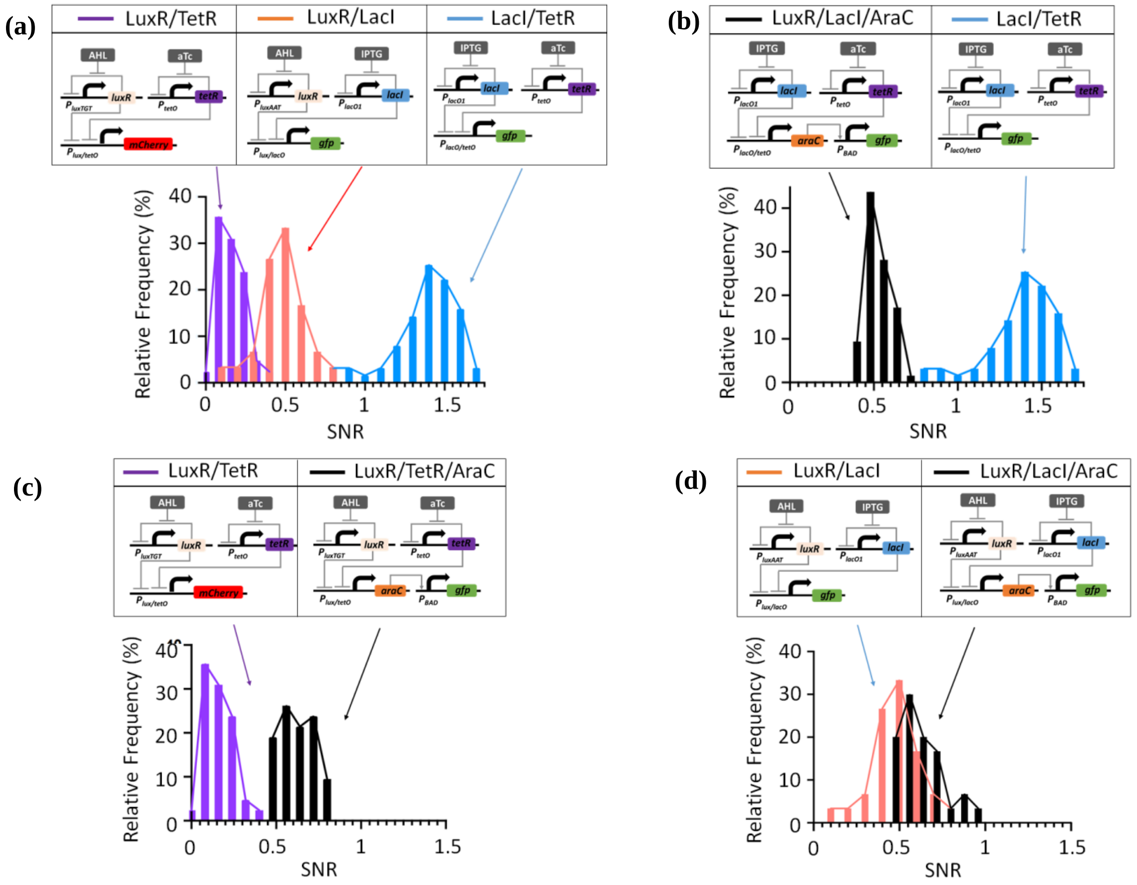

Supplementary Fig. 98. Signal-to-Noise ratio (SNR) analysis of perceptgene units (Figs. 1 and 2 in the main text). **(a)** SNR analysis for power-law and multiplication circuits. The circuit with only negative feedback (via LacI and TetR) exhibits improved SNR over the other two circuits that contain a positive feedback motif (via LuxR). **(b)**, **(c)** and **(d)** SNR analysis for perceptgene with activation functions computes smooth minimum, maximum and average functions, respectively.

11. Comparing neuromorphic computing with digital and analog computing

Our selection of circuits to design and build was based on two guidelines:

1. To prove that, for the first time, neuromorphic computing principles can be achieved in single living cells by transforming concepts from neural networks to genetic regulatory networks.
2. To construct synthetic gene circuits that perform complex computation with minimal requirements in computational devices and host cell resources.

Three of the circuits we decided to build, min/max/avg, are fundamental building blocks for neuromorphic computing. The other three, majority / 2-bit ADC / ternary switch, demonstrate multi-layered neuronal circuits.
In terms of appreciating the min/max/avg functions, it is essential to recognize that to the best of our knowledge, only "hard" (i.e., discrete) minimum and maximum functions have been demonstrated in synthetic biology. Complicated functions operate using binary AND/OR logic, where each bit has two logic states. In terms of these complicated functions, AND implements binary min, while OR implements binary max. The average function cannot be implemented with an individual single-bit binary logic gate but would instead require digitization of input and output signals and very complex multi-device logic. In sharp contrast, soft functions operate in the analog domain. Our single perceptgenes implement single-device analog min/max/avg computations whereby the single devices transform analog input signals into output values that remain in the analog domain. These operations have not been demonstrated in synthetic biology!
Our multi-layer functions also represent significant progress over existing efforts in synthetic biology. Our majority function (1) demonstrates neuromorphic modularity because the three-input majority function is built from two-layer perceptron and (2) allows us to compare the properties of neuromorphic design with digital design. Our three-input majority function has two main advantages over the previous digital design [4]: (1) We use fewer synthetic parts; our three-bit majority function comprises 15 biological parts (i.e., promoters and genes) in comparison to 22 parts, (2) the neuromorphic circuit is reconfigurable and trainable via learning algorithms that optimize desired behavior efficiently (e.g., reduce error). With this neuromorphic architecture, we minimized error by modulating the weight of $P_{BAD}/AraC$ in a manner similar to backpropagation algorithm. This optimization approach could not have been performed for the existing digital circuit design.
Our other two multi-layer neuromorphic circuits also provide innovation beyond existing approaches. To the best of our knowledge, we are the first to demonstrate a 2-bit analog-to-digital converter (ADC). In general, analog-to-digital converters take as input a graded signal, partition the analog input into several consecutive ranges that cover the entire input range, and assign a digital value to these ranges in a sequential manner. Representing this digital value requires multiple bits if more than two regions are specified. A 1-bit ADC partitions the input range into two, and the output is then a single bit with a value of either 0 or 1. A 2-bit ADC partitions the input range into four, with an output that requires two bits representing each of the four consecutive ranges, namely 00, 01, 10 and 11.
Two recent synthetic biology publications have discussed the notion of analog-to-digital converters. In one publication [67], 1-bit analog-to-digital conversion was used to quantize extracellular inputs (including dihydrojasmone and eugenol) each into single-bit values, and then these were combined into several 2-input logic functions (AND, OR, NOR) still operating with single bit output. In another recent publication [42], a single analog input (H_2O_2) was partitioned into three consecutive ranges, and three separate 1-bit outputs (GFP, RFP, and BFP) were used to indicate which of three ranges was detected. Hence, one of these digital outputs is high for a given analog input value while the other two are low. In conventional ADC circuit design, these three 1-bit outputs are then combined via a second stage digital logic circuit (comprising three 2-input logic gates: one XOR and two AND gates) to create a 2-bit digital representation of the analog input signal. Hence, this work represents only the first stage of a 2-bit ADC, but not the second stage. In terms of biological circuit elements, they used seven transcription units. We estimate that it would require 6-8 additional transcription units to implement their second stage of the 2-bit ADC, which would require a total of 15 transcription units if it was built. In comparison, ours is a fully functional 2-bit ADC implemented using only five transcription units. Besides minimizing the size of the circuits, our perceptgene networks also operate with low

expression levels, mainly in order to maintain low bias levels. In contrast, digital systems often attempt to operate with significant noise margins, and hence high expression levels for ON values. This latter point is further elaborated on in the main narrative.

The implementation of our third multi-layered neuromorphic circuit, the ternary switch, demonstrated the ease of converting one neuromorphic computing to another. Specifically, we started with the 2-bit ADC circuit and increased the LSB perceptgene activation using higher Arabinose, which corresponds to increasing the MSB input weight into the LSB computation. Such ease in changing neuronal network parameters and achieving new functions is an important component for ultimately implementing learning algorithms using gene circuits.

12. Potential Applications of Synthetic Neuromorphic Circuits

For example, a three-input majority function can be fitted to any logic gate with up to three inputs, including the two-input AND gate [68,69], and optimized in applications currently suffering from a trade-off between specificity and sensitivity. Typically, synthetic gene circuits for disease treatment must be highly sensitive to detect biomarkers and deliver the produced therapeutic proteins to target cells and must be precisely specified to protect surrounding healthy cells [57]. Ternary converters may also be helpful in engineering cells whose therapeutic outputs are connected in a closed-loop and are regulated by quantitative levels of disease biomarkers. While circuits behave either in an analog manner, showing insensitivity to disease biomarkers, or in a digital manner, in which they are quantized to a single level of therapeutic proteins, ternary converters with feedback loops can settle at two saturated levels and can precisely adjust the production of the therapeutic proteins (e.g., adiponectin which attenuates insulin-resistance syndrome [70]) to the level required for disease management. Furthermore, data converters may find applications in biotechnology either by coordinating the expression of several genes, using a single inducer or by improving the production yield of the desired biomass in synthetic pathways, using a three-state genetic switch. For example, engineered cells that produce quorum-sensing signals (AHL) and contain a ternary converter, could have multilevel phases, dictated by accumulated AHL levels in the bioreactor. These phases could efficiently optimize the production rate versus the cell growth rate compared to the two-state switch [71].

Another example is that the next-generation therapeutic-based synthetic gene circuits can be self-controlled once administered, replacing the need for exhaustive manipulation by a manually customized, trial-and-error clinical design. Recently, the design cycle of bioproducts has driven the set-up of laboratory automation, foundries (e.g., robotics) and information infrastructures [72] using ‘design, build, test, learn and correct’ heuristics. We expect that adaptive genetic circuits will significantly standardize this cycle, drastically reduce time-to-market and cost, through a generic methodology, using training algorithms suitable for general purpose applications.

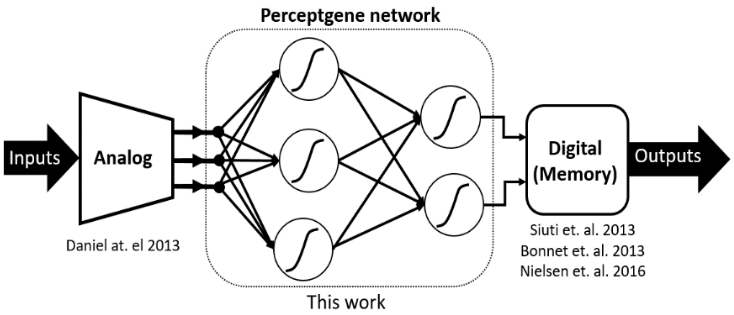

Supplementary Fig. 99. proposed an efficient and reliable computing platform, which combines analog, a perceptgene network, and digital memory for sorting.

Supplementary Fig. 99 shows the proposed platform is compatible with digital and analog computing platforms using data converters. This complementary strategy can leverage the advantages of the three platforms to achieve an efficient and accurate computational approach for scaling the architecture of robust genetic networks in living cells. For instance, analog computing can be applied for front-end calculations (e.g., ratiometric for sensory systems), perceptgene networks can be applied for processing and computation, and digital circuits can be applied back-end data storage (memory) with clear ON and OFF states.

13. FACS Data

All fluorescence intensities were smoothed using Matlab.

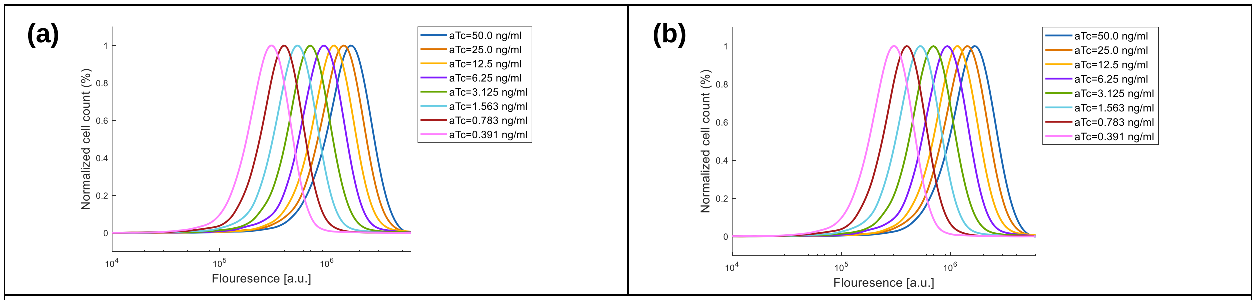

Supplementary Fig100. GFP flow cytometry data for a population of cells containing the synthetic perceptgene based on ANF loops (Fig. 1c). **(a)** IPTG was held constant at 125 µM, and aTc was varied. **(b)** aTc was held constant at 50 ng/ml, and IPTG was varied. Source data are available in the Source data file.

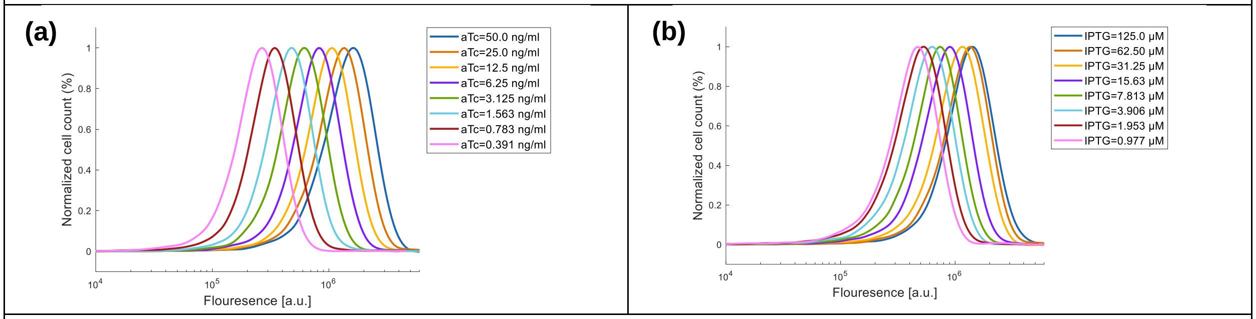

Supplementary Fig. 101. GFP flow cytometry data for a population of cells containing the synthetic perceptgene based on ANF loops (Fig. 1c). **(a)** IPTG was held constant at 62.5 µM, and aTc was varied. **(b)** aTc was held constant at 25 ng/ml, and IPTG was varied. Source data are available in the Source data file. Source data are available in the Source data file

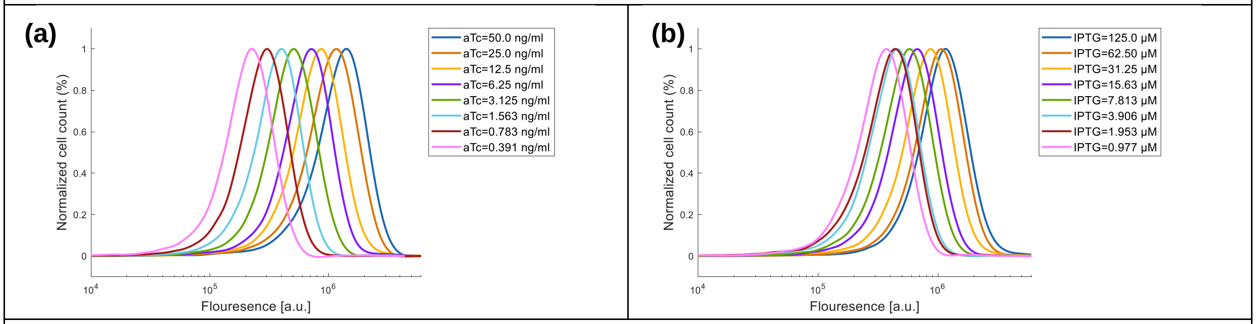

Supplementary Fig. 102. GFP flow cytometry data for a population of cells containing the synthetic perceptgene based on ANF loops (Fig. 1c). **(a)** IPTG was held constant at 31.25 µM, and aTc was varied. **(b)** aTc was held constant at 12.5 ng/ml, and IPTG was varied. Source data are available in the Source data file. Source data are available in the Source data file.

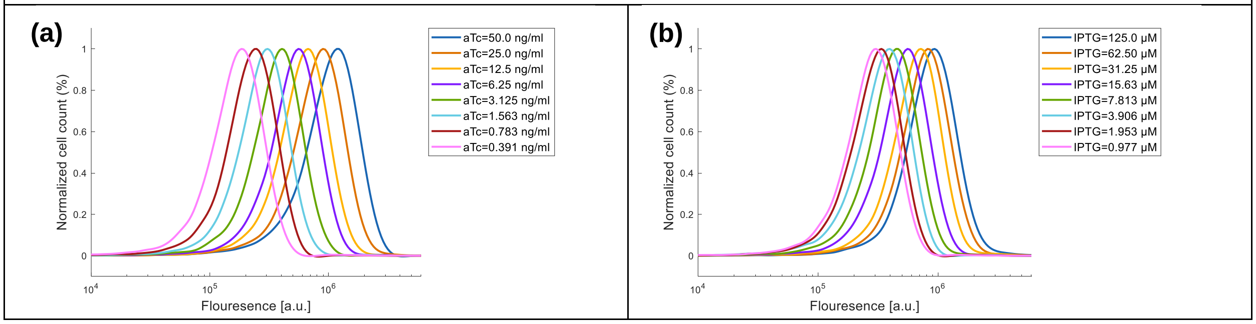

Supplementary Fig. 103. GFP flow cytometry data for a population of cells containing the synthetic perceptgene based on ANF loops (Fig.1c). **(a)** IPTG was held constant at 15.63 μM, and aTc was varied. **(b)** aTc was held constant at 6.25 ng/ml, and IPTG was varied. Source data are available in the Source data file. Source data are available in the Source data file.

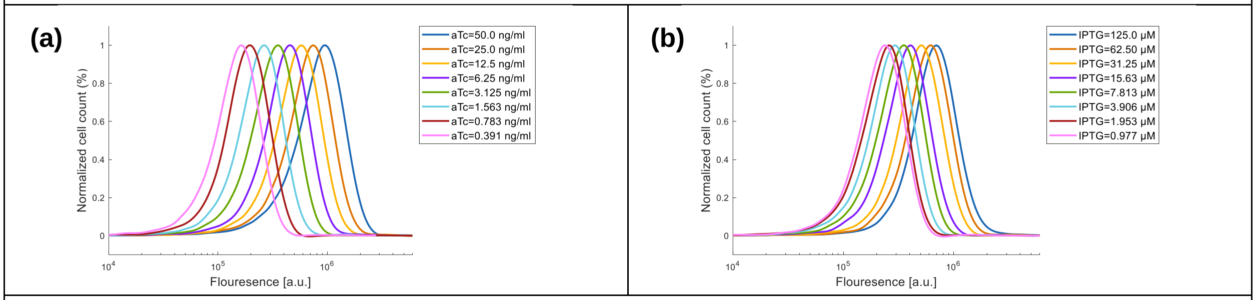

Supplementary Fig. 104. GFP flow cytometry data for a population of cells containing the synthetic perceptgene based on ANF loops (Fig. 1c). **(a)** IPTG was held constant at 7.813 μM, and aTc was varied. **(b)** aTc was held constant at 3.125 ng/ml, and IPTG was varied. Source data are available in the Source data file. Source data are available in the Source data file.

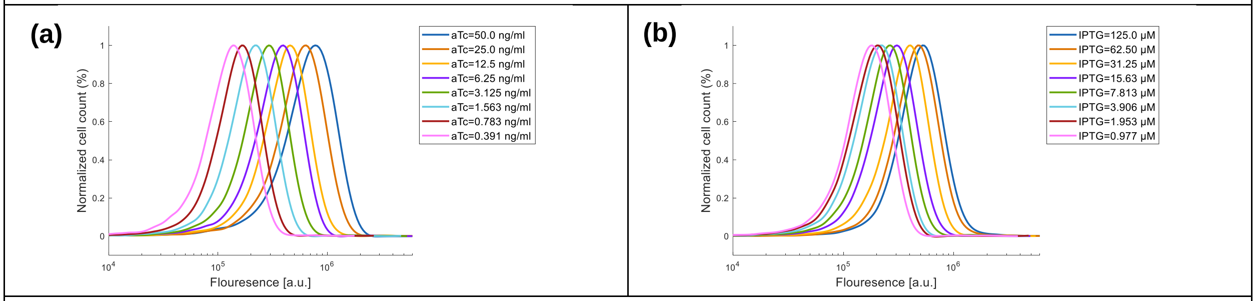

Supplementary Fig. 105. GFP flow cytometry data for a population of cells containing the synthetic perceptgene based on ANF loops (Fig. 1c). **(a)** IPTG was held constant at 3.906 μM and, aTc was varied. **(b)** aTc was held constant at 1.563 ng/ml, and IPTG was varied. Source data are available in the Source data file. Source data are available in the Source data file.

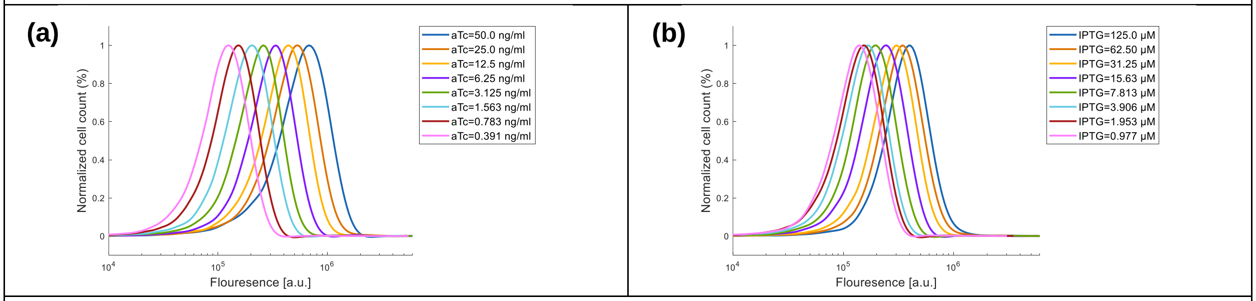

Supplementary Fig. 106. GFP flow cytometry data for a population cells containing the synthetic perceptgene based on ANF loops (Fig. 1c). **(a)** IPTG was held constant at 1.953 μM and aTc was varied. **(b)** aTc was held constant at 0.783 ng/ml and IPTG was varied. Source data are available in the Source data file. Source data are available in the Source data file.

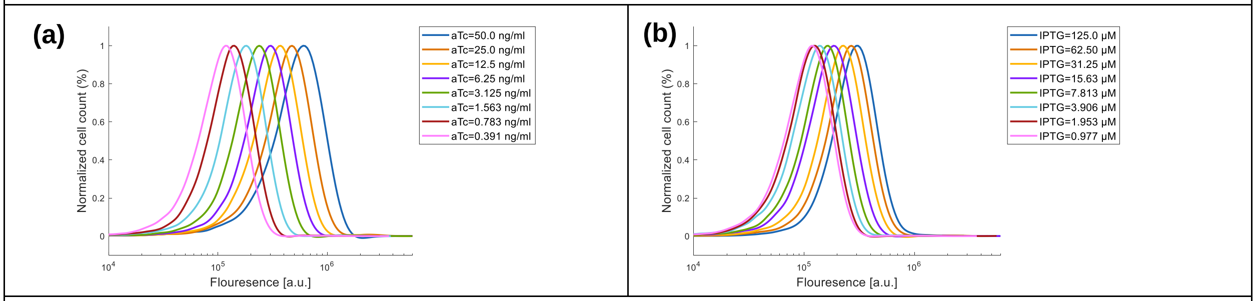

Supplementary Fig. 107. GFP flow cytometry data for a population of cells containing the synthetic perceptgene based on ANF loops (Fig. 1c). **(a)** IPTG was held constant at 0.977 μM, and aTc was varied. **(b)** aTc was held constant at 0.391 ng/ml, and IPTG was varied. Source data are available in the Source data file. Source data are available in the Source data file.

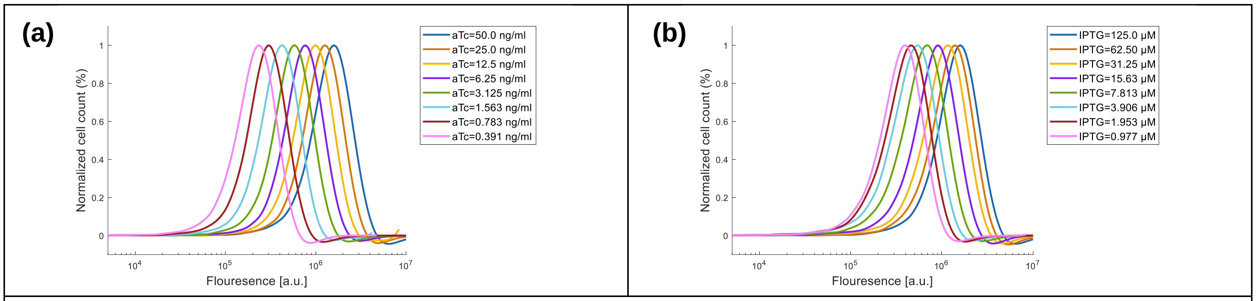

Supplementary Fig. 108. GFP flow cytometry data for a population of cells containing the synthetic perceptgene based on ANF loops. In this circuit, P_{lacO} within the ANF was replaced by P_{lacO1} (Fig. 1d). **(a)** IPTG was held constant at 125 µM, and aTc was varied. **(b)** aTc was held constant at 50 ng/ml, and IPTG was varied. Source data are available in the Source data file. Source data are available in the Source data file.

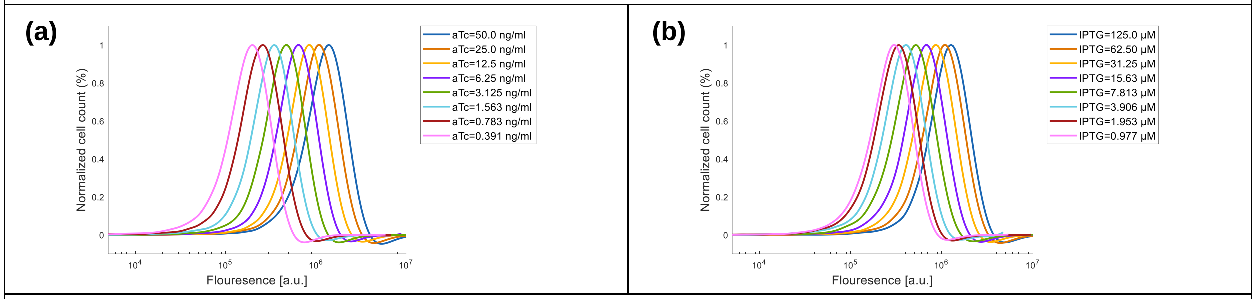

Supplementary Fig. 109. GFP flow cytometry data for a population of cells containing the synthetic perceptgene based on ANF loops. In this circuit, P_{lacO} within the ANF was replaced by P_{lacO1} (Fig. 1d). **(a)** IPTG was held constant at 62.5 µM, and aTc was varied. **(b)** aTc was held constant at 25 ng/ml, and IPTG was varied. Source data are available in the Source data file. Source data are available in the Source data file.

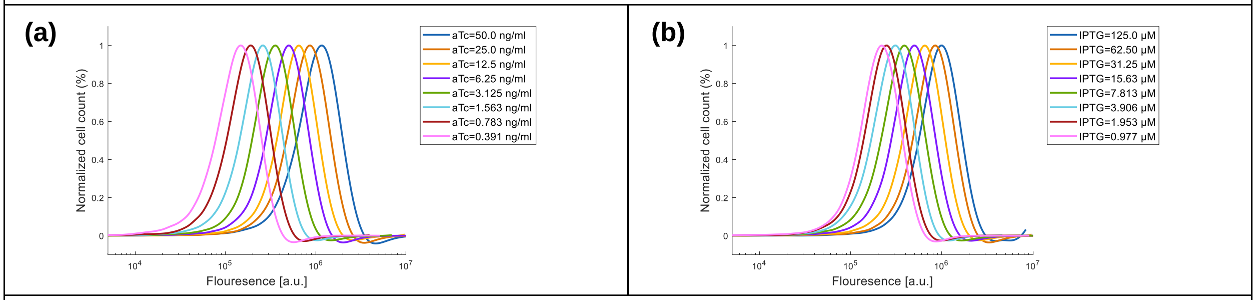

Supplementary Fig. 110. GFP flow cytometry data for a population of cells containing the synthetic perceptgene based on ANF loops. In this circuit, P_{lacO} within the ANF was replaced by P_{lacO1} (Fig. 1d). **(a)** IPTG was held constant at 31.25 µM, and aTc was varied. **(b)** aTc was held constant at 12.5 ng/ml, and IPTG was varied. Source data are available in the Source data file. Source data are available in the Source data file.

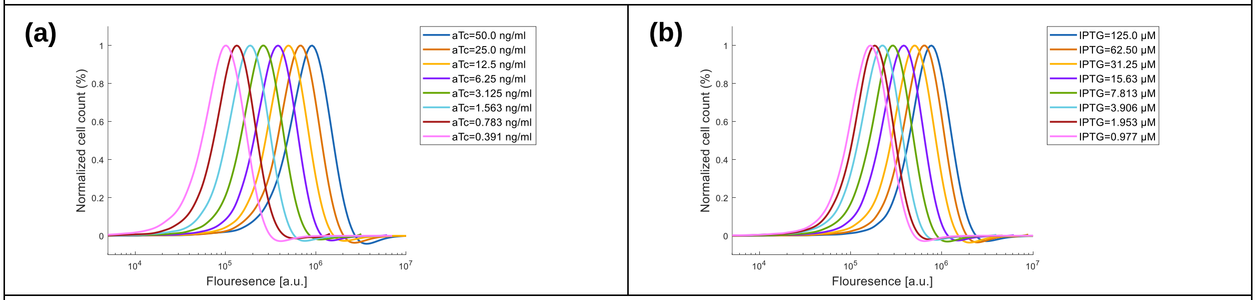

Supplementary Fig. 111. GFP flow cytometry data for a population of cells containing the synthetic perceptgene based on ANF loops. In this circuit, P_{lacO} within the ANF was replaced by P_{lacO1} (Fig. 1d). **(a)** IPTG was held constant at 15.63 µM, and aTc was varied. **(b)** aTc was held constant at 6.25 ng/ml, and IPTG was varied. Source data are available in the Source data file.

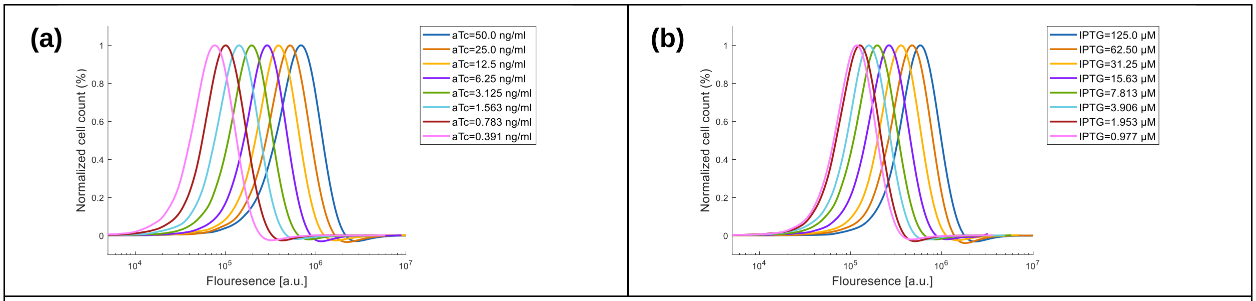

Supplementary Fig. 112. GFP flow cytometry data for a population of cells containing the synthetic perceptgene based on ANF loops. In this circuit, P_{lacO} within the ANF was replaced by P_{lacO1} (Fig. 1d). **(a)** IPTG was held constant at 7.813 μM and aTc was varied. **(b)** aTc was held constant at 3.125 ng/ml and IPTG was varied. Source data are available in the Source data file.

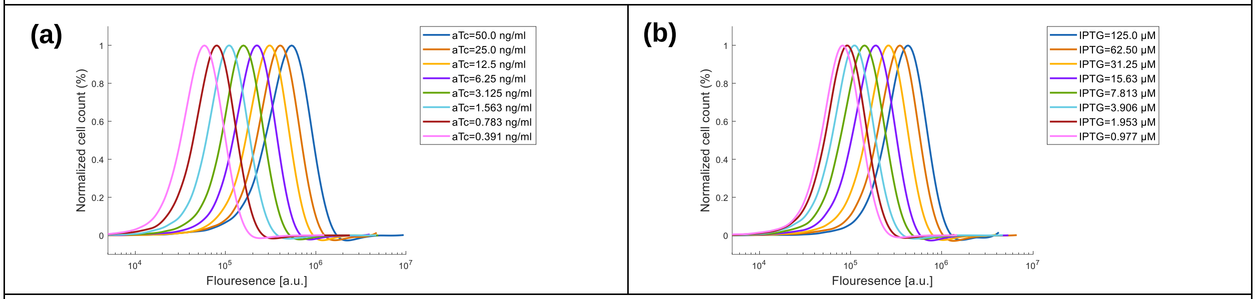

Supplementary Fig. 113. GFP flow cytometry data for a population of cells containing the synthetic perceptgene based on ANF loops. In this circuit, P_{lacO} within the ANF was replaced by P_{lacO1} (Fig. 1d). **(a)** IPTG was held constant at 3.906 μM, and aTc was varied. **(b)** aTc was held constant at 1.563 ng/ml, and IPTG was varied. Source data are available in the Source data file.

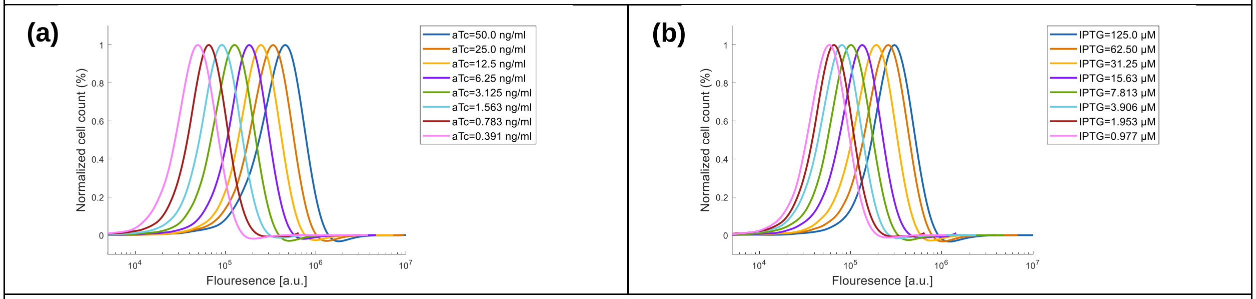

Supplementary Fig. 114. GFP flow cytometry data for a population of cells containing the synthetic perceptgene based on ANF loops. In this circuit, P_{lacO} within the ANF was replaced by P_{lacO1} (Fig. 1d). **(a)** IPTG was held constant at 1.953 μM, and aTc was varied. **(b)** aTc was held constant at 0.783 ng/ml, and IPTG was varied. Source data are available in the Source data file.

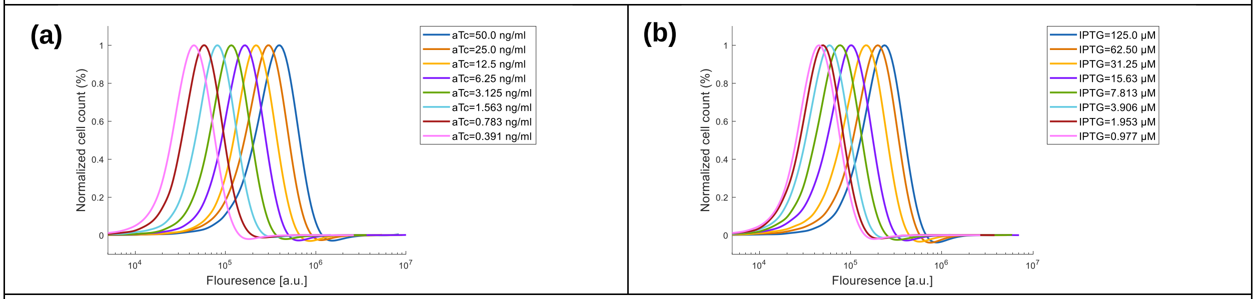

Supplementary Fig. 115. GFP flow cytometry data for a population of cells containing the synthetic perceptgene based on ANF loops. In this circuit, P_{lacO} within the ANF was replaced by P_{lacO1} (Fig. 1d). **(a)** IPTG was held constant at 0.977 μM, and aTc was varied. **(b)** aTc was held constant at 0.391 ng/ml, and IPTG was varied. Source data are available in the Source data file.

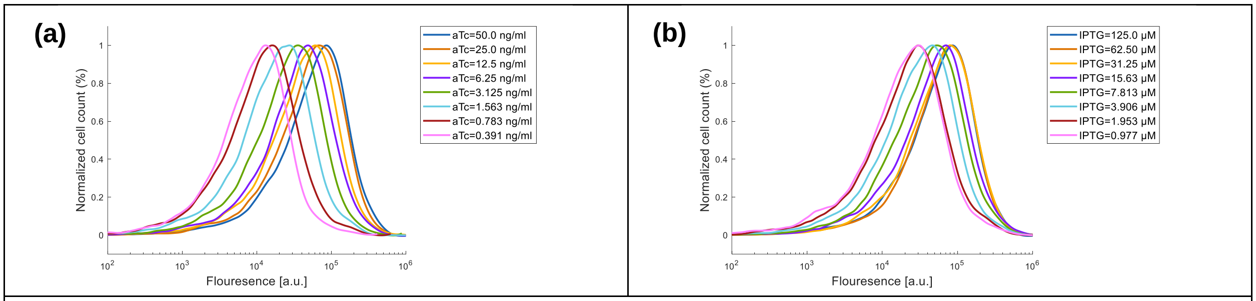

Supplementary Fig. 116. GFP flow cytometry data for a population of cells containing the synthetic perceptgene based on ANF loops. In this circuit, P_{lacO} within the ANF was replaced by P_{lacO1} and AraC truncated was used to improve the compatibility of Arabinose and IPTG (Fig. 1h). **(a)** Arabinose was held constant at 0.04 mM, IPTG was held constant at 125 µM, and aTc was varied. **(b)** Arabinose was held constant at 0.04 mM, aTc was held constant at 50 ng/ml, and IPTG was varied. Source data are available in the Source data file.

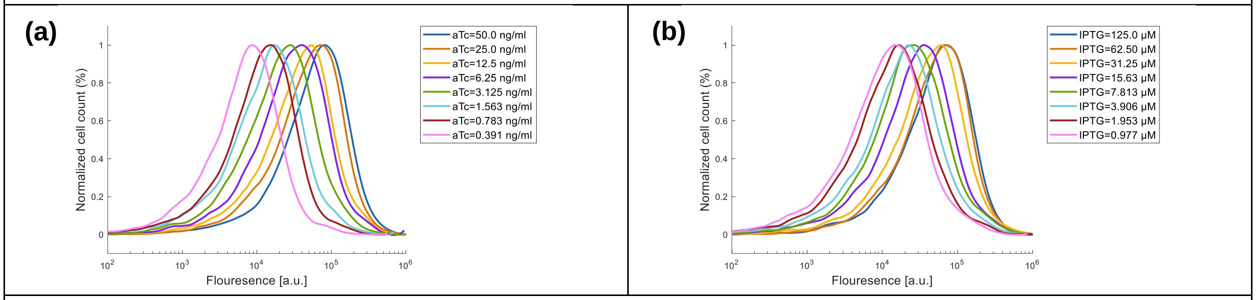

Supplementary Fig. 117. GFP flow cytometry data for a population of cells containing the synthetic perceptgene based on ANF loops. In this circuit, P_{lacO} within the ANF was replaced by P_{lacO1} and AraC truncated was used to improve the compatibility of Arabinose and IPTG (Fig. 1h). **(a)** Arabinose was held constant at 0.04 mM, IPTG was held constant at 62.5 µM, and aTc was varied. **(b)** Arabinose was held constant at 0.04 mM, aTc was held constant at 25 ng/ml, and IPTG was varied. Source data are available in the Source data file.

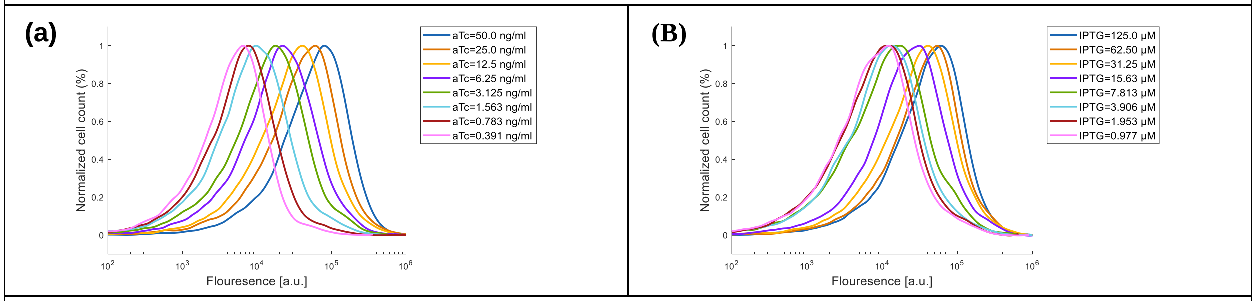

Supplementary Fig. 118. GFP flow cytometry data for a population of cells containing the synthetic perceptgene based on ANF loops. In this circuit, P_{lacO} within the ANF was replaced by P_{lacO1} and AraC truncated was used to improve the compatibility of Arabinose and IPTG (Fig. 1h). **(a)** Arabinose was held constant at 0.04 mM, IPTG was held constant at 31.25 µM and aTc was varied. **(b)** Arabinose was held constant at 0.04 mM, aTc was held constant at 12.5 ng/ml and IPTG was varied. Source data are available in the Source data file.

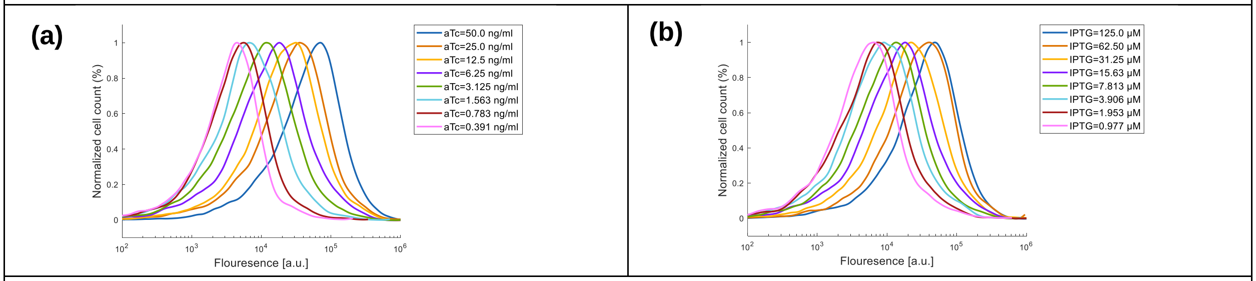

Supplementary Fig. 119. GFP flow cytometry data for a population of cells containing the synthetic perceptgene based on ANF loops. In this circuit, P_{lacO} within the ANF was replaced by P_{lacO1} and AraC truncated was used to improve the compatibility of Arabinose and IPTG (Fig. 1h). **(a)** Arabinose was held constant at 0.04 mM, IPTG was held constant at 15.63 µM, and aTc was varied. **(b)** Arabinose was held constant at 0.04 mM, aTc was held constant at 6.25 ng/ml, and IPTG was varied. Source data are available in the Source data file.

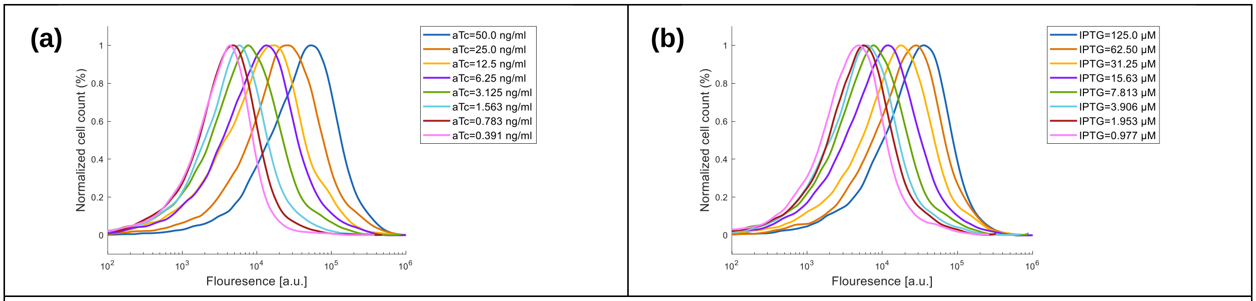

Supplementary Fig. 120. GFP flow cytometry data for a population of cells containing the synthetic perceptgene based on ANF loops. In this circuit, P_{lacO} within the ANF was replaced by P_{lacO1} and AraC truncated was used to improve the compatibility of Arabinose and IPTG (Fig. 1h). **(a)** Arabinose was held constant at 0.04 mM, IPTG was held constant at 7.813 μM, and aTc was varied. **(b)** Arabinose was held constant at 0.04 mM, aTc was held constant at 3.125 ng/ml, and IPTG was varied. Source data are available in the Source data file.

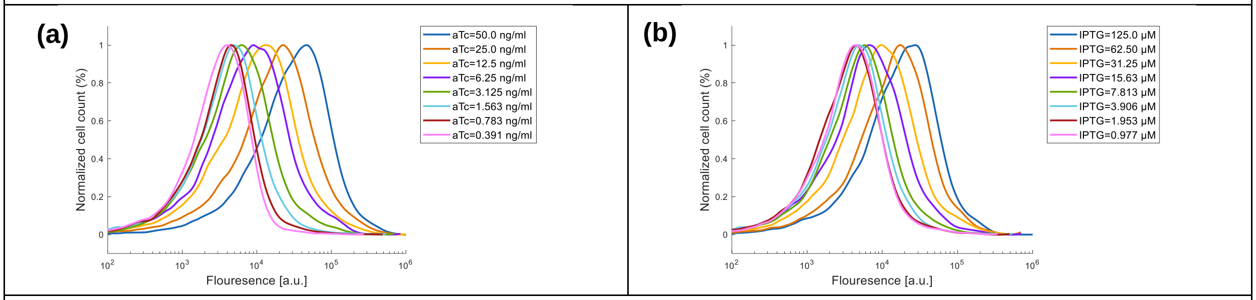

Supplementary Fig. 121. GFP flow cytometry data for a population of cells containing the synthetic perceptgene based on ANF loops. In this circuit, P_{lacO} within the ANF was replaced by P_{lacO1} and AraC truncated was used to improve the compatibility of Arabinose and IPTG (Fig.1h). **(a)** Arabinose was held constant at 0.04 mM, IPTG was held constant at 3.906 μM, and aTc was varied. **(b)** Arabinose was held constant at 0.04 mM, aTc was held constant at 1.563 ng/ml, and IPTG was varied. Source data are available in the Source data file.

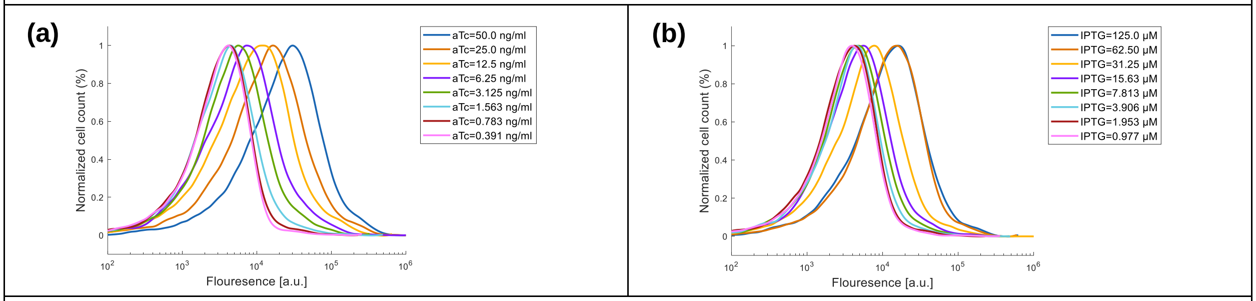

Supplementary Fig. 122. GFP flow cytometry data for a population of cells containing the synthetic perceptgene based on ANF loops. In this circuit, P_{lacO} within the ANF was replaced by P_{lacO1} and AraC truncated was used to improve the compatibility of Arabinose and IPTG (Fig. 1h). **(a)** Arabinose was held constant at 0.04 mM, IPTG was held constant at 1.953 μM, and aTc was varied. **(b)** Arabinose was held constant at 0.04 mM, aTc was held constant at 0.783 ng/ml, and IPTG was varied. Source data are available in the Source data file.

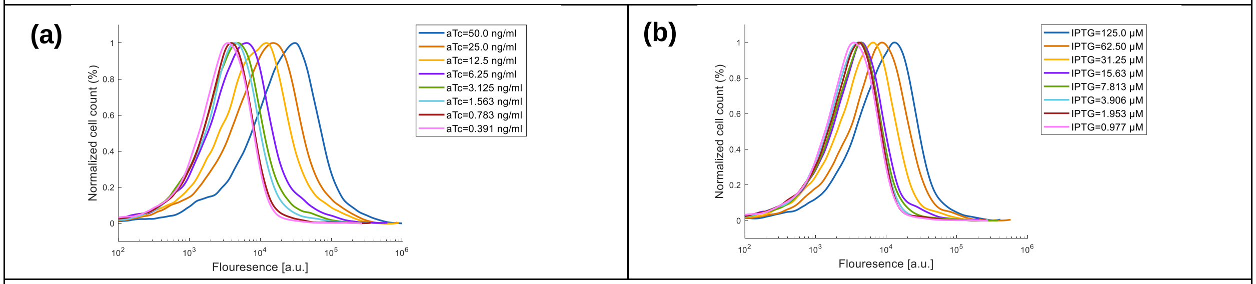

Supplementary Fig. 123. GFP flow cytometry data for a population of cells containing the synthetic perceptgene based on ANF loops. In this circuit, P_{lacO} within the ANF was replaced by P_{lacO1} and AraC truncated was used to improve the compatibility of Arabinose and IPTG (Fig. 1h). **(a)** Arabinose was held constant at 0.04 mM, IPTG was held constant at 0.977 μM, and aTc was varied. **(b)** Arabinose was held constant at 0.04 mM, aTc was held constant at 0.391 ng/ml, and IPTG was varied. Source data are available in the Source data file.

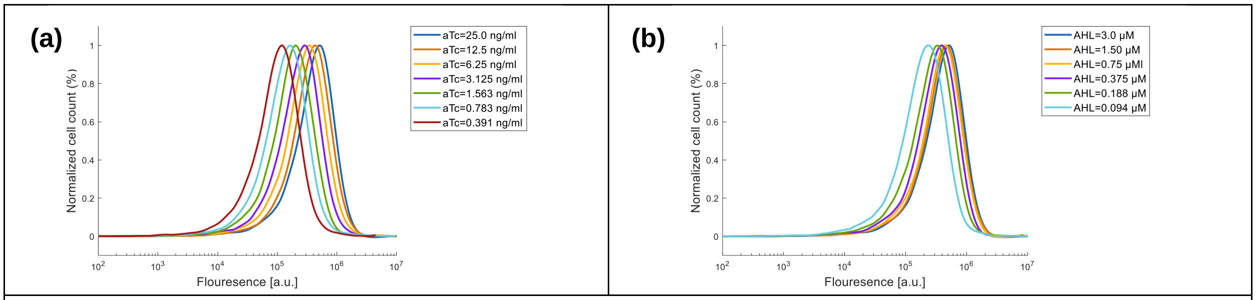

Supplementary Fig. 124. GFP flow cytometry data for a population of cells containing the synthetic perceptgene based on ANF and APF loops. In this circuit, P_{tetO} promoter was regulated by TetR through ANF loop and mutated P_{luxTGT} promoter was regulated by LuxR through APF loop (Fig. 2b). **(a)** AHL was held constant at 3.0 μM and aTc was varied. **(b)** aTc was held constant at 25 ng/ml and AHL was varied. Source data are available in the Source data file.

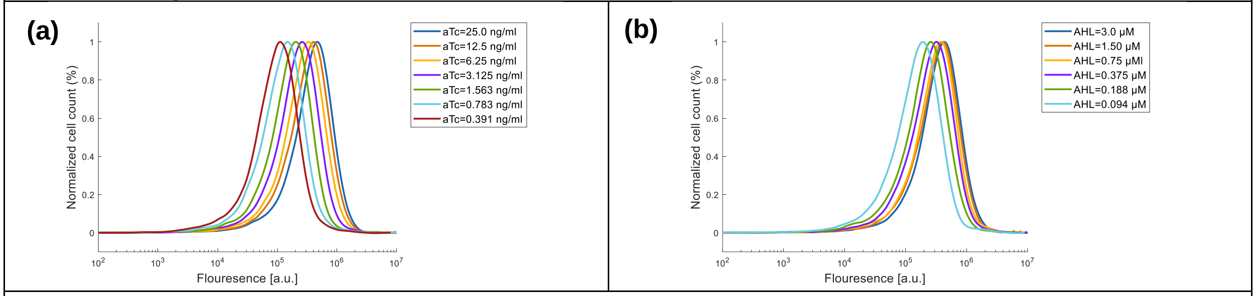

Supplementary Fig. 125. GFP flow cytometry data for a population of cells containing the synthetic perceptgene based on ANF and APF loops. In this circuit, P_{tetO} promoter was regulated by TetR through ANF loop and mutated P_{luxTGT} promoter was regulated by LuxR through APF loop (Fig. 2b). **(a)** AHL was held constant at 1.50 μM and aTc was varied. **(b)** aTc was held constant at 12.5 ng/ml and AHL was varied. Source data are available in the Source data file.

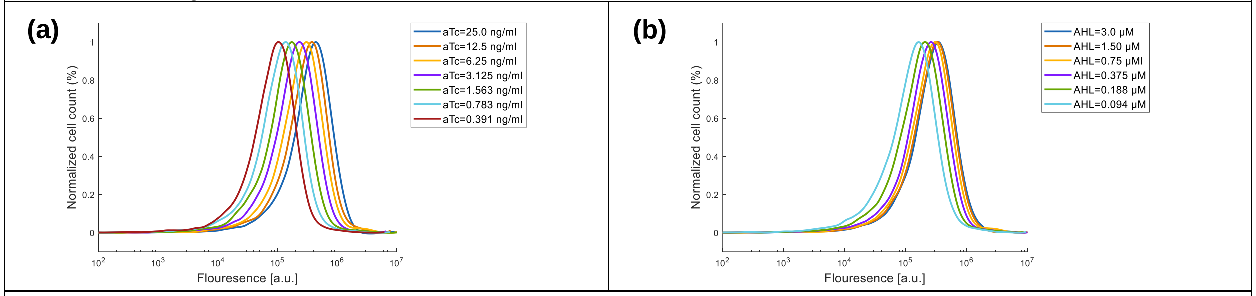

Supplementary Fig. 126. GFP flow cytometry data for a population of cells containing the synthetic perceptgene based on ANF and APF loops. In this circuit, P_{tetO} promoter was regulated by TetR through ANF loop and mutated P_{luxTGT} promoter was regulated by LuxR through APF loop (Fig. 2b). **(a)** AHL was held constant at 0.75 μM and aTc was varied. **(b)** aTc was held constant at 6.25 ng/ml and AHL was varied. Source data are available in the Source data file.

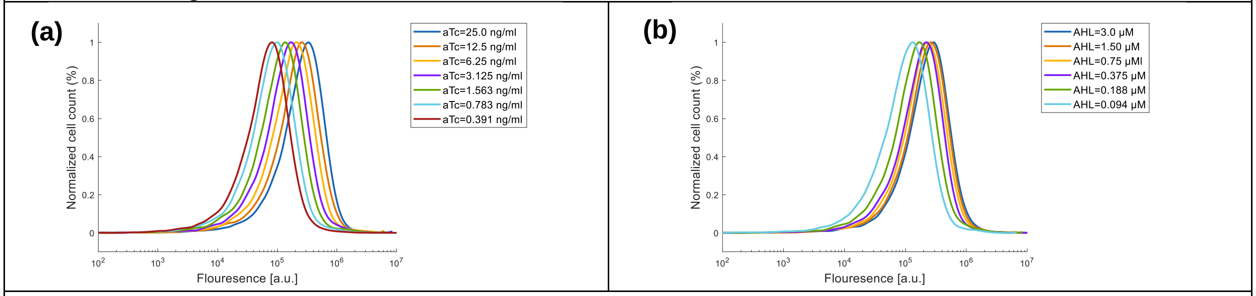

Supplementary Fig. 127. GFP flow cytometry data for a population of cells containing the synthetic perceptgene based on ANF and APF loops. In this circuit, P_{tetO} promoter was regulated by TetR through ANF loop and mutated P_{luxTGT} promoter was regulated by LuxR through APF loop (Fig. 2b). **(a)** AHL was held constant at 0.375 μM and aTc was varied. **(b)** aTc was held constant at 3.125 ng/ml and AHL was varied. Source data are available in the Source data file.

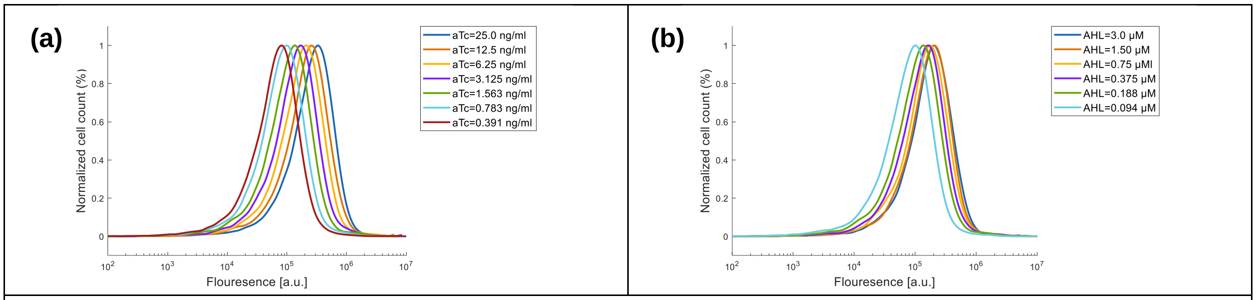

Supplementary Fig. 128. GFP flow cytometry data for a population of cells containing the synthetic perceptgene based on ANF and APF loops. In this circuit, P_{tetO} promoter was regulated by TetR through ANF loop and mutated P_{luxTGT} promoter was regulated by LuxR through APF loop (Fig. 2b). **(a)** AHL was held constant at 0.188 μM and aTc was varied. **(b)** aTc was held constant at 1.563 ng/ml and AHL was varied. Source data are available in the Source data file.

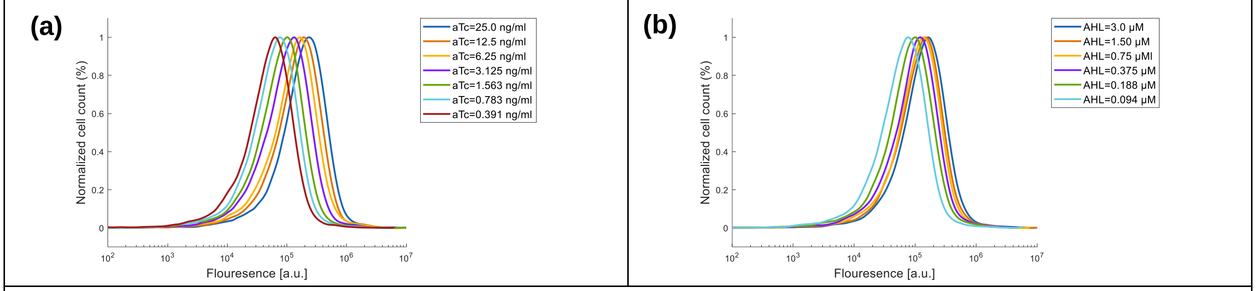

Supplementary Fig. 129. GFP flow cytometry data for a population of cells containing the synthetic perceptgene based on ANF and APF loops. In this circuit, P_{tetO} promoter was regulated by TetR through ANF loop and mutated P_{luxTGT} promoter was regulated by LuxR through APF loop (Fig. 2b). **(a)** AHL was held constant at 0.094 μM and aTc was varied. **(b)** aTc was held constant at 0.783 ng/ml and AHL was varied. Source data are available in the Source data file.

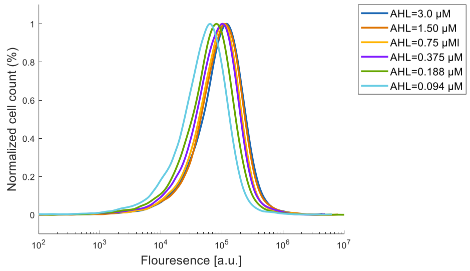

Supplementary Fig. 130. GFP flow cytometry data for a population of cells containing the synthetic perceptgene based on ANF and APF loops. In this circuit, P_{tetO} promoter was regulated by TetR through ANF loop and mutated P_{luxTGT} promoter was regulated by LuxR through APF loop (Fig. 2b). aTc was held constant at 0.0.391 ng/ml and AHL was varied. Source data are available in the Source data file.

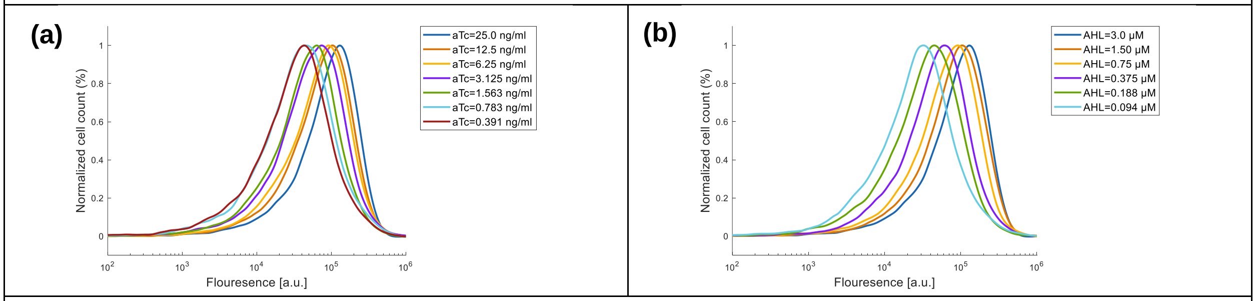

Supplementary Fig. 131. GFP flow cytometry data for a population of cells containing the synthetic perceptgene based on ANF and APF loops. In this circuit, P_{tetO} promoter was regulated by TetR through ANF loop and mutated P_{luxTGT} promoter was regulated by LuxR through APF loop. The output of the power-law and multiplication function was replaced by AraC activator, which regulate P_{BAD} promote (Fig. 2e). **(a)** Arabinose was held constant at 0.5 mM, AHL was held constant at 3.0 μM and aTc was varied. **(b)** Arabinose was held constant at 0.5 mM, aTc was held constant at 25 ng/ml and AHL was varied. Source data are available in the Source data file.

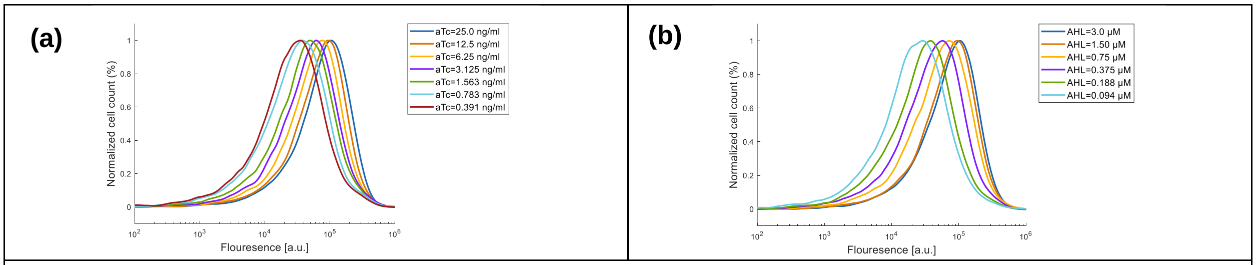

Supplementary Fig. 132. GFP flow cytometry data for a population of cells containing the synthetic perceptgene based on ANF and APF loops. In this circuit, P_{tetO} promoter was regulated by TetR through ANF loop and mutated P_{luxTGT} promoter was regulated by LuxR through APF loop. The output of the power-law and multiplication function was replaced by AraC activator, which regulate P_{BAD} promoter (Fig. 2e). **(a)** Arabinose was held constant at 0.5 mM, AHL was held constant at 1.5 μM and aTc was varied. **(b)** Arabinose was held constant at 0.5 mM, aTc was held constant at 12.5 ng/ml and AHL was varied. Source data are available in the Source data file.

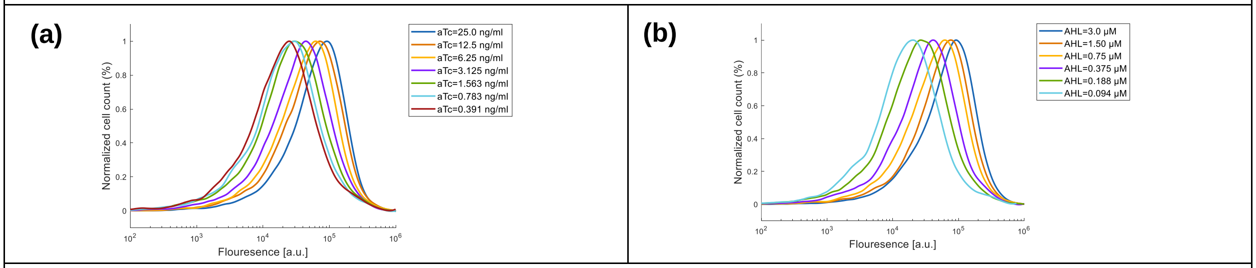

Supplementary Fig. 133. GFP flow cytometry data for a population of cells containing the synthetic perceptgene based on ANF and APF loops. In this circuit, P_{tetO} promoter was regulated by TetR through ANF loop and mutated P_{luxTGT} promoter was regulated by LuxR through APF loop. The output of the power-law and multiplication function was replaced by AraC activator, which regulate P_{BAD} promoter (Fig. 2e). **(a)** Arabinose was held constant at 0.5 mM, AHL was held constant at 0.75 μM and aTc was varied. **(b)** Arabinose was held constant at 0.5 mM, aTc was held constant at 6.25 ng/ml and AHL was varied.

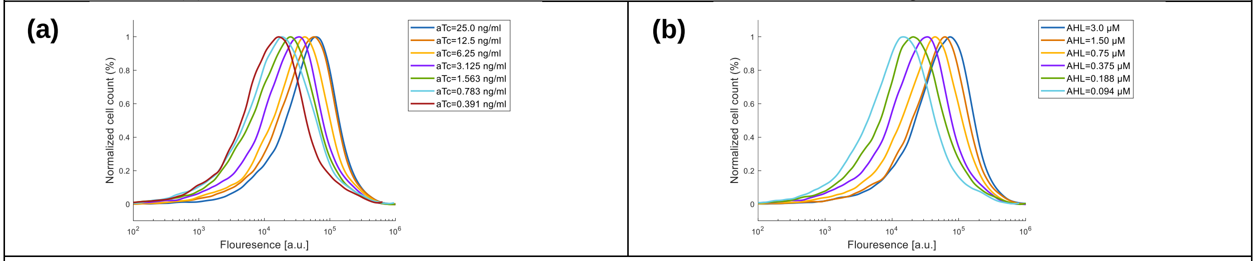

Supplementary Fig. 134. GFP flow cytometry data for a population of cells containing the synthetic perceptgene based on ANF and APF loops. In this circuit, P_{tetO} promoter was regulated by TetR through ANF loop and mutated P_{luxTGT} promoter was regulated by LuxR through APF loop. The output of the power-law and multiplication function was replaced by AraC activator, which regulate P_{BAD} promoter (Fig. 2e). **(a)** Arabinose was held constant at 0.5 mM, AHL was held constant at 0.375 μM and aTc was varied. **(b)** Arabinose was held constant at 0.5 mM, aTc was held constant at 3.125 ng/ml and AHL was varied. Source data are available in the Source data file.

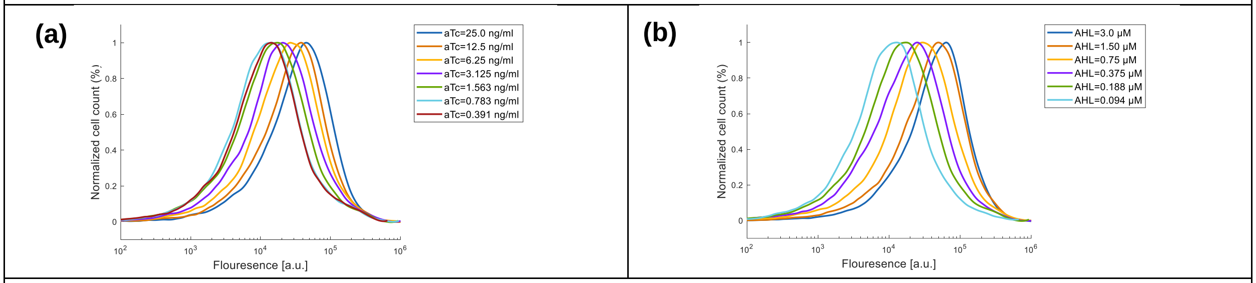

Supplementary Fig. 135. GFP flow cytometry data for a population of cells containing the synthetic perceptgene based on ANF and APF loops. In this circuit, P_{tetO} promoter was regulated by TetR through ANF loop and mutated P_{luxTGT} promoter was regulated by LuxR through APF loop. The output of the power-law and multiplication function was replaced by AraC activator, which regulate P_{BAD} promoter (Fig. 2e). **(a)** Arabinose was held constant at 0.5 mM, AHL was held constant at 0.188 μM and aTc was varied. **(b)** Arabinose was held constant at 0.5 mM, aTc was held constant at 1.563 ng/ml and AHL was varied. Source data are available in the Source data file.

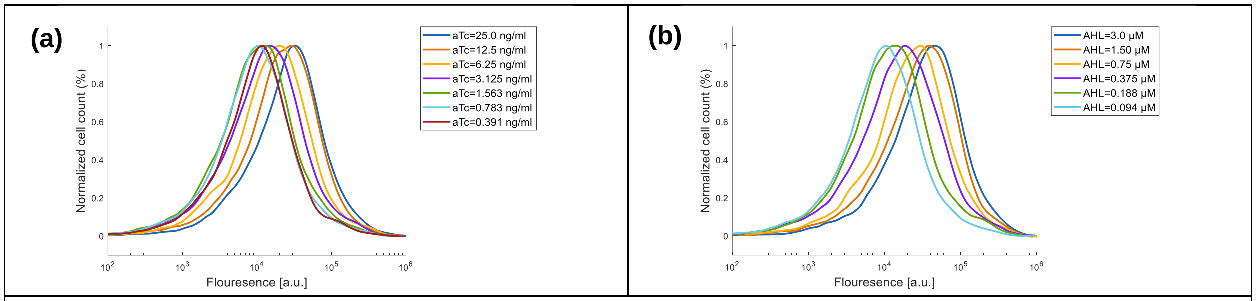

Supplementary Fig. 136. GFP flow cytometry data for a population of cells containing the synthetic perceptgene based on ANF and APF loops. In this circuit, P_{tetO} promoter was regulated by TetR through ANF loop and mutated P_{luxTGT} promoter was regulated by LuxR through APF loop. The output of the power-law and multiplication function was replaced by AraC activator, which regulate P_{BAD} promoter (Fig. 2e). **(a)** Arabinose was held constant at 0.5 mM, AHL was held constant at 0.094 μM and aTc was varied. **(b)** Arabinose was held constant at 0.5 mM, aTc was held constant at 0.783 ng/ml and AHL was varied. Source data are available in the Source data file.

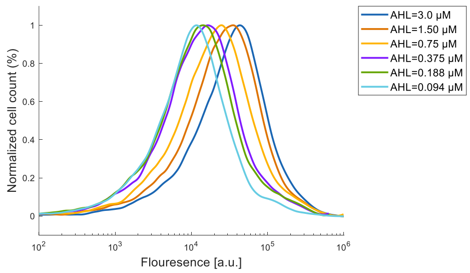

Supplementary Fig. 137. GFP flow cytometry data for a population of cells containing the synthetic perceptgene based on ANF and APF loops. In this circuit, P_{tetO} promoter was regulated by TetR through ANF loop and mutated P_{luxTGT} promoter was regulated by LuxR through APF loop. The output of the power-law and multiplication function was replaced by AraC activator, which regulate P_{BAD}D promoter (Fig. 2e). Arabinose was held constant at 0.5 mM, aTc was held constant at 0.391 ng/ml and AHL was varied. Source data are available in the Source data file.

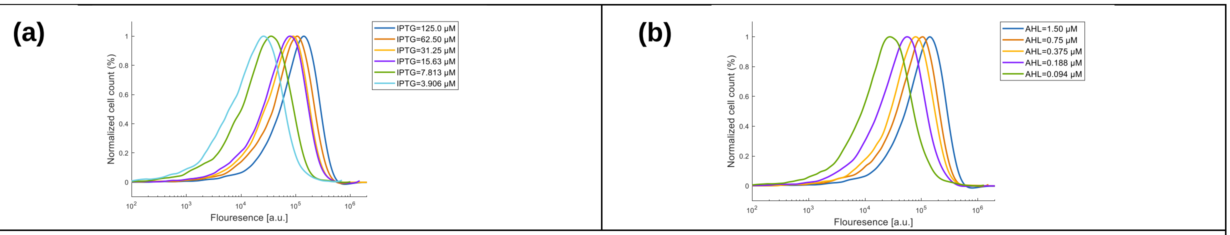

Supplementary Fig. 138. GFP flow cytometry data for a population of cells containing the synthetic perceptgene based on ANF and APF loops. In this circuit, synthetic average-meter based on perceptgene model was calculated using P_{lacO1} promoter, mutated P_{luxAAT} promoter and a combinatorial promoter ($P_{lux/tetO}$). P_{BAD} was used to set the logistic curve of the analog inputs (Fig. 2h). **(a)** Arabinose was held constant at 0.5 mM, AHL was held constant at 1.5 μM and IPTG was varied. **(b)** Arabinose was held constant at 0.5 mM, IPTG was held constant at 125 μM and AHL was varied. Source data are available in the Source data file.

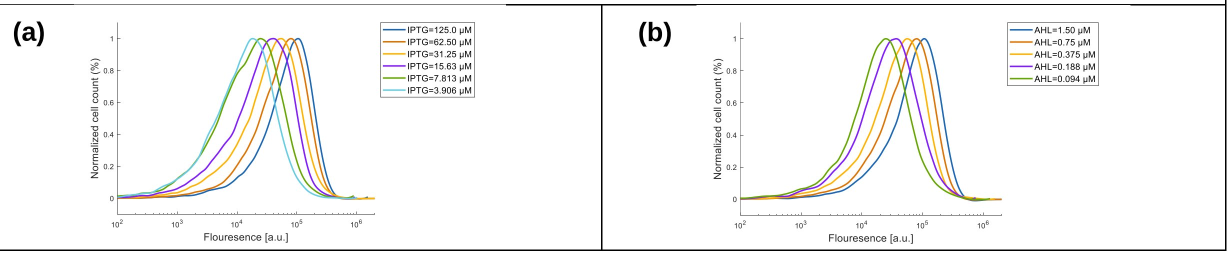

Supplementary Fig. 139. GFP flow cytometry data for a population of cells containing the synthetic perceptgene based on ANF and APF loops. In this circuit, synthetic average-meter based on perceptgene model was calculated using P_{lacO1} promoter, mutated P_{luxAAT} promoter and a combinatorial promoter ($P_{lux/tetO}$). P_{BAD} was used to set the logistic curve of the analog inputs (Fig. 2h). **(a)** Arabinose was held constant at 0.5 mM, AHL was held constant at 0.75 μM and IPTG was varied. **(b)** Arabinose was held constant at 0.5 mM, IPTG was held constant at 62.5 μM and AHL was varied. Source data are available in the Source data file.

(a)

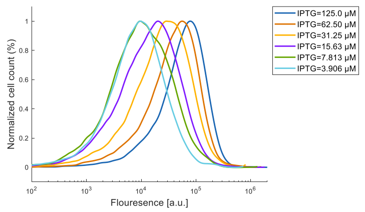

(b)

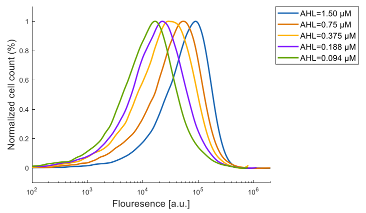

Supplementary Fig. 140. GFP flow cytometry data for a population of cells containing the synthetic perceptgene based on ANF and APF loops. In this circuit, synthetic average-meter based on perceptgene model was calculated using P_{lacO1} promoter, mutated P_{luxAAT} promoter and a combinatorial promoter ($P_{lux/tetO}$). P_{BAD} was used to set the logistic curve of the analog inputs (Fig. 2h). **(a)** Arabinose was held constant at 0.5 mM, AHL was held constant at 0.375 μM and IPTG was varied. **(b)** Arabinose was held constant at 0.5 mM, IPTG was held constant at 31.25 μM and AHL was varied. Source data are available in the Source data file.

(a)

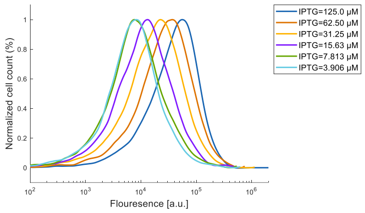

(b)

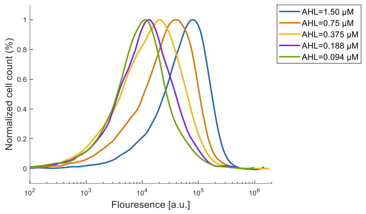

Supplementary Fig. 141. GFP flow cytometry data for a population of cells containing the synthetic perceptgene based on ANF and APF loops. In this circuit, synthetic average-meter based on perceptgene model was calculated using P_{lacO1} promoter, mutated P_{luxAAT} promoter and a combinatorial promoter ($P_{lux/tetO}$). P_{BAD} was used to set the logistic curve of the analog inputs (Fig. 2h). **(a)** Arabinose was held constant at 0.5 mM, AHL was held constant at 0.188 μM and IPTG was varied. **(b)** Arabinose was held constant at 0.5 mM, IPTG was held constant at 15.63 μM and AHL was varied. Source data are available in the Source data file.

(a)

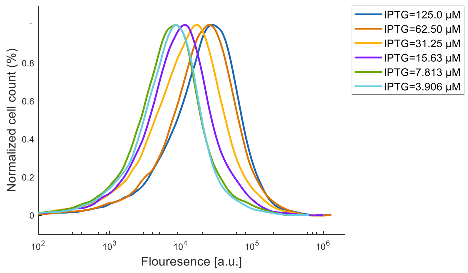

(b)

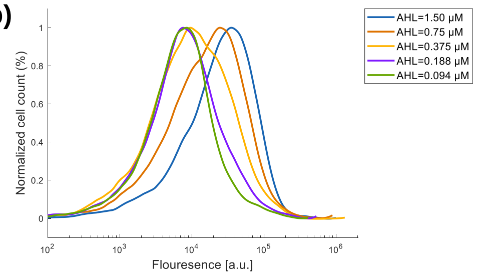

Supplementary Fig. 142. GFP flow cytometry data for a population of cells containing the synthetic perceptgene based on ANF and APF loops. In this circuit, synthetic average-meter based on perceptgene model was calculated using P_{lacO1} promoter, mutated P_{luxAAT} promoter and a combinatorial promoter ($P_{lux/tetO}$). P_{BAD} was used to set the logistic curve of the analog inputs (Fig. 2h). **(a)** Arabinose was held constant at 0.5 mM, AHL was held constant at 0.094 μM and IPTG was varied. **(b)** Arabinose was held constant at 0.5 mM, IPTG was held constant at 7.813 μM and AHL was varied. Source data are available in the Source data file.

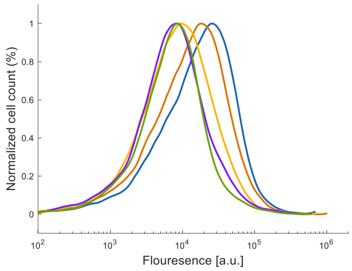

Supplementary Fig. 143. GFP flow cytometry data for a population of cells containing the synthetic perceptgene based on ANF and APF loops. In this circuit, synthetic average-meter based on perceptgene model was calculated using P_{lacO1} promoter, mutated P_{luxAAT} promoter and a combinatorial promoter ($P_{\mathrm{lux/tetO}}$). P_{BAD} was used to set the logistic curve of the analog inputs (Fig. 2h). Arabinose was held constant at 0.5 mM, IPTG was held constant at 3.906 μM and AHL was varied. Source data are available in the Source data file.

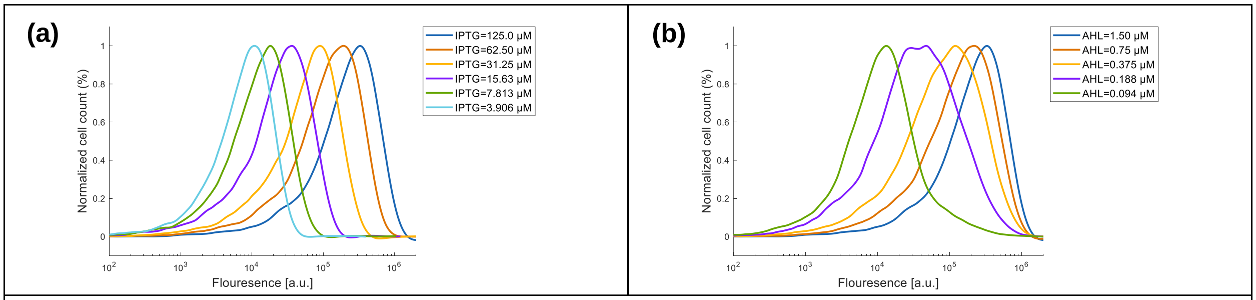

Supplementary Fig. 144. GFP flow cytometry data for a population of cells containing the synthetic perceptgene based on ANF and APF loops. In this circuit, P_{lacO1} promoter was regulated by LacI through ANF loop and mutated P_{luxAAT} promoter was regulated by LuxR through APF loop (Supplementary Fig. 37 and Fig. 39). **(a)** Arabinose was held constant at 0.5 mM, AHL was held constant at 1.5 μM and IPTG was varied. **(b)** Arabinose was held constant at 0.5 mM, IPTG was held constant at 125 μM and AHL was varied. Source data are available in the Source data file.

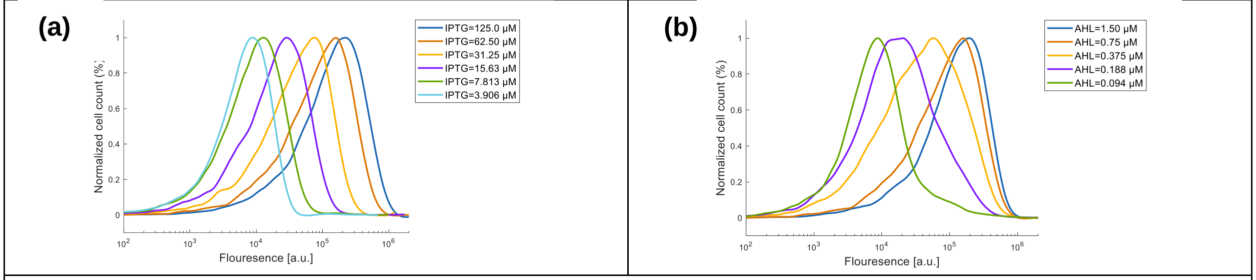

Supplementary Fig. 145. GFP flow cytometry data for a population of cells containing the synthetic perceptgene based on ANF and APF loops. In this circuit, P_{lacO1} promoter was regulated by LacI through ANF loop and mutated P_{luxAAT} promoter was regulated by LuxR through APF loop (Supplementary Fig. 37 and Fig. 39). **(a)** Arabinose was held constant at 0.5 mM, AHL was held constant at 0.75 μM and IPTG was varied. **(b)** Arabinose was held constant at 0.5 mM, IPTG was held constant at 62.5 μM and AHL was varied. Source data are available in the Source data file.

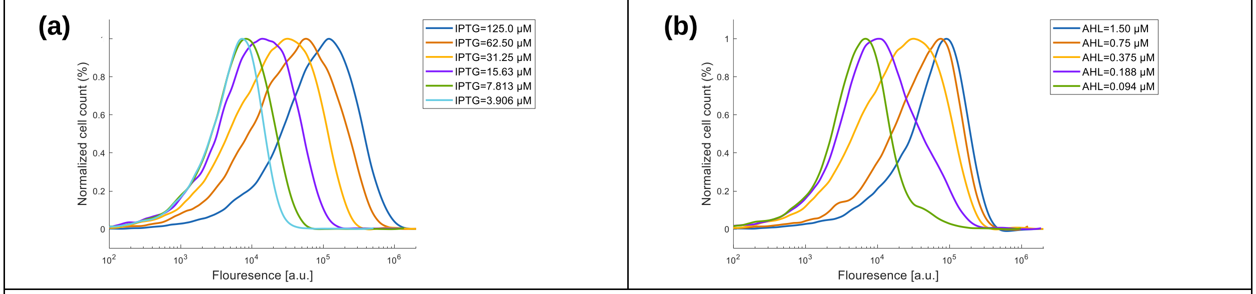

Supplementary Fig. 146. GFP flow cytometry data for a population of cells containing the synthetic perceptgene based on ANF and APF loops. In this circuit, P_{lacO1} promoter was regulated by LacI through ANF loop and mutated P_{luxAAT} promoter was regulated by LuxR through APF loop (Supplementary Fig. 37 and Fig. 39). **(a)** Arabinose was held constant at 0.5 mM, AHL

was held constant at 0.375 μM and IPTG was varied. **(b)** Arabinose was held constant at 0.5 mM, IPTG was held constant at 31.25 μM and AHL was varied. Source data are available in the Source data file.

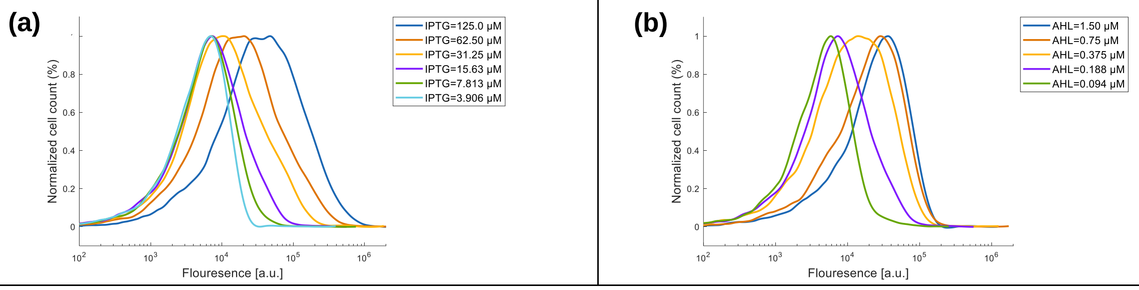

Supplementary Fig. 147. GFP flow cytometry data for a population of cells containing the synthetic perceptgene based on ANF and APF loops. In this circuit, P_{lacO1} promoter was regulated by LacI through ANF loop and mutated P_{luxAAT} promoter was regulated by LuxR through APF loop (Supplementary Fig. 37 and Fig. 39). **(a)** Arabinose was held constant at 0.5 mM, AHL was held constant at 0.188 μM and IPTG was varied. **(b)** Arabinose was held constant at 0.5 mM, IPTG was held constant at 15.63 μM and AHL was varied. Source data are available in the Source data file.

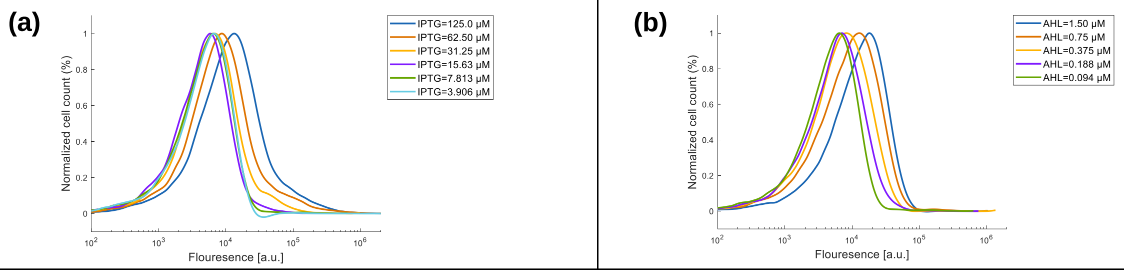

Supplementary Fig. 148. GFP flow cytometry data for a population of cells containing the synthetic perceptgene based on ANF and APF loops. In this circuit, P_{lacO1} promoter was regulated by LacI through ANF loop and mutated P_{luxAAT} promoter was regulated by LuxR through APF loop (Supplementary Fig. 37 and Fig. 39). **(a)** Arabinose was held constant at 0.5 mM, AHL was held constant at 0.094 μM and IPTG was varied. **(b)** Arabinose was held constant at 0.5 mM, IPTG was held constant at 7.813 μM and AHL was varied. Source data are available in the Source data file.

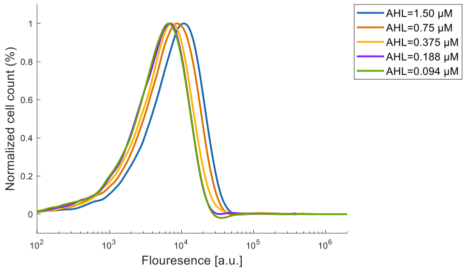

Supplementary Fig. 149. GFP flow cytometry data for a population of cells containing the synthetic perceptgene based on ANF and APF loops. In this circuit, P_{lacO1} promoter was regulated by LacI through ANF loop and mutated P_{luxAAT} promoter was regulated by LuxR through APF loop (Supplementary Fig. 37 and Fig. 39). Arabinose was held constant at 0.5 mM, IPTG was held constant at 3.906 μM and AHL was varied. Source data are available in the Source data file.

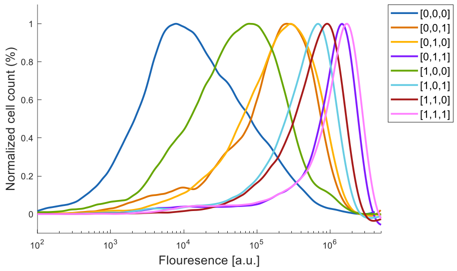

Supplementary Fig. 150. GFP flow cytometry data for a population of cells containing the synthetic multilayer perceptgene network (Fig. 3d). Measured response of majority circuit. AHL [0.1875, 0.3μM], IPTG [7.8125, 125μM], aTc [1.5625, 25ng/mL] and Arabinose [0.25mM]. Source data are available in the Source data file.

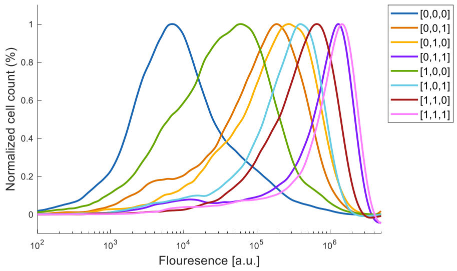

Supplementary Fig. 151. GFP flow cytometry data for a population of cells containing the synthetic multilayer perceptgene network (Fig. 3d). Measured response of majority circuit. AHL [0.1875, 0.3μM], IPTG [7.8125, 125μM], aTc [1.5625, 25ng/mL] and Arabinose [0.125 mM]. Source data are available in the Source data file.

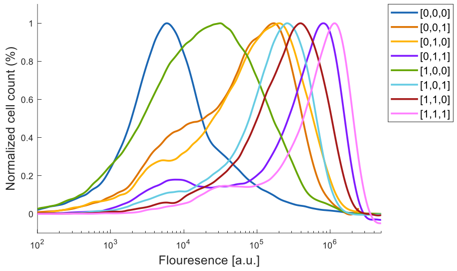

Supplementary Fig. 152. GFP flow cytometry data for a population of cells containing the synthetic multilayer perceptgene network (Fig. 3d). Measured response of majority circuit. AHL [0.1875, 0.3μM], IPTG [7.8125, 125μM], aTc [1.5625, 25ng/mL] and Arabinose [0.0625 mM]. Source data are available in the Source data file.

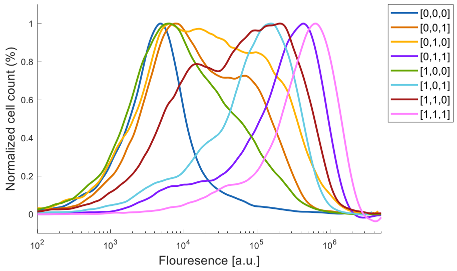

Supplementary Fig. 153. GFP flow cytometry data for a population of cells containing the synthetic multilayer perceptgene network (Fig. 3d). Measured response of majority circuit. AHL [0.1875, 0.3μM], IPTG [7.8125, 125μM], aTc [1.5625, 25ng/mL] and Arabinose [0.03125 mM]. Source data are available in the Source data file.

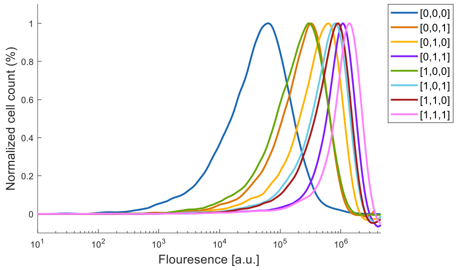

Supplementary Fig. 154. GFP flow cytometry data for a population of cells containing a 3-input perceptgene network and back propagation algorithm with TCTA Mutation (Fig. 3g and Supplementary Fig. 57b). AHL [0.1875, 0.3μM], IPTG [7.8125, 125μM], aTc [1.5625, 25ng/mL] and Arabinose [0.25mM].

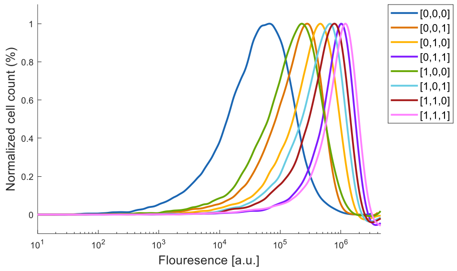

Supplementary Fig. 155. GFP flow cytometry data for a population of cells containing a 3-input perceptgene network and back propagation algorithm with TCTA Mutation (Fig. 3g and Supplementary Fig. 57b). AHL [0.1875, 0.3μM], IPTG [7.8125, 125μM], aTc [1.5625, 25ng/mL] and Arabinose [0.125mM].

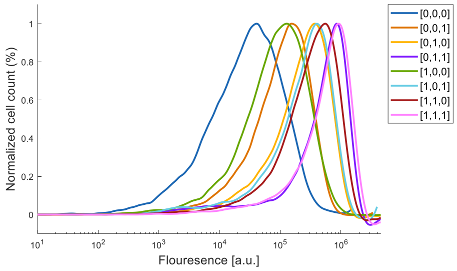

Supplementary Fig. 156. GFP flow cytometry data for a population of cells containing a 3-input perceptgene network and back propagation algorithm with TCTA Mutation (Fig. 3g and Supplementary Fig. 57b). AHL [0.1875, 0.3μM], IPTG [7.8125, 125μM], aTc [1.5625, 25ng/mL] and Arabinose [0.0625mM].

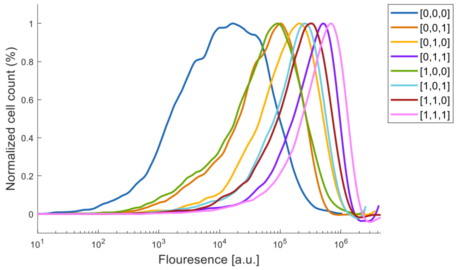

Supplementary Fig. 157. GFP flow cytometry data for a population of cells containing a 3-input perceptgene network and back propagation algorithm with TCTA Mutation (Fig. 3g and Supplementary Fig. 57b). AHL [0.1875, 0.3μM], IPTG [7.8125, 125μM], aTc [1.5625, 25ng/mL] and Arabinose [0.03125 mM].

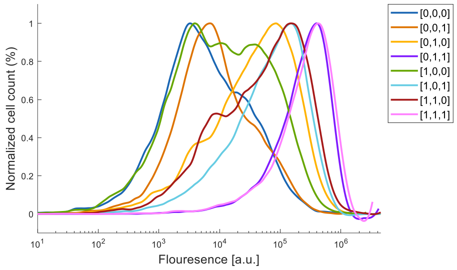

Supplementary Fig. 158. GFP flow cytometry data for a population of cells containing a 3-input perceptgene network and back propagation algorithm with TCTA Mutation (Fig. 3g and Supplementary Fig. 57b). AHL [0.1875, 0.3μM], IPTG [7.8125, 125μM], aTc [1.5625, 25ng/mL] and Arabinose [0.015625 mM].

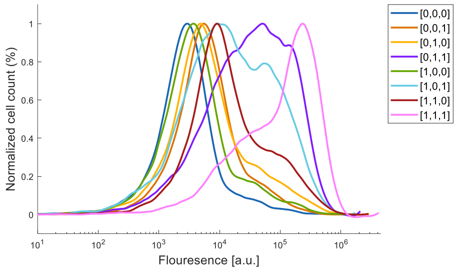

Supplementary Fig. 159. GFP flow cytometry data for a population of cells containing flow cytometry data for a population of cells containing a 3-input perceptgene network and back propagation algorithm with TCTA Mutation (Fig. 3g and Supplementary Fig. 57b). AHL [0.1875, 0.3μM], IPTG [7.8125, 125μM], aTc [1.5625, 25ng/mL] and Arabinose [0.0078125mM].

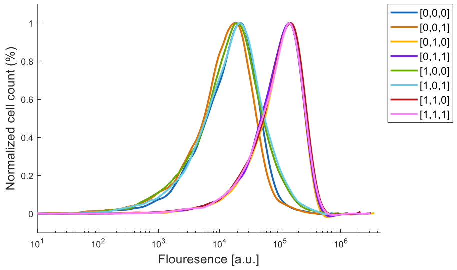

Supplementary Fig. 160. GFP flow cytometry data for a population of cells containing the first perceptgene layer with 2-input from the 3-input network with TCTA Mutation (Supplementary Fig. 59b). AHL [0.1875, 0.3μM], IPTG [7.8125, 125μM], aTc [1.5625, 25ng/mL] and Arabinose [0.25mM].

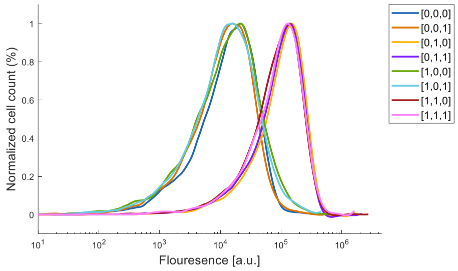

Supplementary Fig. 161. GFP flow cytometry data for a population of cells containing the first perceptgene layer with 2-input from the 3-input network with TCTA Mutation (Supplementary Fig. 59b). AHL [0.1875, 0.3μM], IPTG [7.8125, 125μM], aTc [1.5625, 25ng/mL] and Arabinose [0.125mM].

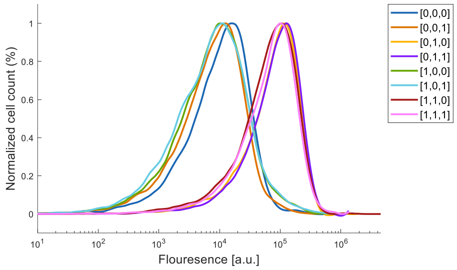

Supplementary Fig. 162. GFP flow cytometry data for a population of cells containing the first perceptgene layer with 2-input from the 3-input network with TCTA Mutation (Supplementary Fig. 59b). AHL [0.1875, 0.3μM], IPTG [7.8125, 125μM], aTc [1.5625, 25ng/mL] and Arabinose [0.0625mM].

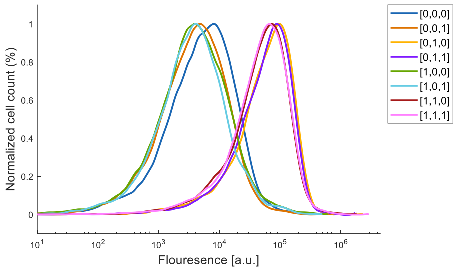

Supplementary Fig. 163. GFP flow cytometry data for a population of cells containing the first perceptgene layer with 2-input from the 3-input network with TCTA Mutation (Supplementary Fig. 59b). AHL [0.1875, 0.3μM], IPTG [7.8125, 125μM], aTc [1.5625, 25ng/mL] and Arabinose [0.03125mM].

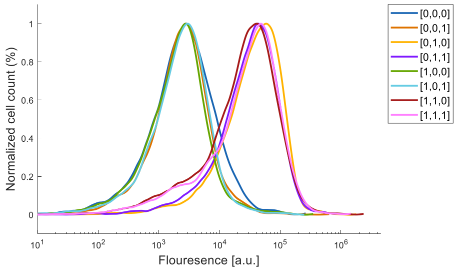

Supplementary Fig. 164. GFP flow cytometry data for a population of cells containing the first perceptgene layer with 2-input from the 3-input network with TCTA Mutation (Supplementary Fig. 59b). AHL [0.1875, 0.3μM], IPTG [7.8125, 125μM], aTc [1.5625, 25ng/mL] and Arabinose [0.015625mM].

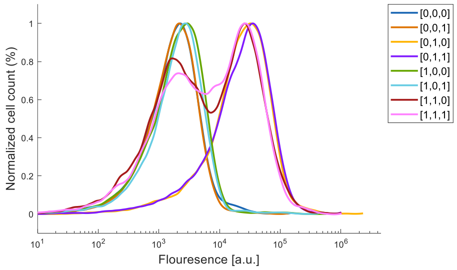

Supplementary Fig. 165. GFP flow cytometry data for a population of cells containing the first perceptgene layer with 2-input from the 3-input network with TCTA Mutation (Supplementary Fig. 59b). AHL [0.1875, 0.3μM], IPTG [7.8125, 125μM], aTc [1.5625, 25ng/mL] and Arabinose [0.0078125mM].

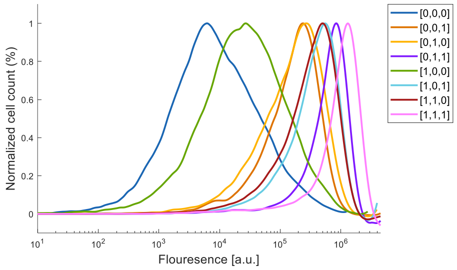

Supplementary Fig. 166. GFP flow cytometry data for a population of cells containing a 3-input perceptgene network and back propagation algorithm with GTTG Mutation (Fig. 3g and Supplementary Fig. 57d). AHL [0.1875, 0.3µM], IPTG [7.8125, 125µM], aTc [1.5625, 25ng/mL] and Arabinose [0.25mM].

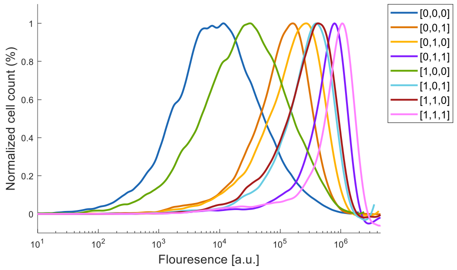

Supplementary Fig. 167. GFP flow cytometry data for a population of cells containing a 3-input perceptgene network and back propagation algorithm with GTTG Mutation (Fig. 3g and Supplementary Fig. 57d).). AHL [0.1875, 0.3µM], IPTG [7.8125, 125µM], aTc [1.5625, 25ng/mL] and Arabinose [0.125mM].

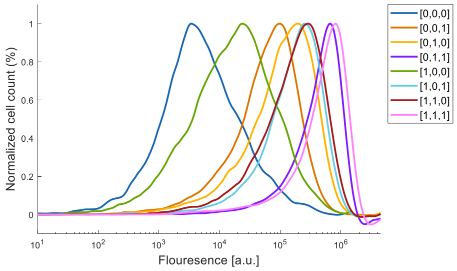

Supplementary Fig. 168. GFP flow cytometry data for a population of cells containing a 3-input perceptgene network and back propagation algorithm with GTTG Mutation (Fig. 3g and Supplementary Fig. 57d).). AHL [0.1875, 0.3µM], IPTG [7.8125, 125µM], aTc [1.5625, 25ng/mL] and Arabinose [0.0625mM].

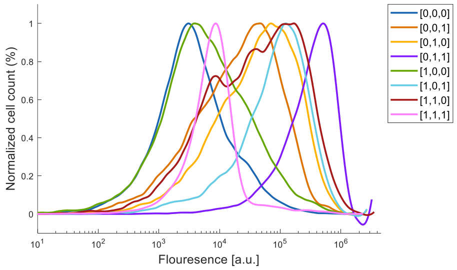

Supplementary Fig. 169. GFP flow cytometry data for a population of cells containing a 3-input perceptgene network and back propagation algorithm with GTTG Mutation (Fig. 3g and Supplementary Fig. 57d).). AHL [0.1875, 0.3µM], IPTG [7.8125, 125µM], aTc [1.5625, 25ng/mL] and Arabinose [0.03125 mM].

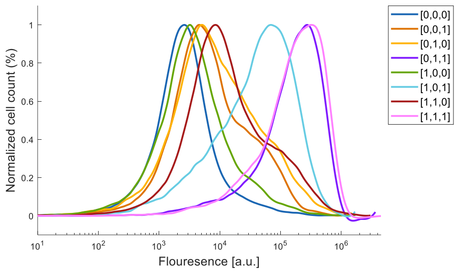

Supplementary Fig. 170. GFP flow cytometry data for a population of cells containing a 3-input perceptgene network and back propagation algorithm with GTTG Mutation (Fig. 3g and Supplementary Fig. 57d).). AHL [0.1875, 0.3μM], IPTG [7.8125, 125μM], aTc [1.5625, 25ng/mL] and Arabinose [0.015625 mM].

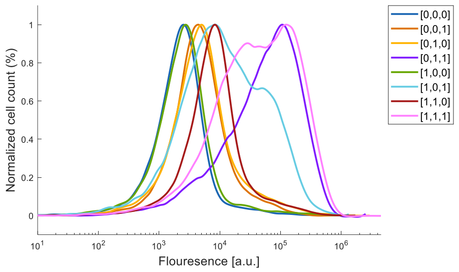

Supplementary Fig. 171. GFP flow cytometry data for a population of cells containing a 3-input perceptgene network and back propagation algorithm with GTTG Mutation (Fig. 3g and Supplementary Fig. 57d).). AHL [0.1875, 0.3μM], IPTG [7.8125, 125μM], aTc [1.5625, 25ng/mL] and Arabinose [0.0078125mM].

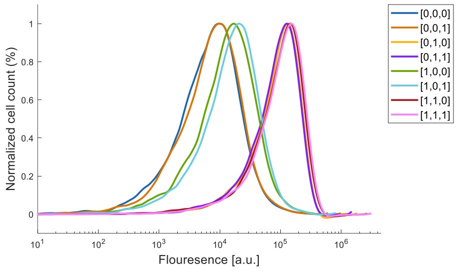

Supplementary Fig. 172. GFP flow cytometry data for a population of cells containing the first perceptgene layer with 2-input from the 3-input network with GTTG Mutation (Supplementary Fig. 59d). AHL [0.1875, 0.3μM], IPTG [7.8125, 125μM], aTc [1.5625, 25ng/mL] and Arabinose [0.25mM].

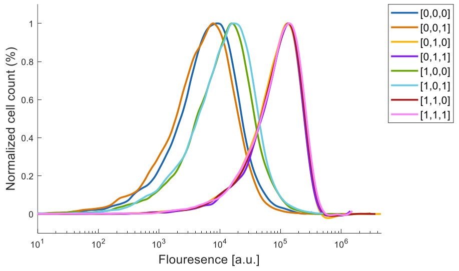

Supplementary Fig. 173. GFP flow cytometry data for a population of cells containing the first perceptgene layer with 2-input from the 3-input network with GTTG Mutation (Supplementary Fig. 59d). AHL [0.1875, 0.3μM], IPTG [7.8125, 125μM], aTc [1.5625, 25ng/mL] and Arabinose [0.125mM].

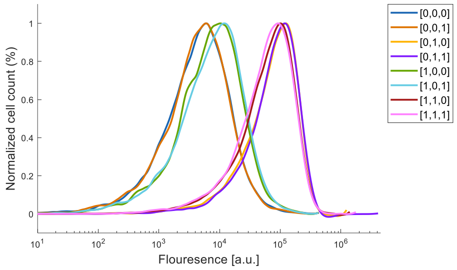

Supplementary Fig. 174. GFP flow cytometry data for a population of cells containing the first perceptgene layer with 2-input from the 3-input network with GTTG Mutation (Supplementary Fig. 59d). AHL [0.1875, 0.3µM], IPTG [7.8125, 125µM], aTc [1.5625, 25ng/mL] and Arabinose [0.0625mM].

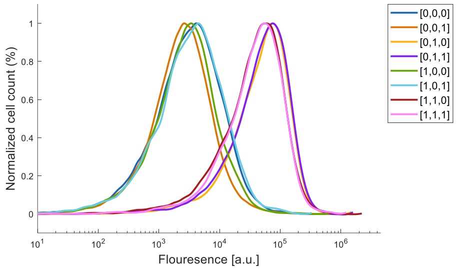

Supplementary Fig. 175. GFP flow cytometry data for a population of cells containing the first perceptgene layer with 2-input from the 3-input network with GTTG Mutation (Supplementary Fig. 59d). AHL [0.1875, 0.3µM], IPTG [7.8125, 125µM], aTc [1.5625, 25ng/mL] and Arabinose [0.03125mM].

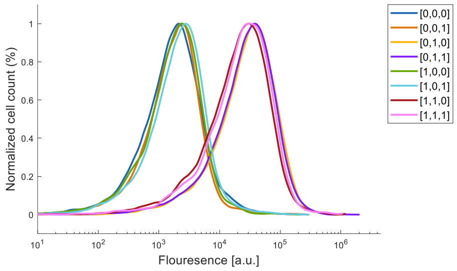

Supplementary Fig. 176. GFP flow cytometry data for a population of cells containing the first perceptgene layer with 2-input from the 3-input network with GTTG Mutation (Supplementary Fig. 59d AHL [0.1875, 0.3µM], IPTG [7.8125, 125µM], aTc [1.5625, 25ng/mL] and Arabinose [0.015625mM].

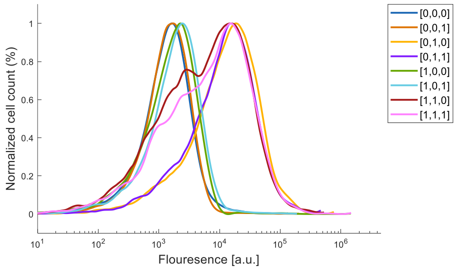

Supplementary Fig. 177. GFP flow cytometry data for a population of cells containing the first perceptgene layer with 2-input from the 3-input network with GTTG Mutation (Supplementary Fig. 59d). AHL [0.1875, 0.3µM], IPTG [7.8125, 125µM], aTc [1.5625, 25ng/mL] and Arabinose [0.0078125].

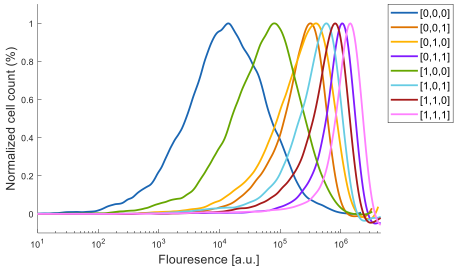

Supplementary Fig. 178. GFP flow cytometry data for a population of cells containing flow cytometry data for a population of cells containing a 3-input perceptgene network and back propagation algorithm with GAGC Mutation (Fig. 3g and Supplementary Fig. 57f). AHL [0.1875, 0.3μM], IPTG [7.8125, 125μM], aTc [1.5625, 25ng/mL] and Arabinose [0.25mM].

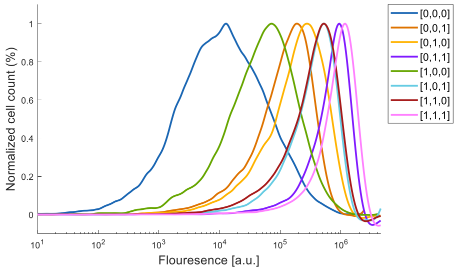

Supplementary Fig. 179. GFP flow cytometry data for a population of cells containing flow cytometry data for a population of cells containing a 3-input perceptgene network and back propagation algorithm with GAGC Mutation (Fig. 3g and Supplementary Fig. 57f).). AHL [0.1875, 0.3μM], IPTG [7.8125, 125μM], aTc [1.5625, 25ng/mL] and Arabinose [0.125mM].

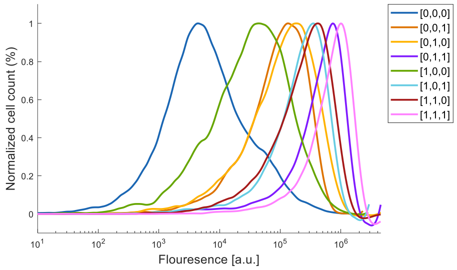

Supplementary Fig. 180. GFP flow cytometry data for a population of cells containing flow cytometry data for a population of cells containing a 3-input perceptgene network and back propagation algorithm with GAGC Mutation (Fig. 3g and Supplementary Fig. 57f).). AHL [0.1875, 0.3μM], IPTG [7.8125, 125μM], aTc [1.5625, 25ng/mL] and Arabinose [0.0625mM].

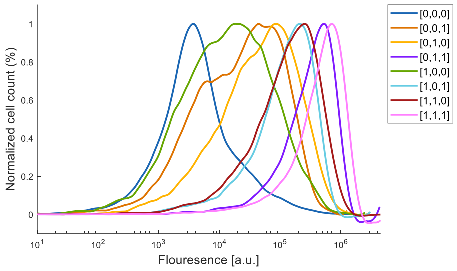

Supplementary Fig. 181. GFP flow cytometry data for a population of cells containing flow cytometry data for a population of cells containing a 3-input perceptgene network and back propagation algorithm with GAGC Mutation (Fig. 3g and Supplementary Fig. 57f).). AHL [0.1875, 0.3μM], IPTG [7.8125, 125μM], aTc [1.5625, 25ng/mL] and Arabinose [0.03125 mM].

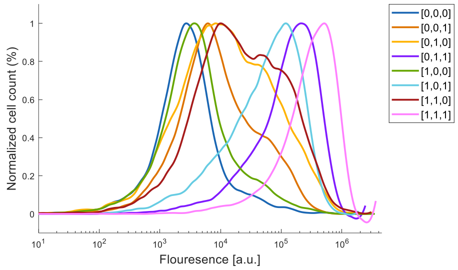

Supplementary Fig. 182. GFP flow cytometry data for a population of cells containing flow cytometry data for a population of cells containing a 3-input perceptgene network and back propagation algorithm with GAGC Mutation (Fig. 3g and Supplementary Fig. 57f).). AHL [0.1875, 0.3μM], IPTG [7.8125, 125μM], aTc [1.5625, 25ng/mL] and Arabinose [0.015625 mM].

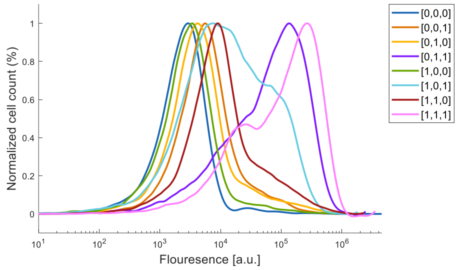

Supplementary Fig. 183. GFP flow cytometry data for a population of cells containing flow cytometry data for a population of cells containing a 3-input perceptgene network and back propagation algorithm with GAGC Mutation (Fig. 3g and Supplementary Fig. 57f).). AHL [0.1875, 0.3μM], IPTG [7.8125, 125μM], aTc [1.5625, 25ng/mL] and Arabinose [0.0078125mM].

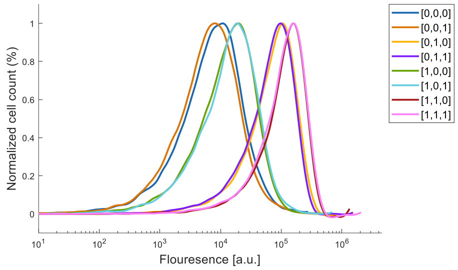

Supplementary Fig. 184. GFP flow cytometry data for a population of cells containing the first perceptgene layer with 2-input from the 3-input network with GAGC Mutation (Supplementary Fig. 59f). AHL [0.1875, 0.3μM], IPTG [7.8125, 125μM], aTc [1.5625, 25ng/mL] and Arabinose [0.25mM].

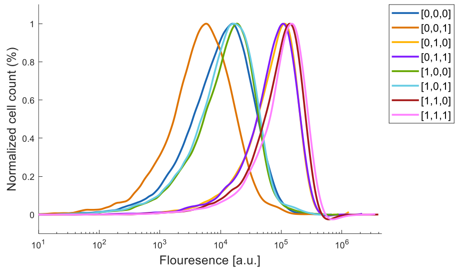

Supplementary Fig. 185. GFP flow cytometry data for a population of cells containing the first perceptgene layer with 2-input from the 3-input network with GAGC Mutation (Supplementary Fig. 59f). AHL [0.1875, 0.3μM], IPTG [7.8125, 125μM], aTc [1.5625, 25ng/mL] and Arabinose [0.125mM].

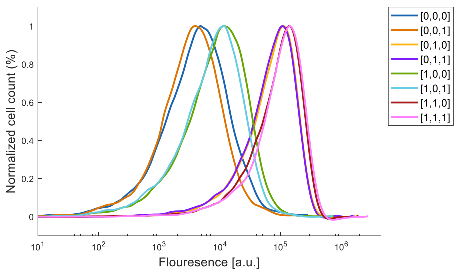

Supplementary Fig. 186. GFP flow cytometry data for a population of cells containing the first perceptgene layer with 2-input from the 3-input network with GAGC Mutation (Supplementary Fig. 59f). AHL [0.1875, 0.3μM], IPTG [7.8125, 125μM], aTc [1.5625, 25ng/mL] and Arabinose [0.0625mM].

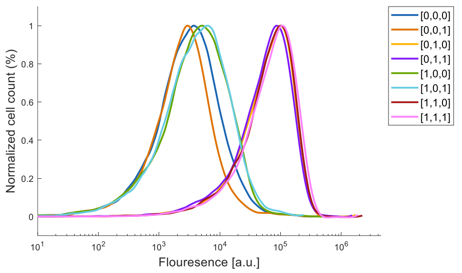

Supplementary Fig. 187. GFP flow cytometry data for a population of cells containing the first perceptgene layer with 2-input from the 3-input network with GAGC Mutation (Supplementary Fig. 59f). AHL [0.1875, 0.3μM], IPTG [7.8125, 125μM], aTc [1.5625, 25ng/mL] and Arabinose [0.03125mM].

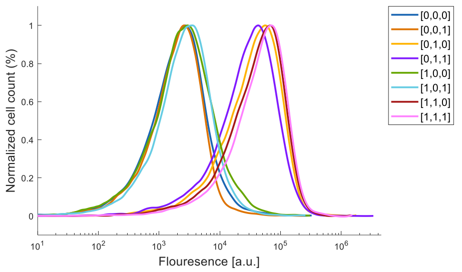

Supplementary Fig. 188. GFP flow cytometry data for a population of cells containing the first perceptgene layer with 2-input from the 3-input network with GAGC Mutation (Supplementary Fig. 59f). AHL [0.1875, 0.3μM], IPTG [7.8125, 125μM], aTc [1.5625, 25ng/mL] and Arabinose [0.015625mM].

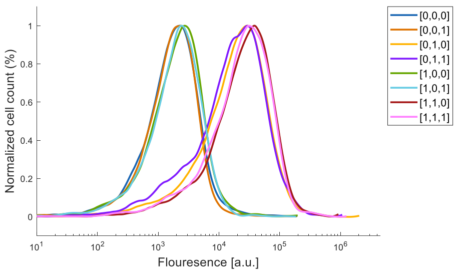

Supplementary Fig. 189. GFP flow cytometry data for a population of cells containing the first perceptgene layer with 2-input from the 3-input network with GAGC Mutation (Supplementary Fig. 59f). AHL [0.1875, 0.3μM], IPTG [7.8125, 125μM], aTc [1.5625, 25ng/mL] and Arabinose [0.0078125].

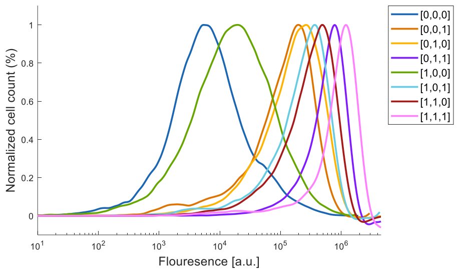

Supplementary Fig. 190. GFP flow cytometry data for a population of cells containing flow cytometry data for a population of cells containing a 3-input perceptgene network and back propagation algorithm with TGGG Mutation (Fig. 3g and Supplementary Fig. 57h). AHL [0.1875, 0.3μM], IPTG [7.8125, 125μM], aTc [1.5625, 25ng/mL] and Arabinose [0.25mM].

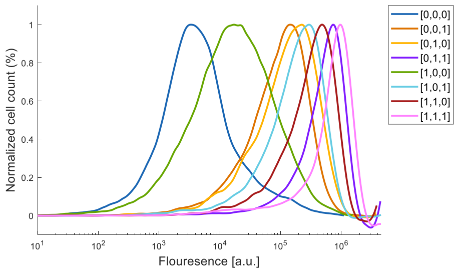

Supplementary Fig. 191. GFP flow cytometry data for a population of cells containing flow cytometry data for a population of cells containing a 3-input perceptgene network and back propagation algorithm with TGGG Mutation (Fig. 3g and Supplementary Fig. 57h). AHL [0.1875, 0.3μM], IPTG [7.8125, 125μM], aTc [1.5625, 25ng/mL] and Arabinose [0.125mM].

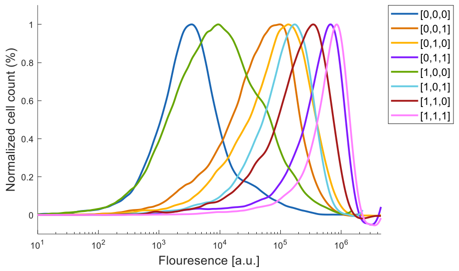

Supplementary Fig. 192. GFP flow cytometry data for a population of cells containing flow cytometry data for a population of cells containing a 3-input perceptgene network and back propagation algorithm with TGGG Mutation (Fig. 3g and Supplementary Fig. 57h). AHL [0.1875, 0.3μM], IPTG [7.8125, 125μM], aTc [1.5625, 25ng/mL] and Arabinose [0.0625mM].

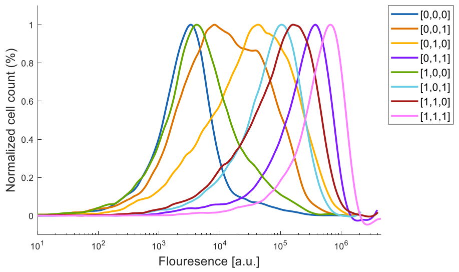

Supplementary Fig. 193. GFP flow cytometry data for a population of cells containing flow cytometry data for a population of cells containing a 3-input perceptgene network and back propagation algorithm with TGGG Mutation (Fig. 3g and Supplementary Fig. 57h). AHL [0.1875, 0.3μM], IPTG [7.8125, 125μM], aTc [1.5625, 25ng/mL] and Arabinose [0.03125 mM].

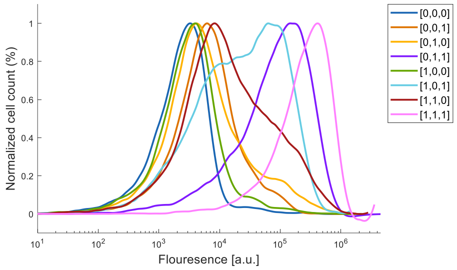

Supplementary Fig. 194. GFP flow cytometry data for a population of cells containing flow cytometry data for a population of cells containing a 3-input perceptgene network and back propagation algorithm with TGGG Mutation (Fig. 3g and Supplementary Fig. 57h). AHL [0.1875, 0.3μM], IPTG [7.8125, 125μM], aTc [1.5625, 25ng/mL] and Arabinose [0.015625 mM].

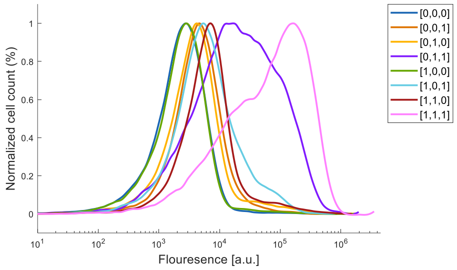

Supplementary Fig. 195. GFP flow cytometry data for a population of cells containing flow cytometry data for a population of cells containing a 3-input perceptgene network and back propagation algorithm with TGGG Mutation (Fig. 3g and Supplementary Fig. 57h). AHL [0.1875, 0.3μM], IPTG [7.8125, 125μM], aTc [1.5625, 25ng/mL] and Arabinose [0.0078125mM].

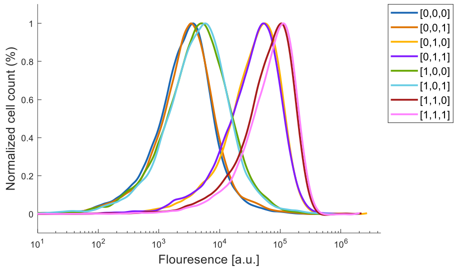

Supplementary Fig. 196. GFP flow cytometry data for a population of cells containing the first perceptgene layer with 2-input from the 3-input network with TGGG Mutation (Supplementary Fig. 59h). AHL [0.1875, 0.3μM], IPTG [7.8125, 125μM], aTc [1.5625, 25ng/mL] and Arabinose [0.25mM].

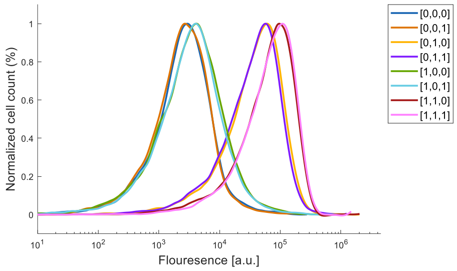

Supplementary Fig. 197. GFP flow cytometry data for a population of cells containing the first perceptgene layer with 2-input from the 3-input network with TGGG Mutation (Supplementary Fig. 59h). AHL [0.1875, 0.3μM], IPTG [7.8125, 125μM], aTc [1.5625, 25ng/mL] and Arabinose [0.125mM].

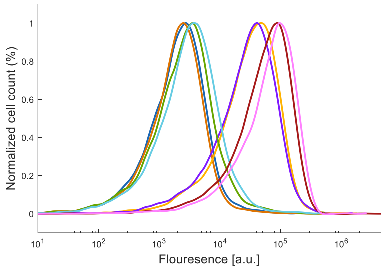

Supplementary Fig. 198. GFP flow cytometry data for a population of cells containing the first perceptgene layer with 2-input from the 3-input network with TGGG Mutation (Supplementary Fig. 59h). AHL [0.1875, 0.3µM], IPTG [7.8125, 125µM], aTc [1.5625, 25ng/mL] and Arabinose [0.0625mM].

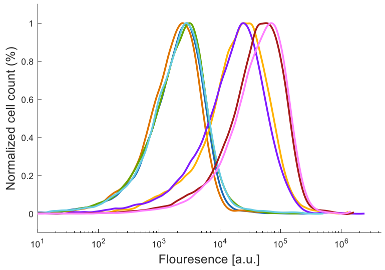

Supplementary Fig. 199. GFP flow cytometry data for a population of cells containing the first perceptgene layer with 2-input from the 3-input network with TGGG Mutation (Supplementary Fig. 59h). AHL [0.1875, 0.3µM], IPTG [7.8125, 125µM], aTc [1.5625, 25ng/mL] and Arabinose [0.03125mM].

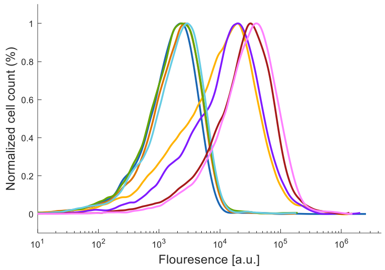

Supplementary Fig. 200. GFP flow cytometry data for a population of cells containing the first perceptgene layer with 2-input from the 3-input network with TGGG Mutation (Supplementary Fig. 59h). AHL [0.1875, 0.3µM], IPTG [7.8125, 125µM], aTc [1.5625, 25ng/mL] and Arabinose [0.015625mM].

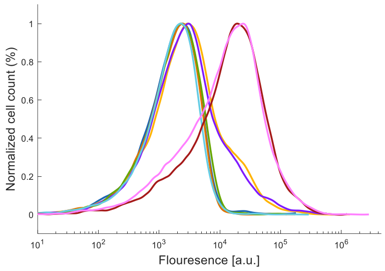

Supplementary Fig. 201. GFP flow cytometry data for a population of cells containing the first perceptgene layer with 2-input from the 3-input network with TGGG Mutation (Supplementary Fig. 59h). AHL [0.1875, 0.3µM], IPTG [7.8125, 125µM], aTc [1.5625, 25ng/mL] and Arabinose [0.0078125].

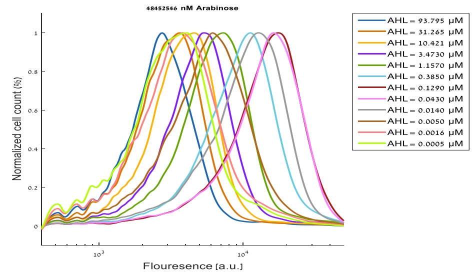

Supplementary Fig. 202. GFP flow cytometry data for a population of cells containing the genetic circuit to implement LSB using a forward P_{BAD} promoter and an antisense P_{ux} promoter (Supplementary Fig. 67a and Fig. 68).

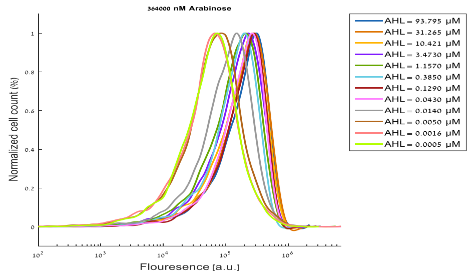

Supplementary Fig. 203. GFP flow cytometry data for a population of cells containing the genetic circuit to implement 2-bit ADC, using a graded PF that regulates P_{BAD} promoter and a combinatorial antisense $P_{lux/tetO}$, while $TetR$ repressor is regulated by MSB Circuit (Fig. 4c blue plot and Fig.4d).Source data are available in the Source data file.

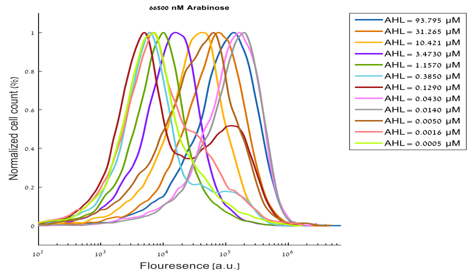

Supplementary Fig. 204. GFP flow cytometry data for a population of cells containing the genetic circuit to implement 2-bit hybrid ADC, where LSB circuit is built from two GFP signals: (1) Forward P_{BAD} promoter with antisense P_{lux} promoter and (2) the P_{rhlR} promoter (Fig. 4f and g). Source data are available in the Source data file.

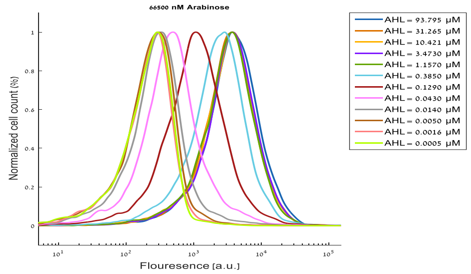

Supplementary Fig. 205. mCherry flow cytometry data for a population of cells containing the genetic circuit to implement 2-bit hybrid ADC. Where the P_{lux} of MSB circuit which is located on MCP, regulates the output mCherry signal (Fig. 4f and g). Source data are available in the Source data file.

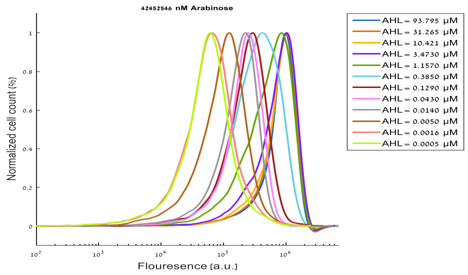

Supplementary Fig. 206. GFP flow cytometry data for a population of cells containing the genetic circuit to implement ternary data converter, based on the regulation of $TetR$ by MSB (Fig. 4h). Source data are available in the Source data file.

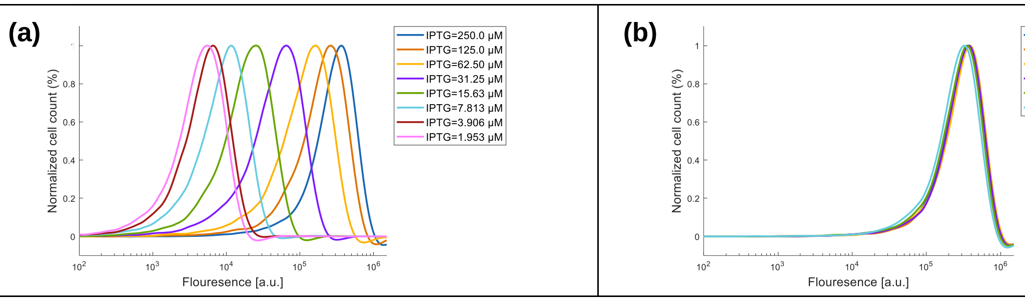

Supplementary Fig. 207. GFP flow cytometry data for a population of cells containing APF ($P_{luxTCTA}$) and ANF (P_{lacO1}) loops and combinatorial promoter ($P_{lux/lacO}$-GFP) to power-law and multiplication function (Supplementary Fig. 56a and c). **(a)** AHL was held constant at 3.0 μM and IPTG was varied. **(b)** IPTG was held constant at 250 μM and AHL was varied. Source data are available in the Source data file.

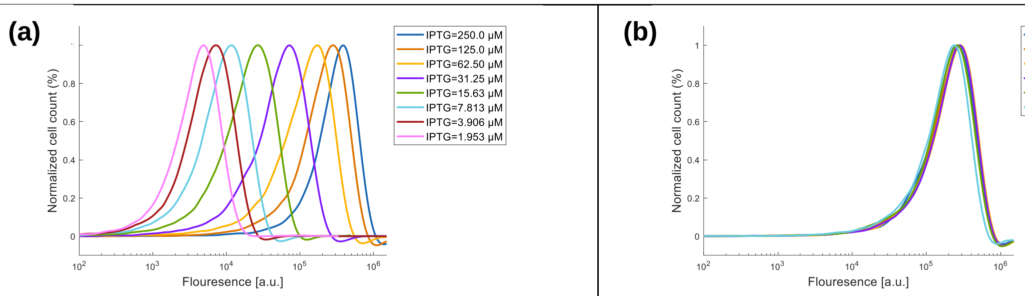

Supplementary Fig. 208. GFP flow cytometry data for a population of cells containing APF ($P_{luxTCTA}$) and ANF (P_{lacO1}) loops and combinatorial promoter ($P_{lux/lacO}$-GFP) to power-law and multiplication function (Supplementary Fig. 56a and c). **(a)** AHL was held constant at 1.5 μM and IPTG was varied. **(b)** IPTG was held constant at 125 μM and AHL was varied. Source data are available in the Source data file.

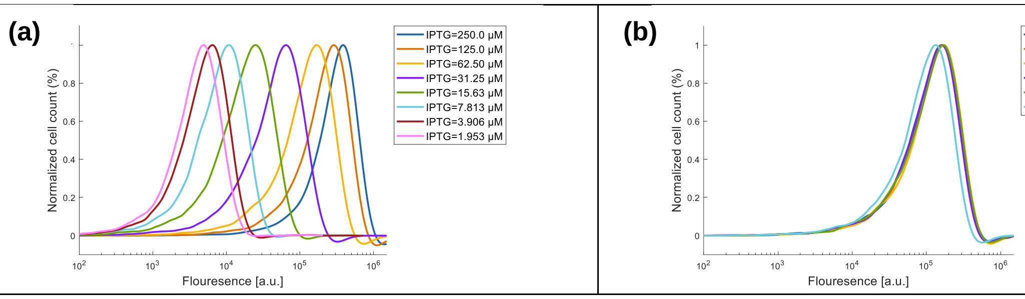

Supplementary Fig. 209 GFP flow cytometry data for a population of cells containing APF ($P_{luxTCTA}$) and ANF (P_{lacO1}) loops and combinatorial promoter ($P_{lux/lacO}$-GFP) to power-law and multiplication function (Supplementary Fig. 56a and c). **(a)** AHL was held constant at 0.75 μM and IPTG was varied. **(b)** IPTG was held constant at 62.5 μM and AHL was varied. Source data are available in the Source data file.

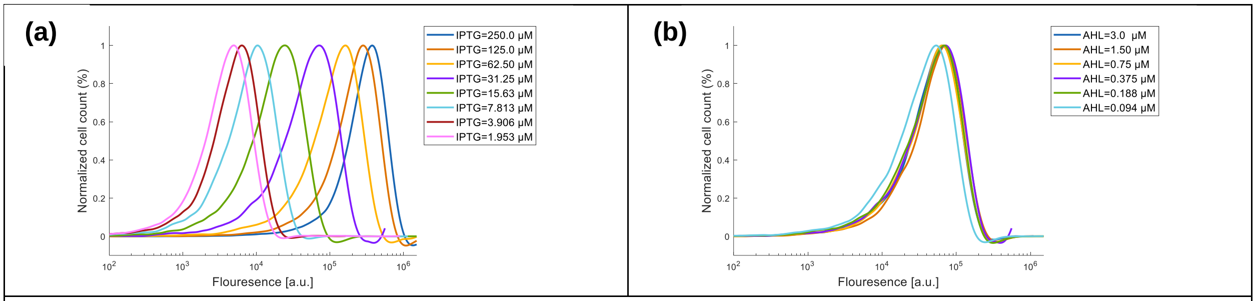

Supplementary Fig. 210. GFP flow cytometry data for a population of cells containing APF ($P_{luxTCTA}$) and ANF (P_{lacO1}) loops and combinatorial promoter ($P_{lux/lacO}$-GFP) to power-law and multiplication function (Supplementary Fig. 56a and c). **(a)** AHL was held constant at 0.375 μM and IPTG was varied. **(b)** IPTG was held constant at 31.25 μM and AHL was varied. Source data are available in the Source data file.

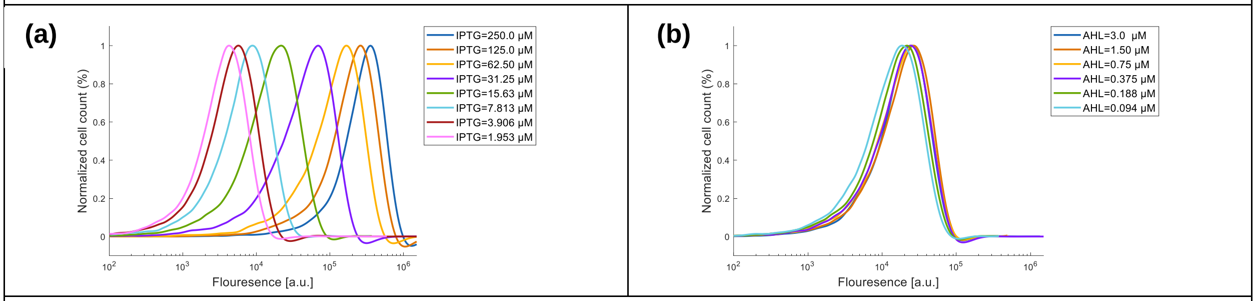

Supplementary Fig. 211. GFP flow cytometry data for a population of cells containing APF ($P_{luxTCTA}$) and ANF (P_{lacO1}) loops and combinatorial promoter ($P_{lux/lacO}$-GFP) to power-law and multiplication function (Supplementary Fig. 56a and c). **(a)** AHL was held constant at 0.188 μM and IPTG was varied. **(b)** IPTG was held constant at 15.63 μM and AHL was varied. Source data are available in the Source data file.

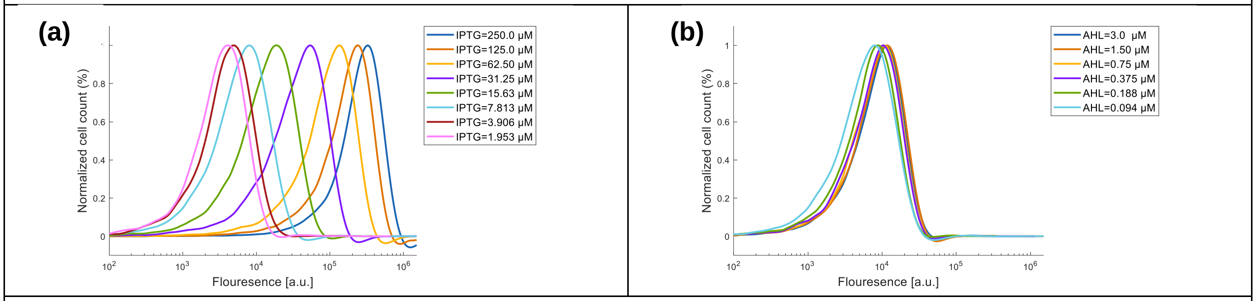

Supplementary Fig. 212. GFP flow cytometry data for a population of cells containing APF ($P_{luxTCTA}$) and ANF (P_{lacO1}) loops and combinatorial promoter ($P_{lux/lacO}$-GFP) to power-law and multiplication function (Supplementary Fig. 56a and c). **(a)** AHL was held constant at 0.094 μM and IPTG was varied. **(b)** IPTG was held constant at 7.813 μM and AHL was varied. Source data are available in the Source data file.

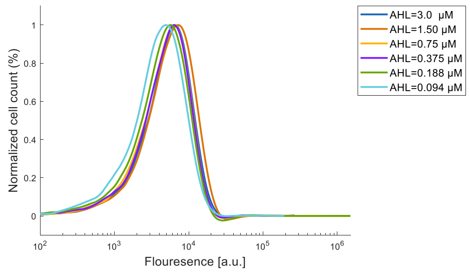

Supplementary Fig. 213. GFP flow cytometry data for a population of cells containing APF ($P_{luxTCTA}$) and ANF (P_{lacO1}) loops and combinatorial promoter ($P_{lux/lacO}$-GFP) to power-law and multiplication function (Supplementary Fig. 56a and c). IPTG was held constant at 3.906 μM and AHL was varied. Source data are available in the Source data file.

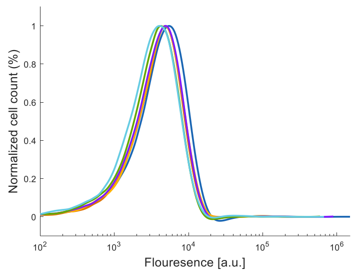

Supplementary Fig. 214. GFP flow cytometry data for a population of cells containing APF ($P_{luxTCTA}$) and ANF (P_{lacO1}) loops and combinatorial promoter ($P_{lux/lacO}$-GFP) to power-law and multiplication function (Supplementary Fig. 56a and c). IPTG was held constant at 1.953 μM and AHL was varied. Source data are available in the Source data file.

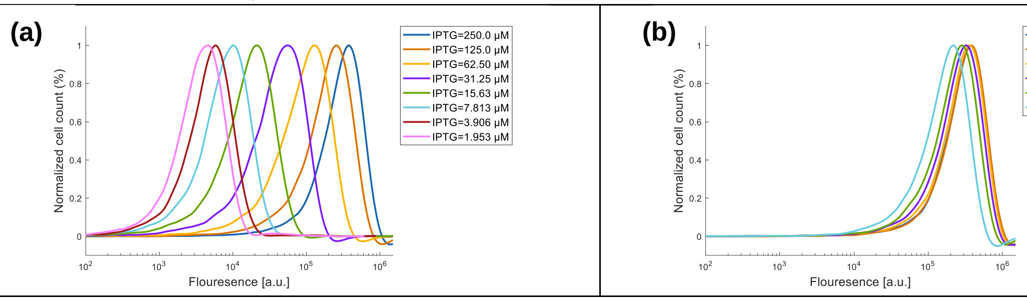

Supplementary Fig. 215. GFP flow cytometry data for a population of cells containing APF ($P_{luxGTTG}$) and ANF (P_{lacO1}) loops and combinatorial promoter ($P_{lux/lacO}$-GFP) to power-law and multiplication function (Supplementary Fig. 56a and d). **(a)** AHL was held constant at 3.0 μM and IPTG was varied. **(b)** IPTG was held constant at 250 μM and AHL was varied. Source data are available in the Source data file.

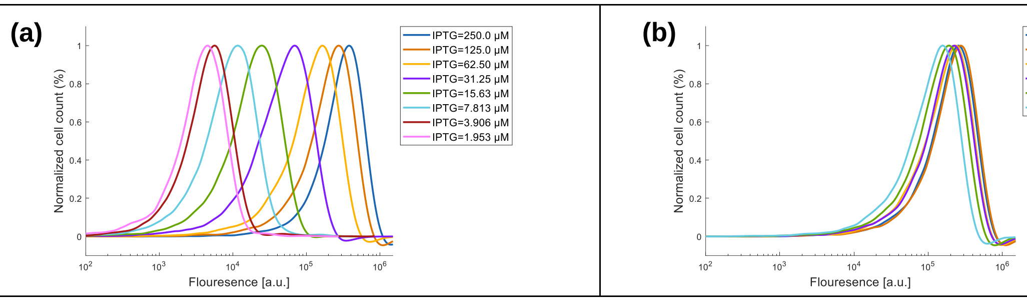

Supplementary Fig. 216. GFP flow cytometry data for a population of cells containing APF ($P_{luxGTTG}$) and ANF (P_{lacO1}) loops and combinatorial promoter ($P_{lux/lacO}$-GFP) to power-law and multiplication function (Supplementary Fig. 56a and d). **(a)** AHL was held constant at 1.5 μM and IPTG was varied. **(b)** IPTG was held constant at 125 μM and AHL was varied. Source data are available in the Source data file.

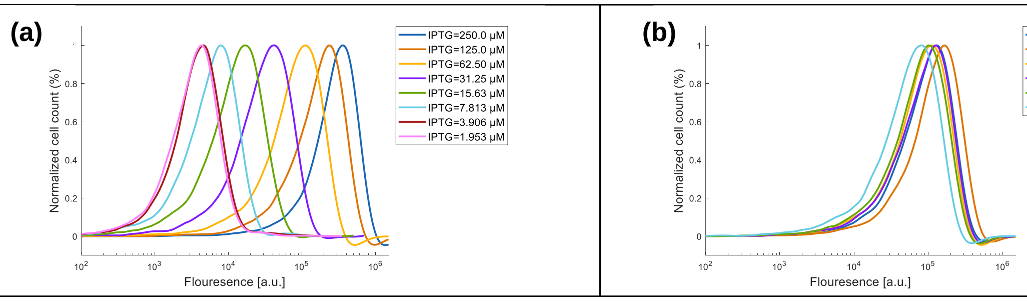

Supplementary Fig. 217. GFP flow cytometry data for a population of cells containing APF ($P_{luxGTTG}$) and ANF (P_{lacO1}) loops and combinatorial promoter ($P_{lux/lacO}$-GFP) to power-law and multiplication function (Supplementary Fig. 56a and d). **(a)** AHL was held constant at 0.75 μM and IPTG was varied. **(b)** IPTG was held constant at 62.5 μM and AHL was varied. Source data are available in the Source data file.

(a)

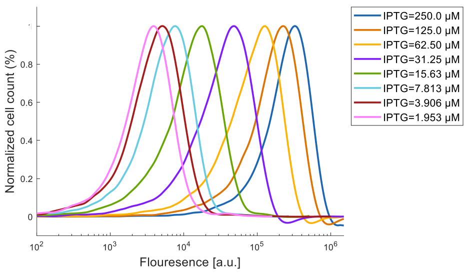

(b)

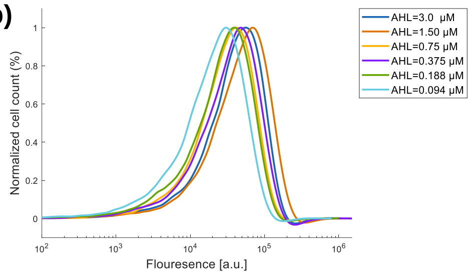

Supplementary Fig. 218. GFP flow cytometry data for a population of cells containing APF ($P_{luxGTTG}$) and ANF (P_{lacO1}) loops and combinatorial promoter ($P_{lux/lacO}$-GFP) to power-law and multiplication function (Supplementary Fig. 56a and d). **(a)** AHL was held constant at 0.375 μM and IPTG was varied. **(b)** IPTG was held constant at 31.25 μM and AHL was varied. Source data are available in the Source data file.

(a)

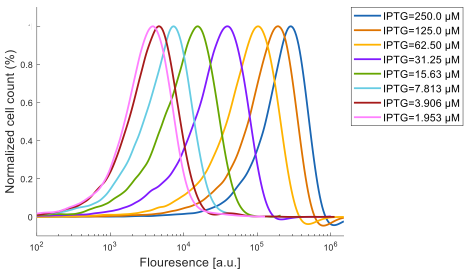

(b)

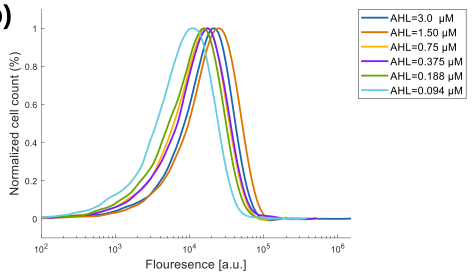

Supplementary Fig. 219. GFP flow cytometry data for a population of cells containing APF ($P_{luxGTTG}$) and ANF (P_{lacO1}) loops and combinatorial promoter ($P_{lux/lacO}$-GFP) to power-law and multiplication function (Supplementary Fig. 56a and d). **(a)** AHL was held constant at 0.188 μM and IPTG was varied. **(b)** IPTG was held constant at 15.63 μM and AHL was varied. Source data are available in the Source data file.

(a)

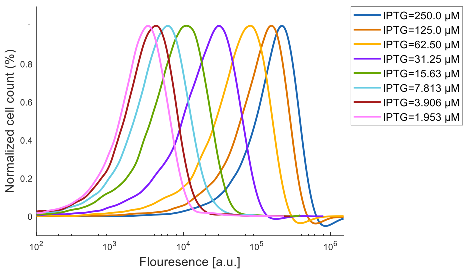

(b)

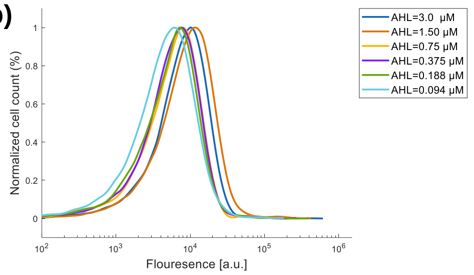

Supplementary Fig. 220 GFP flow cytometry data for a population of cells containing APF ($P_{luxGTTG}$) and ANF (P_{lacO1}) loops and combinatorial promoter ($P_{lux/lacO}$-GFP) to power-law and multiplication function (Supplementary Fig. 56a and d). **(a)** AHL was held constant at 0.094 μM and IPTG was varied. **(b)** IPTG was held constant at 7.813 μM and AHL was varied. Source data are available in the Source data file.

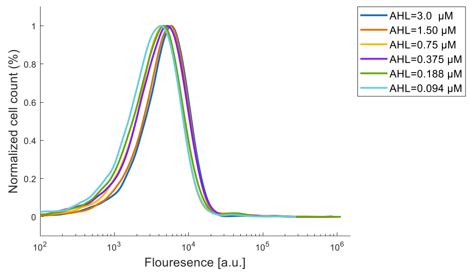

Supplementary Fig. 221. GFP flow cytometry data for a population of cells containing APF ($P_{luxGTTG}$) and ANF (P_{lacO1}) loops and combinatorial promoter ($P_{lux/lacO}$-GFP) to power-law and multiplication function (Supplementary Fig. 56a and d). IPTG was held constant at 3.906 μM and AHL was varied. Source data are available in the Source data file.

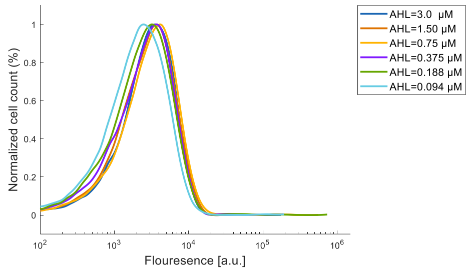

Supplementary Fig. 222. GFP flow cytometry data for a population of cells containing APF ($P_{luxGTTG}$) and ANF (P_{lacO1}) loops and combinatorial promoter ($P_{lux/lacO}$-GFP) to power-law and multiplication function (Supplementary Fig. 56a and d). IPTG was held constant at 1.953 μM and AHL was varied. Source data are available in the Source data file.

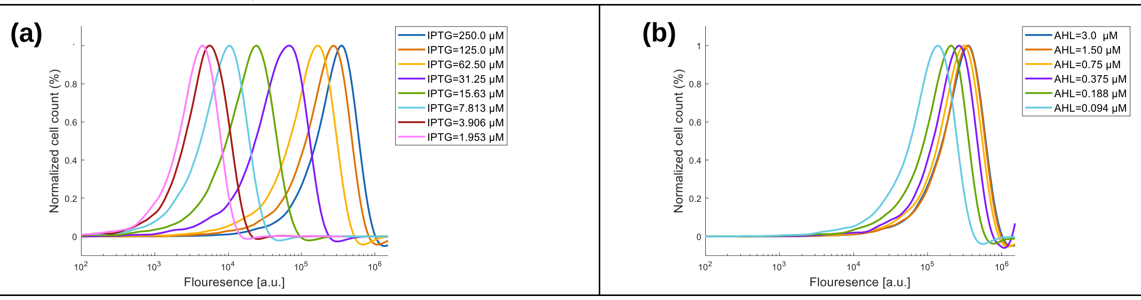

Supplementary Fig. 223. GFP flow cytometry data for a population of cells containing APF ($P_{luxGAGC}$) and ANF (P_{lacO1}) loops and combinatorial promoter ($P_{lux/lacO}$-GFP) to power-law and multiplication function (Supplementary Fig. 56a and e). **(a)** AHL was held constant at 3.0 μM and IPTG was varied. **(b)** IPTG was held constant at 250 μM and AHL was varied. Source data are available in the Source data file.

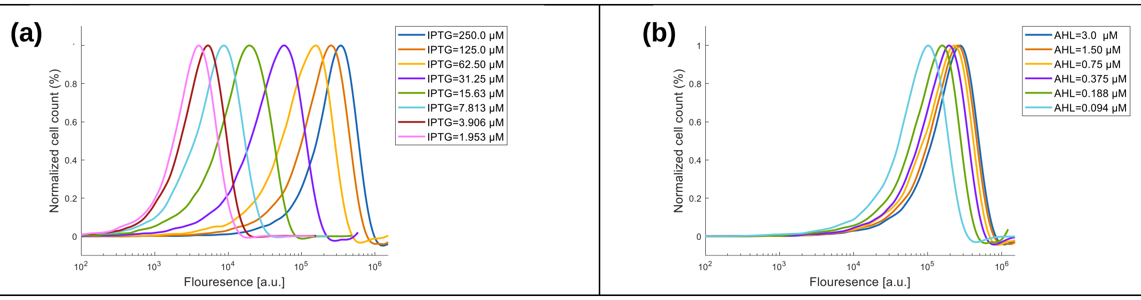

Supplementary Fig. 224. GFP flow cytometry data for a population of cells containing APF ($P_{luxGAGC}$) and ANF (P_{lacO1}) loops and combinatorial promoter ($P_{lux/lacO}$-GFP) to power-law and multiplication function (Supplementary Fig. 56a and e). **(a)** AHL was held constant at 1.5 μM and IPTG was varied. **(b)** IPTG was held constant at 125 μM and AHL was varied. Source data are available in the Source data file.

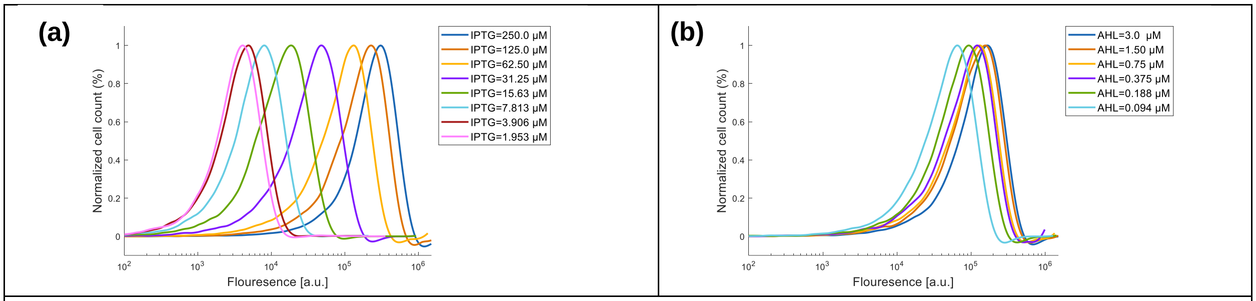

Supplementary Fig. 225. GFP flow cytometry data for a population of cells containing APF ($P_{luxGAGC}$) and ANF (P_{lacO1}) loops and combinatorial promoter ($P_{lux/lacO}$-GFP) to power-law and multiplication function (Supplementary Fig. 56a and e). **(a)** AHL was held constant at 0.75 µM and IPTG was varied. **(b)** IPTG was held constant at 62.5 µM and AHL was varied. Source data are available in the Source data file.

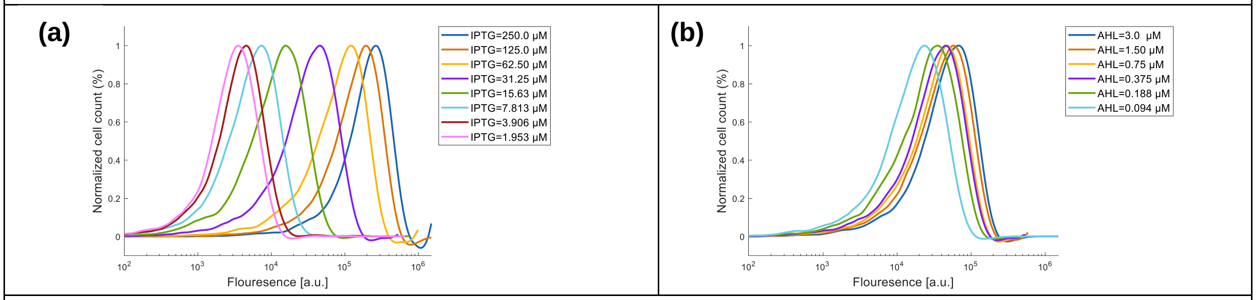

Supplementary Fig. 226. GFP flow cytometry data for a population of cells containing APF ($P_{luxGAGC}$) and ANF (P_{lacO1}) loops and combinatorial promoter ($P_{lux/lacO}$-GFP) to power-law and multiplication function (Supplementary Fig. 56a and e). **(a)** AHL was held constant at 0.375 µM and IPTG was varied. **(b)** IPTG was held constant at 31.25 µM and AHL was varied. Source data are available in the Source data file.

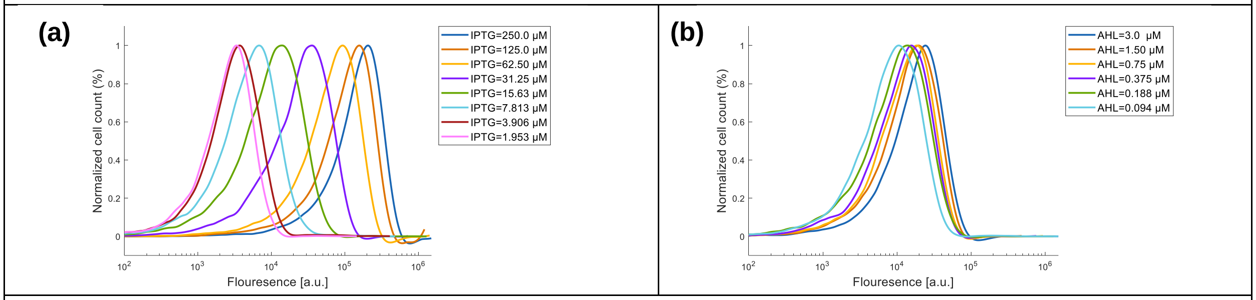

Supplementary Fig. 227. GFP flow cytometry data for a population of cells containing APF ($P_{luxGAGC}$) and ANF (P_{lacO1}) loops and combinatorial promoter ($P_{lux/lacO}$-GFP) to power-law and multiplication function (Supplementary Fig. 56a and e). **(a)** AHL was held constant at 0.188 µM and IPTG was varied. **(b)** IPTG was held constant at 15.63 µM and AHL was varied. Source data are available in the Source data file.

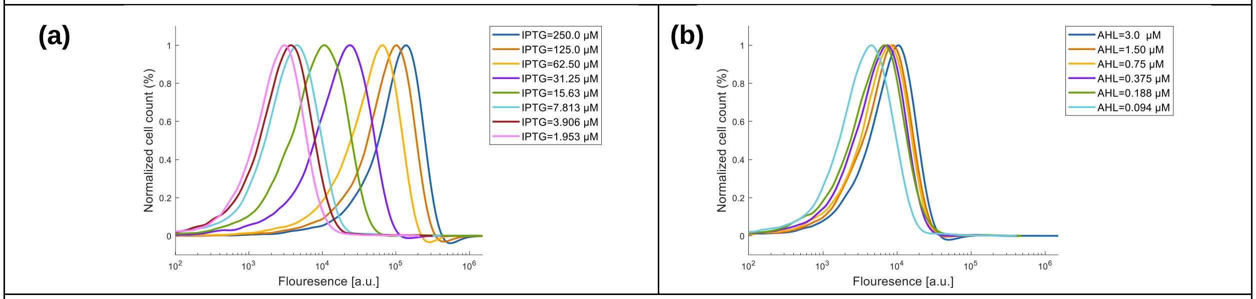

Supplementary Fig. 228. GFP flow cytometry data for a population of cells containing APF ($P_{luxGAGC}$) and ANF (P_{lacO1}) loops and combinatorial promoter ($P_{lux/lacO}$-GFP) to power-law and multiplication function (Supplementary Fig. 56a and e). **(a)** AHL was held constant at 0.094 µM and IPTG was varied. **(b)** IPTG was held constant at 7.813 µM and AHL was varied. Source data are available in the Source data file.

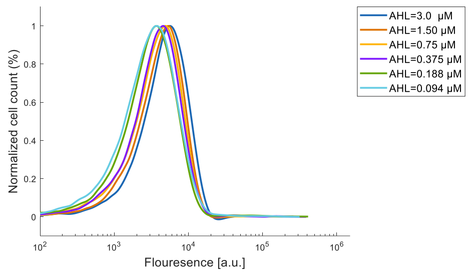

Supplementary Fig. 229. GFP flow cytometry data for a population of cells containing APF ($P_{luxGAGC}$) and ANF (P_{lacO1}) loops and combinatorial promoter ($P_{lux/lacO}$-GFP) to power-law and multiplication function (Supplementary Fig. 56a and e). IPTG was held constant at 3.906 μM and AHL was varied. Source data are available in the Source data file.

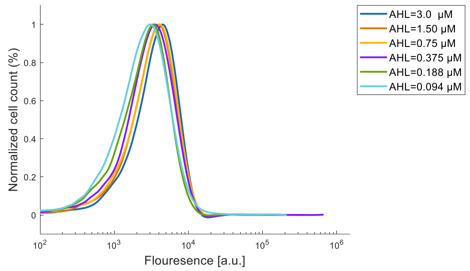

Supplementary Fig. 230. GFP flow cytometry data for a population of cells containing APF ($P_{luxGAGC}$) and ANF (P_{lacO1}) loops and combinatorial promoter ($P_{lux/lacO}$-GFP) to power-law and multiplication function (Supplementary Fig. 56a and e). IPTG was held constant at 1.953 μM and AHL was varied. Source data are available in the Source data file.

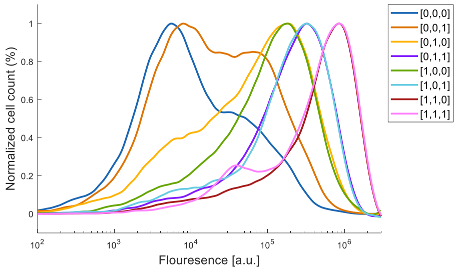

Supplementary Fig. 231. GFP flow cytometry data for a population of cells containing the synthetic multilayer perceptgene displays a new logic function for three input (AHL, IPTG and aTc) (Supplementary Fig. 78). Measured response of majority circuit. AHL [0.1875, 0.3μM], IPTG [7.8125, 125μM], aTc [1.5625, 25ng/mL] and Arabinose [0.03125 mM]. Source data are available in the Source data file.

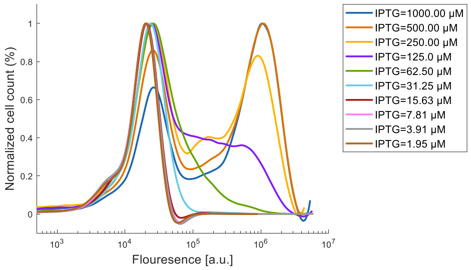

Supplementary Fig. 232. GFP flow cytometry data for a population of cells containing ExsA under combinatorial promoter ($P_{lacO/tetO}$-GFP) and ExsD P_{luxTGT} (Fig. 5c). AHL was held constant at 1.33 μM and IPTG was varied. Source data are available in the Source data file.

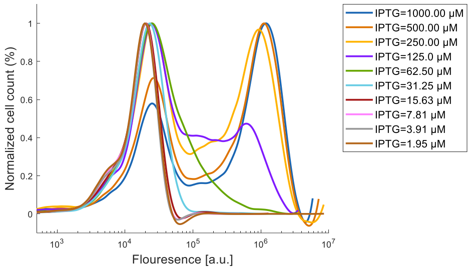

Supplementary Fig. 233. GFP flow cytometry data for a population of cells containing ExsA under combinatorial promoter ($P_{lacO/tetO}$-GFP) and ExsD P_{luxTGT} (Fig. 5c). AHL was held constant at 0.88 μM and IPTG was varied. Source data are available in the Source data file.

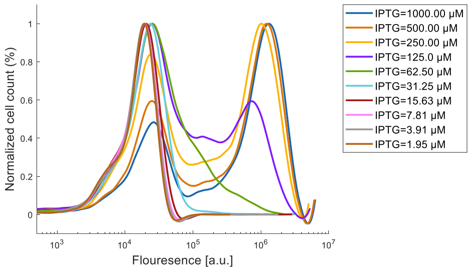

Supplementary Fig. 234. GFP flow cytometry data for a population of cells containing ExsA under combinatorial promoter ($P_{lacO/tetO}$-GFP) and ExsD P_{luxTGT} (Fig. 5c). AHL was held constant at 0.59 μM and IPTG was varied. Source data are available in the Source data file.

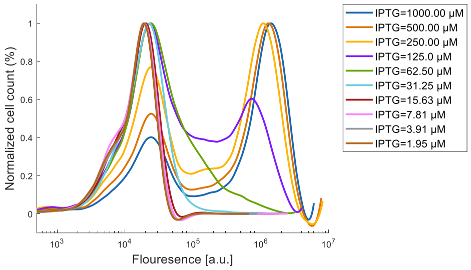

Supplementary Fig. 235. GFP flow cytometry data for a population of cells containing ExsA under combinatorial promoter ($P_{lacO/tetO}$-GFP) and ExsD P_{luxTGT} (Fig. 5c). AHL was held constant at 0.39 μM and IPTG was varied. Source data are available in the Source data file.

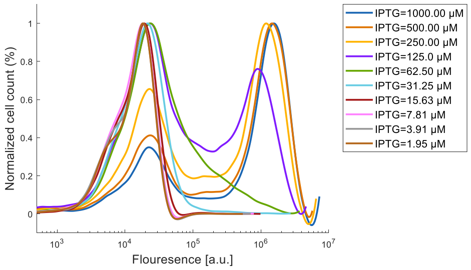

Supplementary Fig. 236. GFP flow cytometry data for a population of cells containing ExsA under combinatorial promoter ($P_{lacO/tetO}$-GFP) and ExsD P_{luxTGT} (Fig. 5c). AHL was held constant at 0.26 µM and IPTG was varied. Source data are available in the Source data file.

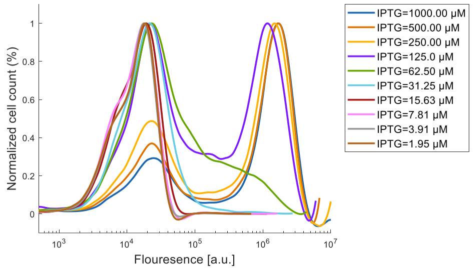

Supplementary Fig. 237. GFP flow cytometry data for a population of cells containing ExsA under combinatorial promoter ($P_{lacO/tetO}$-GFP) and ExsD P_{luxTGT} (Fig. 5c). AHL was held constant at 0.17 µM and IPTG was varied. Source data are available in the Source data file.

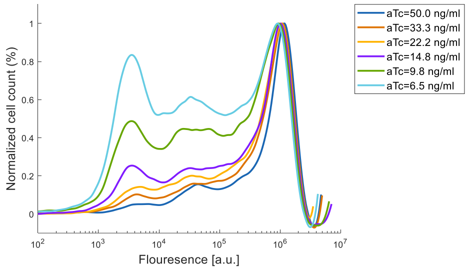

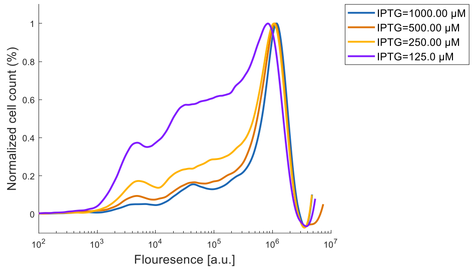

Supplementary Fig. 238. GFP flow cytometry data for a population of cells of two-input programmable perceptgene genetic circuit when ANF loops (P_{lacO1} and P_{tetO}) are located on MCP with AHL=0 (Fig. 5e). **(a)** IPTG was held constant at 1000 µM and aTc was varied. **(b)** aTc was held constant at 50 ng/ml and IPTG was varied. Source data are available in the Source data file.

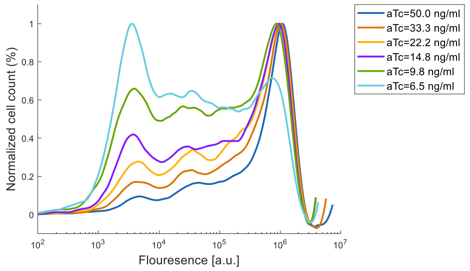

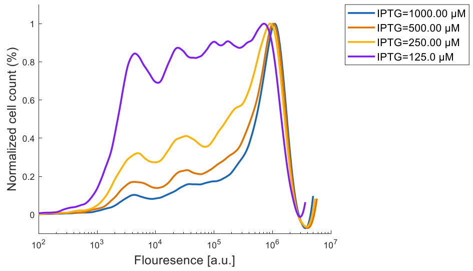

Supplementary Fig. 239. GFP flow cytometry data for a population of cells of two-input programmable perceptgene genetic circuit when ANF loops (P_{lacO1} and P_{tetO}) are located on MCP with AHL=0 (Fig. 5e). **(a)** IPTG was held constant at 500 µM and aTc was varied. **(b)** aTc was held constant at 33.3 ng/ml and IPTG was varied (Fig. 5e). Source data are available in the Source data file.

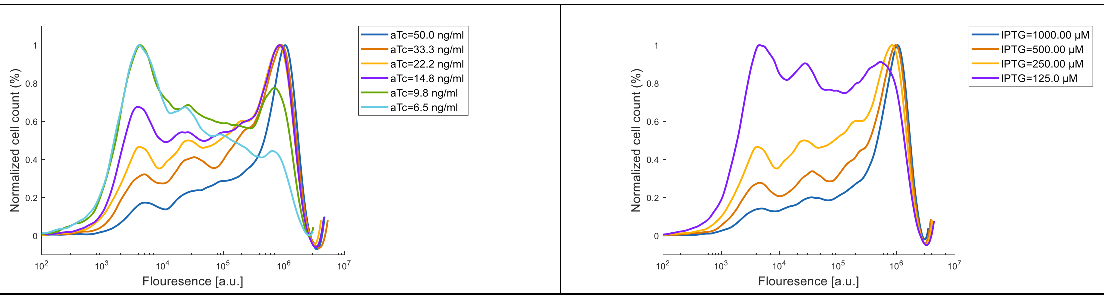

Supplementary Fig. 240. GFP flow cytometry data for a population of cells of two-input programmable perceptgene genetic circuit when ANF loops (P_{lacO1} and P_{tetO}) are located on MCP with AHL=0 (Fig. 5e). **(a)** IPTG was held constant at 250 μM and aTc was varied. **(b)** aTc was held constant at 22.2 ng/ml and IPTG was varied. Source data are available in the Source data file.

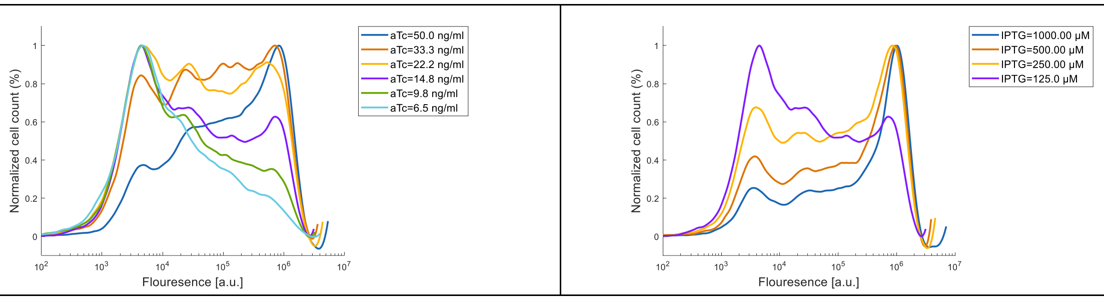

Supplementary Fig. 241. GFP flow cytometry data for a population of cells of two-input programmable perceptgene genetic circuit when ANF loops (P_{lacO1} and P_{tetO}) are located on MCP with AHL=0 (Fig. 5e). **(a)** IPTG was held constant at 125 μM and aTc was varied. **(b)** aTc was held constant at 14.8 ng/ml and IPTG was varied. Source data are available in the Source data file.

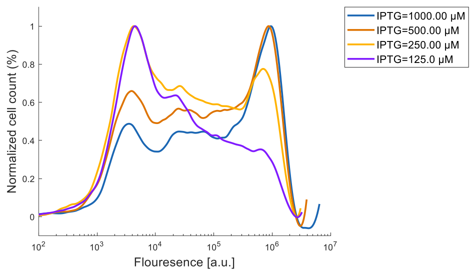

Supplementary Fig. 242. GFP flow cytometry data for a population of cells of two-input programmable perceptgene genetic circuit when ANF loops (P_{lacO1} and P_{tetO}) are located on MCP with AHL=0 (Fig. 5e). aTc was held constant at 9.8 ng/ml and IPTG was varied. Source data are available in the Source data file.

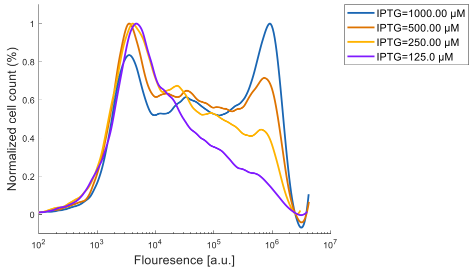

Supplementary Fig. 243. GFP flow cytometry data for a population of cells of two-input programmable perceptgene genetic circuit when ANF loops (P_{lacO1} and P_{tetO}) are located on MCP with AHL=0 (Fig. 5e). aTc was held constant at 5.5 ng/ml and IPTG was varied. Source data are available in the Source data file.

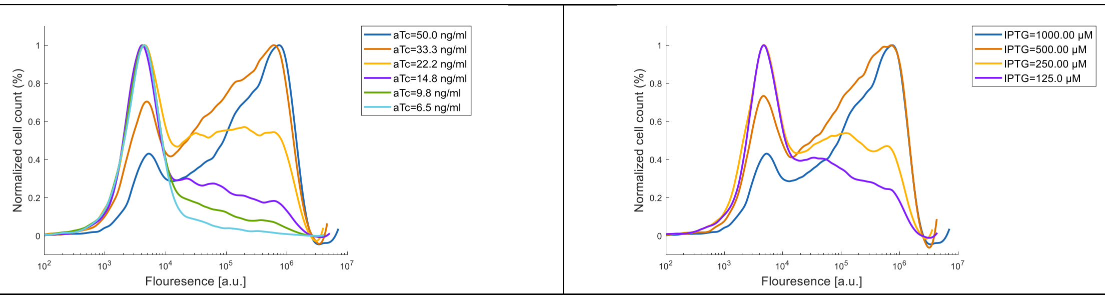

Supplementary Fig. 244. GFP flow cytometry data for a population of cells of two-input programmable perceptgene genetic circuit when ANF loops (P_{lacO1} and P_{tetO}) are located on MCP with AHL=0.34 μM (Fig. 5f). **(a)** IPTG was held constant at 1000 μM and aTc was varied. **(b)** aTc was held constant at 50 ng/ml and IPTG was varied. Source data are available in the Source data file.

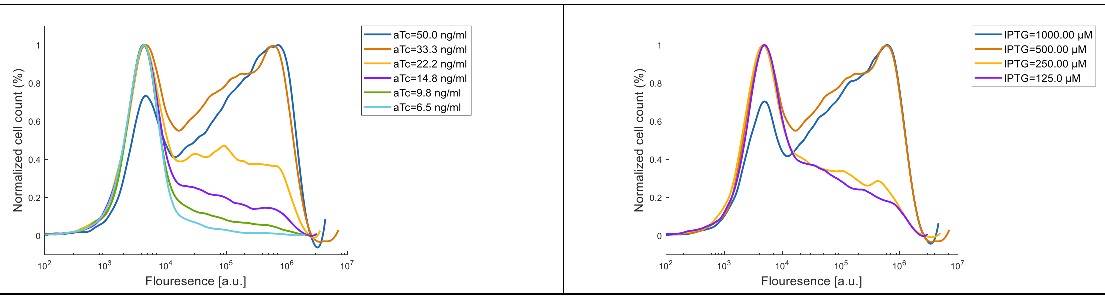

Supplementary Fig. 245. GFP flow cytometry data for a population of cells of two-input programmable perceptgene genetic circuit when ANF loops (P_{lacO1} and P_{tetO}) are located on MCP with AHL=0.34 μM (Fig. 5f). **(a)** IPTG was held constant at 500 μM and aTc was varied. **(b)** aTc was held constant at 33.3 ng/ml and IPTG was varied. Source data are available in the Source data file.

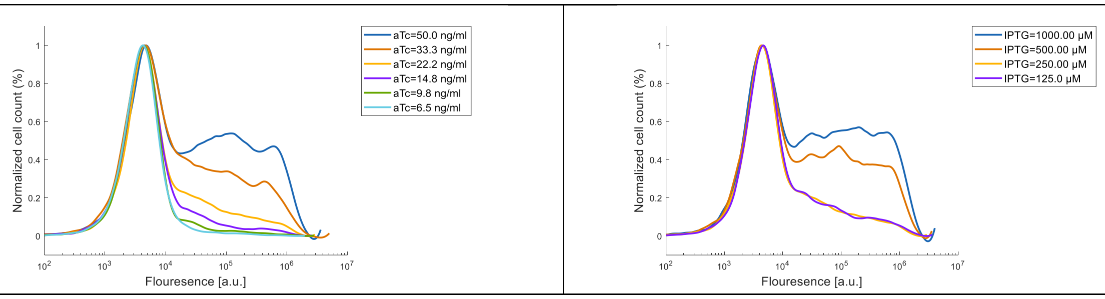

Supplementary Fig. 246. GFP flow cytometry data for a population of cells of two-input programmable perceptgene genetic circuit when ANF loops (P_{lacO1} and P_{tetO}) are located on MCP with AHL=0.34 μM (Fig. 5f). **(a)** IPTG was held constant at 250 μM and aTc was varied. **(b)** aTc was held constant at 22.2 ng/ml and IPTG was varied. Source data are available in the Source data file.

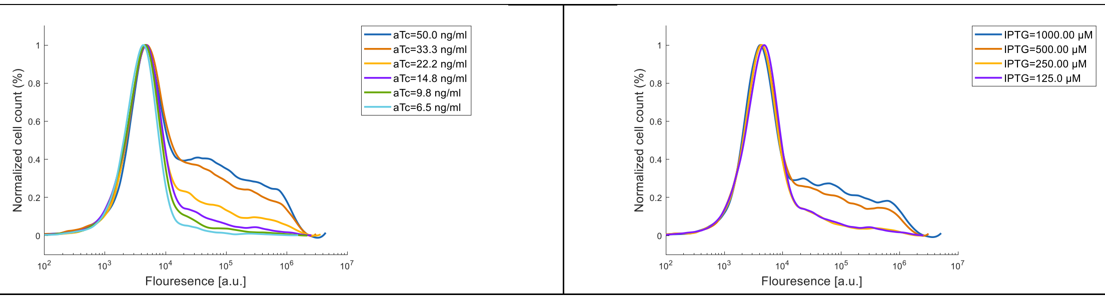

Supplementary Fig. 247. GFP flow cytometry data for a population of cells of two-input programmable perceptgene genetic circuit when ANF loops (P_{lacO1} and P_{tetO}) are located on MCP with AHL=0.34 μM (Fig. 5f). **(a)** IPTG was held constant at

125 µM and aTc was varied. **(b)** aTc was held constant at 14.8 ng/ml and IPTG was varied. Source data are available in the Source data file.

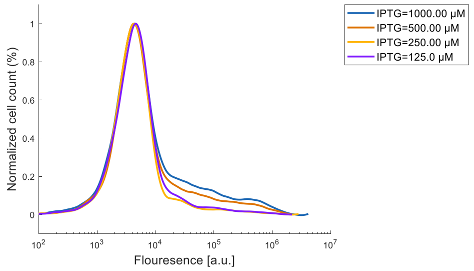

Supplementary Fig. 248. GFP flow cytometry data for a population of cells of two-input programmable perceptgene genetic circuit when ANF loops (P_{lacO1} and P_{tetO}) are located on MCP with AHL=0.34 µM (Fig. 5f). aTc was held constant at 9.8 ng/ml and IPTG was varied. Source data are available in the Source data file.

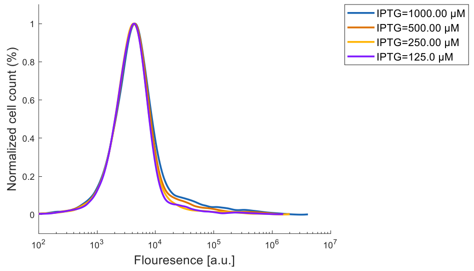

Supplementary Fig. 249. GFP flow cytometry data for a population of cells of two-input programmable perceptgene genetic circuit when ANF loops (P_{lacO1} and P_{tetO}) are located on MCP with AHL=0.34 µM (Fig. 5f). aTc was held constant at 5.5 ng/ml and IPTG was varied. Source data are available in the Source data file.

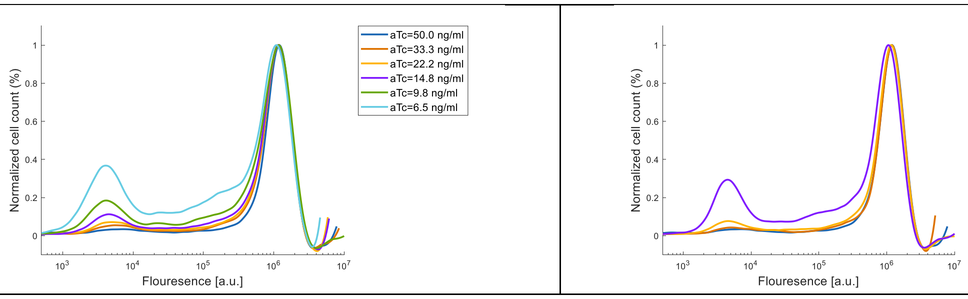

Supplementary Fig. 250. GFP flow cytometry data for a population of cells of two-input programmable perceptgene genetic circuit when ANF loops (P_{lacO1} and P_{tetO}) are located on LCP with AHL=0 (Fig. 5g). **(a)** IPTG was held constant at 1000 µM and aTc was varied. **(b)** aTc was held constant at 50 ng/ml and IPTG was varied. Source data are available in the Source data file.

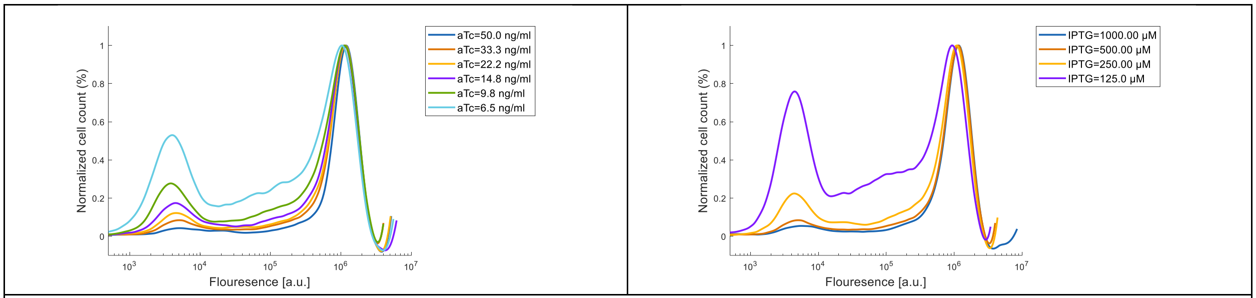

Supplementary Fig. 251. GFP flow cytometry data for a population of cells of two-input programmable perceptgene genetic circuit when ANF loops (P_{lacO1} and P_{tetO}) are located on LCP with AHL=0 (Fig. 5g). **(a)** IPTG was held constant at 500 μM and aTc was varied. **(b)** aTc was held constant at 33.3 ng/ml and IPTG was varied. Source data are available in the Source data file.

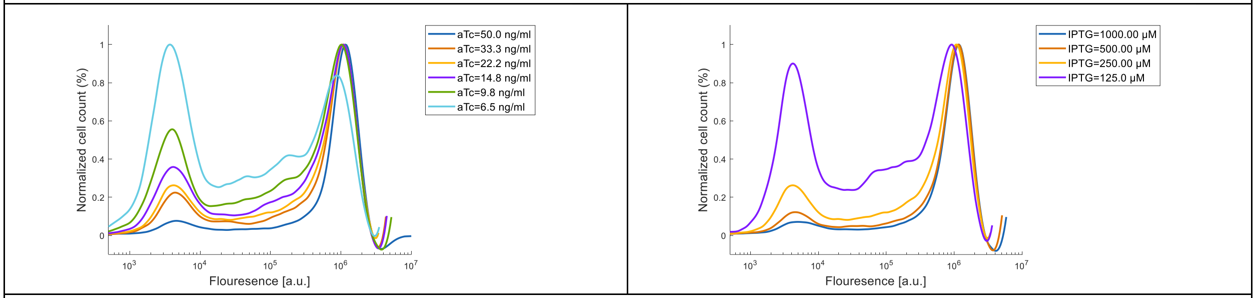

Supplementary Fig. 252. GFP flow cytometry data for a population of cells of two-input programmable perceptgene genetic circuit when ANF loops (P_{lacO1} and P_{tetO}) are located on LCP with AHL=0 (Fig. 5g). **(a)** IPTG was held constant at 250 μM and aTc was varied. **(b)** aTc was held constant at 22.2 ng/ml and IPTG was varied. Source data are available in the Source data file.

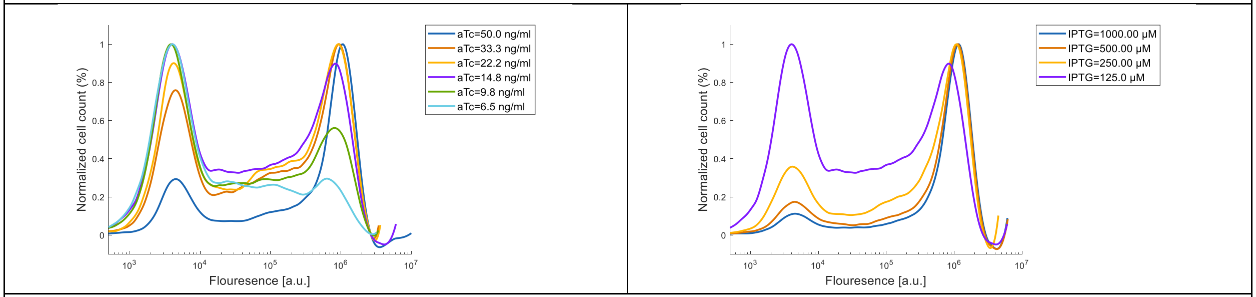

Supplementary Fig. 253. GFP flow cytometry data for a population of cells of two-input programmable perceptgene genetic circuit when ANF loops (P_{lacO1} and P_{tetO}) are located on LCP with AHL=0 (Fig. 5g). **(a)** IPTG was held constant at 125 μM and aTc was varied. **(b)** aTc was held constant at 14.8 ng/ml and IPTG was varied. Source data are available in the Source data file.

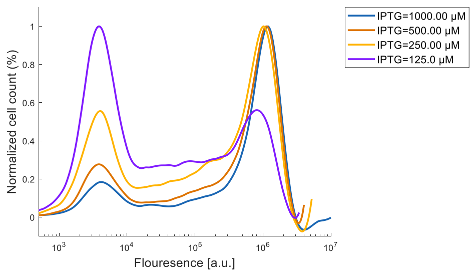

Supplementary Fig. 254. GFP flow cytometry data for a population of cells of two-input programmable perceptgene genetic circuit when ANF loops (P_{lacO1} and P_{tetO}) are located on LCP with AHL=0 (Fig. 5g). aTc was held constant at 9.8 ng/ml and IPTG was varied. Source data are available in the Source data file.

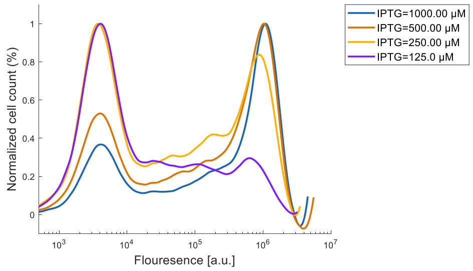

Supplementary Fig. 255. GFP flow cytometry data for a population of cells of two-input programmable perceptgene genetic circuit when ANF loops (P_{lacO1} and P_{tetO}) are located on LCP with AHL=0 (Fig. 5g). aTc was held constant at 6.5 ng/ml and IPTG was varied. Source data are available in the Source data file.

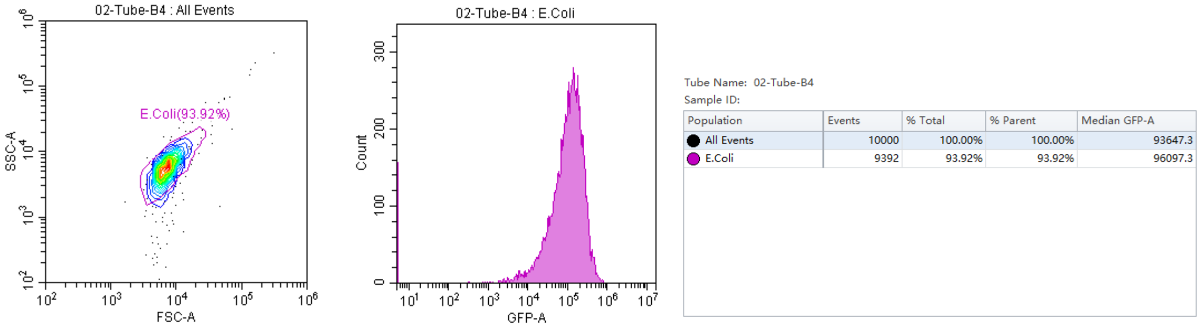

Supplementary Fig. 256. Shows a typical behavior of fluorescence. Forward and side scattering were used by CytExpert 2.2 software.

14. List of biological parts used in this study

Part	Description and source	DNA sequence	Mutation done in this study
araC	AraC coding sequence[12]	atggctgaagcgcaaaatgatcccctgctgccgggatactcgtttaacgcccatctg<br>gtggcgggtttaacgccgattgaggccaacggttatctcgatttttttatcgaccgacc<br>gctgggaatgaaaggttatattctcaatctcaccattcgcggtcaggggggtggtgaa<br>aaatcagggacgagaatttgtctgccgaccgggtgatattttgctgttcccgccagg<br>agagattcatcactacggtcgtcatccggaggctcgcgaatggtatcaccagtggg<br>tttactttcgtccgcgcgcctactggcatgaatggcttaactggccgtcaatatttgcca<br>atacgggtttctttcgcccggatgaagcgcaccagccgcatttcagcgacctgtttgg<br>gcaaatcattaacgccgggcaaggggaagggcgctattcggagctgctggcgat<br>aaatctgcttgagcaattgttactgcggcgcatggaagcgattaacgagtcgctcca<br>tccaccgatggataatcgggtacgcgaggcttgtcagtacatcagcgatcacctgg<br>cagacagcaattttgatatcgccagcgtcgcacagcatgtttgcttgtcgccgtcgcg<br>tctgtcacatcttttccgccagcagttagggattagcgtcttaagctggcgcgaggac<br>caacgcattagtcaggcgaagctgcttttgagcactacccggatgcctatcgccac<br>cgtcggtcgcaatgttggttttgacgatcaactctatttctcgcgagtatttaaaaaatg<br>caccggggccagcccgagcgagtttcgtgccggttgtgaagaaaaagtgaatgat<br>gtagccgtcaagttgtcataa	
araC (Truncated)		atggctgaagcgcaaaatgatcccctgctgccgggatactcgtttaacgcccatctg<br>gtggcgggtttaacgccgattgaggccaacggttatctcgatttttttatcgaccgacc<br>gctgggaatgaaaggttatattctcaatctcaccattcgcggtcaggggggtggtgaa<br>aaatcagggacgagaatttgtctgccgaccgggtgatattttgctgttcccgccagg<br>agagattcatcactacggtcgtcatccggaggctcgcgaatggtatcaccagtggg<br>tttactttcgtccgcgcgcctactggcatgaatggcttaactggccgtcaatatttgcca<br>atacgggtttctttcgcccggatgaagcgcaccagccgcatttcagcgacctgtttgg<br>gcaaatcattaacgccgggcaaggggaagggcgctattcggagctgctggcgat<br>aaatctgcttgagcaattgttactgcggcgcatggaagcgattaacgagtcgctcca<br>tccaccgatggataatcgggtacgcgaggcttgtcagtacatcagcgatcacctgg<br>cagacagcaattttgatatcgccagcgtcgcacagcatgtttgcttgtcgccgtcgcg<br>tctgtcacatcttttccgccagcagttagggattagcgtcttaagctggcgcgaggac<br>caacgcattagtcaggcgaagctgcttttgagcactacccggatgcctatcgccac<br>cgtcggtcgcaatgttggttttgacgatcaactctatttctcgcgagtatttaaaaaatg<br>caccggggccagcccgagcgagtttcgtgccggttaa	
GFP	Enhanced Green Fluorescent Protein coding sequence [73]	atg**a**gtaaaggagaagaacttttcactggagttgtcccaattcttgttgaattagatgg<br>tgatgttaatgggcacaaattttctgtcagtggagagggtgaaggtgatgcaacata<br>cggaaaacttacccttaaatttatttgcactactggaaaactacctgttccatggcca<br>acacttgtcactactttcggttatggtgttcaatgctttgcgagatacccagatcatatg<br>aaacagcatgactttttcaagagtgccatgcccgaaggttatgtacaggaaagaa<br>ctatatttttcaaagatgacgggaactacaagacacgtgctgaagtcaagtttgaag<br>gtgatacccttgttaatagaatcgagttaaaaggtattgattttaaagaagatggaaa<br>cattcttggacacaaattggaatacaactataactcacacaatgtatacatcatggc<br>agacaaacaaaagaatggaatcaaagttaacttcaaaattagacacaacattga<br>agatggaagcgttcaactagcagaccattatcaacaaaatactccaattggcgatg<br>gccctgtccttttaccagacaaccattacctgtccacacaatctgccctttcgaaaga<br>tcccaacgaaaagagagaccacatggtccttcttgagtttgtaacagctgctgggat<br>tacacatggcatggatgaactatacaaataa	Either a or c
LacI	LacI coding sequence [74]	gtgaaaccagtaacgttatacgatgtcgcagagtatgccggtgtctcttatcagacc<br>gtttcccgcgtggtgaaccaggccagccacgtttctgcgaaaacgcgggaaaaa<br>gtggaagcggcgatggcggagctgaattacattcccaaccgcgtggcacaacaa<br>ctggcgggcaaacagtcgttgctgattggcgttgccacctccagtctggccctgcac<br>gcgccgtcgcaaattgtcgcggcgattaaatctcgcgccgatcaactgggtgccag<br>cgtggtggtgtcgatggtagaacgaagcggcgtcgaagcctgtaaagcggcggt<br>gcacaatcttctcgcgcaacgcgtcagtgggctgatcattaactatccgctggatga<br>ccaggatgccattgctgtggaagctgcctgcactaatgttccggcgttatttcttgatgt<br>ctctgaccagacacccatcaacagtattattttctcccatgaagacggtacgcgact<br>gggcgtggagcatctggtcgcattgggtcaccagcaaatcgcgctgttagcgggc<br>ccattaagttctgtctcggcgcgtctgcgtctggctggctggcataaatatctcactcg	

		caatcaaattcagccgatagcggaacgggaaggcgactggagtgccatgtccgg<br>ttttcaacaaaccatgcaaatgctgaatgagggcatcgttcccactgcgatgctggtt<br>gccaacgatcagatggcgctgggcgcaatgcgcgccattaccgagtccgggctg<br>cgcgttggtgcggatatctcggtagtgggatacgacgataccgaagacagctcatg<br>ttatatcccgccgttaaccaccatcaaacaggattttcgcctgctggggcaaaccag<br>cgtggaccgcttgctgcaactctctcagggccaggcggtgaagggcaatcagctg<br>ttgcccgtctcactggtgaaaagaaaaaccaccctggcgcccaatacgcaaacc<br>gcctctccccgcgcgttggccgattcattaatgcagctggcacgacaggtttcccga<br>ctggaaagcgggcagtga	
LuxR	LuxR coding sequence (BBa_C0062) [75], induced by AHL (3OC6HSL)	atgaaaaacataaatgccgacgacacatacagaataattaataaaattaaagctt<br>gtagaagcaataatgatattaatcaatgcttatctgatatgactaaaatggtacattgt<br>gaatattatttactcgcgatcatttatcctcattctatggttaaatctgatatttcaatccta<br>gataattaccctaaaaaatggaggcaatattatgatgacgctaatttaataaaatat<br>gatcctatagtagattattctaactccaatcattcaccaattaattggaatatatttgaa<br>aacaatgctgtaaataaaaaatctccaaatgtaattaaagaagcgaaaacatcag<br>gtcttatcactgggtttagtttccctattcatacggctaacaatggcttcggaatgcttag<br>ttttgcacattcagaaaaagacaactatatagatagtttatttttacatgcgtgtatgaa<br>cataccattaattgttccttctctagttgataattatcgaaaaataaatatagcaaataa<br>taaatcaaacaacgatttaaccaaaagagaaaaagaatgtttagcgtgggcatgc<br>gaaggaaaaagctcttgggatatttcaaaaatattaggttgcagtgagcgtactgtc<br>actttccatttaaccaatgcgcaaatgaaactcaatacaacaaaccgctgccaaa<br>gtatttctaaagcaattttaacaggagcaattgattgcccatactttaaaaattaataa	
mCherry	Red Fluorescent Protein coding sequence [73]	atggtgagcaagggcgaagaagataacatggccatcatcaaggagttcatgcgc<br>ttcaaggtgcacatggagggctccgtgaacggccacgagttcgagatcgagggc<br>gagggcgagggccgcccctacgagggcacccagaccgccaagctgaaggtga<br>ccaagggtggccccctgcccttcgcctgggacatcctgtcccctcagttcatgtacg<br>gctccaaggcctacgtgaagcaccccgccgacatccccgactacttgaagctgtc<br>cttccccgagggcttcaagtgggagcgcgtgatgaacttcgaggacggcggcgtg<br>gtgaccgtgacccaggactcctccctgcaggacggcgagttcatctacaaggtga<br>agctgcgcggcaccaacttcccctccgacggccccgtaatgcagaagaagacc<br>atgggctgggaggcctcctccgagcggatgtaccccgaggacggcgccctgaag<br>ggcgagatcaagcagaggctgaagctgaaggacggcggccactacgacgctg<br>aggtcaagaccacctacaaggccaagaagcccgtgcagctgcccggcgcctac<br>aacgtcaacatcaagttggacatcacctcccacaacgaggactacaccatcgtgg<br>aacagtacgaacgcgccgagggccgccactccaccggcggcatggacgagct<br>gtacaagtaa	
tetR	tetR coding sequence [74]	atgtccagattagataaaagtaaagtgattaacagcgcattagagctgcttaatgag<br>gtcggaatcgaaggtttaacaacccgtaaactcgcccagaagctaggtgtagagc<br>agcctacattgtattggcatgtaaaaaataagcgggctttgctcgacgccttagccat<br>tgagatgttagataggcaccatactcacttttgccctttagaaggggaaagctggca<br>agatttttacgtaataacgctaaaagttttagatgtgctttactaagtcatcgcgatgg<br>agcaaaagtacatttaggtacacggcctacagaaaaacagtatgaaactctcga<br>aaatcaattagcctttttatgccaacaaggtttttcactagagaatgcattatatgcact<br>cagcgctgtggggcattttactttaggttgcgtattggaagatcaagagcatcaagtc<br>gctaaagaagaaagggaaacacctactactgatagtatgccgccattattacgac<br>aagctatcgaattatttgatcaccaaggtgcagagccagccttcttattcggccttga<br>attgatcatatgcggattagaaaaacaacttaaatgtgaaagtgggtcctaa	
T7ptag	T7 Tag coding sequence [20]	atgattaccgtgcactagaataccattaacattgctaagaacgacttctctgacatcg<br>aactggctgctatcccgttcaacactctggctgaccattacggtgagcgtttagctcg<br>cgaacagttggcccttgagcatgagtcttacgagatgggtgaagcacgcttccgca<br>agatgtttgagcgtcaacttaaagctggtgaggttgcggataacgctgccgccaag<br>cctctcatcactaccctactccctaagatgattgcacgcatcaacgactggtttgagg<br>aagtgaaagctaagcgcggcaagcgcccgacagccttccagttcctgtaggaaa<br>tcaagccggaagccgtagcgtacatcaccattaagaccactctggcttgcctaacc<br>agtgctgacaatacaaccgttcaggctgtagcaagcgcaatcggtcgggccattg<br>aggacgaggctcgcttcggtcgtatccgtgaccttgaagctaagcacttcaagaaa<br>aacgttgaggaacaactcaacaagcgcgtagggcacgtctacaagaaagcattt<br>atgcaagttgtcgaggctgacatgctctctaagggtctactcggtggcgaggcgtgg<br>tcttcgtggcataaggaagactctattcatgtaggagtacgctgcatcgagatgctca<br>ttgagtcaaccggaatggttagcttacaccgccaaaatgctggcgtagtaggtcaa<br>gactctgagactatcgaactcgcacctgaatacgctgaggctatcgcaacccgtgc<br>aggtgcgctggctggcatctctccgatgttccaaccttgcgtagttcctcctaagccgt	

		ggactggcattactggtggtggctattgggctaacggtcgtcgtcctctggcgctggt<br>gcgtactcacagtaagaaagcactgatgcgctacgaagacgtttacatgcctgag<br>gtgtacaaagcgattaacattgcgcaaaacaccgcatggaaaatcaacaagaa<br>agtcctagcggtcgccaacgtaatcaccaagtggaagcattgtccggtcgaggac<br>atccctgcgattgagcgtgaagaactcccgatgaaaccggaagacatcgacatg<br>aatcctgaggctctcaccgcgtggaaacgtgctgccgctgctgtgtaccgcaagga<br>caaggctcgcaagtctcgccgtatcagccttgagttcatgcttgagcaagccaata<br>agtttgctaaccataaggccatctggttcccttacaacatggactggcgcggtcgtgt<br>ttacgctgtgtcaatgttcaacccgcaaggtaacgatatgaccaaaggactgcttac<br>gctggcgaaaggtaaaccaatcggtaaggaaggttactactggctgaaaatcca<br>cggtgcaaactgtgcgggtgtcgataaggttccgttccctgagcgcatcaagttcatt<br>gaggaaaaccacgagaacatcatggcttgcgctaagtctccactggagaacactt<br>ggtgggctgagcaagattctccgttctgcttccttgcgttctgctttgagtacgctgggg<br>tacagcaccacggcctgagctataactgctcccttccgctggcgtttgacgggtcttg<br>ctctggcatccagcacttctccgcgatgctccgagatgaggtaggtggtcgcgcggt<br>taacttgcttcctagtgaaaccgttcaggacatctacgggattgttgctaagaaagtc<br>aacgagattctacaagcagacgcaatcaatgggaccgataacgaagtagttacc<br>gtgaccgatgagaacactggtgaaatctctgagaaagtcaagctgggcactaag<br>gcactggctggtcaatggctggcttacggtgttactcgcagtgtgactaagcgttcag<br>tcatgacgctggcttacgggtccaaagagttcggcttccgtcaacaagtgctggaa<br>gataccattcagccagctattgattccggcaagggtctgatgttcactcagccgaat<br>caggctgctggatacatggctaagctgatttgggaatctgtgagcgtgacggtggta<br>gctgcggttgaagcaatgaactggcttaagtctgctgctaagctgctggctgctgag<br>gtcaaagataagaagactggagagattcttcgcaagcgttgcgctgtgcattgggt<br>aactcctgatggtttccctgtgtggcaggaatacaagaagcctattcagacgcgctt<br>gaacctgatgttcctcggtcagttccgcttacagcctaccattaacaccaacaaaga<br>tagcgagattgatgcacacaaacaggagtctggtatcgctcctaactttgtacacag<br>ccaagacggtagccaccttcgtaagactgtagtgtgggcacacgagaagtacgg<br>aatcgaatcttttgcactgattcacgactccttcggtaccattccggctgacgctgcga<br>acctgttcaaagcagtgcgcgaaactatggttgacacatatgagtcttgtgatgtact<br>ggctgatttctacgaccagttcgctgaccagttgcacgagtctcaattggacaaaat<br>gccagcacttccggctaaaggtaacttgaacctccgtgacatcttagagtcggactt<br>cgcgttcgcataa	
SupD	SupD-tRNA coding sequence (BBa_K228001) [20]	caattcggagagatgccggagcggctgaacggaccggtctctaaaaccggagta<br>ggggcaactctaccgggggttcaaatcccccctctctccgccactacagatccttagc<br>gaaagctaaggattttttttaagct	
LAA + stop codon		aggcctgcagcaaacgacgaaaactacgctttagcagcttaa	
LVA + stop codon		aggcctgctgcaaacgacgaaaactacgctttagtagcttaa	
P_{BAD}	*araBAD* promoter [12]	aagaaaccaattgtccatattgcatcagacattgccgtcactgcgtcttttactggctc<br>ttctcgctaaccaaaccggtaaccccgcttattaaaagcattctgtaacaaagcgg<br>gaccaaagccatgacaaaaacgcgtaacaaaagtgtctataatcacggcagaa<br>aagtccacattgattatttgcacggcgtcacactttgctatgccatagcatttttatccat<br>aagattagcggatcctacctgacgctttttatcgcaactctctactgtttctccat	
$P_{J231119}$		TTGACAGCTAGCTCAGTCCTAGGTATAATACTAGT	
LKsg3		aattcgctagcccaaaaaaa	
SG6		gagttgcgataaaaagcgtc	
gRNA		GTTTTAGAGCTAGAAATAGCAAgttaaaataagGCTAGTCCG<br>TTATCAACTTGAAAAAGTGGCACCGAGTCGGTGC	
dCas9		ATGGATAAGAAATACTCAATAGGCTTAGCTATCGGCACA<br>AATAGCGTCGGATGGGCGGTGATCACTGATGAATATAA<br>GGTTCCGTCTAAAAAGTTCAAGGTTCTGGGAAATACAGA<br>CCGCCACAGTATCAAAAAAAATCTTATAGGGGCTCTTTT<br>ATTTGACAGTGGAGAGACAGCGGAAGCGACTCGTCTCA<br>AACGGACAGCTCGTAGAAGGTATACACGTCGGAAGAAT<br>CGTATTTGTTATCTACAGGAGATTTTTTCAAATGAGATGG<br>CGAAAGTAGATGATAGTTTCTTTCATCGACTTGAAGAGT<br>CTTTTTTGGTGGAAGAAGACAAGAAGCATGAACGTCATC<br>CTATTTTTGGAAATATAGTAGATGAAGTTGCTTATCATGA	

		GAAATATCCAACTATCTATCATCTGCGAAAAAAATTGGTA<br>GATTCTACTGATAAAGCGGATTTGCGCTTAATCTATTTG<br>GCCTTAGCGCATATGATTAAGTTTCGTGGTCATTTTTTGA<br>TTGAGGGAGATTTAAATCCTGATAATAGTGATGTGGACA<br>AACTATTTATCCAGTTGGTACAAACCTACAATCAATTATT<br>TGAAGAAAACCCTATTAACGCAAGTGGAGTAGATGCTAA<br>AGCGATTCTTTCTGCACGATTGAGTAAATCAAGACGATT<br>AGAAAATCTCATTGCTCAGCTCCCCGGTGAGAAGAAAAA<br>TGGCTTATTTGGGAATCTCATTGCTTTGTCATTGGGTTTG<br>ACCCCTAATTTTAAATCAAATTTTGATTTGGCAGAAGATG<br>CTAAATTACAGCTTTCAAAAGATACTTACGATGATGATTT<br>AGATAATTTATTGGCGCAAATTGGAGATCAATATGCTGA<br>TTTGTTTTTGGCAGCTAAGAATTTATCAGATGCTATTTTA<br>CTTTCAGATATCCTAAGAGTAAATACTGAAATAACTAAGG<br>CTCCCCTATCAGCTTCAATGATTAAACGCTACGATGAAC<br>ATCATCAAGACTTGACTCTTTTAAAAGCTTTAGTTCGACA<br>ACAACTTCCAGAAAAGTATAAAGAAATCTTTTTTGATCAA<br>TCAAAAAACGGATATGCAGGTTATATTGATGGGGGAGCT<br>AGCCAAGAAGAATTTTATAAATTTATCAAACCAATTTTAG<br>AAAAAATGGATGGTACTGAGGAATTATTGGTGAAACTAA<br>ATCGTGAAGATTTGCTGCGCAAGCAACGGACCTTTGACA<br>ACGGCTCTATTCCCCATCAAATTCACTTGGGTGAGCTGC<br>ATGCTATTTTGAGAAGACAAGAAGACTTTTATCCATTTTT<br>AAAAGACAATCGTGAGAAGATTGAAAAAATCTTGACTTTT<br>CGAATTCCTTATTATGTTGGTCCATTGGCGCGTGGCAAT<br>AGTCGTTTTGCATGGATGACTCGGAAGTCTGAAGAAACA<br>ATTACCCCATGGAATTTTGAAGAAGTTGTCGATAAAGGT<br>GCTTCAGCTCAATCATTTATTGAACGCATGACAAACTTTG<br>ATAAAAATCTTCCAAATGAAAAAGTACTACCAAAACATAG<br>TTTGCTTTATGAGTATTTTACGGTTTATAACGAATTGACA<br>AAGGTCAAATATGTTACTGAAGGAATGCGAAAACCAGCA<br>TTTCTTTCAGGTGAACAGAAGAAAGCCATTGTTGATTTAC<br>TCTTCAAAACAAATCGAAAAGTAACCGTTAAGCAATTAAA<br>AGAAGATTATTTCAAAAAAATAGAATGTTTTGATAGTGTT<br>GAAATTTCAGGAGTTGAAGATAGATTTAATGCTTCATTAG<br>GTACCTACCATGATTTGCTAAAAATTATTAAAGATAAAGA<br>TTTTTTGGATAATGAAGAAAATGAAGATATCTTAGAGGAT<br>ATTGTTTTAACATTGACCTTATTTGAAGATAGGGAGATGA<br>TTGAGGAAAGACTTAAAACATATGCTCACCTCTTTGATG<br>ATAAGGTGATGAAACAGCTTAAACGTCGCCGTTATACTG<br>GTTGGGGACGTTTGTCTCGAAAATTGATTAATGGTATTA<br>GGGATAAGCAATCTGGCAAAACAATATTAGATTTTTTGAA<br>ATCAGATGGTTTTGCCAATCGCAATTTTATGCAGCTGAT<br>CCATGATGATAGTTTGACATTTAAAGAAGACATTCAAAAA<br>GCACAAGTGTCTGGACAAGGCGATAGTTTACATGAACAT<br>ATTGCAAATTTAGCTGGTAGCCCTGCTATTAAAAAAGGT<br>ATTTTACAGACTGTAAAAGTTGTTGATGAATTGGTCAAAG<br>TAATGGGGCGGCATAAGCCAGAAAATATCGTTATTGAAA<br>TGGCACGTGAAAATCAGACAACTCAAAAGGGCCAGAAA<br>AATTCGCGAGAGCGTATGAAACGAATCGAAGAAGGTAT<br>CAAAGAATTAGGAAGTCAGATTCTTAAAGAGCATCCTGT<br>TGAAAATACTCAATTGCAAAATGAAAAGCTCTATCTCTAT<br>TATCTCCAAAATGGAAGAGACATGTATGTGGACCAAGAA<br>TTAGATATTAATCGTTTAAGTGATTATGATGTCGATGCCA<br>TTGTTCCACAAAGTTTCCTTAAAGACGATTCAATAGACAA<br>TAAGGTCTTAACGCGTTCTGATAAAAATCGTGGTAAATC<br>GGATAACGTTCCAAGTGAAGAAGTAGTCAAAAAGATGAA<br>AAACTATTGGAGACAACTTCTAAACGCCAAGTTAATCAC<br>TCAACGTAAGTTTGATAATTTAACGAAAGCTGAACGTGG<br>AGGTTTGAGTGAACTTGATAAAGCTGGTTTTATCAAACG<br>CCAATTGGTTGAAACTCGCCAAATCACTAAGCATGTGGC<br>ACAAATTTTGGATAGTCGCATGAATACTAAATACGATGAA	

		AATGATAAACTTATTCGAGAGGTTAAAGTGATTACCTTAA<br>AATCTAAATTAGTTTCTGACTTCCGAAAAGATTTCCAATT<br>CTATAAAGTACGTGAGATTAACAATTACCATCATGCCCAT<br>GATGCGTATCTAAATGCCGTCGTTGGAACTGCTTTGATT<br>AAGAAATATCCAAAACTTGAATCGGAGTTTGTCTATGGT<br>GATTATAAAGTTTATGATGTTCGTAAAATGATTGCTAAGT<br>CTGAGCAAGAAATAGGCAAAGCAACCGCAAAATATTTCT<br>TTTACTCTAATATCATGAACTTCTTCAAAACAGAAATTAC<br>ACTTGCAAATGGAGAGATTCGCAAACGCCCTCTAATCGA<br>AACTAATGGGGAAACTGGAGAAATTGTCTGGGATAAAG<br>GGCGAGATTTTGCCACAGTGCGCAAAGTATTGTCCATG<br>CCCCAAGTCAATATTGTCAAGAAAACAGAAGTACAGACA<br>GGCGGATTCTCCAAGGAGTCAATTTTACCAAAAAGAAAT<br>TCGGACAAGCTTATTGCTCGTAAAAAAGACTGGGATCCA<br>AAAAAATATGGTGGTTTTGATAGTCCAACGGTAGCTTATT<br>CAGTCCTAGTGGTTGCTAAGGTGGAAAAAGGGAAATCG<br>AAGAAGTTAAAATCCGTTAAAGAGTTACTAGGGATCACA<br>ATTATGGAAAGAAGTTCCTTTGAAAAAAATCCGATTGACT<br>TTTTAGAAGCTAAAGGATATAAGGAAGTTAAAAAAGACTT<br>AATCATTAAACTACCTAAATATAGTCTTTTTGAGTTAGAA<br>AACGGTCGTAAACGGATGCTGGCTAGTGCCGGAGAATT<br>ACAAAAAGGAAATGAGCTGGCTCTGCCAAGCAAATATGT<br>GAATTTTTTATATTTAGCTAGTCATTATGAAAAGTTGAAG<br>GGTAGTCCAGAAGATAACGAACAAAAACAATTGTTTGTG<br>GAGCAGCATAAGCATTATTTAGATGAGATTATTGAGCAA<br>ATCAGTGAATTTTCTAAGCGTGTTATTTTAGCAGATGCCA<br>ATTTAGATAAAGTTCTTAGTGCATATAACAAACATAGAGA<br>CAAACCAATACGTGAACAAGCAGAAAATATTATTCATTTA<br>TTTACGTTGACGAATCTTGGAGCTCCCGCTGCTTTTAAA<br>TATTTTGATACAACAATTGATCGTAAACGATATACGTCTA<br>CAAAAGAAGTTTTAGATGCCACTCTTATCCATCAATCCAT<br>CACTGGTCTTTATGAAACACGCATTGATTTGAGTCAGCT<br>AGGAGGTGAC	
$P_{BAD\ \_RD1}$		atagcatttttatccataagattagcggatcctacctgacgctttttatcgcaactctcta<br>ctgtttctccataccgtttttttgggctagc	
ExsA	ExsA gene [76]	atgcaaggagccaaatctcttggccgaaagcagataacgtcttgtcattggaacatt<br>ccaactttcgaatacagggtaaacaaggaagagggcgtatatgttctgctcgagg<br>gcgaactgaccgtccaggacatcgattccactttttgcctggcgcctggcgagttgct<br>tttcgtccgccgcggaagctatgtcgtaagtaccaagggaaaggacagccgaata<br>ctctggattccattatctgcccagtttctacaaggcttcgtccagcgcttcggcgcgct<br>gttgagtgaagtcgagcgttgcgacgagcccgtgccgggcatcatcgcgttcgctg<br>ccacgcctctgctggccggttgcgtcaaggggttgaaggaattgcttgtgcatgagc<br>atccgccgatgctcgcctgcctgaagatcgaggagttgctgatgctcttcgcgttcag<br>tccgcaggggccgctgctgatgtcggtcctgcggcaactgagcaaccggcatgtc<br>gagcgtctgcagctattcatggagaagcactacctcaacgagtggaagctgtccg<br>acttctcccgcgagttcggcatggggctgaccaccttcaaggagctgttcggcagtg<br>tctatggggtttcgccgcgcgcctggatcagcgagcggagaatcctctatgcccatc<br>agttgctgctcaacagcgacatgagcatcgtcgacatcgccatggaggcgggcttt<br>tccagtcagtcctatttcacccagagctatcgccgccgtttcggctgcacgccgagc<br>cgctcgcggcaggggaaggacgaatgccgggctaaaaataactga	
ExsD	ExsD gene [76]	atggagcaggaagacgataagcagtactcccgagaagcggtgttcgctggcag<br>gcgggtatccgtggtgggctcggacgcccgctcgcggggtcgggtgccgggttac<br>gcatcgagcagtttgtatcgtgagtccggaatcatcagtgcgcggcaactggcgttg<br>ctgcagcggatgctgccgcgcctgcggctggagcaactgttccgctgcgagtggtt<br>gcagcagcgcctggcgcgcggcctggcgctggggcgcgaagaggtgcggcag<br>attctcctctgcgcggcgcaggacgacgacggctggtgctccgaactgggcgacc<br>gggtcaacctcgccgtgccgcagtcgatgatcgactgggtcctgctgccggtctatg<br>gctggtgggaaagcctgctcgaccaggcgatccccggctggcgcctgtcgctggt<br>ggagctggagacccagtcccggcaactgcgagtcaagtccgaattctggtcccgc<br>gtggccgagctggagccggagcaggcccgcgaggaactggccagggtcgcca<br>agtgccaggcgcgcacccaggaacaggtggccgaactggccggcaagctgga	

		gacggcttcggcactggcgaagagcgcctggccgaactggcagcggggcatgg cgacgctgctcgccagcggcgggctggccggcttcgagccgatccccgaggtcct cgaatgcctctggcaacctctctgccggctggacgacgacgtcggcgcggcgga cgccgtccaggcctggctgcacgaacgcaacctgtgccaggcacaggatcactt ctactggcagagctga	
pexsD	Promoter [76]	gaaggacgaatgccgggctaaaaataactgacgtttttgaaagcccggtagcgg ctgcatgagtagaatcggcccaaat	
P_{lacO}	PLlacO-1 promoter [74]	**aattgtgagcggataacaa**ttgacattgtgagcggataacaagatactgagcac atcagcaggacgcactgacc	
$P_{laco/teto}$		tacaacgtcgtgttaaattgtgagcggataacaatttagttgacatttatgcttccggct cgtataattccacccctatcagtgatagagagcgttacccaac	
$P^{*}_{laco/teto}$		ttttcagcaggacgcactgacctTTGTGAGCGGATAACAATTTAGTT GACATTTATGCTTCCGGCTCGTATAATTCCACCCCTATC AGTGATAGAGAgatactgagcacatat	
P_{lacO1}		ttgacattgtgagcggataacaagatactgagcacatcagcaggacgcactgacc	PLlacO-1_Deleting 1 binding site
P_{lux}	Lux promoter, BBa_R0062 [75]	**acct**gtaggatcgtacaggtttacgcaagaaaatggtttgtta**tag**tcgaataaa	
$P_{lux(AAT)}$		**aatt**gtaggatcgtacaggtttacgcaagaaaatggtttgttatagtcgaataaa	The "**acct**" of *Plux* (in bold) was mutated to "**aatt**" by site directed Mutagenesis.
$P_{lux(TGT)}$		**tgtt**gtaggatcgtacaggtttacgcaagaaaatggtttgttatagtcgaataaa	The "**acct**" of *Plux* (in bold) was mutated to "**tgtt**" by site directed Mutagenesis.
$P_{lux(LBL)}$		acctgtaggatcgtacaggtttacgcaagaaaatggtttgtta**ctt**tcgaataaa	The "**tag**" of *Plux* (in bold) was mutated to "**ctt**" by site directed Mutagenesis.
P_{luxM56}		**tggg**gtaggatcgtacaggtttacgcaagaaaatggtttgttatagtcgaataaa	The "**acct**" of *Plux* (in bold) was mutated to "**TGGG**" by site directed Mutagenesis.
$p_{lux/laco}$		acctgtaggatcgtacaggtttacttgtgagcggataacaatatagtgtgtggaattgt gagcggataacaatt	
$p_{lux/teto}$		acctgtaggatcgtacaggtttacgcaagaaaatggtttgttatagtcgaatatccct atcagtgatagaga	
P_{rhlR}		tcctgtgaaatctggcagttaccgttagctttcgaattggctaaaaagtgttc	
P_{teto}	PLtetO-1 promoter [74]	tccctatcagtgatagagattgacatccctatcagtgatagagatactgagcacatc agcaggacgcactgacc	
P_{teto*}	PLtetO* promoter [76]	ttttcagcaggacgcactgacctccctatcagtgatagagattgacatccctatcagt gatagagatactgagcacatat	
PT7		taatacgactcactatagggaga	
RBS1	BBa_B0030	attaaagaggagaaa	
RBS2	BBa_B0031	tcacacaggaaacc	
RBS3	BBa_B0034	aaagaggagaaa	
RiboJ	sTRSV HHRz	agctgtcaccggatgtgctttccggtctgatgagtccgtgaggacgaaacagcctct acaaataattttgtttaa	

rrnB T1 terminator	transcription terminator T1 from the *E.coli* rrnB gene	caaataaaacgaaaggctcagtcgaaagactgggcctttcgttttatctgttgtttgtc<br>ggtgaacgctctcctgagtaggacaaat	
p15A	medium-copy-number p15A origin of replication	cggtcgttcgactgcggcgagcggaaatggcttacgaacggggcggagatttcct<br>ggaagatgccaggaagatacttaacagggaagtgagagggccgcggcaaagc<br>cgtttttccataggctccgcccccctgacaagcatcacgaaatctgacgctcaaatc<br>agtggtggcgaaacccgacaggactataaagataccaggcgtttccccctggcg<br>gctccctcgtgcgctctcctgttcctgcctttcggtttaccggtgtcattccgctgttatgg<br>ccgcgtttgtctcattccacgcctgacactcagttccgggtaggcagttcgctccaag<br>ctggactgtatgcacgaaccccccgttcagtccgaccgctgcgccttatccggtaac<br>tatcgtcttgagtccaacccggaaagacatgcaaaagcaccactggcagcagcc<br>actggtaattgatttagaggagttagtcttgaagtcatgcgccggttaaggctaaact<br>gaaaggacaagttttggtgactgcgctcctccaagccagttacctcggttcaaaga<br>gttggtagctcagagaaccttcgaaaaaccgccctgcaaggcggttttttcgttttca<br>gagcaagagattacgcgcagaccaaaacgatctcaagaagatcatcttattaatc<br>agataaaatatttctagatttcagtgcaatttatctcttcaaatgtagcacctgaagtca<br>gccccatacgatataagttgtt	
pSC101	Low-copy replication origin	gtacgggttttgctgcccgcaaacgggctgttctggtgttgctagtttgttatcagaatc<br>gcagatccggcttcaggtttgccggctgaaagcgctatttcttccagaattgccatga<br>tttttccccacgggaggcgtcactggctcccgtgttgtcggcagctttgattcgataa<br>gcagcatcgcctgtttcaggctgtctatgtgtgactgttgagctgtaacaagttgtctca<br>ggtgttcaatttcatgttctagttgctttgttttactggtttcacctgttctattaggtgttacat<br>gctgttcatctgttacattgtcgatctgttcatggtgaacagctttaaatgcaccaaaaa<br>ctcgtaaaagctctgatgtatctatcttttttacaccgttttcatctgtgcatatggacagtt<br>ttccctttgatatctaacggtgaacagttgttctacttttgtttgttagtcttgatgcttcactg<br>atagatacaagagccataagaacctcagatccttccgtatttagccagtatgttctct<br>agtgtggttcgttgtttttgcgtgagccatgagaacgaaccattgagatcatgcttactt<br>tgcatgtcactcaaaaattttgcctcaaaactggtgagctgaatttttgcagttaaagc<br>atcgtgtagtgtttttcttagtccgttacgtaggtaggaatctgatgtaatggttgttggta<br>ttttgtcaccattcatttttatctggttgttctcaagttcggttacgagatccatttgtctatct<br>agttcaacttggaaaatcaacgtatcagtcgggcggcctcgcttatcaaccaccaa<br>tttcatattgctgtaagtgtttaaatctttacttattggtttcaaaacccattggttaagcctt<br>ttaaactcatggtagttattttcaagcattaacatgaacttaaattcatcaaggctaatc<br>tctatatttgccttgtgagttttcttttgtgttagttcttttaataaccactcataaatcctcata<br>gagtatttgttttcaaaagacttaacatgttccagattatattttatgaatttttttaactgg<br>aaaagataaggcaatatctcttcactaaaaactaattctaatttttcgcttgagaactt<br>ggcatagtttgtccactggaaaatctcaaagcctttaaccaaaggattcctgatttcc<br>acagttctcgtcatcagctctctggttgctttagctaatacaccataagcattttccctac<br>tgatgttcatcatctgagcgtattggttataagtgaacgataccgtccgttctttccttgta<br>gggttttcaatcgtggggttgagtagtgccacacagcataaaattagcttggtttcatg<br>ctccgttaagtcatagcgactaatcgctagttcatttgctttgaaaacaactaattcag<br>acatacatctcaattggtctaggtgattttaatcactataccaattgagatgggctagt<br>caatgataattactagtccttttcctttgagttgtgggtatctgtaaattctgctagaccttt<br>gctggaaaacttgtaaattctgctagaccctctgtaaattccgctagacctttgtgtgttt<br>tttttgtttatattcaagtggttataatttatagaataaagaaagaataaaaaaagata<br>aaaagaatagatcccagccctgtgtataactcactactttagtcagttccgcagtatt<br>acaaaaggatgtcgcaaacgctgtttgctcctctacaaaacagaccttaaaaccct<br>aaaggcttaagtagcaccctcgcaagctcgggcaaatcgctgaatattccttttgtct<br>ccgaccatcaggcacctgagtcgctgtctttttcgtgacattcagttcgctgcgctcac<br>ggctctggcagtgaatgggggtaaatggcactacaggcgccttttatggattcatgc<br>aaggaaactacccataatacaagaaaagcccgtcacgggcttctcagggcgtttt<br>atggcgggtctgctatgtggtgctatctgactttttgctgttcagcagttcctgccctctg<br>attttccagtctgaccacttcggattatcccgtgacaggtcattcagactggctaatgc<br>acccagtaaggcagcggtatcatcaacaggcttacccgtcttactgtccctagt	
ColE1	High-copy replication origin	cgttcggctgcggcgagcggtatcagctcactcaaaggcggtaatacggttatcca<br>cagaatcaggggataacgcaggaaagaacatgtgagcaaaaggccagcaaa<br>aggccaggaaccgtaaaaaggccgcgttgctggcgtttttccataggctccgcccc<br>cctgacgagcatcacaaaaatcgacgctcaagtcagaggtggcgaaacccgac<br>aggactataaagataccaggcgtttccccctggaagctccctcgtgcgctctcctgtt<br>ccgaccctgccgcttaccggatacctgtccgcctttctcccttcgggaagcgtggcg<br>ctttctcaatgctcacgctgtaggtatctcagttcggtgtaggtcgttcgctccaagctg	

		ggctgtgtgcacgaaccccccgttcagcccgaccgctgcgccttatccggtaactat<br>cgtcttgagtccaacccggtaagacacgacttatcgccactggcagcagccactg<br>gtaacaggattagcagagcgaggtatgtaggcggtgctacagagttcttgaagtgg<br>tggcctaactacggctacactagaaggacagtatttggtatctgcgctctgctgaag<br>ccagttaccttcggaaaaagagttggtagctcttgatccggcaaacaaaccaccg<br>ctggtagcggtggtttttttgtttgcaagcagcagattacgcgcagaaaaaaaggat<br>ctcaagaagatcctttgatcttttctacggggtctgacgctcagtggaacgaaaactc<br>acgttaagggattttggtcatg	
cmR		aaattacgccccgccctgccactcatcgcagtactgttgtaattcattaagcattctgc<br>cgacatggaagccatcacaaacggcatgatgaacctgaatcgccagcggcatc<br>agcaccttgtcgccttgcgtataatatttgcccatggtgaaaacgggggcgaagaa<br>gttgtccatattggccacgtttaaatcaaaactggtgaaactcacccagggattggct<br>gagacgaaaaacatattctcaataaaccctttagggaaataggccaggttttcacc<br>gtaacacgccacatcttgcgaatatatgtgtagaaactgccggaaatcgtcgtggta<br>ttcactccagagcgatgaaaacgtttcagtttgctcatggaaaacggtgtaacaag<br>ggtgaacactatcccatatcaccagctcaccgtctttcattgccatacgaaattccgg<br>atgagcattcatcaggcgggcaagaatgtgaataaaggccggataaaacttgtgc<br>ttatttttctttacggtctttaaaaaggccgtaatatccagctgaacggtctggttatagg<br>tacattgagcaactgactgaaatgcctcaaaatgttctttacgatgccattgggatat<br>atcaacggtggtatatccagtgatttttttctccattttagcttccttagctcctgaaaatct<br>cgataactcaaaaaatacgcccggtagtgatcttatttcattatggtgaaagttggaa<br>cctcttacgtgcccgatcaa	
ampR		gtaaacttggtctgacagttaccaatgcttaatcagtgaggcacctatctcagcgatc<br>tgtctatttcgttcatccatagttgcctgactccccgtcgtgtagataactacgatacgg<br>gagggcttaccatctggccccagtgctgcaatgataccgcgagacccacgctcac<br>cggctccagatttatcagcaataaaccagccagccggaagggccgagcgcaga<br>agtggtcctgcaactttatccgcctccatccagtctattaattgttgccgggaagctag<br>agtaagtagttcgccagttaatagtttgcgcaacgttgttgccattgctacaggcatcg<br>tggtgtcacgctcgtcgtttggtatggcttcattcagctccggttcccaacgatcaagg<br>cgagttacatgatcccccatgttgtgcaaaaaagcggttagctccttcggtcctccga<br>tcgttgtcagaagtaagttggccgcagtgttatcactcatggttatggcagcactgcat<br>aattctcttactgtcatgccatccgtaagatgcttttctgtgactggtgagtactcaacc<br>aagtcattctgagaatagtgtatgcggcgaccgagttgctcttgcccggcgtcaata<br>cgggataataccgcgccacatagcagaactttaaaagtgctcatcattggaaaac<br>gttcttcggggcgaaaactctcaaggatcttaccgctgttgagatccagttcgatgta<br>acccactcgtgcacccaactgatcttcagcatcttttactttcaccagcgtttctgggtg<br>agcaaaaacaggaaggcaaaatgccgcaaaaaagggaataagggcgacac<br>ggaaatgttgaatactcatactcttcctttttcaatattattgaagcatttatcagggttatt<br>gtctcatgagcggatacatatttgaatgtatttagaaaaataaacaaataggggttcc<br>gcgcacatttccccgaaaagtgccacct	
kanR		tcgaaccccagagtcccgctcagaagaactcgtcaagaaggcgatagaaggcg<br>atgcgctgcgaatcgggagcggcgataccgtaaagcacgaggaagcggtcagc<br>ccattcgccgccaagctcttcagcaatatcacgggtagccaacgctatgtcctgata<br>gcggtccgccacacccagccggccacagtcgatgaatccagaaaagcggccat<br>tttccaccatgatattcggcaagcaggcatcgccatgggtcacgacgagatcctcg<br>ccgtcgggcatgcgcgccttgagcctggcgaacagttcggctggcgcgagcccct<br>gatgctcttcgtccagatcatcctgatcgacaagaccggcttccatccgagtacgtg<br>ctcgctcgatgcgatgtttcgcttggtggtcgaatgggcaggtagccggatcaagcg<br>tatgcagccgccgcattgcatcagccatgatggatactttctcggcaggagcaagg<br>tgagatgacaggagatcctgccccggcacttcgcccaatagcagccagtcccttc<br>ccgcttcagtgacaacgtcgagcacagctgcgcaaggaacgcccgtcgtggcca<br>gccacgatagccgcgctgcctcgtcctgcagttcattcagggcaccggacaggtc<br>ggtcttgacaaaaagaaccgggcgcccctgcgctgacagccggaacacggcgg<br>catcagagcagccgattgtctgttgtgcccagtcatagccgaatagcctctccaccc<br>aagcggccggagaacctgcgtgcaatccatcttgttcaatcatgcgaaacgatcct<br>catcctgtctcttgatcagatcttgatcccctgcgccatcagatccttggcggcaaga<br>aagccatccagtttactttgcagggcttcccaaccttaccagagggcgccccagct<br>ggcaattcc	

15. Plasmid Maps

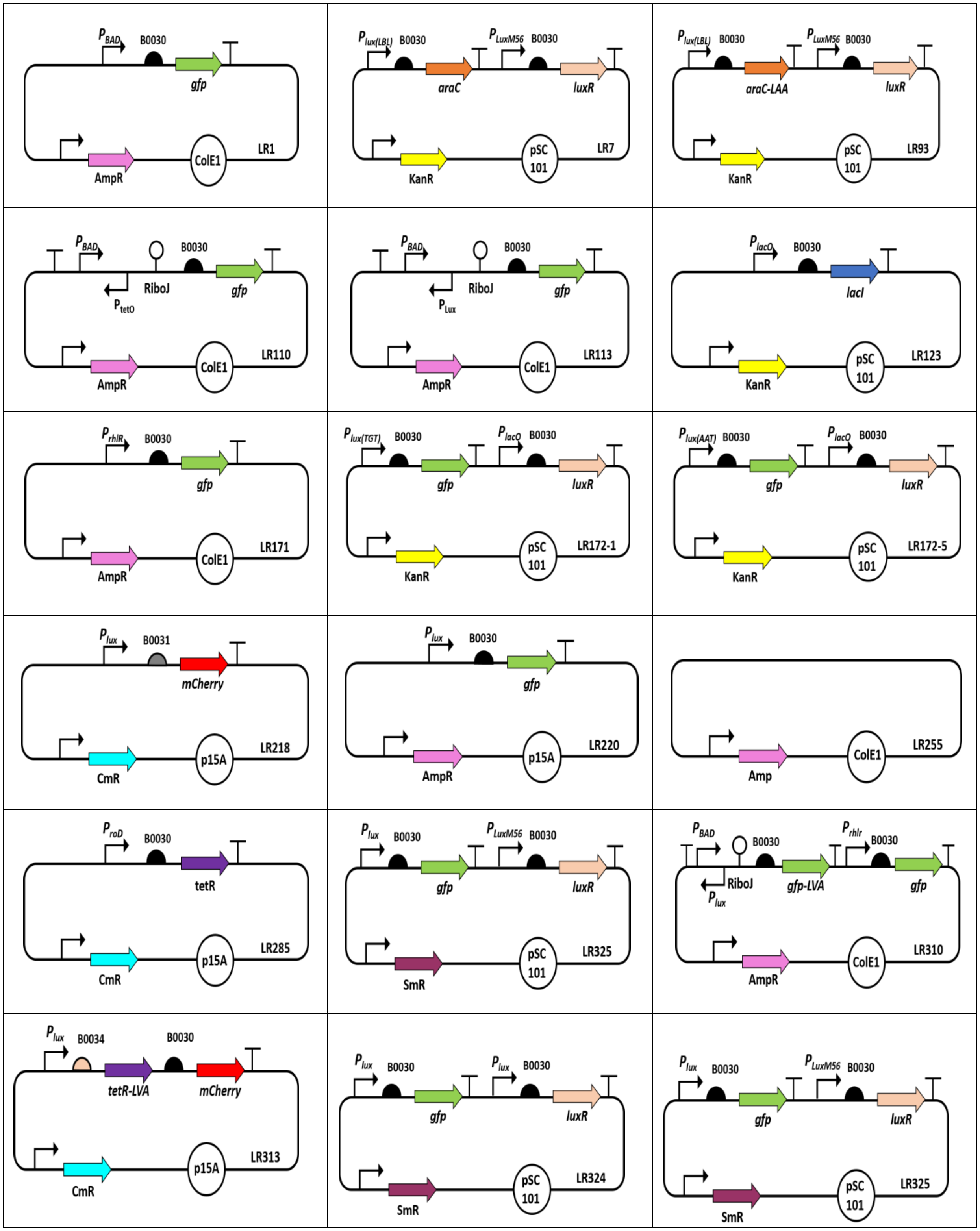

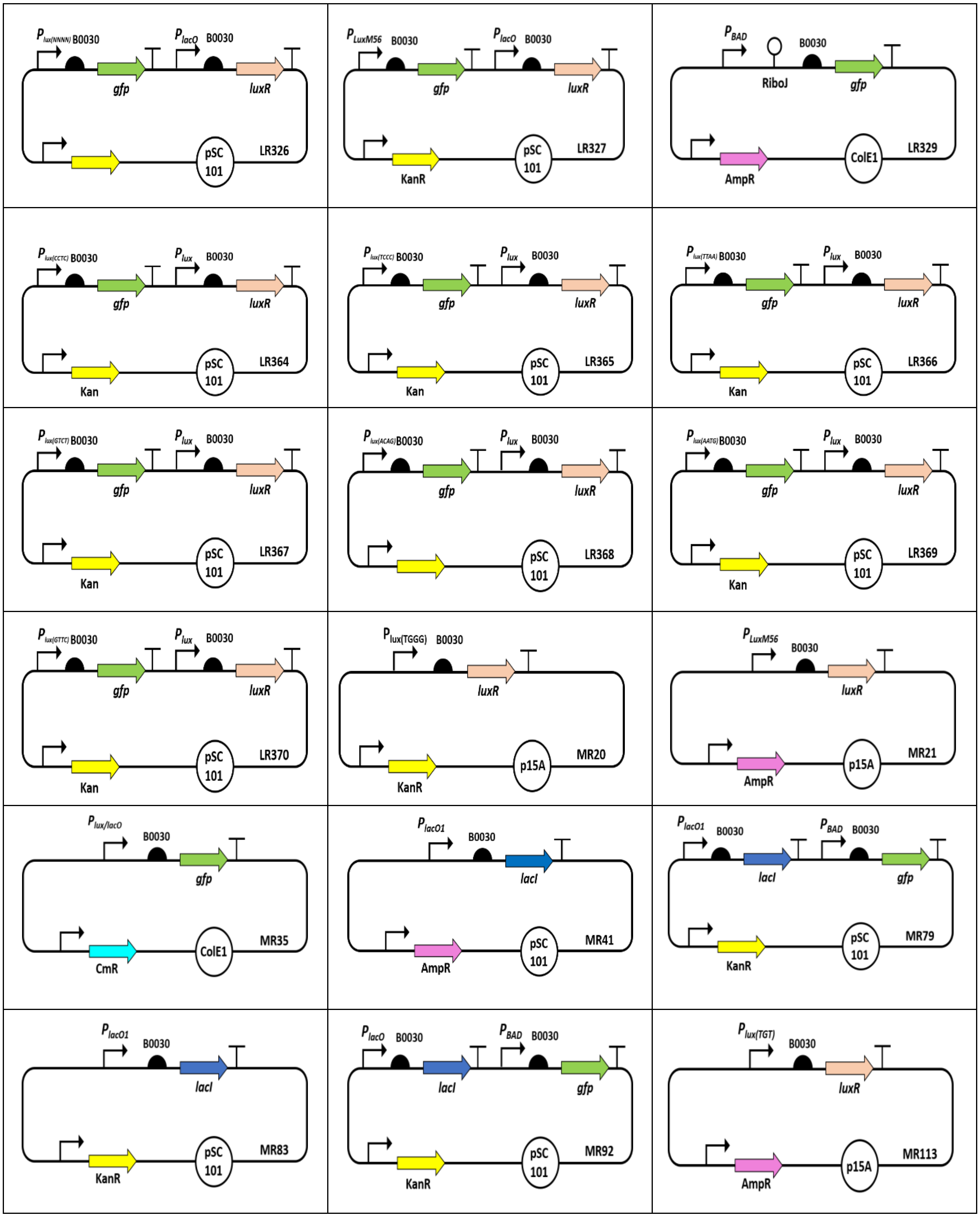
B0030
gfp
luxR
pSC 101
LR326
PluxM56
PlacO
KanR
LR327
PBAD
RiboJ
AmpR
ColE1
LR329
Plux
Kan
LR364
LR365
LR366
LR367
LR368
LR369
LR370
Plux(TGGG)
p15A
MR20
PLuxM56
MR21
Plux/lacO
CmR
MR35
PlacO1
lacI
MR41
MR79
MR83
MR92
Plux(TGT)
MR113

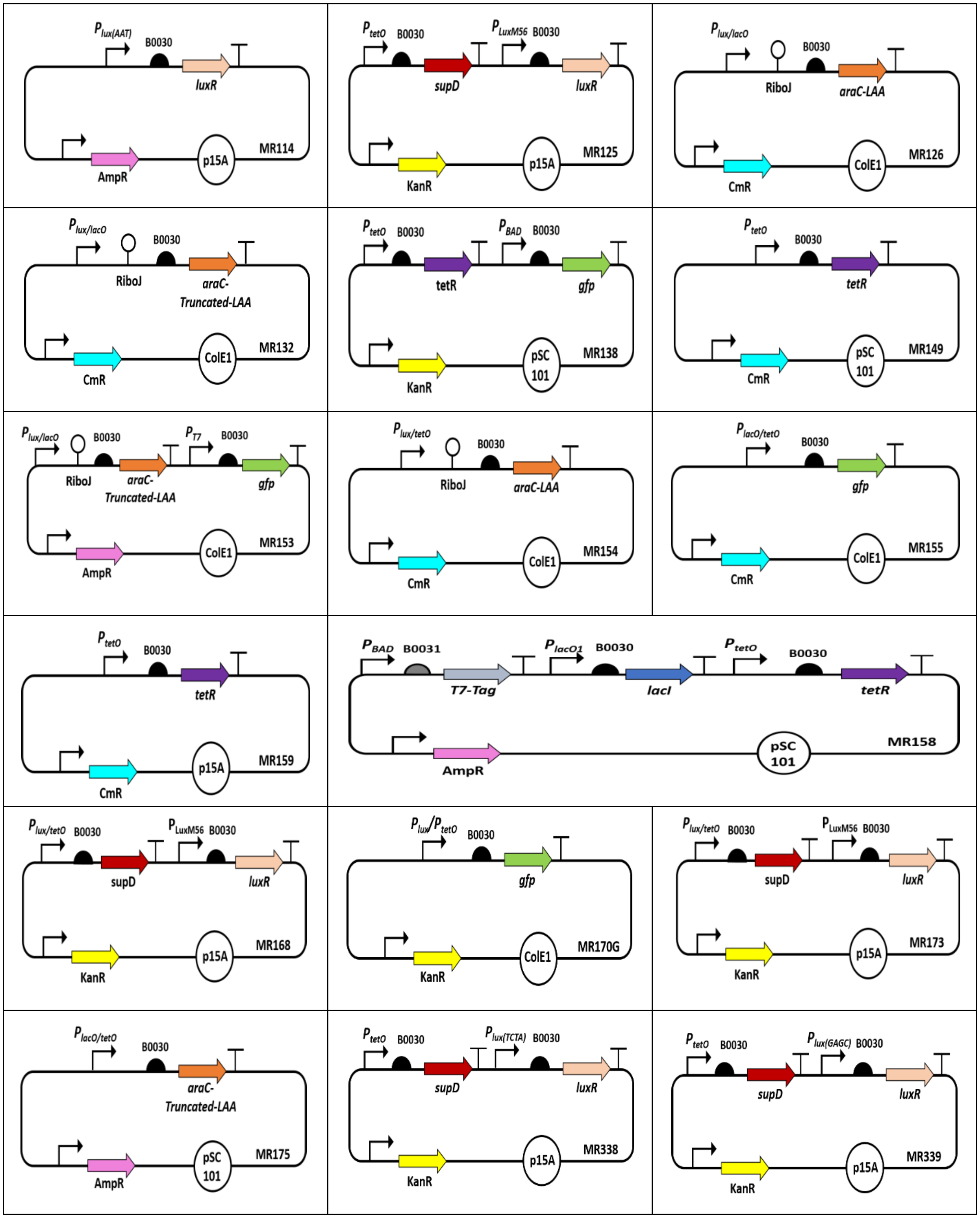
Plux(AAT) B0030 luxR AmpR p15A MR114
PtetO B0030 supD PLuxM56 B0030 luxR KanR p15A MR125
Plux/lacO RiboJ B0030 araC-LAA CmR ColE1 MR126
Plux/lacO RiboJ B0030 araC-Truncated-LAA CmR ColE1 MR132
PtetO B0030 tetR PBAD B0030 gfp KanR pSC 101 MR138
PtetO B0030 tetR CmR pSC 101 MR149
Plux/lacO RiboJ B0030 araC-Truncated-LAA PT7 B0030 gfp AmpR ColE1 MR153
Plux/tetO RiboJ B0030 araC-LAA CmR ColE1 MR154
PlacO/tetO B0030 gfp CmR ColE1 MR155
PtetO B0030 tetR CmR p15A MR159
PBAD B0031 T7-Tag PlacO1 B0030 lacI PtetO B0030 tetR AmpR pSC 101 MR158
Plux/tetO B0030 supD PLuxM56 B0030 luxR KanR p15A MR168
Plux/PtetO B0030 gfp KanR ColE1 MR170G
Plux/tetO B0030 supD PLuxM56 B0030 luxR KanR p15A MR173
PlacO/tetO B0030 araC-Truncated-LAA AmpR pSC 101 MR175
PtetO B0030 supD Plux(TCTA) B0030 luxR KanR p15A MR338
PtetO B0030 supD Plux(GAGC) B0030 luxR KanR p15A MR339

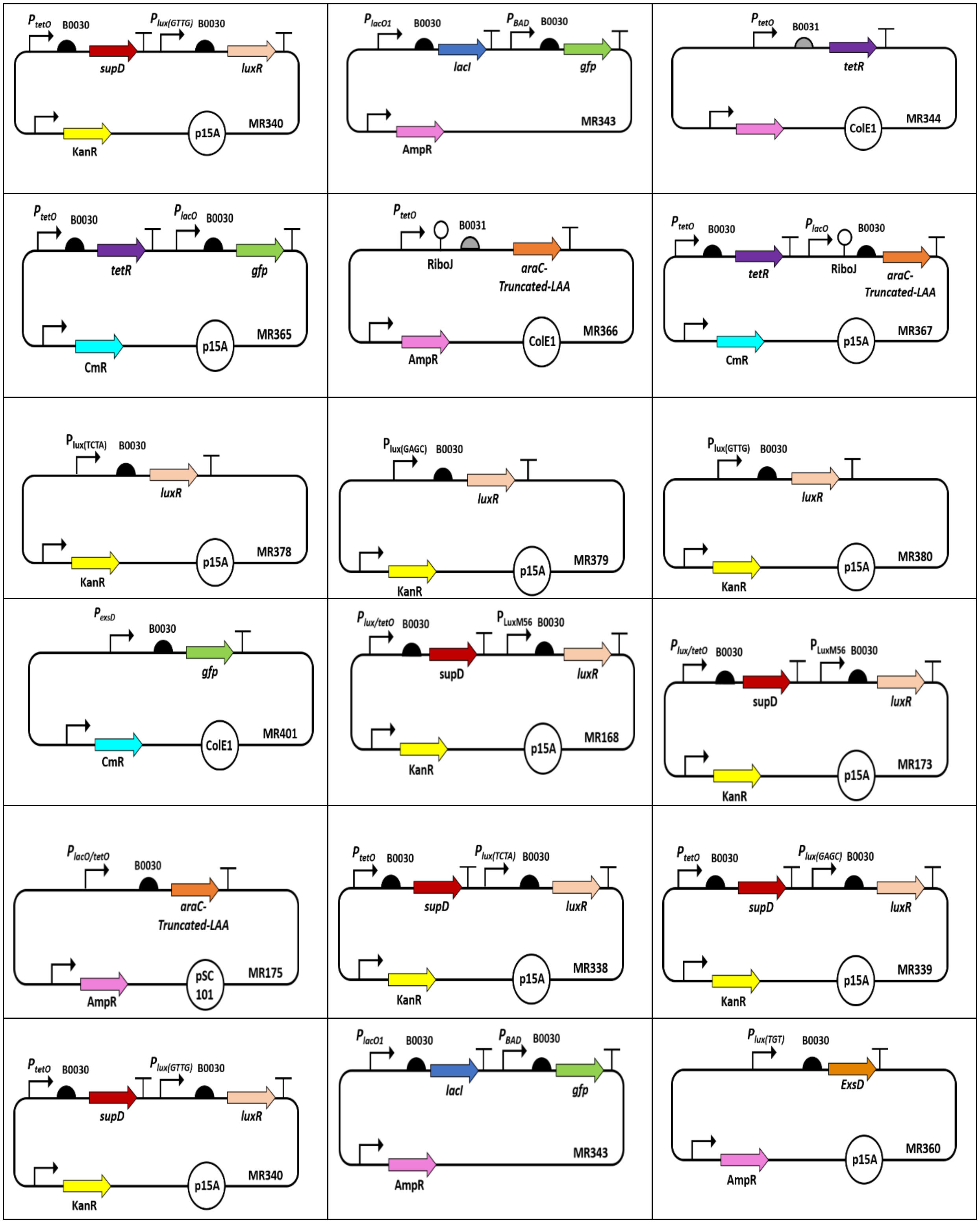
PtetO B0030 supD Plux(GTTG) B0030 luxR KanR p15A MR340
PlacO1 B0030 lacI PBAD B0030 gfp AmpR MR343
PtetO B0031 tetR ColE1 MR344
PtetO B0030 tetR PlacO B0030 gfp CmR p15A MR365
PtetO RiboJ B0031 araC-Truncated-LAA AmpR ColE1 MR366
PtetO B0030 tetR PlacO RiboJ B0030 araC-Truncated-LAA CmR p15A MR367
Plux(TCTA) B0030 luxR KanR p15A MR378
Plux(GAGC) B0030 luxR KanR p15A MR379
Plux(GTTG) B0030 luxR KanR p15A MR380
PexsD B0030 gfp CmR ColE1 MR401
Plux/tetO B0030 supD PLuxM56 B0030 luxR KanR p15A MR168
Plux/tetO B0030 supD PLuxM56 B0030 luxR KanR p15A MR173
PlacO/tetO B0030 araC-Truncated-LAA AmpR pSC 101 MR175
PtetO B0030 supD Plux(TCTA) B0030 luxR KanR p15A MR338
PtetO B0030 supD Plux(GAGC) B0030 luxR KanR p15A MR339
PtetO B0030 supD Plux(GTTG) B0030 luxR KanR p15A MR340
PlacO1 B0030 lacI PBAD B0030 gfp AmpR MR343
Plux(TGT) B0030 ExsD AmpR p15A MR360

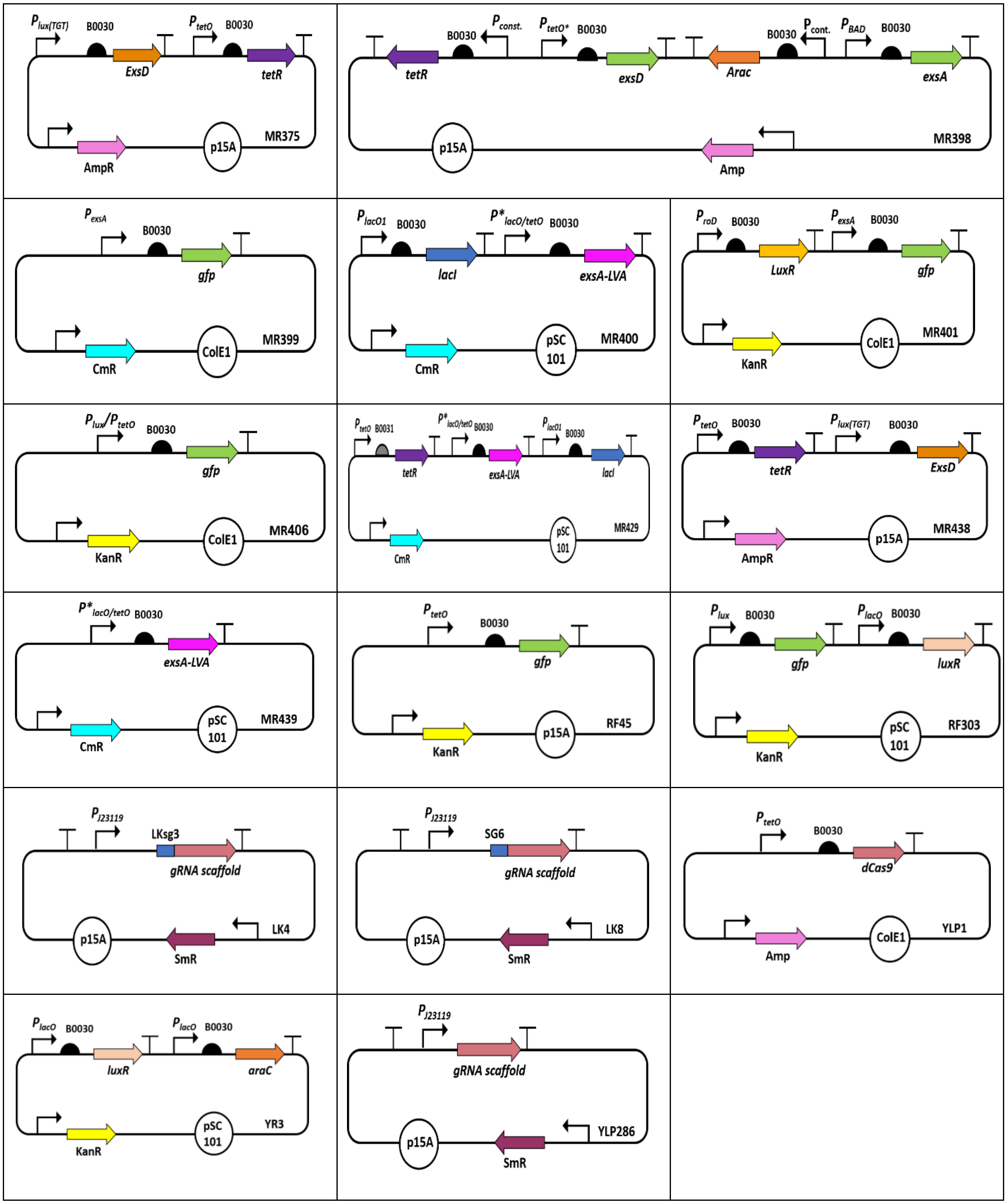
MR375
Plux(TGT)
B0030
ExsD
PtetO
B0030
tetR
AmpR
p15A
MR398
B0030
Pconst.
PtetO*
B0030
tetR
exsD
Arac
B0030
Pcont.
PBAD
B0030
exsA
p15A
Amp
MR399
PexsA
B0030
gfp
CmR
ColE1
MR400
PlacO1
B0030
lacI
P*lacO/tetO
B0030
exsA-LVA
CmR
pSC 101
MR401
ProD
B0030
LuxR
PexsA
B0030
gfp
KanR
ColE1
MR406
Plux/PtetO
B0030
gfp
KanR
ColE1
MR429
PtetO
B0031
tetR
P*lacO/tetO
B0030
exsA-LVA
PlacO1
B0030
lacI
CmR
pSC 101
MR438
PtetO
B0030
tetR
Plux(TGT)
B0030
ExsD
AmpR
p15A
MR439
P*lacO/tetO
B0030
exsA-LVA
CmR
pSC 101
RF45
PtetO
B0030
gfp
KanR
p15A
RF303
Plux
B0030
gfp
PlacO
B0030
luxR
KanR
pSC 101
PJ23119
LKsg3
gRNA scaffold
LK4
p15A
SmR
PJ23119
SG6
gRNA scaffold
LK8
p15A
SmR
PtetO
B0030
dCas9
YLP1
Amp
ColE1
PlacO
B0030
luxR
PlacO
B0030
araC
YR3
KanR
pSC 101
PJ23119
gRNA scaffold
YLP286
p15A
SmR

16. List of strains used in this study

Host Strain	Plasmids	Figure
E. coli 10-beta	LR123 + MR155 + MR159	Fig. 1d, Supplementary Figs. 13 and 17a
E. coli 10-beta	MR92 + MR159 + MR175	Fig. 1g
E. coli 10-beta	MR113 + MR149 + MR406	Fig. 2b
E. coli 10-beta	MR113 + MR138 + MR154	Fig. 2d
E. coli 10-beta	MR79 + MR114 + MR126	Fig. 2g, Supplementary Fig. 37b
E. coli 10-beta	MR125 + MR153 + MR158	Fig. 3c, Supplementary Fig. 44
E. coli 10-beta	MR153 + MR158 + MR338	Fig. 3g, Supplementary Fig.57b
E. coli 10-beta	MR153 + MR158 + MR340	Fig. 3g, Supplementary Fig.57d
E. coli 10-beta	MR153 + MR158 + MR339	Fig. 3g, Supplementary Fig.57f
E. coli 10-beta	MR153 + MR158 + MR125	Fig. 3g, Supplementary Fig.57h
E. coli 10-beta	LR7 + LR309 + LR313	Fig. 4c, Supplementary Fig. 71a
E. coli 10-beta	LR7 + LR218 + LR310	Fig. 4f, Supplementary Figs. 74a, 95 and 97
E. coli 10-beta	MR83 + MR155 + MR159	Supplementary Fig. 17b
E. coli 10-beta	LR1 + LR93	Supplementary Fig. 22a
E. coli 10-beta	MR400 + MR401 + MR375	Fig. 5c
E. coli 10-beta	MR401 + MR438 + MR439	Figs. 5e and 5f
E. coli 10-beta	MR401 + MR429 + MR360	Fig. 5g
E. coli 10-beta	RF303	Supplementary Fig. 26a - OL Wild type, Supplementary Fig. 27
E. coli 10-beta	LR324	Supplementary Fig. 26a - APF Wild type
E. coli 10-beta	LR327	Supplementary Fig. 26b - OL mutated
E. coli 10-beta	LR325	Supplementary Fig. 26b - APF mutated
E. coli 10-beta	LR172-5	Supplementary Fig. 27 - P_{luxAAT}
E. coli 10-beta	LR172-1	Supplementary Fig. 27 - P_{luxTGT}
E. coli 10-beta	MR21 + MR35 + MR83	Supplementary Fig. 48
E. coli 10-beta	MR149 + RF45	Supplementary Fig. 50
E. coli 10-beta	MR35 + MR41 + MR378	Supplementary Figs. 56a and c
E. coli 10-beta	MR35 + MR41 + MR380	Supplementary Figs. 56a and d
E. coli 10-beta	MR35 + MR41 + MR379	Supplementary Figs. 56a and f
E. coli 10-beta	MR132 + MR343 + MR378	Supplementary Fig. 59b
E. coli 10-beta	MR132 + MR343 + MR380	Supplementary Fig. 59d
E. coli 10-beta	MR132 + MR343 + MR379	Supplementary Fig. 59f
E. coli 10-beta	MR132 + MR343 + MR20	Supplementary Fig. 59h
E. coli 10-beta	LR113 + YR3	Supplementary Fig. 65a
E. coli 10-beta	LR329 + YR3	Supplementary Fig. 65a - Control

E. coli 10-beta	LR7+LR113	Supplementary Fig. 67a
E. coli 10-beta	LR7+LR220	Supplementary Fig. 67b
E. coli 10-beta	LR7+LR329	Supplementary Fig. 67c
E. coli 10-beta	LR7+LR110	Supplementary Fig. 67d
E. coli 10-beta	LR7+LR285+LR309	Supplementary Fig. 69c
E. coli 10-beta	LR7+LR309	Supplementary Fig. 69d
E. coli 10-beta	LR7+LR171	Supplementary Fig. 73
E. coli 10-beta	MR153 + MR158 + MR168	Supplementary Fig. 78a
E. coli 10-beta	MR398 + MR399	Supplementary Fig. 86
E. coli 10-beta	LKsg3: LK4 +RF42 + YLP1	Supplementary Fig. 85
E. coli 10-beta	SG6: LK8 +RF42 + YLP1	Supplementary Fig. 85
E. coli 10-beta	Control: YLP286 +RF42 + LR255	Supplementary Fig. 85
E. coli 10-beta	Control: RF303+LR319<br>LR326+LR319	Supplementary Fig. 86
E. coli 10-beta	Control: LR324+LR319<br>LR364+LR319<br>LR365+LR319<br>LR366+LR319<br>LR367+LR319<br>LR368+LR319<br>LR369+LR319<br>LR370+LR319	Supplementary Fig. 89

17. Supplementary References

1. Medina, G., Juárez, K., Valderrama, B. & Soberón-Chávez, G. Mechanism of Pseudomonas aeruginosa RhlR Transcriptional Regulation of the rhlAB Promoter. *J. Bacteriol.* **185**, 5976–5983 (2003).
2. Daniel, R., Rubens, J. R., Sarpeshkar, R. & Lu, T. K. Synthetic analog computation in living cells. *Nature* **497**, 619–623 (2013).
3. Madar, D., Dekel, E., Bren, A. & Alon, U. Negative auto-regulation increases the input dynamic-range of the arabinose system of Escherichia coli. *BMC Syst. Biol.* **5**, 111 (2011).
4. Nielsen, A. A. K. *et al.* Genetic circuit design automation. *Science* **352**, (2016).
5. Elowitz, M. B., Levine, A. J., Siggia, E. D. & Swain, P. S. Stochastic gene expression in a single cell. *Science (80-.).* **297**, (2002).
6. Swain, P. S., Elowitz, M. B. & Siggia, E. D. Intrinsic and extrinsic contributions to stochasticity in gene expression. *Proc. Natl. Acad. Sci. U. S. A.* **99**, (2002).
7. Ozbudak, E. M., Thattai, M., Kurtser, I., Grossman, A. D. & Van Oudenaarden, A. Regulation of noise in the expression of a single gene. *Nat. Genet.* **31**, (2002).
8. Milo Ron, P. R. *Biology by the Numbers*. *Physical Biology* (2008). doi:10.1142/9781848162013_0010.
9. Ackers, G. K., Johnsontt, A. D. & Shea, M. A. Quantitative model for gene regulation by A phage repressor. *Proc. NatL Acad. Sci. USA* **79**, 1129–1133 (1982).
10. Bintu, L. *et al.* Transcriptional regulation by the numbers: models. *Curr Opin Genet Dev* **15**, 116–124 (2005).
11. Cox, R. S., Surette, M. G., Elowitz, M. B. & Elowitz, M. B. Programming gene expression with combinatorial promoters. *Mol. Syst. Biol.* **3**, (2007).
12. Lee, N. L., Gielow, W. 0 & Wallace, R. G. Mechanism of araC autoregulation and the domains of two overlapping promoters, Pc and PBAD' in the L-arabinose regulatory region ofEscherichia coli. *Biochemistry* **78**, 752–756 (1981).
13. Andersen, J. B. *et al.* New unstable variants of green fluorescent protein for studies of transient gene expression in bacteria. *Appl. Environ. Microbiol.* **64**, 2240–2246 (1998).
14. Tamsir, A., Tabor, J. J. & Voigt, C. A. Robust multicellular computing using genetically encoded NOR gates and chemical 'wiresg'. *Nature* **469**, (2011).
15. Daniel, R., Lu, T. & Rubens, J. Front-End Analog Signal Processing For Cellular Computation. 1–12 (2017).
16. Jarrett, K., Kavukcuoglu, K., Ranzato, M. A. & LeCun, Y. What is the best multi-stage architecture for object recognition? in *IEEE 12th International Conference on Computer Vision* 2146–2153 (IEEE, 2009). doi:10.1109/ICCV.2009.5459469.
17. Nair, V. & Hinton, G. E. Rectified Linear Units Improve Restricted Boltzmann Machines. in *ICML 27th International Conference on Machine Learning* (ICML, 2010).
18. Smooth maximum - Wikipedia.
19. Gardner, T. S., Cantor, C. R. & Collins, J. J. Construction of a genetic toggle switch in Escherichia coli. *Nature* **403**, 339–342 (2000).
20. Anderson, J. C., Voigt, C. A. & Arkin, A. P. Environmental signal integration by a modular and gate. *Mol. Syst. Biol.* **3**, (2007).
21. Jeruzalmi, D. & Steitz, T. A. Structure of T7 RNA polymerase complexed to the transcriptional inhibitor T7 lysozyme. *EMBO J.* **17**, 4101–4113 (1998).
22. Part:BBa B0031 - parts.igem.org.

23. Knuth, D. E. Introduction to combinatorial algorithms and Boolean functions. in *The Art of Computer Programming* 64–74 (Addison-Wesley Professional, 2008).
24. Anderson, J. A. *An introduction to neural networks*. (MIT Press, 1995).
25. Siuti, P., Yazbek, J. & Lu, T. K. Synthetic circuits integrating logic and memory in living cells. *Nat. Biotechnol.* **31**, 448–452 (2013).
26. Bonnet, J., Yin, P., ME, O., P, S. & Endy, D. Amplifying Genetic Logic Gates. *Science* **340**, 599–603 (2013).
27. Snyman, J. A. *PRACTICAL MATHEMATICAL OPTIMIZATION: An Introduction to Basic Optimization Theory and Classical and New Gradient-Based Algorithms*. (Springer, Boston, MA, 2005). doi:https://doi.org/10.1007/b105200.
28. Qian, N. On the momentum term in gradient descent learning algorithms. *Neural Networks* **12**, 145–151 (1999).
29. Daniel, R., Rizik, L. & Daniel, L. Design Principles of Data Converters for Building Large-Scale Gene Networks in Living Cells. in *NanoCom '17 Proceedings of the 4th ACM International Conference on Nanoscale Computing and Communication* (2017). doi:10.1145/3109453.3131226.
30. Walden, R. H. Analog-to-digital converter survey and analysis. *IEEE J. Sel. Areas Commun.* **17**, 539–550 (1999).
31. Avitabile, G., Forti, M., Manetti, S. & Marini, M. On a class of nonsymmetrical neural networks with application to ADC. *IEEE Trans. Circuits Syst.* **38**, 202–209 (1991).
32. Danial, L., Wainstein, N., Kraus, S. & Kvatinsky, S. Breaking Through the Speed-Power-Accuracy Tradeoff in ADCs using a Memristive Neuromorphic Architecture. *IEEE Trans. Emerg. Top. Comput. Intell.* **2**, 396–409 (2018).
33. Selinger, D. W. *et al.* RNA expression analysis using a 30 base pair resolution Escherichia coli genome array. *Nat. Biotechnol.* **18**, 1262–1268 (2000).
34. Georg, J. *et al.* Evidence for a major role of antisense RNAs in cyanobacterial gene regulation. *Mol. Syst. Biol.* (2009) doi:10.1038/msb.2009.63.
35. Güell, M. *et al.* Transcriptome complexity in a genome-reduced bacterium. *Science* **326**, 1268–1271 (2009).
36. Brophy, J. A. N. & Voigt, C. A. Antisense transcription as a tool to tune gene expression. *Mol. Syst. Biol.* (2016) doi:10.15252/MSB.20156540.
37. Chatterjee, A., Drews, L., Mehra, S., Takano, E. & Kaznessis, Y. N. Convergent Transcription in the Butyrolactone Regulon in Streptomyces coelicolor Confers a Bistable Genetic Switch for Antibiotic Biosynthesis. *PLoS One* **6**, 21974 (2011).
38. Chatterjee, A. *et al.* Convergent transcription confers a bistable switch in Enterococcus faecalis conjugation. *Proc. Natl. Acad. Sci.* **108**, 9721–9726 (2011).
39. Fozo, E. M., Hemm, M. R. & Storz, G. Small toxic proteins and the antisense RNAs that repress them. *Microbiol. Mol. Biol. Rev.* **72**, 579–589 (2008).
40. Lou, C. *et al.* Ribozyme-based insulator parts buffer synthetic circuits from genetic context. *Nat Biotechnol* **30**, 1137–1142 (2012).
41. Moon, T. S., Lou, C., Tamsir, A., Stanton, B. C. & Voigt, C. A. Genetic programs constructed from layered logic gates in single cells. *Nature* **491**, 249–53 (2012).
42. Rubens, J. R., Selvaggio, G. & Lu, T. K. Synthetic mixed-signal computation in living cells. *Nat. Commun.* **7**, (2016).
43. Murmann, B. & Boser, B. E. A 12-bit 75-MS/s Pipelined ADC Using Open-Loop Residue Amplification. *IEEE J. Solid-State Circuits* **38**, 2040–2050 (2003).

44. Ferrell, J. E. Signaling Motifs and Weber's Law. *Mol. Cell* **36**, 724–727 (2009).
45. Nilgiriwala, K. S., Joséjiméjoséjiménez, J., Rivera, P. M. & Vecchio, D. Del. Synthetic Tunable Amplifying Buffer Circuit in E. coli. (2014) doi:10.1021/sb5002533.
46. Salis, H. M., Mirsky, E. A. & Voigt, C. A. Automated design of synthetic ribosome binding sites to control protein expression. *Nat. Biotechnol.* **27**, 946–950 (2009).
47. Fleur, T. La, Hossain, A. & Salis, H. M. Automated Model-Predictive Design of Synthetic Promoters to Control Transcriptional Profiles in Bacteria. *bioRxiv* (2021).
48. Alper, H., Fischer, C., Nevoigt, E. & Stephanopoulos, G. Tuning genetic control through promoter engineering. *Proc. Natl. Acad. Sci. U. S. A.* **102**, 12678–12683 (2005).
49. Li, Y. *et al.* Modular construction of mammalian gene circuits using TALE transcriptional repressors. *Nat. Chem. Biol.* **11**, 207–213 (2015).
50. Stanton, B. C. *et al.* Genomic mining of prokaryotic repressors for orthogonal logic gates. *Nat. Chem. Biol.* **10**, 99–105 (2014).
51. Liu, X. *et al.* De novo design of programmable inducible promoters. *Nucleic Acids Res.* **47**, 10452–10463 (2019).
52. Espah Borujeni, A., Mishler, D. M., Wang, J., Huso, W. & Salis, H. M. Automated physics-based design of synthetic riboswitches from diverse RNA aptamers. *Nucleic Acids Res.* **44**, 1–13 (2016).
53. Zucca, S. *et al.* Multi-Faceted Characterization of a Novel LuxR-Repressible Promoter Library for Escherichia coli. *PLoS One* **10**, e0126264 (2015).
54. Cameron, D. E. & Collins, J. J. Tunable protein degradation in bacteria. *Nat. Biotechnol.* **32**, 1276–1281 (2014).
55. Landry, B. P., Palanki, R., Dyulgyarov, N., Hartsough, L. A. & Tabor, J. J. Phosphatase activity tunes two-component system sensor detection threshold. *Nat. Commun.* **9**, 1–10 (2018).
56. Segall-Shapiro, T. H., Meyer, A. J., Ellington, A. D., Sontag, E. D. & Voigt, C. A. A 'resource allocator' for transcription based on a highly fragmented T7 RNA polymerase. *Mol. Syst. Biol.* (2014) doi:10.15252/msb.20145299.
57. Morel, M., Shtrahman, R., Rotter, V., Nissim, L. & Bar-Ziv, R. H. Cellular heterogeneity mediates inherent sensitivity-specificity tradeoff in cancer targeting by synthetic circuits. *Proc. Natl. Acad. Sci. U. S. A.* **113**, 8133–8138 (2016).
58. Hopfield, J. J. & Tank, D. W. Neural computation of decisions in optimization problems. *Biol. Cybern.* **52**, 141–152 (1985).
59. Weinberg, B. H. *et al.* Large-scale design of robust genetic circuits with multiple inputs and outputs for mammalian cells. *Nat. Biotechnol.* **35**, 453–462 (2017).
60. Haykin, S. *Neural Networks: A Comprehensive Foundation*. *Neural Networks* vol. 19 (Pearson Education, 2004).
61. Prezioso, M. *et al.* Training and operation of an integrated neuromorphic network based on metal-oxide memristors. *Nature* **521**, 61–64 (2015).
62. Neftci, E. *et al.* Synthesizing cognition in neuromorphic electronic systems. *Proc. Natl. Acad. Sci. U. S. A.* **110**, E3468--76 (2013).
63. Qian, L., Winfree, E. & Bruck, J. Neural network computation with DNA strand displacement cascades. *Nature* (2011) doi:10.1038/nature10262.
64. Hopfield, J. J. Neural networks and physical systems with emergent collective computational abilities. *Proc. NatL Acad. Sci. USA* **79**, 2554–2558 (1982).
65. Sarpeshkar, R. Analog synthetic biology. *Philos. Trans. A. Math. Phys. Eng. Sci.* **372**,

(2014).
66. Adler, M. & Alon, U. Fold-change detection in biological systems. *Current Opinion in Systems Biology* (2018) doi:10.1016/j.coisb.2017.12.005.
67. Müller, M. *et al.* Designed cell consortia as fragrance-programmable analog-to-digital converters. *Nat. Chem. Biol.* 309–316 (2017) doi:10.1038/nchembio.2281.
68. Kotula, J. W. *et al.* Programmable bacteria detect and record an environmental signal in the mammalian gut. *Proc Natl Acad Sci* **111**, 4838–4843 (2014).
69. Schukur, L., Geering, B., Charpin-El Hamri, G. & Fussenegger, M. Implantable synthetic cytokine converter cells with AND-gate logic treat experimental psoriasis. *Sci. Transl. Med* **7**, (2015).
70. Ye, H. *et al.* Self-adjusting synthetic gene circuit for correcting insulin resistance. *Nat. Publ. Gr.* **1**, 5 (2016).
71. Soma, Y., Tsuruno, K., Wada, M., Yokota, A. & Hanai, T. Metabolic flux redirection from a central metabolic pathway toward a synthetic pathway using a metabolic toggle switch. (2014) doi:10.1016/j.ymben.2014.02.008.
72. Kitney, R. I. & Freemont, P. S. Engineering biology: a key driver of the bio-economy. *Eng. Biol.* **1**, 3–6 (2017).
73. Introduction to Fluorescent Proteins | MicroscopyU.
74. Lutz, R. & Bujard, H. Independent and tight regulation of transcriptional units in Escherichia coli via the LacR/O, the TetR/O and AraC/I1-I2 regulatory elements. *Nucleic Acids Res.* **25**, 1203–1210 (1997).
75. Canton, B., Labno, A. & Endy, D. Refinement and standardization of synthetic biological parts and devices. *Nat. Biotechnol.* **26**, 787–793 (2008).
76. Shopera, T. *et al.* Robust, tunable genetic memory from protein sequestration combined with positive feedback. *Nucleic Acids Res.* **43**, 9086–9094 (2015).
